# Synthesis and Study of Building Blocks with Dibenzo[*b,f*]oxepine: Potential Microtubule Inhibitors

**DOI:** 10.3390/ijms25116155

**Published:** 2024-06-03

**Authors:** Piotr Tobiasz, Filip Borys, Marta Kucharska, Marcin Poterała, Hanna Krawczyk

**Affiliations:** 1 Department of Organic Chemistry, Faculty of Chemistry, Warsaw University of Technology, Noakowskiego 3, 00-664 Warsaw, Poland; fborys@gmail.com (F.B.); marta.kucharska4.dokt@pw.edu.pl (M.K.); marcin.poterala@pw.edu.pl (M.P.); 2 Nencki Institute of Experimental Biology, Polish Academy of Sciences, 3 Pasteur Street, 02-093 Warsaw, Poland

**Keywords:** azo-dibenzo[*b,f*]oxepine derivative, biological activity, photoisomerization, tubulin inhibitor

## Abstract

The synthesis of biphenylmethoxydibenzo[*b,f*]oxepine or photoswitchable fluorinated dibenzo[*b,f*]oxepine derivatives with one or three azo bonds, potential microtubule inhibitors, is described. Our studies provide a concise method for constructing derivatives containing the dibenzo[*b,f*]oxepine skeleton. An analysis of products was run using experimental and theoretical methods. Next, we evaluated the *E*/*Z* isomerization of *azo*-dibenzo[*b,f*]oxepine derivatives, which could be photochemically controlled using visible-wavelength light.

## 1. Introduction

Microtubules are among the most attractive anticancer drug targets and play crucial roles in cellular processes such as morphogenesis, motility, organelle and vesicle trafficking, and chromosome segregation during mitosis [1]. Microtubules are highly dynamic polymers formed in a polymerization process and through the depolymerization of αβ-tubulin heterodimers. In all eukaryotic cells, two globular proteins—*α*- and *β*-tubulin—are present around 50 kDa. These two molecules form an α-/β-tubulin heterodimer with two guanosine triphosphate (GTP) molecules. These are cylindrical polymers. The dimensions of the heterodimer are 4 nm × 5 nm × 8 nm, and its molecular weight is about 100 kDa. These subunits associate longitudinally to form protofilaments, and the protofilaments associate laterally to generate a microtubule. Under certain conditions, these heterodimers attach head to tail and form protein fiber. Self-assembly of protofilaments results in a pipe-like structure known as a microtubule. In cells, microtubules are regulated mainly by their polymerization dynamics. If the microtubule dynamic is disrupted, the cell cycle is arrested in the interphase or during mitosis, between metaphase and anaphase, which leads to cell death. As a result of the association, most often among the 13 protofilaments, a microtubule forms hollow tubes (hollow rods) whose lengths reach several micrometers [2,3]. This type of polymerization results in the two ends differing in their attachment kinetics. At the so-called “plus” end, polymerization and depolymerization are faster than at the “minus” end. The ends of microtubules undergo rapid lengthening and shortening by adding and removing tubulin heterodimers, a phenomenon termed ‘dynamic instability’. Microtubule-targeting agents (MTAs) interacting with tubulin interfere with this dynamic equilibrium, which can result in cell arrest during the interphase and, as a consequence, apoptosis. Although MTAs have shown effectiveness in cancer treatment, their usefulness in clinical applications is limited due to complex and burdensome synthesis or isolation procedures and the adverse side effects associated with their extended and short-term use, mainly potent toxicity to normal cells and primary or acquired resistance.

Photopharmacology, in this context, offers a promising alternative. Photopharmacology is focused on the design, synthesis, research, and use of drugs whose activity can be controlled by light. For conventional drugs, activity is present throughout the body of a patient during the time the drug is present, which may cause adverse effects. After excretion, the build-up of active drugs can have a negative impact on the environment and lead to the emergence of drug-resistant pathogens. Some of the adverse effects can be avoided by using prodrugs, which liberate the active substance at a later stage. Adverse effects and environmental build-up of drugs might be avoided entirely by using drugs whose activity can be controlled reversibly in space and time [4,5]. Azo compounds meet the latter criteria. The presence of double bonds in the *azo* group allows for the absorption of light in the UV-vis range and the presence of interesting optical properties. A *unique feature* is the “clean” and efficient photochemical isomerization around the azo bond when the chromophore absorbs the photon, showing photochromic properties. The absorption spectra of azo bonds depend on the presence of substituents, especially strongly electron-donating and electron-withdrawing substituents. Unsubstituted azobenzene and related compounds have a significant absorption band in the ultraviolet range (λ < 400 nm). In compounds substituted with, for example, amino groups, it is shifted towards longer wavelengths of light [6]. Our last study provided a concise method for constructing the *azo*-dibenzo[*b,f*]oxepine skeleton [7]. It is worth noting that dibenzo[*b,f*]oxepine derivatives show anticancer activity [8]. Herein, we extend our research to studies of novel biphenyl-methoxydibenzo[*b,f*]oxepines or photoswitchable fluorinated dibenzo[*b,f*]oxepine derivatives as potential tubulin polymerization inhibitors. Results from synthesis and molecular docking, as well as the investigation of spectroscopic properties of the obtained compounds, are presented.

## 2. Results and Discussion

Multiple synthetic routes provide access to the dibenzo[*b,f*] oxepine scaffold [9]. One of these has focused primarily on the combination of Ullmann coupling and the Friedele–Crafts reaction [10]. An efficient synthesis is a two-step protocol that involves Ullmann coupling and ring-closing metathesis reactions [11]. The nucleophilic aromatic substitution reaction (SNAr) has been often used for the formation of biaryl ethers [12]. Xanthene ring expansion using Wagner–Meerwein rearrangement or Mn(III)-based oxidative radical rearrangement is also another method [13]. In the synthesis of dibenzo[*b,f*]oxepines, a sequential Heck reaction and Pd-catalyzed etherification have also been applied [14]. Another noteworthy approach is to prepare dibenzo[*b,f*]oxepines with various functional groups via a one-pot cascade reaction [15] under Cu-assisted and Cu-free conditions [16]. Synthetic approaches to naturally occurring dibenzo[*b,f*]oxepines are concerned mainly with the preparation of various bauhinoxepins [17]. In our experiments, we used a two-step synthesis of dibenzo[*b,f*]oxepines that involves the condensation of 2,4-dinitrotoluene with various substituted methoxyaldehydes and subsequent cyclization of the obtained stilbenes [18].

**Scheme 1:**
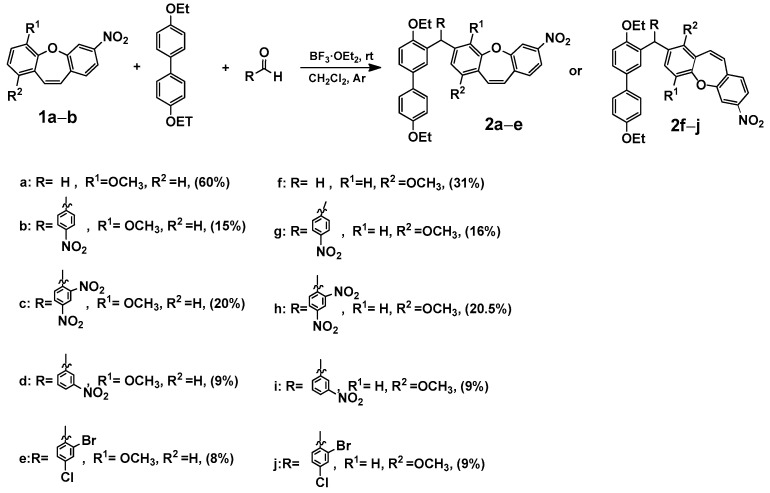
Synthesis of 3-((4,4′-diethoxy-[1,1′-biphenyl]-3-yl)methyl)-1 (**2a**–**e**) or 4-methoxy-7-nitrodibenzo[*b,f*]oxepine derivatives (**2f**–**j**). Product yields are presented in brackets (for more information, see the Supporting Materials).

**Scheme 2:**
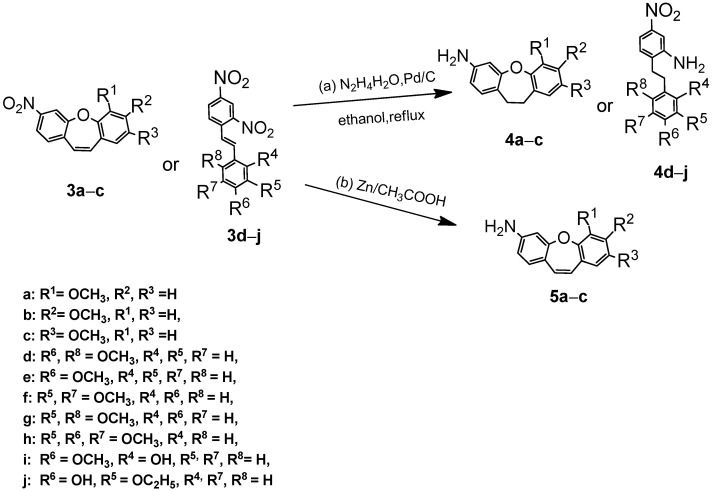
Synthesis of substituted dibenzo[*b,f*]oxepines ((**4a**–**c**) and (**5a**–**c**)) and stilbenes (**4d**–**j**). Product yields are presented in brackets (for more information, see the Supporting Materials).

**Scheme 3:**
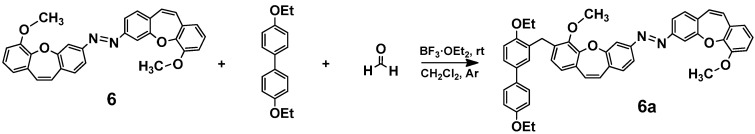
Scheme of *azo*-dibenzo[*b,f*]oxepine dimer (**6**) with 4,4′-diethoxy-1,1′-biphenyl synthesis.

**Scheme 4:**
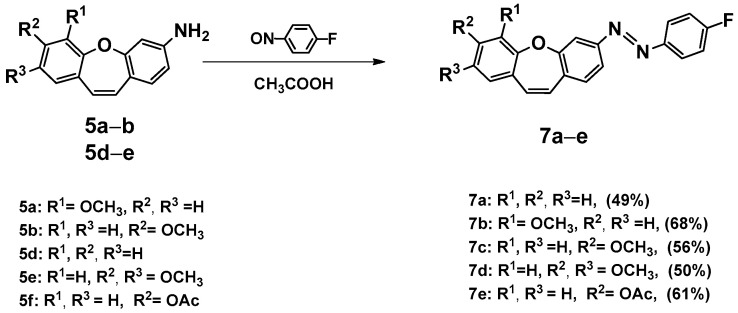
Synthesis of (*E*)-1-(4-fluorophenyl)-2-(methoxydibenzo[*b,f*]oxepin-3-yl)diazenes (**7a**–**e**). Product yields are presented in brackets (for more information, see the Supporting Materials).

**Scheme 5:**
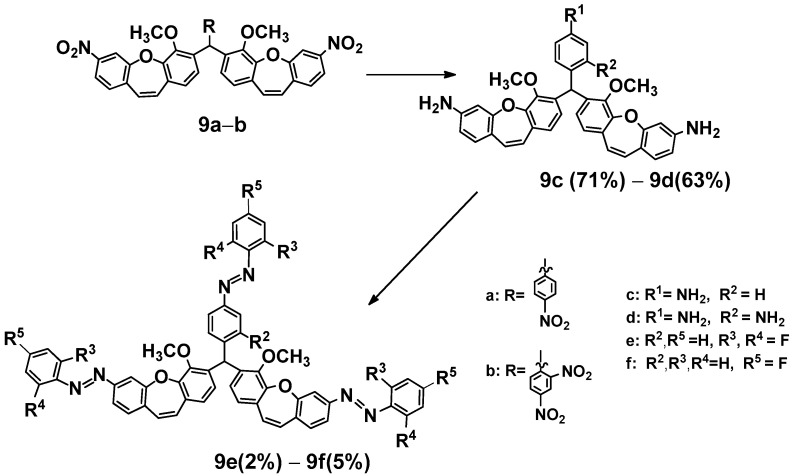
Synthesis of fluoro derivatives of (*E*)-methoxydibenzo[*b,f*]oxepine diazenes (**9e**, **9f**). Product yields are presented in brackets (for more information, see the Supporting Materials).

At the outset of our quest to design building blocks with dibenzo[*b,f*]oxepine for use in photopharmacology, we studied the incorporation of aryl groups in the dibenzo[*b,f*]oxepine framework with formaldehyde. Our previous publication used paraformaldehyde and boron trifluoride diethyl dietherate to obtain new substituted dioxanes, oxanes, cyclic compounds, or dimmers [19]. Encouraged by the success of previous syntheses and in search of new photo-switching connections, we started to examine the reaction of dibenzo[*b,f*]oxepine with biphenyl derivative and with azo switching (Scheme 1, Scheme 2, Scheme 3, Scheme 4 and Scheme 5). Before the synthesis of azo or biphenyl dibenzo[*b,f*]oxepine derivatives, we performed a computational study (see Supplementary Materials) in which we checked whether the arranged compounds were docked to one of the possible binding sites in tubulin, i.e., the colchicine site. Colchicine binds tubulin tightly, but its severe toxicity to normal tissues has hampered its clinical use [20]. There are many studies on the effects of colchicine derivatives, which are summarized in the review article by Kumar et al. [21]. Although numerous analogues of colchicine have been synthesized in the hope of developing novel, useful drugs with more favorable pharmacological profiles, there is still no compound that can be used in cancer therapy. Research published in 2000 by Hamel et al. [22] can be considered the first proposal of an approach based on the structure of compounds interacting with the colchicine site. The colchicine binding site was finally identified in 2004 by Ravelli et al. [23]. They reported the structure of tubulin in complex with *N*-deacetyl-*N*-(2- mercaptoacetyl)colchicine (DAMA-colchicine) and with the RB3 protein stathmin-like domain (PDB ID: 1SA0 [24]).

### 2.1. Computational Aspects

We analyzed the geometry connections of dibenzo[*b,f*]oxepin with biphenyl derivative and the *E*/*Z* isomers in the *azo* molecules (see Supplementary Materials) using density functional theory (DFT) calculations. The optimum ground-state geometry for 3-((4,4′-diethoxy-[1,1′-biphenyl]-3-yl)methyl)-4-methoxy-7-nitrodibenzo[*b,f*]oxepine ((**2a**), (**2f**), (**7b*E***)**,** (**9b*Z***), (**9e**)**,** and (**9f**) and ***E*** and ***Z*** compounds) was calculated using density functional theory (DFT) (Scheme 1, Scheme 4 and Scheme 5). In the calculations, the B3LYP functional, 6–31 g*, and the continuum model (PCM; Gaussian 03W, [25,26] see Supplementary Materials) were used to simulate the effects of the solvent, DMSO.

### 2.2. Molecular Docking

In the next step, we modeled the interaction between (**2a**) and (**2f**) (Scheme 1) and (**7b**) (Scheme 4) and between (**9e**) and (**9f**) and *E* and *Z* isomers (Scheme 5), as well as the colchicine binding sites of α- and β-tubulin (see Supplementary Materials). The molecular docking of compounds (**2a**) and (**2f**) and isomers *E* and *Z* of (**7b**), (**9e**), and (**9f**) into the 3D X-ray structure of tubulin (PDB code: 1SA0) [24] was carried out using Auto-Dock Vina software (https://vina.scripps.edu/, accessed on 27 May 2024) (the Broyden–Fletcher–Goldfarb–Shanno (BFGS) method,) [27]. In the binding mode, compounds (**2a)**, (**2f**), (**7b**), (**9e**), and (**9f**), as well as *E* and *Z* isomers, bind to the colchicine binding site of tubulin via hydrophobic interactions, and hydrogen bonds stabilize binding. The calculated binding energies were used as parameters for the selection of the cluster of docking to be evaluated (see Supplementary Materials), in which the binding mode of the lowest-energy structure was located (selection of the cluster in docking for the lowest-energy structure of the investigated molecule). The selected structures of (**2a**), (**2f**), (**7b**), (**9e**), and (**9f**), as well as *E* and *Z* isomers, had estimated binding free energies of −10.0 kcal/mol, −10.4 kcal/mol, −9.6 kcal/mol, −9.2 kcal/mol, −9.1 kcal/mol, −9.7 kcal/mol, −6.0 kcal/mol, and −11.0 kcal/mol, respectively (binding free energy of control compound colchicine was −8.8 kcal/mol) [28]. The models for all investigated compounds were similar to those between colchicine and the colchicine binding site. In the binding models of (**2a**), (**2f**), (**7b**), (**9e**), and (**9f**), as well as *E* and *Z* isomers, more details revealed that there were some critical roles in the interaction between the tested compounds and tubulin (see Supplementary Materials). Compounds (**2a**), (**2f**), (**7b**), (**9e**), and (**9f**), as well as *E* and *Z* isomers, were embedded in the hydrophobic pocket occupied by the A ring of colchicine (van der Waals contact with Valβ238, Thr β239, Leuβ255, Leuβ252, Leuβ248, Leuβ242, Lysβ352, Lysβ254, and Alaβ250 for (**2a**); Ileβ378, Valβ238, Thr β239, Leuβ255, Leuβ248, Leuβ242, Lysβ352, Lysβ254, and Alaβ250 for (**2b**); Lys352, Lysβ254, Alaβ316, Asnβ258, and Valαβ181 for (**7b*E***); Ileβ378, Valβ318, Lysβ352, Lysβ254, Leuβ255, Leuβ248, Asnβ258, and Alaβ250 for (**7b*Z***); Valβ238, Leuβ255, Leuβ248, Leuβ242, Lysβ352, and Alaβ316 for (**9e*E***); Ileβ378, Valβ318, Alaβ316, Leuβ255, Leuβ248, and Lysβ352 for (**9e*Z***); Lysβ352, Leuβ255, Leuβ248, Leuβ242, and Alaβ250 for (**9f*E***); and Lysβ352, Lysβ254, Alaβ316, Leuβ255, and Leuβ248 for (**9e*Z***)). All contacts for all explored compounds are presented in the Supplementary Materials. These results suggest that the compounds under investigation could interact strongly with tubulin, similarly to colchicine. These encouraging results from docking studies prompted us to synthesize the new molecules presented below.

### 2.3. Synthesis, Spectroscopic Data, and Photochemical Characteristics

In our previous publication [7], we analyzed the changes induced by a substituent in the ring of dibenzo[*b,f*]oxepine using the SCS (substituent-induced chemical shift) parameter [29] for ^13^C NMR chemical shifts. In the reaction study, we took paraformaldehyde as the probe. To expand the scope of this reaction, we examined the reaction in the presence of various aldehydes and 4,4′-diethoxy-1,1′-biphenyl. We synthesized compounds (**2a**–**2j**) consisting of dibenzo[*b,f*]oxepine and a 4,4′-diethoxy-1,1′-biphenyl part, and the coupler was an appropriate aldehyde (Scheme 1).

These results suggested that connecting a new biphenyl substituent with an expanded linker to dibenzo[*b,f*]oxepine is relatively easy. Therefore, we also studied the activity of symmetrical dibenzo [*b,f*]oxepine azo compounds [7]. We first explored the reaction of dibenzo[*b,f*]oxepine (**3a**) with hydrazine/palladium on carbon for the reduction of a nitro group according to the procedure described by Lin and Yang (part (a) in Scheme 2) [30]. We applied them using ethanol as a solvent. Unfortunately, the double bond of (**3a**) was also reduced in this reaction. To test this reduction method, we next examined the reaction of hydrazine and palladium on activated carbon with various dibenzo[*b,f*]oxepines (**3b**, **3c**), as well as the derivatives of stilbene (**3d**–**j**). The results are summarized in Scheme 2. The nitro group and the double bond were reacted in all cases (**4a**–**4j**). To reduce only the amino group (part (b) in Scheme 2), we used Zn in acetic acid acting on nitrodibenzo[*b,f*]oxepines (**3a**–**c**) to convert them to amino derivatives (**5a**–**c**) [31].

Next, according to the procedure described in reference [7], we obtained an *azo* dimer (**6**). In order to obtain mixed azo connections with the dibenzo[*b,f*]oxepine skeleton, we introduced 4,4′-diethoxy-1,1′-biphenyl to the *azo*-dibenzo[*b,f*]oxepine dimer (**6**) using paraformaldehyde (Scheme 3).

Unfortunately, the reaction is not selective. In the mass spectrum of one fraction, peaks can be observed at *m*/*z* (TOF MS ES+) 729.4264, 983.6297, 995.6541, and 1237.8293 (see Supplementary Materials). We concluded that the use of subsequent aldehydes would only increase the number of products. This result contributed to our decision to introduce an azo bond to a nonsymmetric dibenzo[*b,f*]oxepine. We decided to introduce fluoride into the molecule because, as described by Hecht et al. [32,33], it is well-tolerated by the body [34], and its presence may shift the bands in the *UV-vis* spectra towards higher (not harmful for humans) frequencies. We obtained five amines using the optimized conditions for *azo* bond synthesis [31] (**5a**, **5b**, and **5d**–**f**). In the reactions of aminedibenzo[*b,f*]oxepine derivatives (**5a**, **5b**, and **5d**–**f**) with 1-fluoro-4-nitrosobenzene obtained in situ from the oxidation of 4-fluoroaniline were a set of five products (**7a**–**e**). All compounds’ structures were determinate by an analysis of spectra from high-resolution nuclear magnetic resonance spectroscopy (^1^H,^13^C and ^19^F NMR) (see Supplementary Materials).

To obtain a clearer picture of the changes induced by a substituent in (**7a**–**e**), we used the SCS [29] parameter (substituent-induced chemical shifts, Table 1) for ^13^C NMR chemical shifts. The interpretation of the SCS in the ^13^C NMR spectra is a well-established method for investigating the electronic interactions in various molecular systems despite the absence of any simple and general relationship between the chemical shifts and the electron density of a given nucleus [35,36,37]. The data in Table 1 were collected as substituent-induced chemical shifts. For the carbon-labeled i, SCS was defined as the difference between the chemical shift in the parent compound, i.e., HX: SCSi (RX) = δi(RX) − δi(HX). By analyzing these data, we observed that the substituent effect on the ^13^C chemical shift was most significant in its position orthorelative to the methoxy group, namely −17.44 for (**7b**), −14.31 and −13.89 (∆ = 0.42) for (**7c**), −18.19 and −18.22 (∆ = 0.03) for (**7d**), and −6.30 and −6.74 (∆ = 0.44) for (**7e**), which justified the reactivity (**7b**–**7d**) or not (**7e**) (increased of electron density) in this position in response to electrophile attack. Comparing these results to our previous studies on the effect of substituents on methoxy-3-nitrodibenzo[*b,f*]oxepines [7], we can conclude that replacing the nitro group in the 3 position with the azo group does not change the chemical shift (electron density) in the ring with methoxy groups and probably should not affect dibenzo[*b,f*]oxepine properties.

In the next step, we measured ^19^F spectra of (**7a**–**7e**) at ten wavelengths from 390 nm to 610 nm to check which wavelengths produced the most *Z* isomer after exposure. As a solvent, we chose DMSO due to its ability to dissolve polar and nonpolar molecules, which is crucial for the analytical methods used in this study. Additionally, its intermediate polarity allows for a good approximation of organic and aqueous solvents. Furthermore, DMSO is used for stock solution preparation, which is illuminated and then diluted into aqueous systems for biological activity assessment [38,39]. The results obtained for the tested compounds (**7a**–**7e**) are summarized in Table 2.

**Figure 1:**
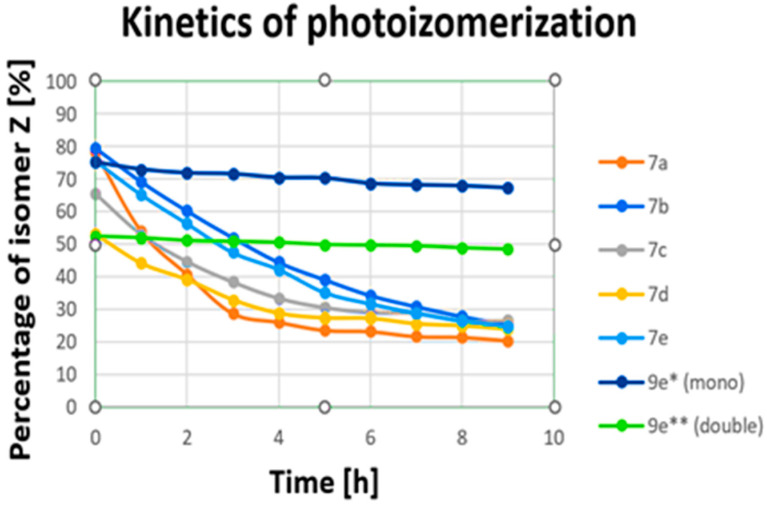
Changes over time (photoisomerization kinetics) in transitions from *Z* to *E* upon irradiation for (**7a**–**7e**) irradiated a 390 nm and for (**9e**) irradiated a 535 nm (for sample concentration of 100 mM in DMSO). * Examined for two *azo* bonds from dibenzo[*b,f*]oxepine; ** examined for one *azo* stilbene.

We can observe that for electromagnetic radiation in the wavelength range of 430 nm to 610 nm a, transitions from *E* to *Z* of about 44–0% were obtained. The best results for all compounds were achieved at wavelengths of 390 nm and 400 nm (79.24–52.63%), corresponding to near-ultraviolet and visible ranges. Then, we measured changes over time (transitions from Z to *E* upon irradiation with 390 nm) for all (**7a**–**7e**) compounds (Figure 1, see Supplementary Materials). After nine hours, all (**7a**–**7e**) compounds changed configuration from Z to *E* to levels above 20% (from 26.58% to 20.47%). A small amount of the *Z* isomer remained after 9 h. However, energy photons from the *UV-vis* range are prone to both scattering in tissue and absorption by endogenous chromophores [40]. They also contribute to the photodamage of cells [41,42]. These processes severely limit the depth of penetration and are responsible for the toxicity of *UV* light. Fixation, plasma membrane permeabilization, and cytoskeleton destruction can be observed upon irradiation with shorter wavelengths [41]. Unfortunately, the obtained compounds (**7a**–**7e**) change configurations most effectively when exposed to *UV* light, which is toxic for cells. The most obvious approach for designing visible-light switches is to extend the π system of a known compound in order to lower the HOMO–LUMO gap and, hence, red-shift its absorption [32,33,43]. Considering this and looking for new, better-functioning switches, we synthesized compounds with more than one *azo* bond.

We obtained compounds (**9c**, **9d**) with three amine groups in reactions with nitro compounds (**9a**) or (**9b**) with acetic acid and zinc (Scheme 5). Then, two products ((**9e**) or (**9f**)) were obtained from (**9c**) or (**9d**) with the appropriate nitrosobenzenes generated in situ by oxidation of substituted anilines. Furthermore, it is known from our current and previous [44] experiments that dibenzo[*b,f*]oxepine and *azo* stilbene can dock into the colchicine binding site of α- and β-tubulin.

In the next step, we also measured the ^19^F spectra of (**9e**, **9f**) upon irradiation of 10 wavelengths from 390 nm to 610 nm in DMS to check which wavelengths produced the most *Z* isomer after exposure. The obtained results are summarized in Table 2a,b. Signals from part of the dibenzo[*b,f*]oxepine (equivalent signals) and from *azo* stilbene rings can be observed for (**9e**) and (**9f**). These signals are easy to interpret because they are in a 2:1 ratio (see Supporting Materials). From Table 2, we can see that for electromagnetic radiation of dibenzp[*b,f*]oxepine parts in the wavelength range 390 nm to 400 nm, transitions from *E* to Z of above 60% are obtained (**9e**: 63.18, 61.99; **9f**: 73.49, 70.62). In part of the *azo* stilbene, a maximum a transitions from *E* to Z of above 69% is observed in the range of green light from 505 nm to 535 nm for (**9e**). However, for (**9f**), a transition from *E* to Z is in the range of violet light 44.71% of the time. We also measured changes over time (transitions from *Z* to *E* upon irradiation with 535 nm) for compound (**9e**) (Figure 1, see Supplementary Materials). Notably, within 9 h, the amount of Z isomer in the dibenzo[*b,f*]oxepine parts, as well as in the *azo* stilbene group, decreased slightly (52.49–48.47% (Δ7.7%) and 75.17–67.30% (Δ10.5%), respectively). Analyzing Table 2, we can draw the following conclusions: For the fluorine substituent in the para position connected to dibenzo[*b,f*]oxepine, both for mono derivatives (**7a**–**7e**) and for the dimers (**9f**) and (**9e**), the maximum transitions from *E* to *Z* in the dibenzo[*b,f*]oxepine part occur for violet light. However, under irradiation with green light (535 nm), the maximum transition from *E* to *Z* is obtained in the part of *azo* 2,6-difluorostibene (**9e**). 

In the next step, we checked whether the π–π* and n–π* bands of *E* and *Z* isomers were separated within the *UV-vis* spectra to allow for selective excitation of the two isomers in the visible range of the spectrum(see Supplementary Materials). The action of photopharmaceuticals containing skeletal fragments of azobenzene as a functionalizing unit allows for control of biological functions with precision in space and time [45,46]. That is why we measured *UV-vis* absorption spectra for selected compounds (**7b**) and (**9e**) at concentrations ranging from 20 to 500 μm in dimethyl sulfoxide (DMSO) in light λ 390 nm and λ 535 nm, respectively (see the Supporting Materials). For compounds (**7b**) and (**9e**), we observed a strong π−π* transition band at short wavelengths (λπ−π* (**7b*E***) ≈ 370 nm; for (**7b*Z***), 312 nm band; for isomers ***E/Z*** (**9e**), 340/360 nm respectively). Only for isomer (**7b*Z***) were the π−π* and n−π* bands separated. Unfortunately, the intensity of bands in the case of *Z* isomers of (**7b**) and (**9e**) was lower than for the *E* isomer (ε***Z***: about 2, 80 μm samples; ε***E***: about 3, 80 μm samples), which indicates that the probability of the transition decreases because of the energy of the transition’s changes.

However, despite the lack of separation of the bands for (**9e**), we can conclude (based on ^19^F NMR spectra) that irradiation with a wavelength of 535 nm causes the (**9e*E***)/(**9e*Z***) switch in approximately 75% of cases for the azo stilbene part and approximately 52% of cases in the azo bonds of dibenzo[*b,f*]oxepines. The outcomes of our investigations open novel areas for future research on the use of switches with multiple azo bonds and increase the available data on useful switches for photopharmacology.

## 3. Conclusions

In this study, we successfully designed and synthesized a potential set of novel photoswitchable dibenzo[*b,f*]oxepine azobenzene hybrid-based potential microtubule dynamic-disrupting agents. Our study provides a concise method for constructing a dibenzo[*b,f*]oxepine-azobenzene hybrid with one or three azo bonds. Furthermore, we explored the switchable properties of the obtained compounds. In summary, we have proven that (**9e**) provides a basis for further improvement and development of the novel potential photoswitchable dibenzo[*b,f*]oxepine-based microtubule polymerization inhibitors. Therefore, further biological research is ongoing on (**9e**) as a molecular photoswitch for use in photopharmacology.

## Figures and Tables

**Table 1:** Carbon-13 chemical shifts for (*E*)-1-(4-fluorophenyl)-2-(dibenzo[*b,f*]oxepin-3-yl)diazenes and substituent-induced chemical shifts for its methoxy-substituted derivatives.

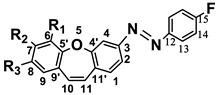																		
	C1	C2	C3	C4	C4′	C5′	C6	C7	C8	C9	C9′	C10	C11	C11′	C12	C13	C14	C15
(**7a**) R1–R3 = H,	129.68	120.85	153.67	114.56	157.87	156.98	121.52	130.28	125.14	129.48	130.35	131.46	129.33	133.28	149.13	124.98	116.09	164.49
(**7b**) R2,R3 = H, R1 = OCH_3_	−0.16	−0.05	−0.04	1.47	−0.11	−11.91	30.38	−17.44	−5.32	−4.49	1.27	−0.13	0.03	0.24	0.04	0.00	−0.05	−0.05
(**7c**) R1,R3 = H, R2 = OCH_3_	−0.11	−0.01	−0.02	0.13	−0.41	1.25	−14.31	31.78	−13.89	0.79	−7.10	−0.19	−2.26	0.44	0.23	−0.02	−0.02	0.06
(**7d**) R1 = H, R2,R3 = OCH_3_	−0.12	0.16	−0.14	−0.36	−0.23	−6.32	−18.19	20.45	21.02	−18.22	−8.38	−0.24	−1.51	0.28	0.01	−0.04	0.00	−0.03
(**7e**) R1,R3 = H, R_2_ = OCOCH_3_	0.01	0.44	−0.02	−0.18	−0.58	0.46	−6.30	21.87	−6.74	0.27	−2.19	−0.89	−0.19	−0.21	−0.03	0.01	−0.01	0.00

**Table 2:** (a) Photostationary state (PSS) compositions for dibenzo[*b,f*]oxepin-diazenes (**7a**–**7e**), (**9e**), and (**9f**) determined by ^19^F NMR analysis (c ≈ 100 mM in DMSO-*d*6), (b) results from (a) presented in a bar chart.

(**a**)									
	**Percentage of Isomer Z [%]**								
**Wavelength [nm]**	**Compound**								
	***7a***	***7b***	***7c***	***7d***	***7e***	*9e* ** (double)	*9e* * (mono)	*9f* ** (double)	*9f* * (mono)
390 nm	77.66	79.24	67.23	52.63	73.48	63.18	29.9	73.49	44.71
400 nm	77.03	74.08	65.97	53.8	68.01	61.99	26.01	70.62	30.61
430 nm	36.39	16.16	42.67	29.92	8.28	16.98	8.62	22.47	8.69
470 nm	27.03	25.54	43.73	39.31	26.46	38.15	51.85	23.96	31.05
505 nm	22.61	22.12	30.39	31.47	21.92	50.31	70.49	20.98	32.39
515 nm	22.48	22.02	32.22	31.8	22.07	51.17	69.3	21.53	32.26
525 nm	21.18	17.35	28.43	26.77	19.24	53.88	69.33	17.14	20.15
535 nm	23.28	19.88	31.57	28.76	21.21	52.49	75.17	19.33	28.68
590 nm	0.96	2.32	4.17	3.38	3.06	28.15	14.51	1.88	0.83
610 nm	0	0	0	0	0	5.2	1.92	0	0
(**b**)									
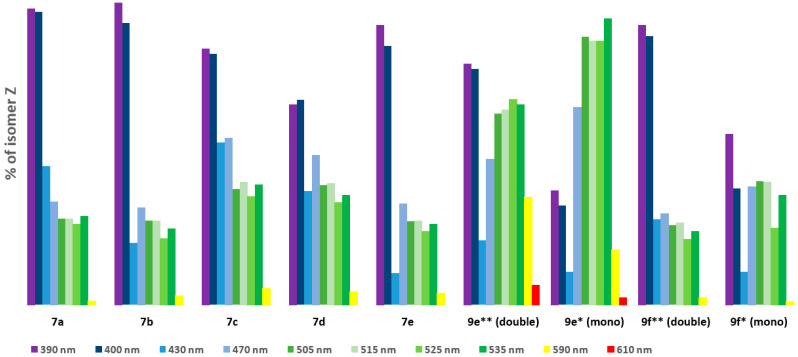									

* Examined for two *azo* bonds from dibenzo[*b,f*]oxepine; ** examined for one *azo* stilbene.

## Data Availability

Data are contained within the article and Supplementary Materials.

## Supplementary Materials

The following supporting information can be downloaded at: https://www.mdpi.com/article/10.3390/ijms25116155/s1. The following are available online [47].

## References

[1] Fojo T. (2008). The Role of Microtubules in Cell Biology, Neurobiology, and Oncology.

[2] Akhmanova A., Steinmetz M.O. (2015). Control of microtubule organization and dynamics: Two ends in the limelight. Nat. Rev. Mol. Cell Biol..

[3] Brouhard G.J., Rice L.M. (2018). Microtubule Dynamics: An interplay of biochemistry and mechanics. Nat. Rev. Mol. Cell Biol..

[4] Velema W.A., Szymanski W., Feringa B.L. (2014). Photopharmacology: Beyond Proof of Principle. J. Am. Chem. Soc..

[5] Lerch M., Hansen M., van Dam G., Szymański W., Feringa B.L. (2016). Emerging Targets in Photopharmacology. Angew. Chem..

[6] Barrett C.J., Mamiya J.I., Yagerc K.G., Ikeda T. (2007). Photo-mechanical effects in azobenzene-containing soft materials. Soft Matter..

[7] Tobiasz P., Borys F., Borecka M., Krawczyk H. (2021). Synthesis and investigations of building blocks with dibenzo[*b,f* ]oxepine for use in photopharmacology. Int. J. Mol. Sci..

[8] Garbicz D., Mielecki D., Wrzesiński M., Pilżys T., Marcinkowski M., Piwowarski J., Dębski J., Palak E., Szczeciński P., Krawczyk H. (2018). Evaluation of anti-cancer activity of stilbene and methoxydibenzo[*b,f*]oxepin derivatives. Curr. Cancer Drug Targets.

[9] Krawczyk H. (2023). Dibenzo[*b,f*]oxepine Molecules Used in Biological Systems and Medicine. Int. J. Mol. Sci..

[10] Harris T.W., Smith H.E., Mobley P.L., Manier D.H., Sulser F. (1982). Affinity of 10-(4-methylpiperazino)dibenz[*b,f*]oxepins for clozapine and spiroperidol binding sites in rat brain. J. Med. Chem..

[11] Bharath Y., Thirupathi B., Ranjit G., Mohapatra D.K. (2013). An Efficient Synthesis of Dibenzo[*b,f*]oxepins by Ring-Closing Metathesis. Asian J. Org. Chem..

[12] Moreno D.R.R., Giorgi G., Salas C.O., Tapia R.A. (2013). New Short Strategy for the Synthesis of the Dibenz[*b,f*]oxepin Scaffold. Molecules.

[13] Storz T., Vangrevelinghe E., Dittmar P. (2005). Synthesis and Wagner-Meerwein Rearrangement of 9-(α-Hydroxyalkyl) xanthenes to 10-Substituted Dibenz [*b,f*] oxepins: Scope, Limitations and ab initio Calculations. Synthesis.

[14] Heesgaard J.T., Mogens L., Morten J., Broendsted N.M. (2012). Three-Step Synthesis of (Thio)xanthene and Dibenzothiepine/Dibenzoxepine by an Intramolecular Mizoroki-Heck Reaction of Diaryl (Thio)Ethers. Synlett.

[15] Choi Y.L., Lim H.S., Lim H.J., Heo J.-N. (2012). One-Pot Transition-Metal-Free Synthesis of Dibenzo[*b,f*]oxepins from 2-Halobenzaldehydes. Org. Lett..

[16] Wang Y., Chen Y., He Q., Xie Y., Yang C. (2013). Copper-Assisted/Copper-Free Synthesis of Functionalized Dibenzo[*b,f*]oxepins and Their Analogs via a One-Pot Tandem Reaction. Helv. Chim. Acta.

[17] Koichi N., Ken N., Yui A., Tadashi K. (2011). Total Synthesis of Bauhinoxepin J: A Biologically Active Dibenzo[*b,f*]oxepin Isolated from Bauhinia purpurea. Eur. J. Org. Chem..

[18] Krawczyk H., Wrzesiński M., Mielecki D., Szczeciński P., Grzesiuk E. (2016). Synthesis of derivatives of methoydibenzo[*b,f*]oxepine in the presence of sodium azide. Tetrahedron.

[19] Tobiasz P., Poterała M., Jaskowska E. (2018). Synthesis and investigation of new cyclic molecules using the stilbene scaffold. RSC Adv..

[20] Zhou J., Giannakakou P. (2005). Targeting Microtubules for Cancer Chemotherapy. Curr. Med. Chem. Anti-Cancer Agents.

[21] Kumar A., Sharma P.R., Mondhe D.M. (2017). Potential anticancer role of colchicine-based derivatives: An overview. Anti-Cancer Drugs.

[22] Bai R., Covell D.G., Pei X.F., Ewell J.B., Nguyen N.Y., Brossi A., Hamel E. (2000). Mapping the binding site of colchicinoids on beta -tubulin. 2-Chloroacetyl-2-demethylthiocolchicine covalently reacts predominantly with cysteine 239 and secondarily with cysteine 354. J. Biol. Chem..

[23] Ravelli R.B., Gigant B., Curmi P.A., Jourdain I., Lachkar S., Sobel A., Knossow M. (2004). Insight into tubulin regulation from a complex with colchicine and a stathmin-like domain. Nature.

[24] RCSB Protein Data Bank—RCSB PDB. http://www.rcsb.org/pdb/home/home.do.

[25] Frisch M.J., Trucks G.W., Schlegel H.B., Scuseria G.E., Robb M.A., Cheeseman J.R., Montgomery J.A., Vreven T., Kudin K.N., Burant J.C. Gaussian 03, Revision E.01, Gaussian Inc., Wallingford. https://gaussian.com/citation/.

[26] Tomasi J., Mennucci B., Cammi R. (2005). Quantum Mechanical Continuum Solvation Models. Chem. Rev..

[27] Trott O., Olson A.J. (2010). AutoDock Vina: Improving the speed and accuracy of docking with a new scoring function, efficient optimization and multithreading. J. Comput. Chem..

[28] Luo Y., Qiu K.M., Lu X., Liu K., Fu J., Zhu H.L. (2011). Synthesis, biological evaluation, and molecular modeling of cinnamic acyl sulfonamide derivatives as novel antitubulin agents. Bioorg. Med. Chem..

[29] Craik D.J. (1984). Substituent Effects on Nuclear Shielding. Ann. Rep. NMR Spectrosc..

[30] Lin C.-K., Yang J.-S. (2013). Fluorescence response of TICT-active aminostilbenes to copper(II) ions: Redox reaction vs ion recognition. Res. Chem. Intermed..

[31] Borys F., Tobiasz P., Joachimiak E., Fabczak H., Krawczyk H. (2023). Systematic studies on *anti*-cancer evaluation of stilbene and dibenzo[*b,f*]oxepine derivatives. Molecules.

[32] Bleger D., Hecht S. (2015). Visible-Light-Activated Molecular Switches. Angew. Chem. Int. Ed..

[33] Bleger D., Schwarz J., Brouwer A.M., Hecht S. (2012). o-Fluoroazobenzenes as Readily Synthesized Photoswitches Offering Nearly Quantitative Two-Way Isomerization with Visible Light. J. Am. Chem. Soc..

[34] Agnetta L., Bermudez M., Riefolo F., Matera C., Claro E., Messerer R., Littmann T., Wolber G., Holzgrabe U., Decker M. (2019). Fluorination of Photoswitchable Muscarinic Agonists Tunes Receptor Pharmacology and Photochromic Properties. J. Med. Chem..

[35] Kara Y.S. (2015). Substituent effect study on experimental 13C NMR chemical shifts of (3-(substitutedphenyl)-cis-4,5-dihydroisoxazole-4,5-diyl)bis(methylene)diacetate derivatives. Spectrochim. Acta A Mol. Biomol. Spectrosc..

[36] Holik M. (1992). Second-order regression of ^13^C substituent chemical shifts with Taft’s sigma constants. Magn. Reson. Chem..

[37] Ewing D.F. (1979). 13C substituent effects in monosubstituted benzenes. Magn. Reson. Chem..

[38] Wegener M., Hansen M.J., Driessen A.J.M., Szymanski W., Feringa B.L. (2017). Photocontrol of Antibacterial Activity: Shifting from 531 UV to Red Light Activation. J. Am. Chem. Soc..

[39] Wutz D.-F., Gluhacevic D., Chakrabarti A., Schmidtkunz K., Robaa D., Erdmann F., Romier C., Sippl W., Jung M., König B. (2017). Photochromic Histone Deacetylase Inhibitors Based on Dithienylethenes and Fulgimides. Org. Biomol. Chem..

[40] Kalka K., Merk H., Mukhtar H.J. (2000). Photodynamic therapy in dermatology. J. Am. Acad. Dermatol..

[41] Brash D.E., Rudolph J.A., Simon J.A., Lin A., McKenna G.J., Baden H.P., Halperin A.J., Ponten J. (1991). A role for sunlight in skin cancer: UV-induced p53 mutations in squamous cell carcinoma. Proc. Natl. Acad. Sci. USA.

[42] Wäldchen S., Lehmann J., Klein T., van de Linde S., Sauer M. (2015). Light-induced cell damage in live-cell super-resolution microscopy. Sci. Rep..

[43] Alachouzos G., Schulte A.M., Mondal A., Szymanski W., Feringa B.L. (2022). Computational Design, Synthesis, and Photochemistry of Cy7-PPG, an Efficient NIR-Activated Photolabile Protecting Group for Therapeutic Applications. Angew. Chem. Int. Ed..

[44] Borys F., Tobiasz P., Sobel J., Krawczyk H. (2022). Synthesis and Study of Dibenzo[*b,f*]oxepine Combined with Fluoroazobenzenes—New Photoswitches for Application in Biological Systems. Molecules.

[45] Kirchner S., Pianowski Z. (2022). Photopharmacology of antimitotic agents. Int. J. Mol. Sci..

[46] Hüll K., Morstein J., Trauner D. (2018). In Vivo Photopharmacology. Chem. Rev..

[47] Pettersen E.F., Goddard T.D., Huang C.C., Couch G.S., Greenblatt D.M., Meng E.C., Ferrin T.E. (2004). UCSF Chimera-a visualization system for exploratory research and analysis. J. Comput. Chem..

