# Supplementary material for: Synthesis and Study of Building Blocks with Dibenzo[b,f]oxepine: Potential Microtubule Inhibitors
Source: Int J Mol Sci. 2024 Jun 3;25(11):6155. doi: 10.3390/ijms25116155 (PMC11172465; doi:10.3390/ijms25116155)
Supplement: Supplementary file 1 [file ijms-25-06155-s001.zip › ijms-2938257-supplementary.pdf]

Synthesis and study of building blocks with dibenzo[*b,f*]oxepine, potential microtubule inhibitors

Piotr Tobiasz,*[1] Filip Borys, [1,2] Marta Kucharska,[1] Marcin Poterała[1] and Hanna Krawczyk,*[1]

1 Department of Organic Chemistry, Faculty of Chemistry, Warsaw University of Technology, Noakowskiego 3,00-664 Warsaw, Poland. hkraw@ch.pw.edu.pl

2 Laboratory of Cytoskeleton and Cilia Biology Nencki Institute of Experimental Biology of Polish Academy of Sciences, 3 Pasteur Street, 02-093Warsaw, Poland.

* Correspondence: hanna.krawczyk@pw.edu.pl, piotr.tobiasz.dokt@pw.edu.pl

Supplementary Materials

Table of Contents:

1. **Computational aspects and molecular docking** 2
2. **Experimental section** 28
3. **NMR spectra of obtained compounds** 32
4. **UV-VIS spectra of 7b and 9f E/Z isomers** 131

References 133

1. Computational aspects

The optimum ground-state geometry for (**2a**, **2f** and **7b*E*** and **7b*Z***, **9e** and **9f** ***E*** and ***Z***) compounds was calculated using the density functional theory (DFT). In calculation, the B3LYP functional and 6-31G* basis set was employed and the continuum model (PCM; Gaussian 03W) [1,2] was used to simulate the effects of the solvent. All the calculations were performed on a server equipped with a 16 quad-core XEON (R) CPU E7310 processor operating at 1.60 GHz. The operating system was Open SUSE 10.3. in DMSO as a solvent. We have performed vibration analysis for the optimized geometry of (**2a**, **2f** and **7b*E*** and **7b*Z***, **9e** and **9f** ***E*** and ***Z***) and obtained no negative frequencies in the potential energy surface for calculated molecules.

- The calculated coordinates of (**2a**) (the part of calculated log file) -

```
     Item               Value     Threshold  Converged?
 Maximum Force            0.000064     0.000450     YES
 RMS     Force            0.000017     0.000300     YES
 Maximum Displacement     0.001416     0.001800     YES
 RMS     Displacement     0.000280     0.001200     YES
    Optimization completed.
                         Standard orientation:
 ---------------------------------------------------------------------
 Center     Atomic     Atomic              Coordinates (Angstroms)
 Number     Number      Type              X           Y           Z
 ---------------------------------------------------------------------
    1          6           0       -1.740988    1.329899   -0.666846
    2          6           0       -2.893774    0.548933   -0.628915
    3          6           0       -1.832519    2.686347   -0.283646
    4          6           0       -4.142645    1.053272   -0.217435
    5          6           0       -3.061260    3.214506    0.125228
    6          6           0       -4.195721    2.401576    0.158606
    7          6           0       -5.349708    0.190929   -0.188011
    8          6           0       -5.274021   -1.165218    0.161373
    9          6           0       -6.402599   -1.987435    0.189984
   10          6           0       -7.657031   -1.455610   -0.133000
   11          6           0       -6.622643    0.701879   -0.510159
   12          6           0       -7.755619   -0.099800   -0.482216
   13          6           0       -0.418115    0.758860   -1.130410
   14          6           0        0.605120    0.522179   -0.027893
   15          6           0        2.555776    0.111305    2.005592
```

16	6	0	2.935526	0.390604	0.680502
17	6	0	3.516830	-0.131842	3.077117
18	8	0	4.264682	0.637417	0.350879
19	6	0	4.789239	-0.576588	2.974593
20	6	0	5.509071	-0.922994	1.754501
21	6	0	5.191852	-0.378806	0.488881
22	6	0	6.582928	-1.833565	1.820816
23	6	0	7.303995	-2.207678	0.694915
24	6	0	5.899148	-0.738106	-0.650236
25	6	0	6.948421	-1.650754	-0.533073
26	6	0	1.980464	0.575838	-0.327634
27	6	0	1.180160	0.051518	2.285722
28	6	0	0.229137	0.253425	1.292263
29	8	0	-0.672140	3.398732	-0.353603
30	6	0	0.741209	5.297088	-0.118351
31	6	0	-0.675068	4.773772	0.041311
32	8	0	-8.825921	-2.160140	-0.138036
33	6	0	-10.208513	-4.084994	0.088637
34	6	0	-8.790567	-3.552051	0.193449
35	7	0	7.700039	-2.017100	-1.730341
36	8	0	8.621916	-2.829268	-1.611584
37	8	0	7.374727	-1.499325	-2.802614
38	8	0	2.405015	0.890068	-1.600138
39	6	0	2.541860	-0.235328	-2.474217
40	1	0	-2.823011	-0.485447	-0.957505
41	1	0	-3.144993	4.252069	0.428205
42	1	0	-5.132502	2.828293	0.506519
43	1	0	-4.315456	-1.592909	0.442742
44	1	0	-6.292325	-3.027716	0.474585
45	1	0	-6.722392	1.741440	-0.810556
46	1	0	-8.731336	0.301534	-0.740831
47	1	0	-0.613530	-0.192698	-1.644613
48	1	0	0.037979	1.423543	-1.870961
49	1	0	3.117096	-0.014936	4.082870
50	1	0	5.322384	-0.787488	3.899134
51	1	0	6.849444	-2.253191	2.786842
52	1	0	8.127113	-2.908314	0.755824
53	1	0	5.658383	-0.288386	-1.604431

54	1	0	0.862264	-0.158178	3.303902
55	1	0	-0.826232	0.218207	1.543689
56	1	0	0.786664	6.349572	0.181183
57	1	0	1.435280	4.725954	0.506028
58	1	0	1.067845	5.217620	-1.160083
59	1	0	-1.377747	5.339008	-0.586728
60	1	0	-1.010296	4.857654	1.084318
61	1	0	-10.225756	-5.151347	0.337518
62	1	0	-10.873019	-3.556961	0.780097
63	1	0	-10.595240	-3.959868	-0.927846
64	1	0	-8.119888	-4.081037	-0.497885
65	1	0	-8.397008	-3.681242	1.211343
66	1	0	2.907872	0.157886	-3.425414
67	1	0	3.261483	-0.961611	-2.077189
68	1	0	1.580739	-0.738578	-2.636276

After PCM corrections, the SCF energy is -1742.85245015 a.u.

Visualization of calculated geometry of (**2a**) :

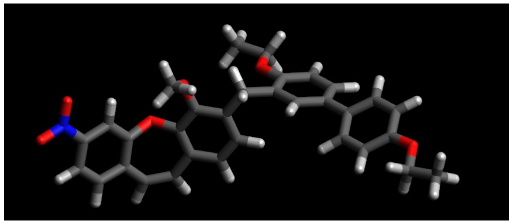

- The calculated coordinates of (**2f**) (the part of calculated log file) -

Item	Value	Threshold	Converged?
Maximum Force	0.000094	0.000450	YES
RMS Force	0.000017	0.000300	YES
Maximum Displacement	0.000250	0.001800	YES
RMS Displacement	0.000078	0.001200	YES

Optimization completed.

Standard orientation:

Center Number	Atomic Number	Atomic Type	Coordinates (Angstroms) X	Y	Z
1	6	0	-1.771673	2.261855	0.746088
2	6	0	-2.693311	1.222182	0.636845
3	6	0	-2.009441	3.452650	0.027654
4	6	0	-3.848157	1.310202	-0.161059

5	6	0	-3.156460	3.569762	-0.765993
6	6	0	-4.056797	2.508191	-0.858199
7	6	0	-4.804566	0.179553	-0.256388
8	6	0	-4.368297	-1.152999	-0.219987
9	6	0	-5.257742	-2.226337	-0.305959
10	6	0	-6.630297	-1.981729	-0.437055
11	6	0	-6.190005	0.399482	-0.387436
12	6	0	-7.087703	-0.655557	-0.478543
13	6	0	-0.539084	2.111080	1.625821
14	6	0	1.483729	2.162057	0.090320
15	6	0	1.873854	-0.613652	0.329493
16	6	0	2.711943	0.175684	-0.477985
17	6	0	2.009774	-2.064020	0.437165
18	8	0	3.677580	-0.412418	-1.293674
19	6	0	3.133471	-2.800919	0.289409
20	6	0	4.477316	-2.302309	0.015470
21	6	0	4.713727	-1.098630	-0.684415
22	6	0	5.598978	-3.033877	0.453440
23	6	0	6.895992	-2.587252	0.238801
24	6	0	6.000467	-0.627096	-0.903547
25	6	0	7.078389	-1.380385	-0.436646
26	6	0	2.530359	1.547720	-0.595482
27	6	0	0.830125	0.047544	1.020712
28	6	0	0.618055	1.429748	0.911620
29	8	0	-1.073346	4.442120	0.166762
30	6	0	-0.159624	6.628822	-0.078473
31	6	0	-1.303396	5.707096	-0.462533
32	8	0	-7.590532	-2.947730	-0.528948
33	6	0	-8.445804	-5.169740	-0.564410
34	6	0	-7.190603	-4.320565	-0.469746
35	7	0	8.434825	-0.893960	-0.677707
36	8	0	9.377512	-1.571731	-0.259152
37	8	0	8.571671	0.171635	-1.285165
38	8	0	-0.034859	-0.707337	1.790819
39	6	0	0.435070	-0.998653	3.114408
40	1	0	-2.506449	0.317955	1.209499
41	1	0	-3.350458	4.476297	-1.327837
42	1	0	-4.923054	2.614040	-1.505576

43	1	0	-3.305374	-1.365370	-0.143621
44	1	0	-4.869578	-3.238352	-0.281715
45	1	0	-6.572805	1.416523	-0.394421
46	1	0	-8.155055	-0.475706	-0.570498
47	1	0	-0.213542	3.098131	1.964838
48	1	0	-0.812455	1.531661	2.511107
49	1	0	1.322659	3.230351	-0.017728
50	1	0	1.102619	-2.585156	0.730482
51	1	0	3.062786	-3.870209	0.477686
52	1	0	5.437987	-3.971405	0.978285
53	1	0	7.753712	-3.152850	0.580239
54	1	0	6.159400	0.293258	-1.450943
55	1	0	3.190925	2.114411	-1.244037
56	1	0	-0.304437	7.612375	-0.537928
57	1	0	-0.112866	6.756847	1.007693
58	1	0	0.798588	6.225997	-0.422608
59	1	0	-1.352396	5.575909	-1.552265
60	1	0	-2.266442	6.115471	-0.126791
61	1	0	-8.181924	-6.231804	-0.522860
62	1	0	-9.126229	-4.948696	0.264203
63	1	0	-8.972237	-4.980141	-1.505428
64	1	0	-6.502949	-4.543184	-1.297557
65	1	0	-6.657751	-4.510915	0.472325
66	1	0	1.372547	-1.566341	3.085560
67	1	0	0.589415	-0.076733	3.688714
68	1	0	-0.342370	-1.600387	3.589659

After PCM corrections, the SCF energy is -1742.85713508 a.u.

Visualization of calculated geometry of (**2f**) :

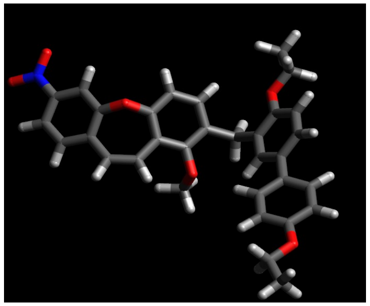

- The calculated coordinates of (**7b-*E***) (the part of calculated log file) -

Item	Value	Threshold	Converged?
Maximum Force	0.000193	0.000450	YES
RMS Force	0.000024	0.000300	YES
Maximum Displacement	0.000012	0.001800	YES

RMS Displacement 0.000003 0.001200 YES
Optimization completed

Standard orientation:

Center Number	Atomic Number	Atomic Type	Coordinates (Angstroms) X	Y	Z
1	6	0	-4.988701	1.993893	0.737625
2	6	0	-4.519544	-0.722868	0.142604
3	6	0	-3.644966	0.274863	-0.308980
4	6	0	-4.273084	-2.144927	-0.100856
5	8	0	-2.597897	-0.030924	-1.169395
6	6	0	-3.079672	-2.755396	-0.279559
7	6	0	-1.760678	-2.130212	-0.285229
8	6	0	-1.557224	-0.782304	-0.640664
9	6	0	-0.621227	-2.880819	0.080188
10	6	0	0.645454	-2.321056	0.119994
11	6	0	-0.296858	-0.202416	-0.597101
12	6	0	0.816697	-0.965306	-0.216883
13	6	0	-3.859225	1.633373	-0.007091
14	6	0	-5.655131	-0.331404	0.880520
15	6	0	-5.880064	1.007330	1.171722
16	7	0	2.052613	-0.278147	-0.223024
17	7	0	3.047238	-0.973014	0.131416
18	6	0	4.455182	1.062040	-0.236795
19	6	0	5.719251	1.635227	-0.209844
20	6	0	4.284694	-0.288183	0.124638
21	6	0	5.397752	-1.049631	0.512493
22	6	0	6.805840	0.850624	0.179705
23	6	0	6.670120	-0.484579	0.542866
24	9	0	8.032122	1.411897	0.203682
25	8	0	-2.934980	2.505860	-0.494048
26	6	0	-3.102795	3.888504	-0.198251
27	1	0	-5.185022	3.033901	0.971263
28	1	0	-5.152590	-2.782606	-0.031554
29	1	0	-3.077925	-3.842236	-0.340552
30	1	0	-0.754133	-3.927658	0.342829

31	1	0	1.510458	-2.909323	0.403953
32	1	0	-0.172426	0.837630	-0.878120
33	1	0	-6.345831	-1.093935	1.229952
34	1	0	-6.756768	1.299466	1.742878
35	1	0	3.588070	1.640771	-0.534603
36	1	0	5.880296	2.673460	-0.483463
37	1	0	5.243748	-2.088710	0.787484
38	1	0	7.543482	-1.056412	0.839387
39	1	0	-2.255168	4.395211	-0.662442
40	1	0	-4.036678	4.279481	-0.621444
41	1	0	-3.089503	4.072020	0.883478

After PCM corrections, the SCF energy is -1168.99070232 a.u.

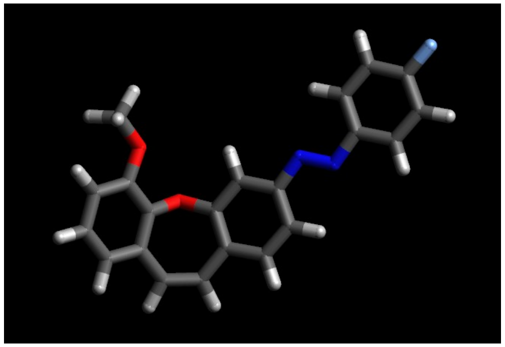

Visualization of calculated geometry of (**7b-*E***) :

- The calculated coordinates of (**7b-*Z***) (the part of calculated log file) -

Item	Value	Threshold	Converged?
Maximum Force	0.000164	0.000450	YES
RMS Force	0.000042	0.000300	YES
Maximum Displacement	0.001784	0.001800	YES
RMS Displacement	0.000331	0.001200	YES

Optimization completed.

Standard orientation:

Center Number	Atomic Number	Atomic Type	Coordinates (Angstroms) X	Y	Z
1	6	0	-4.505694	1.025889	1.529633
2	6	0	-3.469737	-1.372571	0.464444
3	6	0	-3.170184	-0.134904	-0.120444
4	6	0	-2.903160	-2.627992	-0.031442
5	8	0	-2.460946	-0.062193	-1.313729
6	6	0	-1.729003	-2.801822	-0.678396
7	6	0	-0.760505	-1.770971	-1.041653
8	6	0	-1.123737	-0.426984	-1.265060

9	6	0	0.600384	-2.100789	-1.193679
10	6	0	1.562442	-1.143906	-1.490401
11	6	0	-0.181479	0.539435	-1.588886
12	6	0	1.174794	0.193872	-1.669288
13	6	0	-3.669070	1.070855	0.408167
14	6	0	-4.324373	-1.390262	1.585341
15	6	0	-4.829506	-0.206593	2.106200
16	7	0	2.062381	1.207663	-2.152856
17	7	0	3.215132	1.411038	-1.705141
18	6	0	3.014611	0.780810	0.704177
19	6	0	3.637180	0.335732	1.866727
20	6	0	3.728275	0.818029	-0.505068
21	6	0	5.079417	0.442325	-0.528406
22	6	0	4.962882	-0.083641	1.801747
23	6	0	5.697649	-0.041666	0.621402
24	9	0	5.560216	-0.528137	2.927228
25	8	0	-3.300501	2.206214	-0.246183
26	6	0	-3.769385	3.450870	0.262222
27	1	0	-4.915103	1.937752	1.948660
28	1	0	-3.458510	-3.523392	0.241865
29	1	0	-1.416050	-3.824368	-0.881811
30	1	0	0.903132	-3.136842	-1.063294
31	1	0	2.601649	-1.432753	-1.603653
32	1	0	-0.506718	1.555159	-1.787322
33	1	0	-4.573090	-2.343309	2.044132
34	1	0	-5.484761	-0.230284	2.972420
35	1	0	1.984007	1.116297	0.740979
36	1	0	3.110400	0.310686	2.815497
37	1	0	5.632990	0.522416	-1.459321
38	1	0	6.734851	-0.361106	0.618090
39	1	0	-3.348444	4.215171	-0.393016
40	1	0	-4.864707	3.509897	0.233400
41	1	0	-3.423415	3.620529	1.289567

After PCM corrections, the SCF energy is -1168.96836679 a.u.

Visualization of calculated geometry of (**7b-*Z***) :

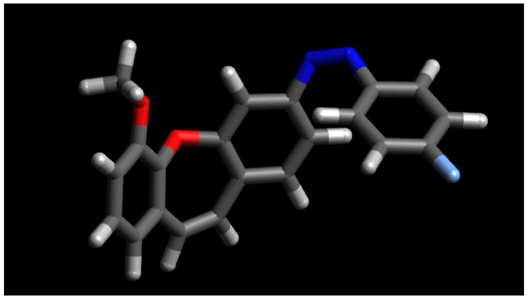

- The calculated coordinates of (**9e-*E***) (the part of calculated log file) -

Item	Value	Threshold	Converged?
Maximum Force	0.000371	0.000450	YES
RMS Force	0.000053	0.000300	YES
Maximum Displacement	0.001051	0.001800	YES
RMS Displacement	0.000174	0.001200	YES

Optimization completed.

Standard orientation:

Center Number	Atomic Number	Atomic Type	Coordinates (Angstroms) X	Y	Z
1	6	0	-0.108298	0.380518	1.316793
2	6	0	0.309249	0.390980	2.652281
3	6	0	-0.699770	-0.798625	0.816050
4	6	0	0.138233	-0.722586	3.466701
5	6	0	-0.844105	-1.930386	1.634684
6	6	0	-0.441114	-1.907264	2.983188
7	6	0	0.075152	1.579147	0.383225
8	6	0	1.366635	2.354898	0.650861
9	6	0	-1.137993	2.514644	0.344832
10	6	0	3.780824	3.811455	1.058354
11	6	0	3.721996	2.752429	0.133930
12	6	0	4.991879	4.585502	1.319930
13	8	0	4.767960	2.491486	-0.741211
14	6	0	6.275432	4.212404	1.129571
15	6	0	6.767905	2.948752	0.594120
16	6	0	6.010313	2.110819	-0.248808
17	6	0	8.081426	2.533042	0.908578
18	6	0	8.619136	1.349940	0.430462
19	6	0	6.539436	0.926102	-0.744250
20	6	0	7.843101	0.531787	-0.409717
21	6	0	2.539375	2.019694	-0.055703
22	6	0	2.603612	4.129489	1.756707
23	6	0	1.423126	3.426115	1.550516

24	7	0	8.268162	-0.687662	-0.986986
25	8	0	-1.244309	-3.105159	1.013173
26	6	0	-2.370793	-3.785452	1.459295
27	6	0	-2.461567	-4.306296	2.766255
28	6	0	-1.461839	-4.070397	3.801216
29	6	0	-0.590009	-3.043230	3.889628
30	6	0	-3.347798	-4.059890	0.510948
31	6	0	-4.451193	-4.863477	0.833783
32	6	0	-4.569669	-5.386095	2.133382
33	6	0	-3.583692	-5.110624	3.066198
34	7	0	-5.369393	-5.074742	-0.221714
35	6	0	-1.338883	3.305755	-0.804139
36	6	0	-2.403277	4.188819	-0.897410
37	6	0	-3.308950	4.305409	0.174431
38	6	0	-3.118560	3.524446	1.320420
39	6	0	-2.046316	2.636168	1.403097
40	7	0	-4.432098	5.168162	0.204842
41	7	0	-4.592431	5.841143	-0.854560
42	6	0	-6.588496	7.050343	0.159904
43	6	0	-7.618964	7.964079	-0.011939
44	6	0	-5.684331	6.725302	-0.877817
45	6	0	-5.895950	7.395094	-2.102647
46	6	0	-7.782009	8.591204	-1.248419
47	6	0	-6.918097	8.309266	-2.306805
48	9	0	-5.066152	7.124861	-3.123581
49	9	0	-6.461382	6.476568	1.365036
50	7	0	-6.299125	-5.886481	0.060745
51	7	0	9.440378	-1.030470	-0.651934
52	6	0	-8.103185	-7.213426	-0.761684
53	6	0	-9.127814	-7.543797	-1.634772
54	6	0	-7.265484	-6.091632	-0.937744
55	6	0	-7.547884	-5.285981	-2.064367
56	6	0	-9.359384	-6.717277	-2.734996
57	6	0	-8.574672	-5.582833	-2.949851
58	9	0	-6.834000	-4.172647	-2.286114
59	9	0	-7.880878	-8.007003	0.299017
60	6	0	9.333420	-3.060207	-2.179473
61	6	0	9.948398	-4.215348	-2.641181

62	6	0	9.923719	-2.229029	-1.198856
63	6	0	11.185064	-2.649031	-0.721638
64	6	0	11.192824	-4.583938	-2.126425
65	6	0	11.822012	-3.799947	-1.158960
66	9	0	11.792665	-1.894997	0.208675
67	9	0	8.143980	-2.735656	-2.704829
68	8	0	2.503253	1.034494	-1.015572
69	6	0	2.939694	-0.257975	-0.573268
70	8	0	-1.039334	-0.878894	-0.515044
71	6	0	-2.424858	-0.651826	-0.807498
72	1	0	0.791255	1.274931	3.056911
73	1	0	0.467531	-0.687037	4.501959
74	1	0	0.175115	1.161127	-0.621839
75	1	0	4.821975	5.544878	1.804933
76	1	0	7.048641	4.895533	1.475834
77	1	0	8.682576	3.174513	1.548497
78	1	0	9.628831	1.047419	0.682859
79	1	0	5.956891	0.313184	-1.422794
80	1	0	2.622757	4.953638	2.465002
81	1	0	0.524532	3.725554	2.081094
82	1	0	-1.484371	-4.776458	4.629034
83	1	0	0.032902	-2.997596	4.780905
84	1	0	-3.243663	-3.683933	-0.500602
85	1	0	-5.425310	-6.001520	2.386040
86	1	0	-3.666798	-5.520508	4.069841
87	1	0	-0.639902	3.220956	-1.633225
88	1	0	-2.555177	4.792161	-1.785694
89	1	0	-3.825995	3.624488	2.138489
90	1	0	-1.922489	2.030915	2.295529
91	1	0	-8.277263	8.171742	0.825276
92	1	0	-8.588824	9.304469	-1.386420
93	1	0	-7.021847	8.783117	-3.276962
94	1	0	-9.724346	-8.429485	-1.444300
95	1	0	-10.159117	-6.956207	-3.429264
96	1	0	-8.746759	-4.919852	-3.791410
97	1	0	9.444658	-4.808340	-3.397487
98	1	0	11.675107	-5.488530	-2.483921
99	1	0	12.788604	-4.063599	-0.743390

100	1	0	2.865824	-0.917033	-1.441082
101	1	0	3.977275	-0.227609	-0.223373
102	1	0	2.298545	-0.637760	0.229861
103	1	0	-2.533284	-0.789860	-1.885599
104	1	0	-3.064690	-1.367639	-0.281070
105	1	0	-2.722351	0.366945	-0.536811

After PCM corrections, the SCF energy is -3344.54697183 a.u.

Visualization of calculated geometry of (**9e-*E***) :

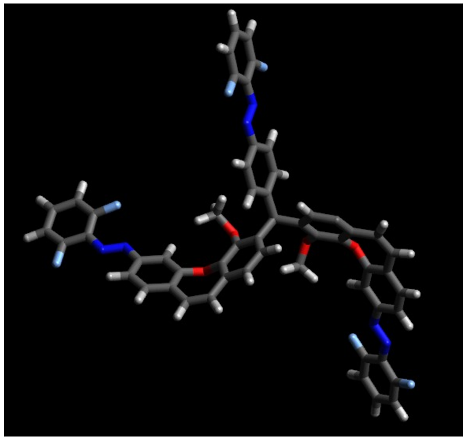

- The calculated coordinates of (**9e-*Z***) (the part of calculated log file) -

Item	Value	Threshold	Converged?
Maximum Force	0.000186	0.000450	YES
RMS Force	0.000037	0.000300	YES
Maximum Displacement	0.001424	0.001800	YES
RMS Displacement	0.000341	0.001200	YES

Optimization completed.

Standard orientation:

Center Number	Atomic Number	Atomic Type	Coordinates (Angstroms) X	Y	Z
1	6	0	0.441496	0.094527	-1.147770
2	6	0	0.238711	-0.202743	-2.501220
3	6	0	1.521516	-0.522659	-0.486454
4	6	0	1.075743	-1.083383	-3.180025
5	6	0	2.361426	-1.404785	-1.178222
6	6	0	2.166869	-1.694365	-2.539945
7	6	0	-0.440443	1.067623	-0.364565
8	6	0	-1.867712	1.166606	-0.907684
9	6	0	0.204920	2.446728	-0.204448
10	6	0	-4.565210	1.362398	-1.802454
11	6	0	-4.182142	0.379774	-0.870964

12	6	0	-5.928239	1.509070	-2.306840
13	8	0	-5.117962	-0.403239	-0.206545
14	6	0	-6.900961	0.575051	-2.360424
15	6	0	-6.840835	-0.808597	-1.901386
16	6	0	-5.950433	-1.265274	-0.905935
17	6	0	-7.717942	-1.756606	-2.464406
18	6	0	-7.707263	-3.089592	-2.079492
19	6	0	-5.965957	-2.584856	-0.469852
20	6	0	-6.858414	-3.502700	-1.045051
21	6	0	-2.847661	0.262542	-0.449340
22	6	0	-3.571226	2.241566	-2.263673
23	6	0	-2.257682	2.154901	-1.818097
24	7	0	-6.846856	-4.894996	-0.748385
25	8	0	3.336756	-2.067223	-0.443499
26	6	0	4.661088	-1.805663	-0.770711
27	6	0	5.193371	-2.244716	-2.000091
28	6	0	4.368771	-2.811842	-3.064003
29	6	0	3.058983	-2.573068	-3.296200
30	6	0	5.452125	-1.197560	0.192232
31	6	0	6.834163	-1.090337	-0.011106
32	6	0	7.398765	-1.528575	-1.219185
33	6	0	6.579526	-2.088134	-2.191818
34	7	0	7.569268	-0.374848	0.978802
35	6	0	-0.086989	3.203607	0.940827
36	6	0	0.433611	4.480523	1.118266
37	6	0	1.283278	5.023872	0.141542
38	6	0	1.632789	4.255104	-0.974880
39	6	0	1.075289	2.992258	-1.157436
40	7	0	1.962401	6.268967	0.294539
41	7	0	1.418582	7.314985	0.716784
42	6	0	-0.938999	7.260430	-0.125069
43	6	0	-2.274099	7.612656	0.014621
44	6	0	0.000591	7.449147	0.898037
45	6	0	-0.467792	8.060001	2.068611
46	6	0	-2.700364	8.183360	1.215127
47	6	0	-1.796691	8.408323	2.256314
48	9	0	0.420860	8.271327	3.059629
49	9	0	-0.517192	6.737300	-1.293254

50	7	0	8.700966	-0.715478	1.392069
51	7	0	-6.752196	-5.373350	0.405481
52	6	0	10.656004	-2.022734	0.758317
53	6	0	11.370902	-3.204861	0.641416
54	6	0	9.296397	-1.986604	1.096925
55	6	0	8.692417	-3.219424	1.383888
56	6	0	10.720972	-4.409940	0.918877
57	6	0	9.377595	-4.423090	1.298106
58	9	0	7.405135	-3.224939	1.782147
59	9	0	11.264748	-0.849739	0.497813
60	6	0	-5.975282	-4.830507	2.647688
61	6	0	-6.117227	-4.264092	3.905118
62	6	0	-6.872216	-4.587269	1.598114
63	6	0	-7.979981	-3.781518	1.899350
64	6	0	-7.227436	-3.452975	4.151981
65	6	0	-8.169571	-3.212025	3.150395
66	9	0	-8.902681	-3.577319	0.938723
67	9	0	-4.913453	-5.618301	2.390096
68	8	0	-2.514000	-0.681806	0.495576
69	6	0	-2.290012	-1.999786	-0.024065
70	8	0	1.772336	-0.215255	0.829609
71	6	0	1.429344	-1.234749	1.780560
72	1	0	-0.594981	0.251069	-3.027511
73	1	0	0.896150	-1.295733	-4.230714
74	1	0	-0.529402	0.655818	0.643586
75	1	0	-6.140929	2.471783	-2.767550
76	1	0	-7.827656	0.849550	-2.860513
77	1	0	-8.408244	-1.428209	-3.237035
78	1	0	-8.367197	-3.814164	-2.546123
79	1	0	-5.284446	-2.878438	0.318786
80	1	0	-3.848126	3.013550	-2.976858
81	1	0	-1.527413	2.877040	-2.168624
82	1	0	4.904324	-3.418145	-3.792085
83	1	0	2.625105	-3.005478	-4.195911
84	1	0	5.001586	-0.843802	1.113142
85	1	0	8.458781	-1.401458	-1.409085
86	1	0	7.015165	-2.408572	-3.134748
87	1	0	-0.731254	2.783159	1.709268

88	1	0	0.213067	5.034813	2.024379
89	1	0	2.327730	4.666083	-1.701425
90	1	0	1.343951	2.416019	-2.037477
91	1	0	-2.954568	7.444237	-0.813495
92	1	0	-3.743359	8.456958	1.339622
93	1	0	-2.103664	8.850736	3.198160
94	1	0	12.414078	-3.169583	0.345586
95	1	0	11.266437	-5.345189	0.841542
96	1	0	8.857234	-5.345309	1.534816
97	1	0	-5.372040	-4.465706	4.667382
98	1	0	-7.360516	-3.007802	5.132937
99	1	0	-9.047681	-2.598851	3.324213
100	1	0	-2.095157	-2.638949	0.840219
101	1	0	-3.168251	-2.371244	-0.562015
102	1	0	-1.424424	-2.015125	-0.695375
103	1	0	1.701664	-0.835537	2.759931
104	1	0	0.351576	-1.439035	1.759507
105	1	0	1.986219	-2.156432	1.589930

After PCM corrections, the SCF energy is -3344.49871082 a.u.

Visualization of calculated geometry of (**9e-*Z***) :

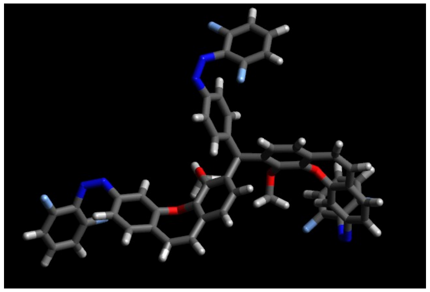

- The calculated coordinates of (**9f-*E***) (the part of calculated log file) -

Item	Value	Threshold	Converged?
Maximum Force	0.000344	0.000450	YES
RMS Force	0.000053	0.000300	YES
Maximum Displacement	0.000831	0.001800	YES
RMS Displacement	0.000194	0.001200	YES

Optimization completed.

Standard orientation:

Center Number	Atomic Number	Atomic Type	Coordinates (Angstroms) X	Y	Z
1	6	0	0.227229	-0.112022	1.066313
2	6	0	0.738580	-0.196259	2.371043
3	6	0	-0.439404	-1.239772	0.530167
4	6	0	0.598900	-1.354385	3.125944
5	6	0	-0.574898	-2.399183	1.304614
6	6	0	-0.079046	-2.475384	2.619836
7	6	0	0.310686	1.161379	0.223520
8	6	0	1.489731	2.064503	0.592775
9	6	0	-1.012882	1.930320	0.216604
10	6	0	3.704340	3.766739	1.172435
11	6	0	3.814007	2.735665	0.223823
12	6	0	4.800018	4.676685	1.504213
13	8	0	4.913630	2.634507	-0.617349
14	6	0	6.128390	4.470754	1.355475
15	6	0	6.792941	3.293453	0.804754
16	6	0	6.175894	2.395425	-0.087518
17	6	0	8.137487	3.029397	1.154024
18	6	0	8.833987	1.937085	0.659234
19	6	0	6.864100	1.304579	-0.599882
20	6	0	8.193341	1.059757	-0.231154
21	6	0	2.733395	1.882287	-0.045696
22	6	0	2.465338	3.922098	1.818417
23	6	0	1.383217	3.096534	1.530252
24	7	0	8.786972	-0.071348	-0.836509
25	8	0	-1.145663	-3.509638	0.696589
26	6	0	-2.355878	-3.961357	1.213592
27	6	0	-2.408992	-4.547981	2.494191
28	6	0	-1.274707	-4.548970	3.410373
29	6	0	-0.267274	-3.652876	3.463640
30	6	0	-3.475499	-3.913525	0.391354
31	6	0	-4.685795	-4.486144	0.816070
32	6	0	-4.750998	-5.110653	2.076530
33	6	0	-3.629513	-5.137703	2.885868
34	7	0	-5.765496	-4.365208	-0.090869
35	6	0	-1.348797	2.703465	-0.911782

36	6	0	-2.538693	3.414593	-0.972987
37	6	0	-3.427541	3.382474	0.118393
38	6	0	-3.091545	2.633154	1.251177
39	6	0	-1.903509	1.906255	1.294946
40	7	0	-4.675559	4.048225	0.175779
41	7	0	-4.970795	4.711295	-0.857239
42	6	0	-7.106577	5.326782	0.291200
43	6	0	-8.311581	6.015042	0.239555
44	6	0	-6.220192	5.373176	-0.801062
45	6	0	-6.560469	6.117200	-1.940774
46	6	0	-8.620212	6.746688	-0.908084
47	6	0	-7.766288	6.811296	-2.003295
48	7	0	-6.864108	-4.839131	0.317745
49	7	0	9.978385	-0.297657	-0.481360
50	6	0	-9.169974	-5.215143	-0.146617
51	6	0	-10.303454	-5.136999	-0.951328
52	6	0	-7.940128	-4.707159	-0.590565
53	6	0	-7.849073	-4.109388	-1.861705
54	6	0	-10.181798	-4.543875	-2.202701
55	6	0	-8.972873	-4.027222	-2.672507
56	6	0	9.942374	-2.248965	-2.049620
57	6	0	10.625620	-3.327096	-2.595620
58	6	0	10.573670	-1.419036	-1.101357
59	6	0	11.895716	-1.688343	-0.713623
60	6	0	11.938551	-3.567421	-2.188263
61	6	0	12.589283	-2.767417	-1.255204
62	8	0	2.869718	0.910323	-1.007621
63	6	0	3.513201	-0.296832	-0.581018
64	8	0	-1.006099	-1.182520	-0.727110
65	6	0	-0.345677	-1.923760	-1.766027
66	9	0	-9.793157	7.411875	-0.954714
67	9	0	12.597659	-4.616343	-2.720696
68	9	0	-11.271390	-4.459864	-2.993170
69	1	0	1.260639	0.657088	2.795826
70	1	0	0.999246	-1.390460	4.135815
71	1	0	0.482741	0.845057	-0.807514
72	1	0	4.491738	5.593049	2.003857
73	1	0	6.798828	5.231985	1.749525

74	1	0	8.635766	3.720454	1.829555
75	1	0	9.863218	1.748781	0.941639
76	1	0	6.385825	0.650544	-1.320446
77	1	0	2.355985	4.719513	2.548880
78	1	0	0.431729	3.275842	2.019300
79	1	0	-1.307243	-5.307916	4.189956
80	1	0	0.442030	-3.752446	4.283233
81	1	0	-3.412382	-3.441539	-0.582391
82	1	0	-5.682290	-5.562044	2.396919
83	1	0	-3.684683	-5.612920	3.862340
84	1	0	-0.666863	2.727915	-1.759118
85	1	0	-2.801682	3.994605	-1.851002
86	1	0	-3.788112	2.614627	2.084506
87	1	0	-1.674907	1.311606	2.174140
88	1	0	-6.833675	4.747725	1.166452
89	1	0	-9.013761	5.997621	1.067455
90	1	0	-5.862592	6.138115	-2.772365
91	1	0	-8.047272	7.393097	-2.875264
92	1	0	-9.218966	-5.666137	0.840024
93	1	0	-11.263458	-5.525562	-0.626997
94	1	0	-6.893941	-3.717238	-2.194020
95	1	0	-8.931766	-3.574525	-3.658413
96	1	0	8.921452	-2.033343	-2.344417
97	1	0	10.164884	-3.982678	-3.328267
98	1	0	12.363677	-1.035742	0.017034
99	1	0	13.611445	-2.992742	-0.968885
100	1	0	3.593098	-0.924239	-1.471518
101	1	0	4.514753	-0.112940	-0.179166
102	1	0	2.914666	-0.815063	0.177471
103	1	0	-0.926654	-1.776694	-2.680310
104	1	0	0.672987	-1.550427	-1.925803
105	1	0	-0.311347	-2.990070	-1.521343

```
After PCM corrections, the SCF energy is -3046.88365680     a.u.
```

Visualization of calculated geometry of (**9f-*E***) :

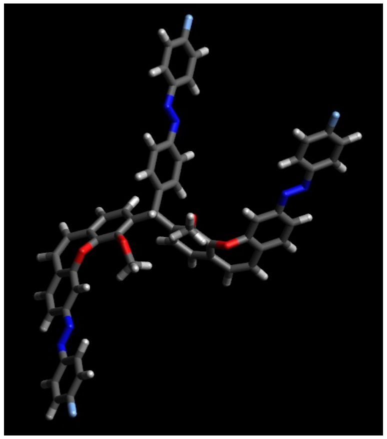

- The calculated coordinates of (**9f-*Z***) (the part of calculated log file) –

Item	Value	Threshold	Converged?
Maximum Force	0.000019	0.000450	YES
RMS Force	0.000002	0.000300	YES
Maximum Displacement	0.000072	0.001800	YES
RMS Displacement	0.000011	0.001200	YES

Optimization completed.

Standard orientation:

Center Number	Atomic Number	Atomic Type	Coordinates (Angstroms) X	Y	Z
1	6	0	-0.826374	0.803398	0.376840
2	6	0	-0.664894	0.968577	1.758036
3	6	0	-1.808919	-0.105724	-0.070171
4	6	0	-1.451526	0.266483	2.665674
5	6	0	-2.581065	-0.834647	0.851202
6	6	0	-2.427834	-0.650742	2.238166
7	6	0	0.036858	1.544332	-0.647029
8	6	0	1.491506	1.706187	-0.196703
9	6	0	-0.560176	2.882034	-1.101348
10	6	0	4.230283	2.005754	0.544409
11	6	0	3.797879	0.891439	-0.198418
12	6	0	5.618492	2.214019	0.954957
13	8	0	4.704429	0.001725	-0.764177
14	6	0	6.585577	1.283838	1.120399
15	6	0	6.486822	-0.157343	0.904945
16	6	0	5.543165	-0.756591	0.045111
17	6	0	7.382565	-1.018378	1.572731
18	6	0	7.368148	-2.395903	1.387203
19	6	0	5.494484	-2.134714	-0.132319
20	6	0	6.429634	-2.958333	0.508445
21	6	0	2.445694	0.728422	-0.547793
22	6	0	3.263369	2.965169	0.895145
23	6	0	1.930432	2.824299	0.523985
24	7	0	6.246148	-4.372956	0.367616
25	8	0	-3.369907	-1.858886	0.339285
26	6	0	-4.739958	-1.874483	0.577215
27	6	0	-5.266351	-1.958372	1.882872

28	6	0	-4.446457	-1.913823	3.090794
29	6	0	-3.221234	-1.362305	3.239916
30	6	0	-5.562177	-1.933134	-0.542360
31	6	0	-6.941325	-2.129947	-0.391149
32	6	0	-7.499877	-2.193727	0.894741
33	6	0	-6.664057	-2.107135	2.001917
34	7	0	-7.734373	-2.065480	-1.583295
35	6	0	-0.214073	3.375900	-2.370850
36	6	0	-0.727917	4.581696	-2.839463
37	6	0	-1.558445	5.356205	-2.015911
38	6	0	-1.929461	4.869452	-0.754967
39	6	0	-1.438641	3.639273	-0.313818
40	7	0	-2.147850	6.524470	-2.605186
41	7	0	-2.256589	7.627044	-2.022308
42	6	0	-2.389900	8.682020	0.143764
43	6	0	-1.824456	9.122481	1.340362
44	6	0	-1.627390	7.939630	-0.769542
45	6	0	-0.265420	7.703622	-0.520150
46	6	0	-0.478961	8.857739	1.566321
47	6	0	0.318893	8.174057	0.655234
48	7	0	-8.676188	-2.847626	-1.845612
49	7	0	7.175826	-5.185469	0.160354
50	6	0	-10.310285	-4.347645	-0.906761
51	6	0	-10.682449	-5.540517	-0.287743
52	6	0	-8.955177	-4.046299	-1.108572
53	6	0	-7.967090	-4.983001	-0.761408
54	6	0	-9.680472	-6.438586	0.060600
55	6	0	-8.331979	-6.195753	-0.179003
56	6	0	8.810417	-3.854884	-1.178800
57	6	0	10.132217	-3.643976	-1.567990
58	6	0	8.514717	-4.803315	-0.185199
59	6	0	9.543947	-5.566433	0.385847
60	6	0	11.134417	-4.376421	-0.940433
61	6	0	10.872734	-5.334285	0.032152
62	8	0	2.077289	-0.339061	-1.335589
63	6	0	1.776153	-1.557053	-0.627412
64	8	0	-1.935249	-0.373355	-1.414666
65	6	0	-2.973216	0.352925	-2.101301

66	9	0	0.089634	9.305287	2.722054
67	9	0	12.427725	-4.159444	-1.311361
68	9	0	-10.035277	-7.618298	0.643397
69	1	0	0.096203	1.644485	2.132385
70	1	0	-1.306079	0.416988	3.731408
71	1	0	0.063464	0.905123	-1.532439
72	1	0	5.862112	3.236006	1.235898
73	1	0	7.536193	1.622729	1.525639
74	1	0	8.111837	-0.582827	2.249754
75	1	0	8.071315	-3.024693	1.921106
76	1	0	4.760533	-2.564788	-0.804592
77	1	0	3.575409	3.840634	1.457104
78	1	0	1.223697	3.605602	0.783745
79	1	0	-4.920522	-2.318045	3.982205
80	1	0	-2.795719	-1.365625	4.240680
81	1	0	-5.126635	-1.872478	-1.533393
82	1	0	-8.569896	-2.304230	1.027368
83	1	0	-7.096036	-2.161918	2.997018
84	1	0	0.449079	2.797637	-3.008760
85	1	0	-0.484171	4.936793	-3.836189
86	1	0	-2.611955	5.432883	-0.128147
87	1	0	-1.754626	3.269379	0.656061
88	1	0	-3.428902	8.898984	-0.082475
89	1	0	-2.403746	9.675578	2.071311
90	1	0	0.342024	7.172187	-1.243557
91	1	0	1.371370	8.015893	0.863500
92	1	0	-11.066179	-3.636463	-1.223738
93	1	0	-11.722767	-5.780792	-0.098734
94	1	0	-6.921325	-4.779917	-0.960423
95	1	0	-7.588840	-6.940311	0.084408
96	1	0	8.016437	-3.297500	-1.661850
97	1	0	10.383858	-2.927922	-2.342538
98	1	0	9.297509	-6.329675	1.116837
99	1	0	11.685262	-5.894878	0.480742
100	1	0	1.577168	-2.310656	-1.390560
101	1	0	2.623057	-1.870945	-0.010670
102	1	0	0.892221	-1.429001	0.005043
103	1	0	-2.994458	-0.038809	-3.119196

```
    104          1          0       -3.944204    0.193042   -1.624109
    105          1          0       -2.747834    1.423472   -2.121668
 ---------------------------------------------------------------------
After PCM corrections, the SCF energy is -3046.84664505      a.u.
```

Visualization of calculated geometry of (**9f-*Z***) :

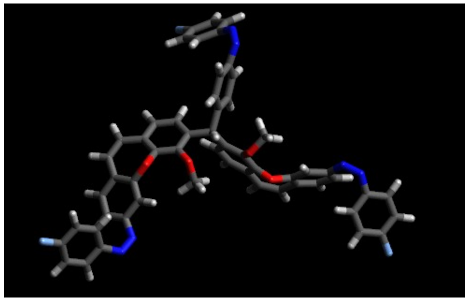

Molecular docking - The molecular docking of the compounds of the (**2a)** and (**2f**) and (**7b**), (**9e**), (**9f**) ***E*** and ***Z*** isomers, into the 3D X-ray structure of tubulin (PDB code: 1SA0)[3] was conducted using the Auto-Dock Vina software (the Broyden–Fletcher–Goldfarb–Shanno (BFGS) method).[4] The microtubule structure (PDB-ID:1SA0) was prepared by the PyMOL graphical user interface and Python scripts provided by AutodockTools. In the tubulin structure: chains C, D, and E were removed as well as small molecules (magnesium ion, GDT, GTP, and DAMA-colchicine). All hydrogens were added. Next Python script *prepare_receptor4.py* was used for .pdbqt file generation. The configurations of the protein/hybrid dimethoxydibenzo[*b*,*f*]oxepine combined with fluoroazobenzenes complex were created using UCSF Chimera software.[5] The graphical user interface, ADT, was employed to set up the enzyme, and all the hydrogens were added. For macromolecules, the generated pdbqt files were saved. The 3D structures of ligand molecules were built, and optimized (a B3LYP functional and 6–31* basis set level) for the (**2a)** and (**2f**) and (**7b**), (**9e**), (**9f**) ***E***, ***Z*** isomers and saved in Mol2 format. The graphical user interface, ADT, was also employed to set up the ligand, and the pdbqt file was saved. The Auto-Dock Vina software was employed for all docking calculations. The AutoDockTools program was used to generate the docking input files. During docking, a grid box of size 21 x 21 x 21 pointed in the x, y, and z directions was built and the maps were center located (39.82, 53.24, -8.21) in the catalytic site of the protein. A grid spacing of 0.375 Å (approximately one–fourth of the length of a carbon-carbon covalent bond) was used for the calculation of the energetic map. The files .pdb for investigated molecules [(**2a**) and (**2f**) and (**7b**), (**9e**), (**9f**) ***E*** and ***Z*** isomers] obtained from DFT calculations were converted it to the pdbqt format with a script from AutoDockTool.

a) **b)** **c)**

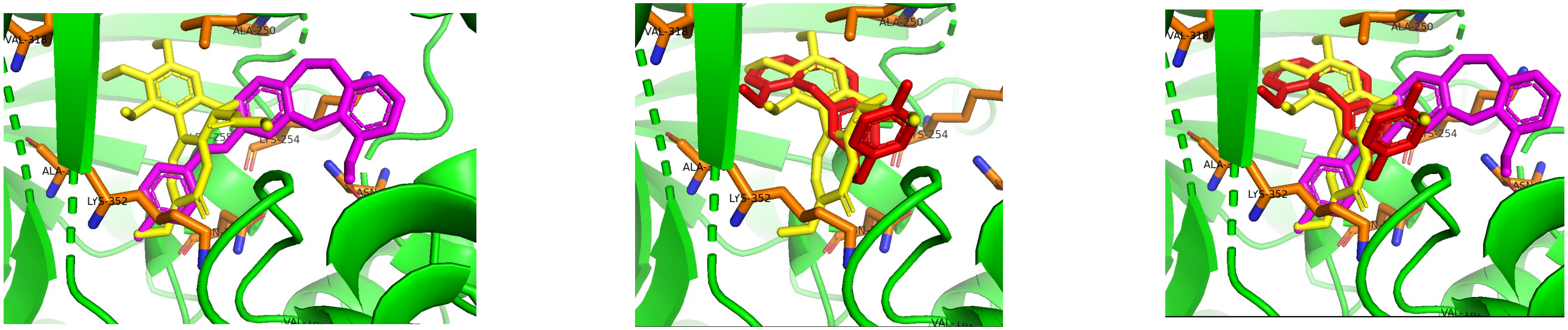

The results of docked structure of colchicine superimposed on the docked structure: a) of **7b*E*** and b) of **7b**Z and c) together **7bE**/**7bZ** (the structure of colchicine in yellow; the structure of **7b*E*** in purple; the structure of **7b*Z*** in red).

<table>
<tr><th>Compound</th><th>Affinity (kcal /mol)</th><th>Connections (date from RCSB Protein Data Bank—RCSB PDB)</th></tr>
<tr><td>2a 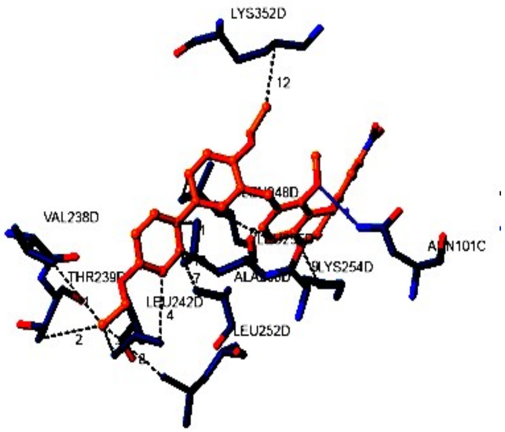
</td><td>-10.0</td><td>
Hydrophobic Interactions
<table>
<tr><th>Index</th><th>Residue</th><th>AA</th><th>Distance</th><th>Ligand Atom</th><th>Protein Atom</th></tr>
<tr><td>1</td><td>238D</td><td>VAL</td><td>3.99</td><td>30</td><td>5042</td></tr>
<tr><td>2</td><td>239D</td><td>THR</td><td>3.79</td><td>30</td><td>5049</td></tr>
<tr><td>3</td><td>242D</td><td>LEU</td><td>3.70</td><td>30</td><td>5070</td></tr>
<tr><td>4</td><td>242D</td><td>LEU</td><td>3.47</td><td>25</td><td>5071</td></tr>
<tr><td>5</td><td>248D</td><td>LEU</td><td>3.63</td><td>20</td><td>5120</td></tr>
<tr><td>6</td><td>248D</td><td>LEU</td><td>3.52</td><td>14</td><td>5118</td></tr>
<tr><td>7</td><td>250D</td><td>ALA</td><td>3.18</td><td>24</td><td>5134</td></tr>
<tr><td>8</td><td>252D</td><td>LEU</td><td>3.96</td><td>30</td><td>5149</td></tr>
<tr><td>9</td><td>254D</td><td>LYS</td><td>3.57</td><td>3</td><td>5168</td></tr>
<tr><td>10</td><td>255D</td><td>LEU</td><td>3.62</td><td>24</td><td>5176</td></tr>
<tr><td>11</td><td>255D</td><td>LEU</td><td>3.26</td><td>23</td><td>5178</td></tr>
<tr><td>12</td><td>352D</td><td>LYS</td><td>3.63</td><td>32</td><td>5877</td></tr>
</table>
Hydrogen Bonds
<table>
<tr><th>Index</th><th>Residue</th><th>AA</th><th>Distance H-A</th><th>Distance D-A</th><th>Donor Angle</th><th>Protein donor?</th><th>Side chain</th><th>Donor Atom</th><th>Acceptor Atom</th></tr>
<tr><td>1</td><td>101C</td><td>ASN</td><td>2.30</td><td>3.05</td><td>132.49</td><td>✔</td><td>✔</td><td>713 [Nam]</td><td>37 [O3]</td></tr>
</table>
</td></tr>
<tr><td>2b 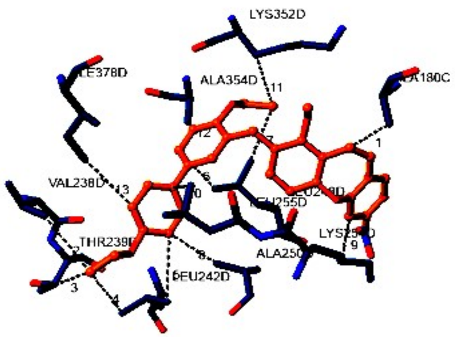
</td><td>-10.4</td><td>
Hydrophobic Interactions
<table>
<tr><th>Index</th><th>Residue</th><th>AA</th><th>Distance</th><th>Ligand Atom</th><th>Protein Atom</th></tr>
<tr><td>1</td><td>180C</td><td>ALA</td><td>3.16</td><td>6464</td><td>1284</td></tr>
<tr><td>2</td><td>238D</td><td>VAL</td><td>3.72</td><td>6491</td><td>5003</td></tr>
<tr><td>3</td><td>239D</td><td>THR</td><td>3.76</td><td>6491</td><td>5010</td></tr>
<tr><td>4</td><td>242D</td><td>LEU</td><td>3.98</td><td>6491</td><td>5031</td></tr>
<tr><td>5</td><td>242D</td><td>LEU</td><td>3.70</td><td>6486</td><td>5032</td></tr>
<tr><td>6</td><td>248D</td><td>LEU</td><td>3.84</td><td>6479</td><td>5081</td></tr>
<tr><td>7</td><td>248D</td><td>LEU</td><td>3.94</td><td>6483</td><td>5082</td></tr>
<tr><td>8</td><td>250D</td><td>ALA</td><td>3.49</td><td>6486</td><td>5095</td></tr>
<tr><td>9</td><td>254D</td><td>LYS</td><td>3.86</td><td>6470</td><td>5129</td></tr>
<tr><td>10</td><td>255D</td><td>LEU</td><td>3.40</td><td>6484</td><td>5139</td></tr>
<tr><td>11</td><td>352D</td><td>LYS</td><td>3.86</td><td>6483</td><td>5836</td></tr>
<tr><td>12</td><td>354D</td><td>ALA</td><td>3.26</td><td>6478</td><td>5852</td></tr>
<tr><td>13</td><td>378D</td><td>ILE</td><td>3.78</td><td>6488</td><td>5962</td></tr>
</table>
</td></tr>
</table>

7b*E*

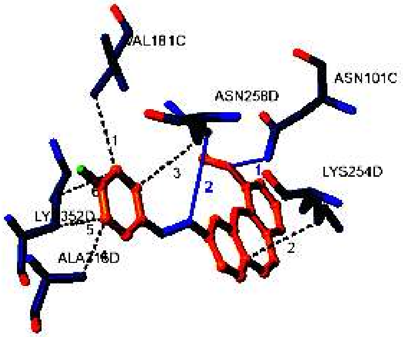

-9.6

Hydrophobic Interactions ····

Index	Residue	AA	Distance	Ligand Atom	Protein Atom
1	181C	VAL	3.40	6480	1291
2	254D	LYS	3.76	6471	5129
3	258D	ASN	3.77	6481	5156
4	316D	ALA	3.44	6478	5555
5	352D	LYS	3.70	6478	5836
6	352D	LYS	3.65	6480	5837

Hydrogen Bonds —

Index	Residue	AA	Distance H-A	Distance D-A	Donor Angle	Protein donor?	Side chain	Donor Atom	Acceptor Atom
1	101C	ASN	2.52	3.33	140.38	✔	✔	674 [Nam]	6484 [O3]
2	258D	ASN	3.23	4.01	137.55	×	✔	6460 [Npl]	5159 [O2]

7b*Z*

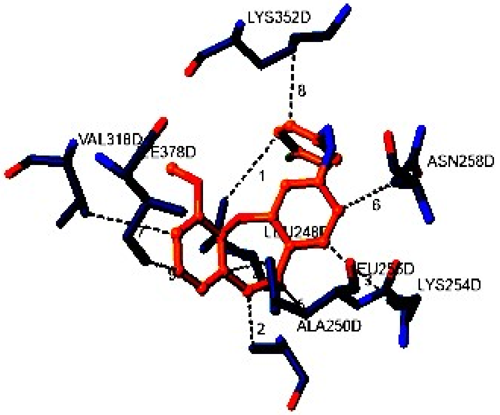

-9.2

Hydrophobic Interactions ····

Index	Residue	AA	Distance	Ligand Atom	Protein Atom
1	248D	LEU	3.84	6478	5082
2	250D	ALA	3.20	6475	5095
3	254D	LYS	3.73	6462	5127
4	255D	LEU	3.33	6474	5139
5	255D	LEU	3.77	6473	5136
6	258D	ASN	3.78	6472	5156
7	318D	VAL	3.95	6467	5566
8	352D	LYS	3.69	6477	5838
9	378D	ILE	3.61	6469	5962

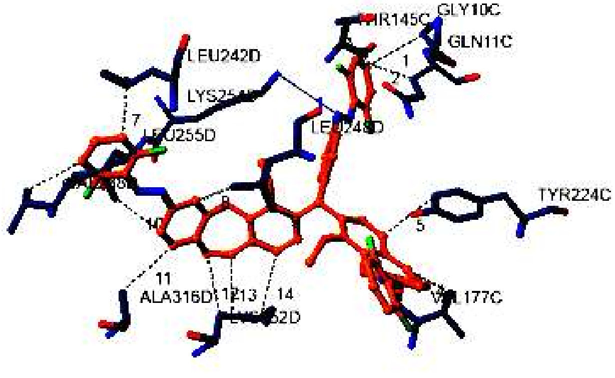

9e*E*

-9.1

⌄ Hydrophobic Interactions

Index	Residue	AA	Distance	Ligand Atom	Protein Atom
1	10C	GLY	3.82	6497	67
2	11C	GLN	3.91	6496	74
3	145C	THR	3.46	6497	1016
4	177C	VAL	3.41	6472	1265
5	224C	TYR	3.52	6469	1635
6	238D	VAL	3.61	6519	5003
7	242D	LEU	2.69	6517	5030
8	248D	LEU	3.97	6512	5081
9	248D	LEU	3.89	6500	5082
10	255D	LEU	3.13	6510	5139
11	316D	ALA	3.79	6508	5555
12	352D	LYS	3.44	6506	5836
13	352D	LYS	3.35	6504	5837
14	352D	LYS	3.73	6505	5838

⌄ Hydrogen Bonds

Index	Residue	AA	Distance H-A	Distance D-A	Donor Angle	Protein donor?	Side chain	Donor Atom	Acceptor Atom
1	254D	LYS	3.45	3.92	110.05	✔	✔	5131 [N3+]	6462 [Npl]

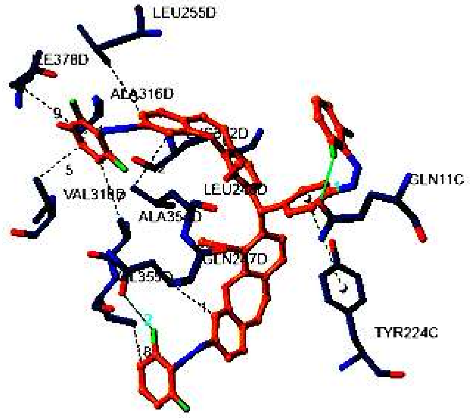

9e*Z*

-9.7

⌄ Hydrophobic Interactions

Index	Residue	AA	Distance	Ligand Atom	Protein Atom
1	247D	GLN	3.54	6505	5070
2	248D	LEU	3.70	6491	5081
3	255D	LEU	3.57	6489	5139
4	316D	ALA	3.47	6496	5555
5	318D	VAL	3.27	6496	5566
6	352D	LYS	3.93	6483	5837
7	354D	ALA	3.72	6495	5852
8	355D	VAL	3.73	6517	5859
9	378D	ILE	3.65	6497	5962

⌄ π-Stacking

Index	Residue	AA	Distance	Angle	Offset	Stacking Type	Ligand Atoms
1	224C	TYR	5.34	68.04	1.34	T	6467, 6468, 6469, 6470, 6471, 6472

⌄ Halogen Bonds

Index	Residue	AA	Distance	Donor Angle	Acceptor Angle	Donor Atom	Acceptor Atom
1	11C	GLN	2.74	145.19	107.53	6525 [F]	78 [O2]
2	247D	GLN	3.03	143.07	129.69	6529 [F]	5074 [O2]

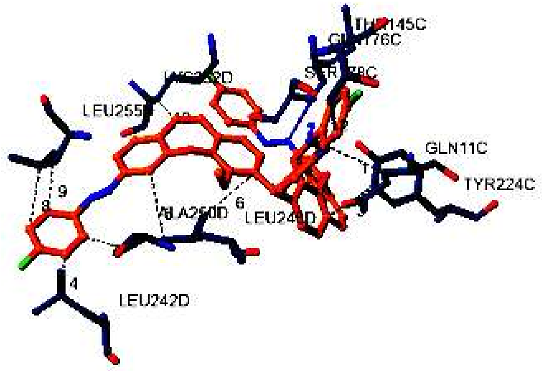

9f*E*

-6.0

⌄ Hydrophobic Interactions ····

Index	Residue	AA	Distance	Ligand Atom	Protein Atom
1	11C	GLN	3.53	6495	74
2	145C	THR	3.64	6496	1016
3	224C	TYR	3.78	6470	1635
4	242D	LEU	2.99	6517	5030
5	248D	LEU	3.81	6512	5081
6	248D	LEU	3.66	6500	5082
7	250D	ALA	3.39	6516	5095
8	255D	LEU	3.62	6519	5137
9	255D	LEU	3.20	6520	5139
10	352D	LYS	2.92	6506	5836

⌄ Hydrogen Bonds —

Index	Residue	AA	Distance H-A	Distance D-A	Donor Angle	Protein donor?	Side chain	Donor Atom	Acceptor Atom
1	176C	GLN	1.84	2.63	134.24	×	×	6460 [Npl]	1254 [O2]
2	178C	SER	2.93	3.85	155.36	✓	×	1267 [Nam]	6460 [Npl]

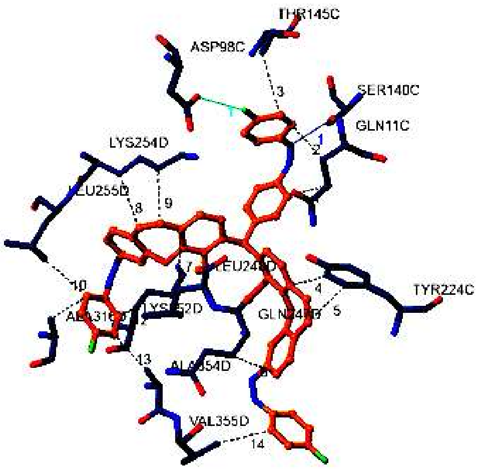

9f*Z*

-11.0

⌄ Hydrophobic Interactions ····

Index	Residue	AA	Distance	Ligand Atom	Protein Atom
1	11C	GLN	3.97	6513	75
2	11C	GLN	3.56	6520	74
3	145C	THR	3.88	6519	1016
4	224C	TYR	3.15	6491	1635
5	224C	TYR	3.95	6494	1633
6	247D	GLN	3.60	6498	5070
7	248D	LEU	3.85	6470	5079
8	254D	LYS	3.42	6477	5127
9	254D	LYS	3.22	6472	5129
10	255D	LEU	3.67	6487	5139
11	316D	ALA	3.35	6487	5555
12	352D	LYS	3.38	6483	5836
13	354D	ALA	3.50	6484	5852
14	355D	VAL	3.47	6507	5859

⌄ Hydrogen Bonds —

Index	Residue	AA	Distance H-A	Distance D-A	Donor Angle	Protein donor?	Side chain	Donor Atom	Acceptor Atom
1	140C	SER	2.17	3.09	156.67	✓	✓	987 [O3]	6465 [N2]

⌄ Halogen Bonds —

Index	Residue	AA	Distance	Donor Angle	Acceptor Angle	Donor Atom	Acceptor Atom
1	98C	ASP	3.27	153.46	129.74	6527 [F]	656 [O3]

2. Experimental section

General information:

Nuclear magnetic resonance (NMR) spectroscopy: Nmr All the spectra were recorded using a Varian VNMRS spectrometer operating at 11.7 T and Varian Mercury VX 9.4 T magnetic field. Measurements were performed for ca. 1.0 M solutions of all the compounds in DMSO-d_6 or $CDCl_3$. The residual signals of DMSO-d6 (2.54 ppm) and $CDCl_3$ (7.26 ppm) in ^{1}H NMR and of the DMSO-d_6 signal (40.4 ppm) and of $CDCl_3$ (77.0 ppm) in ^{13}C NMR spectra were used as the chemical shift references. Spin multiplicities are described as s (singlet), d(doublet), t (triplet), q (quartet), m (multiplet), dd (double doublet). Coupling constants are reported in Hertz. All the proton spectra were recorded using the standard spectrometer software and parameters set: acquisition time 3s, pulse angle 30°. The standard measurement parameter set for ^{13}C NMR spectra was: pulse width 7 μs (the 90o pulse width was 12.5 μs), acquisition time 1 s, spectral width 200 ppm, 1000 scans of 32 K data point were accumulated and after zero-filling to 64 K; and the FID signals were subjected to Fourier transformation after applying a 1 Hz line broadening. The ^{1}H-^{13}Cgs-HSQC and ^{1}H-^{13}Cgs-HMBC spectra were also recorded using the standard Varian software. The measurement parameters guaranteeing the increase in magnetization and the possibility of quantitative integrating the signals were for ^{19}F NMR: pulse width 1 μs (the 90 pulse width was 9 μs), acquisition time 2.3 s, spectral width 241 ppm, 32 scans of 524 K data point were accumulated.

Mass spectrometry (MS): Mass spectra were recorded on spectrometer QTOF Premier firmy Waters and spectrometer LTQ Orbitrap Velos.

UV-VIS spectrometer: EnSpire® multimode plate reader (PerkinElmer, Turku, Finland) with the software EnSpire Workstation version 4.10.3005.1440 (PerkinElmer) in absorbance mode was used for UV-VIS spectroscopic measurements. All experiments were done in at least triplicate.

Melting point: The apparatus used for these measurements was Stuart SMP10 melting point apparatus. We measured the melting point with an accuracy of 1-2 degrees Celsius.

All commercially available compounds were purchased from Merck, Sigma-Aldrich, and used without further purification, Solvents were dried according to standard procedures. The dibenzo [*b, f*] oxepines (**1a-b**) were obtained using the procedure described by us in Tetrahedron (H. Krawczyk, M. Wrzesiński, D. Mielecki, P. Szczeciński, E. Grzesiuk), Synthesis of derivatives of methoxydibenzo[*b,f*]oxepine in the presence of sodium azide. Tetrahedron 2016, 72, 3877-3884.

General procedure of synthesis of compounds (2b-j) (Scheme 1 in the text):

To a solution of 6-methoxy-3-nitrodibenzo[*b,f*]oxepine (1 mmol) or 1-methoxy-7-nitrodibenzo[*b,f*]oxepine (1 mmol) and 4,4'-diethoxybiphenyl (1 mmol) in dichloromethane (20 mL) a proper aldehyde (1 mmol) was added. Afterward, the catalyst – $BF_3{\cdot}Et_2O$ (1 mL) was added carefully to a reaction mixture under an argon atmosphere. The resulting solution turned dark green to brown and was stirred for 3 h-4 days at room temperature. Afterward, the reaction mixture was quenched by the addition of methanol (10 mL). The mixture was transferred to a separation funnel filled with 15 mL of water. The water layer was washed twice with 20 mL of dichloromethane. Organic layers were dried over anhydrous magnesium sulfate and concentrated to dryness in vacuo. The residue was subjected to column chromatography on silica gel using dichloromethane/hexane 1:2 as a mobile phase. The obtained crude product was then again subjected to column chromatography on silica gel using hexane/diethyl ether/dichloromethane 8:2:1 as a mobile phase.

Characterisation Date of (2b-2j)

Product	Conditions	Yield [%]	Melting point [ºC] / color
**(2a)**	Compound described by us in RSC Adv., **2018**, 8, 30678–30682		
**(2b)**	$BF_3 \cdot Et_2O$, CH_2Cl_2, rt, Ar, 3 days	15	159.5-160.5 / light yellow
**(2c)**	$BF_3 \cdot Et_2O$, CH_2Cl_2, rt, Ar, 3 days	20	94-95 / light yellow
**(2d)**	$BF_3 \cdot Et_2O$, CH_2Cl_2, rt, Ar, 4 days	9	183-185 / light yellow
**(2e)**	$BF_3 \cdot Et_2O$, CH_2Cl_2, rt, Ar, 4 days	8	71.5-72.5 / light yellow
**(2f)**	$BF_3 \cdot Et_2O$, CH_2Cl_2, rt, Ar, 3 h	31	157.5-159/ light yellow
**(2g)**	$BF_3 \cdot Et_2O$, CH_2Cl_2, rt, Ar, 3 days	16	107-109 / light yellow
**(2h)**	$BF_3 \cdot Et_2O$, CH_2Cl_2, rt, Ar, 3 days	20.5	105.5-106.5 / light yellow
**(2i)**	$BF_3 \cdot Et_2O$, CH_2Cl_2, rt, Ar, 4 days	9	74-75.5 / light yellow
**(2j)**	$BF_3 \cdot Et_2O$, CH_2Cl_2, rt, Ar, 4 days	9	65-66 / light yellow

General procedure of synthesis of compounds (4a-c) according to [6] (Scheme 2 in the text):

A mixture of **3a-c** (1 mmol), Pd/C (88 mg) in ethanol (10 mL) was heated to reflux and then hydrazine monohydrate (1.33 mL) was added dropwise. The mixture was refluxed for 3 hours and then filtrated off through celite. After filtration the mixture was cooled at -18ºC overnight. The precipitate that was formed was filtrated off and washed 2 times with cold ethanol. The solid was dried in vacuo to obtain products (**4a-c**).

General procedure of synthesis of compounds (4d-j) (Scheme 2 in the text):

A mixture of **3d-j** (1 mmol), Pd/C (120 mg) in ethanol (10 mL) was heated to reflux and then hydrazine monohydrate (1.8 mL) was added dropwise. The mixture was refluxed for 8 hours and then filtrated off through celite. After filtration the mixture was extracted twice with DCM. The combined organic layers were dried over anhydrous magnesium sulfate. The mixture was concentrated in a vacuum to obtain products (**4d-j**).

Product	Conditions	Yield [%]	Melting point [ºC] / color
**(4a)**	$NH_2 \cdot NH_2$, Pd/C, EtOH, reflux, 3 h	76	136-138 / white
**(4b)**	$NH_2 \cdot NH_2$, Pd/C, EtOH, reflux, 3 h	69	135-137 / white

**(4c)**	$NH_2 \cdot NH_2$, Pd/C, EtOH, reflux, 3 h	66	120.5-121.5 / white
**(4d)**	$NH_2 \cdot NH_2$, Pd/C, EtOH, reflux, 8 h	98.6	134-135.5 / dark brown
**(4e)**	$NH_2 \cdot NH_2$, Pd/C, EtOH, reflux, 8 h	97.9	100-102 / grey
**(4f)**	$NH_2 \cdot NH_2$, Pd/C, EtOH, reflux, 8 h	99.9	96-98 / dark red
**(4g)**	$NH_2 \cdot NH_2$, Pd/C, EtOH, reflux, 8 h	96.8	85.5-86.5 / dark grey
**(4h)**	$NH_2 \cdot NH_2$, Pd/C, EtOH, reflux, 8 h	99.8	124.5-126 / brown
**(4i)**	$NH_2 \cdot NH_2$, Pd/C, EtOH, reflux, 8 h	77.6	98-100 / black
**(4j)**	$NH_2 \cdot NH_2$, Pd/C, EtOH, reflux, 8 h	88.9	102-104 / dark red

General procedure of synthesis of compounds (5a-f) (Scheme 2and scheme 4 in the text): We performed the synthesis of compounds **(5a-f)** using the procedure described in Borys, F. et al. Systematic studies on *anti*-cancer evaluation of stilbene and dibenzo[*b,f*]oxepine derivatives. *Molecules,* **2023** 28, 3558, https://doi.org/10.3390/ molecules28083558

General procedure of synthesis of compounds (7a-e) (Scheme 4 in the text):

p-fluoroaniline (5 mmol) was dissolved in DCM (25 mL), and a solution of oxone (10 mmol) in water (25 mL) was added to the mixture. The biphasic mixture was stirred for 3 hours at room temperature. The organic layer was separated, and the aqueous layer was extracted twice with DCM. The combined organic layers were dried over anhydrous magnesium sulfate. The mixture was concentrated in a vacuum and further used without purification. In the second step, 1-fluoro-4-nitrosobenzene and a proper derivative of dibenzo[*b,f*]oxepine (**5a-b**, **5d-e**) (2.5 mmol) were dissolved in glacial acetic acid (25 mL) and stirred overnight. The solution was diluted with water and extracted with ethyl acetate. The organic phase was concentrated in vacuo, and the crude product was purified by flash chromatography (DCM) to obtain the products (**7a-e**)

Characterisation Date of (7a-e)

Product	Conditions	Yield [%]	Color
**(7a)**	1-fluoro-4-nitrosobenzene, CH_3COOH, rt, 16 h	49	orange
**(7b)**	1-fluoro-4-nitrosobenzene, CH_3COOH, rt, 16 h	68	orange
**(7c)**	1-fluoro-4-nitrosobenzene, CH_3COOH, rt, 16 h	56	orange

**(7d)**	1-fluoro-4-nitrosobenzene, CH_3COOH, rt, 16 h	50	orange
**(7e)**	1-fluoro-4-nitrosobenzene, CH_3COOH, rt, 16 h	61	orange

General procedure of synthesis of compounds (9c-d) (Scheme 5 in the text):

Compound **9a-b** (0.15 mmol) was dissolved in acetic acid (50 mL), and after dissolution, activated zinc was added (30 mmol). The mixture was stirred for 48 hours at room temperature. After the reaction, the mixture was filtered through Celite and then concentrated in vacuo. 1 M HCl (75 mL) was added to the crude product, and the resulting mixture was filtered. A water solution of NaOH was added to the filtrate till the pH reached 10. The resulting mixture was cooled at 5ºC for 12 h and then filtered to obtain compounds 9c-d as grey solids.

Characterisation Date of (9c-d)

Product	Conditions	Yield [%]	Melting point [ºC] / color
**(9c)**	Zn, CH_3COOH, rt, 48 h	71	Decomposition above 255 / grey
**(9d)**	Zn, CH_3COOH, rt, 48 h	63	Decomposition above 255 / grey

General procedure of synthesis of compounds (9e-f) (Scheme 5 in the text):

Compound **9c-d** (172 µmol) was dissolved in acetic acid (250 mL), and after dissolution, 1-fluoro-4-nitrosobenzene (34.4 mmol) was added. The mixture was stirred for 4 days at room temperature. After the reaction, the mixture was concentrated in a vacuum and purified by flash chromatography (hexane/ethyl acetate 3/1) to give compounds **9e-f** as red solids.

Characterisation Date of (9e-f)

Product	Conditions	Yield [%]	Melting point [ºC] / color
**(9e)**	1,3-difluoro-2-nitrosobenzene, CH_3COOH, rt, 6 days	2	Decomposition above 260 / grey

**(9f)**	1-fluoro-4-nitrosobenzene, CH_3COOH, rt, 4 days	5	Decomposition above 250 / grey

3. NMR spectra of obtained compounds

All the spectra were recorded using a Varian VNMRS spectrometer operating at 11.7 T and Varian Mercury VX 9.4 T magnetic field. Measurements were performed for ca. 1.0 M solutions of all the compounds in DMSO-d_6 or $CDCl_3$. The residual signals of DMSO-d6 (2.54 ppm) and $CDCl_3$ (7.26 ppm) in ^1H NMR and of the DMSO-d_6 signal (40.4 ppm) and of $CDCl_3$ (77.0 ppm) in ^{13}C NMR spectra were used as the chemical shift references. Spin multiplicities are described as s (singlet), d(doublet), t (triplet), q (quartet), m (multiplet), dd (double doublet). Coupling constants are reported in Hertz. All the proton spectra were recorded using the standard spectrometer software and parameters set: acquisition time 3s, pulse angle 30°. The standard measurement parameter set for ^{13}C NMR spectra was: pulse width 7 μs (the 90° pulse width was 12.5 μs), acquisition time 1 s, spectral width 200 ppm, 1000 scans of 32 K data point were accumulated and after zero-filling to 64 K; and the FID signals were subjected to Fourier transformation after applying a 1 Hz line broadening. The ^1H-^{13}Cgs-HSQC and ^1H-^{13}Cgs-HMBC spectra were also recorded using the standard Varian software.

Copies of ^1H NMR , ^{13}C NMR and ^{19}F NMR spectra of (2b-j), (4a-j), (7a-e), (9c-d) and (9e-f)

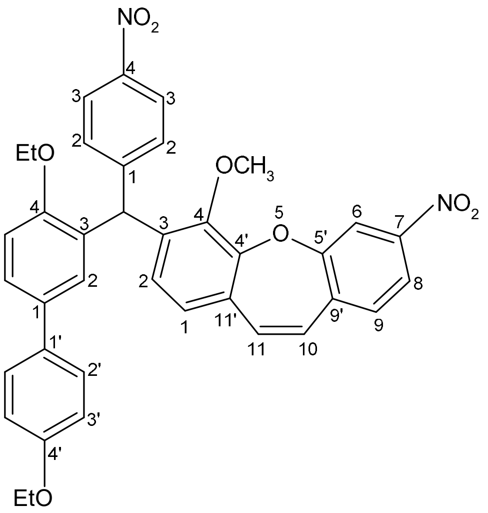

2b

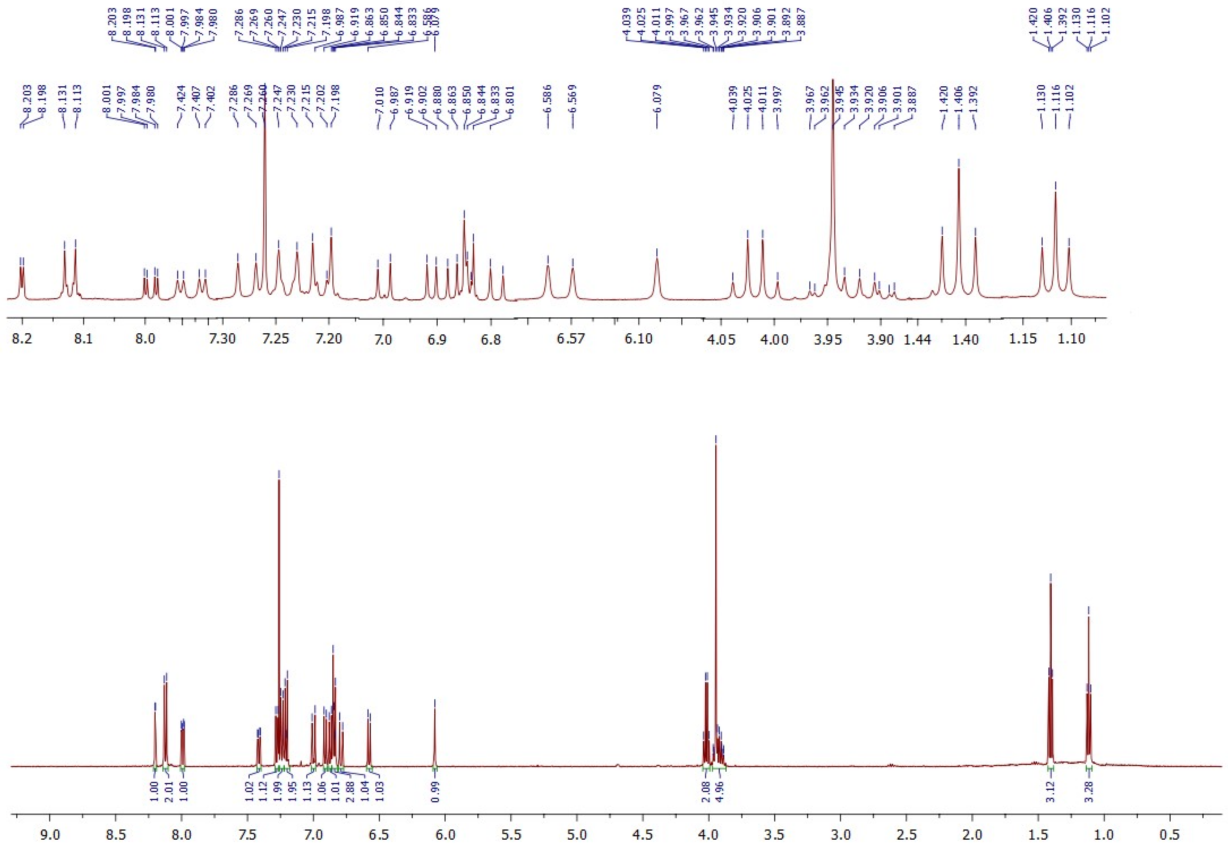

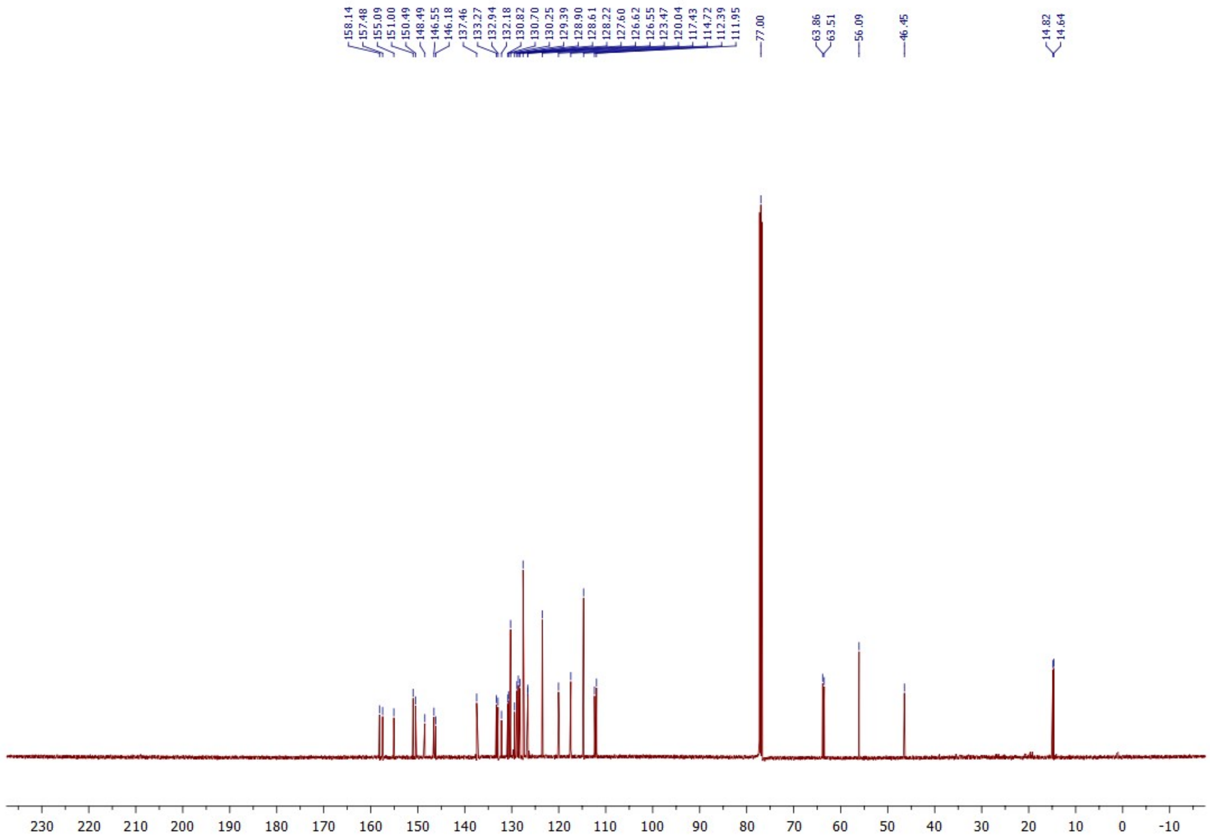

^{1}H NMR (500 MHz, $CDCl_3$, 298 K): δ (ppm): **Biphenyl rings**: 7.41 (1H, dd, $J_{H5,H6}$ = 8.5 Hz, $J_{H2,H6}$ = 2.5 Hz, H_6), 7.21 (2H, d, $J_{H2',H3'}$ = 8.5 Hz, $H_{2'}$), 6.91 (1H, d, H_5), 6.85 (1H, d, H_2), 6.84 (2H, d, $H_{3'}$), 4.02 (2H, q, $J_{OCH2,CH3}$ = 7 Hz, OCH_2), 3.93 (2H, m, OCH_2), 1.41 (3H, t, CH_3), 1.12 (3H, t, CH_3); **Dibenzo[*b*,*f*]oxepine ring**: 8.20 (1H, d, $J_{H6,H8}$ = 2 Hz, H_6), 7.99 (1H, dd, $J_{H8,H9}$ = 8.5 Hz, H_8), 7.28 (1H, d, H_9), 7.00 (1H, AB spin system, d, $J_{H10,H11}$ = 11.5 Hz, H_{11}), 6.87 (1H, d, $J_{H1,H2}$ = 8.5 Hz, H_1), 6.79 (1H, AB spin system, d, H_{10}), 6.58 (1H, d, H_2), 3.95 (3H, s, OCH_3); **Linker**: 8.12 (2H, d, $J_{H2,H3}$ = 9 Hz, H_3), 7.24 (2H, d, H_2), 6.08 (1H, s, CH).

^{13}C NMR (125 MHz, $CDCl_3$, 298 K): δ (ppm): 158.14, 157.48, 155.09, 151.00, 150.49, 148.49, 146.55, 146.18, 137.46, 133.27, 132.94, 132.18, 130.82, 130.70, 130.25, 129.39, 128.90, 128.61, 128.22, 127.60, 126.62, 126.55, 123.47, 120.04, 117.43, 114.72, 112.39, 111.95, 63.86, 63.51, 56.09, 46.45, 14.82, 14.64.

HRMS (ESI): *m/z* calculated for $C_{38}H_{32}N_2O_8$ 644.21532, found: 644.21502.

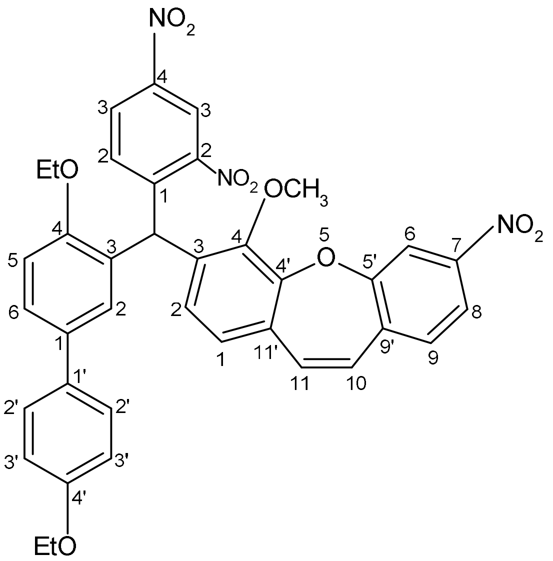

2c

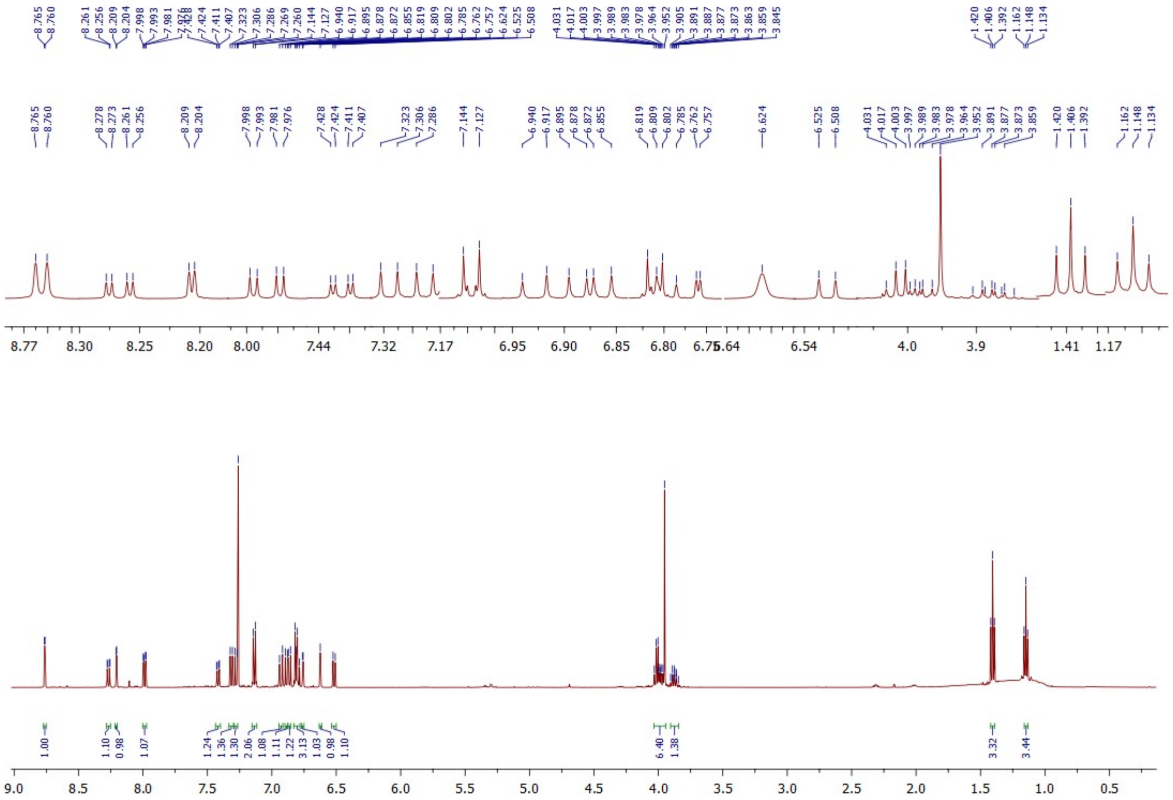

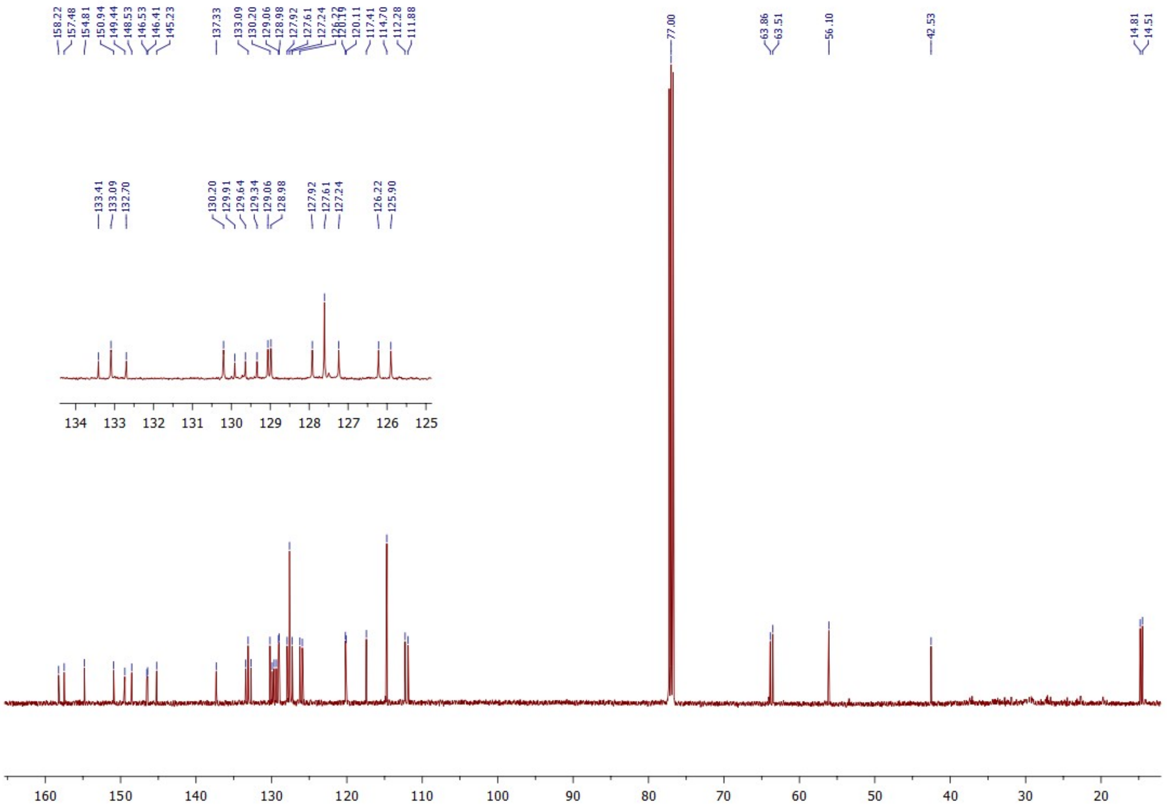

^{1}H NMR (500 MHz, $CDCl_3$, 298 K): δ (ppm): **Biphenyl rings**: 7.42 (1H, dd, $J_{H5,H6}$ = 8.5 Hz, $J_{H3,H5}$ = 2.5 Hz, H_5), 7.14 (2H, d, $J_{H2',H3'}$ = 8.5 Hz, $H_{2'}$), 6.89 (1H, d, H_6), 6.81 (2H, d, $H_{3'}$), 6.76 (1H, d, H_3), 4.01 (2H, q, $J_{OCH2,CH3}$ = 7 Hz, OCH_2), 3.98 (1H, qd, $J_{OCH2,CH3}$ = 7 Hz, $J_{OCH2,OCH2}$ = 2 Hz, OCH_2), 3.88 (1H, qd, OCH_2), 1.41 (3H, t, CH_3), 1.15 (3H, t, CH_3); **Dibenzo[*b,f*]oxepine ring**: 8.21 (1H, d, $J_{H6,H8}$ = 2.5 Hz, H_6), 7.99 (1H, dd, $J_{H8,H9}$ = 8.5 Hz, H_8), 7.28 (1H, d, H_9), 6.93 (1H, AB spin system, d, $J_{H10,H11}$ = 11.5 Hz, H_{11}), 6.86 (1H, d, $J_{H1,H2}$ = 8.5 Hz, H_1), 6.80 (1H, AB spin system, d, H_{10}), 6.52 (1H, d, H_2), 3.95 (3H, s, OCH_3); **Linker**: 8.76 (1H, d, $J_{H2,H4}$ = 2.5 Hz, H_2), 8.24 (1H, dd, $J_{H4,H5}$ = 8.5 Hz, H_4), 7.31 (1H, d, H_5), 6.62 (1H, s, CH).

^{13}C NMR (125 MHz, $CDCl_3$, 298 K): δ (ppm): 158.22, 157.48, 154.81, 150.94, 149.44, 148.53, 146.53, 146.41, 145.23, 137.33, 133.41, 133.09, 132.70, 130.20, 129.91, 129.64, 129.34, 129.06, 128.98, 127.92, 127.61, 127.24, 126.22, 125.90, 120.19, 120.11, 117.41, 114.70, 112.28, 111.88, 63.86, 63.51, 56.10, 42.53, 14.81, 14.51.

HRMS (ESI): *m/z* calculated for $C_{38}H_{31}N_3O_{10}$ 689.20040, found: 689.20020.

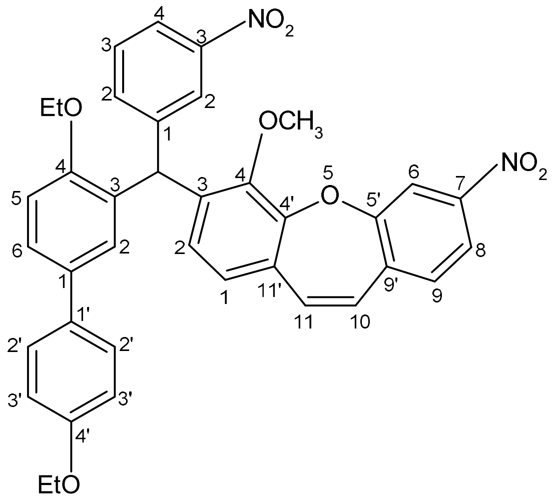

NO2
4
3
3
2
2
EtO
1
OCH3
4
3
4
5
6
7
NO2
5
3
4'
O
5'
6
2
2
8
1
11'
9'
9
1'
11
10
2'
2'
3'
3'
4'
EtO

2d

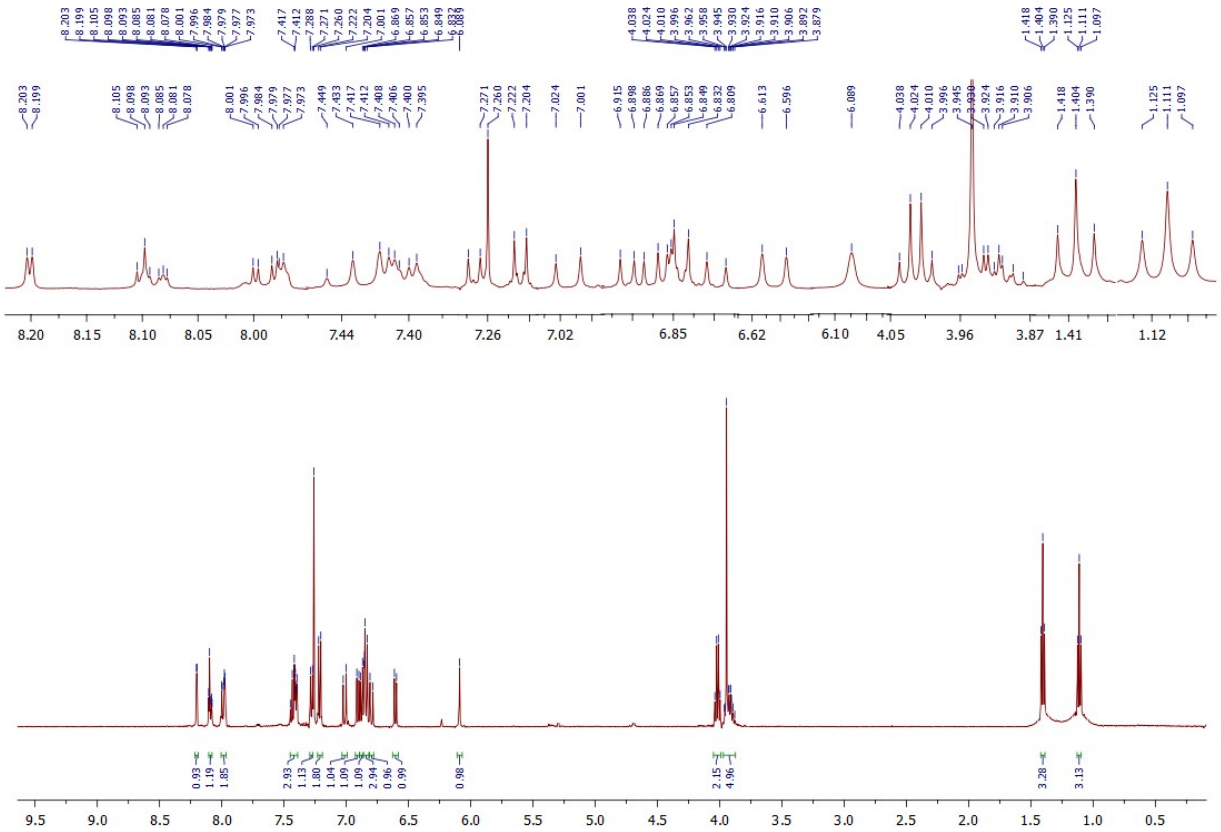

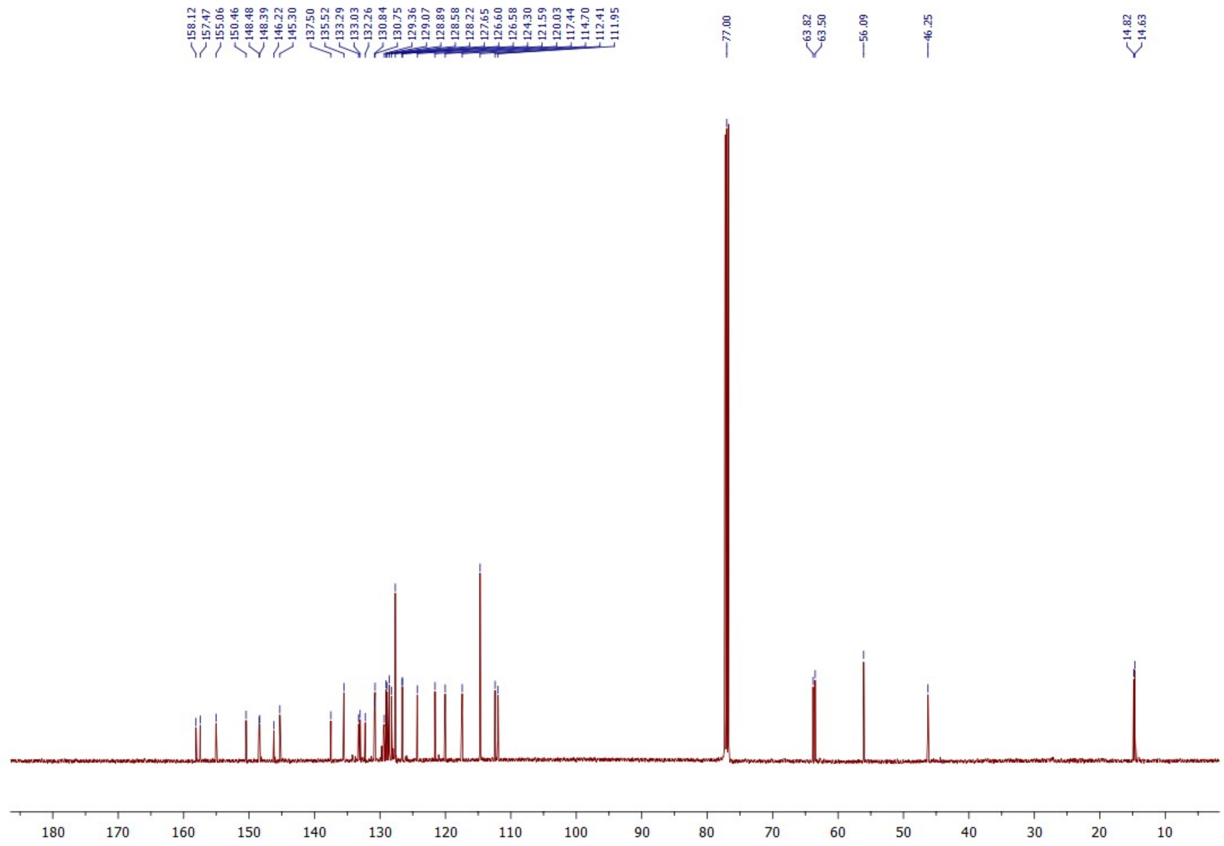

^{1}**H NMR** (500 MHz, $CDCl_3$, 298 K): δ (ppm): **Biphenyl rings**: 7.41 (1H, dd, $J_{H5,H6}$ = 8.5 Hz, $J_{H2,H6}$ = 2.5 Hz, H_6), 7.21 (2H, d, $J_{H2',H3'}$ = 9 Hz, $H_{2'}$), 6.91 (1H, d, H_5), 6.86 (1H, d, H_2), 6.84 (2H, d, $H_{3'}$), 4.02 (2H, q, $J_{OCH2,CH3}$ = 7 Hz, OCH_2), 3.92 (2H, m, OCH_2), 1.40 (3H, t, CH_3), 1.11 (3H, t, CH_3); **Dibenzo[*b*,*f*]oxepine ring**: 8.20 (1H, d, $J_{H6,H8}$ = 2.5 Hz, H_6), 7.99 (1H, dd, $J_{H8,H9}$ = 8.5 Hz, H_8), 7.28 (1H, d, H_9), 7.01 (1H, AB spin system, d, $J_{H10,H11}$ = 11.5 Hz, H_{11}), 6.88 (1H, d, $J_{H1,H2}$ = 8.5 Hz, H_1), 6.80 (1H, AB spin system, d, H_{10}), 6.60 (1H, d, H_2), 3.95 (3H, s, OCH_3); **Linker**: 8.09 (1H, m, H_4), 7.98 (1H, t, $J_{H2,H4,6}$ = 2 Hz, H_2), 7.43 (1H, t, $J_{H5,H4,6}$ = 8 Hz, H_5), 7.40 (1H, m, H_6), 6.09 (1H, s, CH).

^{13}**C NMR** (125 MHz, $CDCl_3$, 298 K): δ (ppm): 158.12, 157.47, 155.06, 150.46, 148.48, 148.39, 146.22, 145.30, 137.50, 135.52, 133.29, 133.03, 132.26, 130.84, 130.75, 129.36, 129.07, 128.89, 128.58, 128.22, 127.65, 126.60, 126.58, 124.30, 121.59, 120.03, 117.44, 114.70, 112.41, 111.95, 63.82, 63.50, 56.09, 46.25, 14.82, 14.63.

HRMS (ESI): *m/z* calculated for $C_{38}H_{32}N_2O_8$ 644.21532, found: 644.21572.

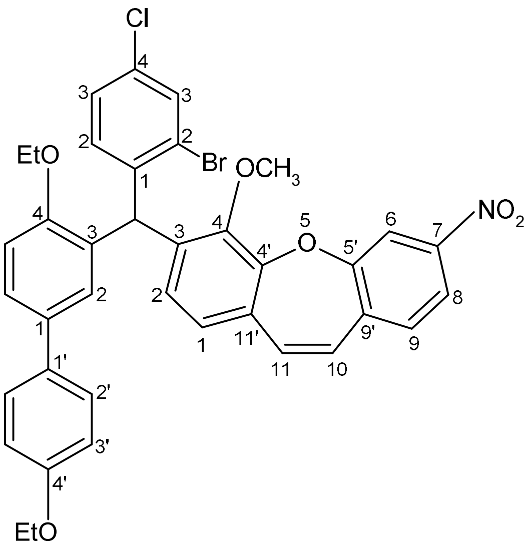

2e

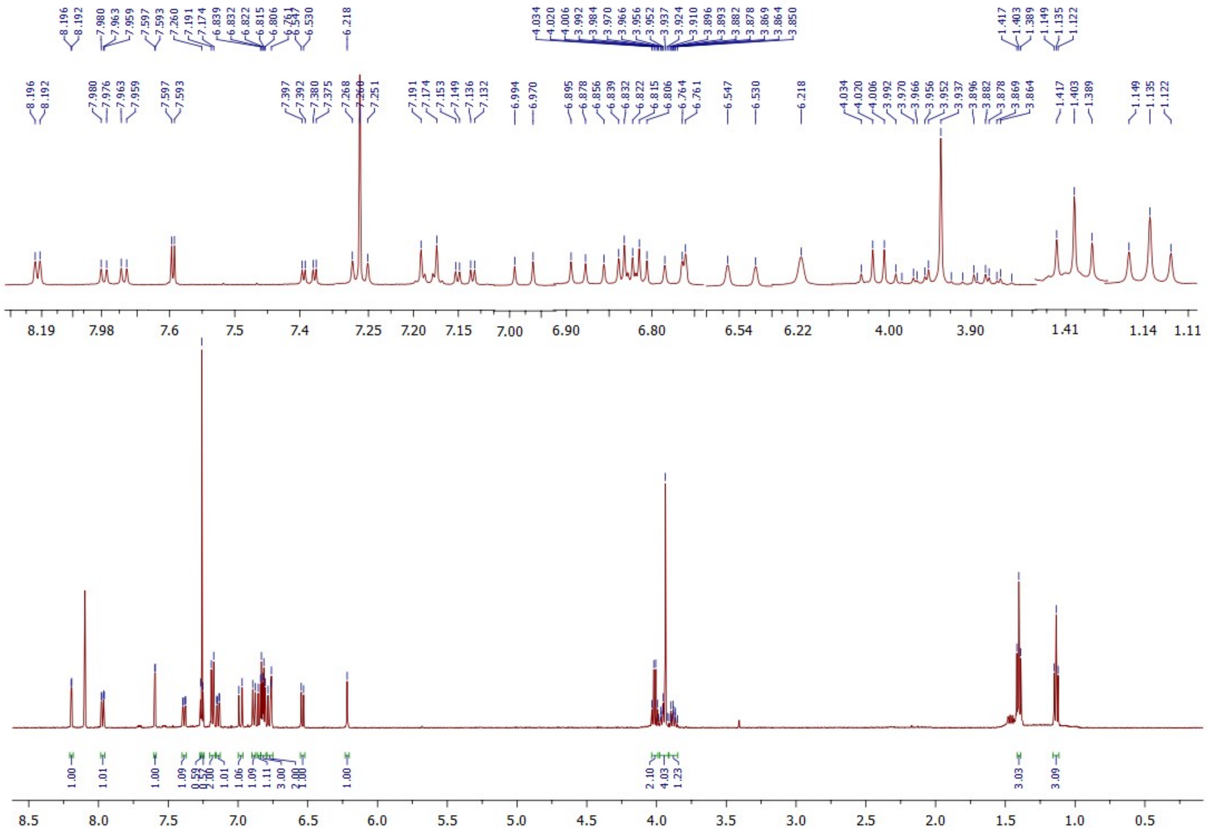

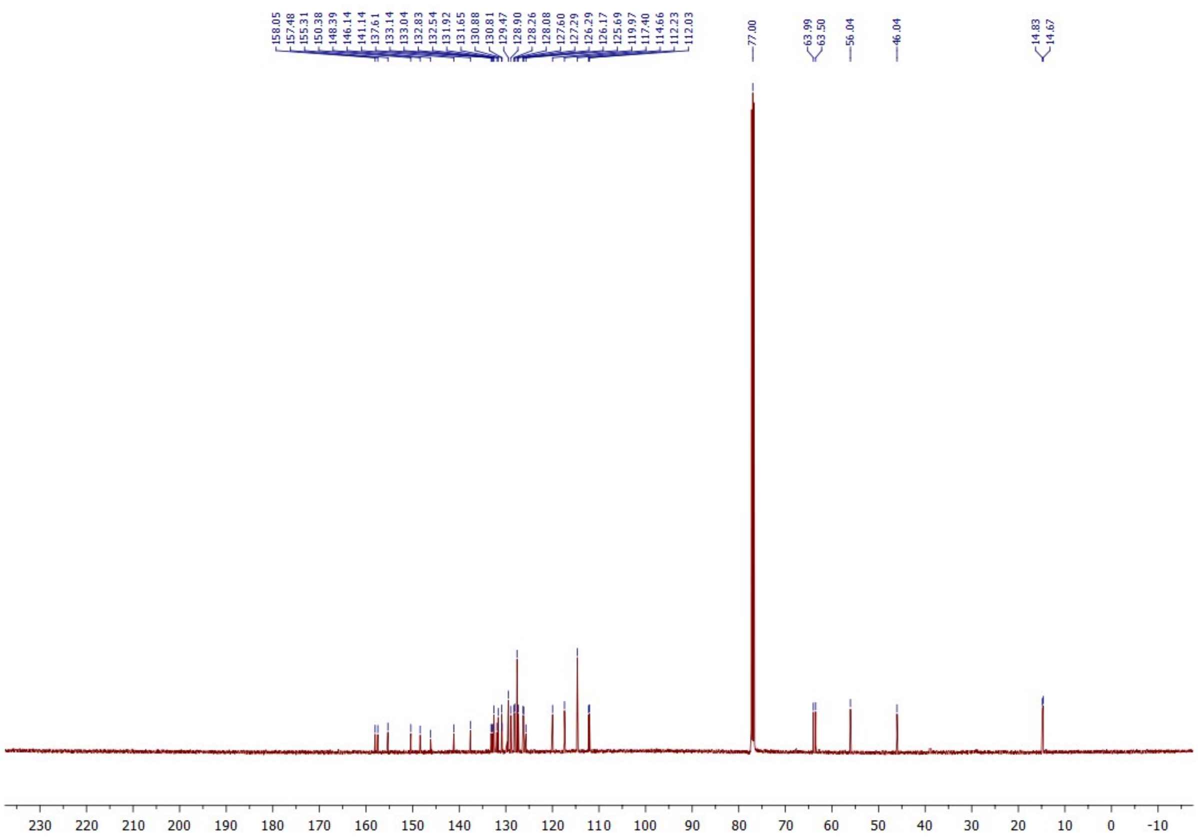

^{1}H NMR (500 MHz, $CDCl_3$, 298 K): δ (ppm): **Biphenyl rings**: 7.39 (1H, dd, $J_{H5.H6}$ = 8.5 Hz, $J_{H2,H6}$ = 2.5 Hz, H_6), 7.18 (2H, d, $J_{H2',H3'}$ = 8.5 Hz, $H_{2'}$), 6.89 (1H, d, H_5), 6.82 (2H, d, $H_{3'}$), 6.76 (1H, d, H_2), 4.01 (2H, q, $J_{OCH2,CH3}$ = 7 Hz, OCH_2), 3.95 (1H, qd, $J_{OCH2,CH3}$ = 7 Hz, $J_{OCH2,OCH2}$ = 2 Hz, OCH_2), 3.88 (1H, qd, OCH_2), 1.40 (3H, t, CH_3), 1.14 (3H, t, CH_3); **Dibenzo[*b,f*]oxepine ring**: 8.19 (1H, d, $J_{H6,H8}$ = 2 Hz, H_6), 7.97 (1H, dd, $J_{H8,H9}$ = 8.5 Hz, H_8), 7.26 (1H, d, H_9), 6.98 (1H, AB spin system, d, $J_{H10.H11}$ = 12 Hz, H_{11}), 6.85 (1H, d, $J_{H1,H2}$ = 8.5 Hz, H_1), 6.77 (1H, AB spin system, d, H_{10}), 6.54 (1H, d, H_2), 3.94 (3H, s, OCH_3); **Linker**: 7.60 (1H, d, $J_{H3,H5}$ = 2 Hz, H_3), 7.14 (1H, dd, $J_{H5,H6}$ = 8.5 Hz, H_5), 6.81 (1H, d, H_6), 6.22 (1H, s, CH).

^{13}C NMR (125 MHz, $CDCl_3$, 298 K): δ (ppm): 158.05, 157.48, 155.31, 150.38, 148.39, 146.14, 141.14, 137.61, 133.14, 133.04, 132.83, 132.54, 131.92, 131.65, 130.88, 130.81, 129.47, 128.90, 128.26, 128.08, 127.60, 127.29, 126.29, 126.17, 125.69, 119.97, 117.40, 114.66, 112.23, 112.03, 63.99, 63.50, 56.04, 46.04, 14.83, 14.67.

HRMS (ESI): *m/z* calculated for $C_{38}H_{31}BrClNO_6$ 711.10178, found: 711.10167.

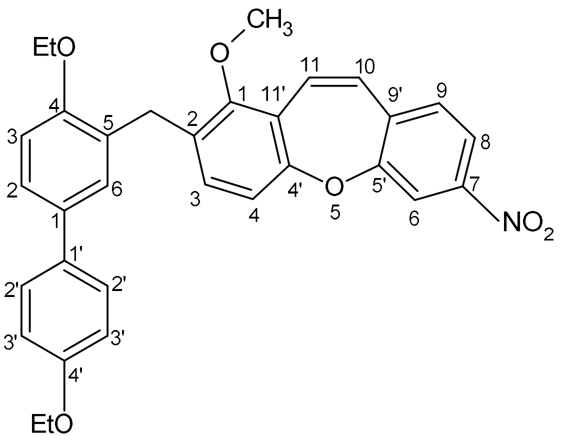

2f

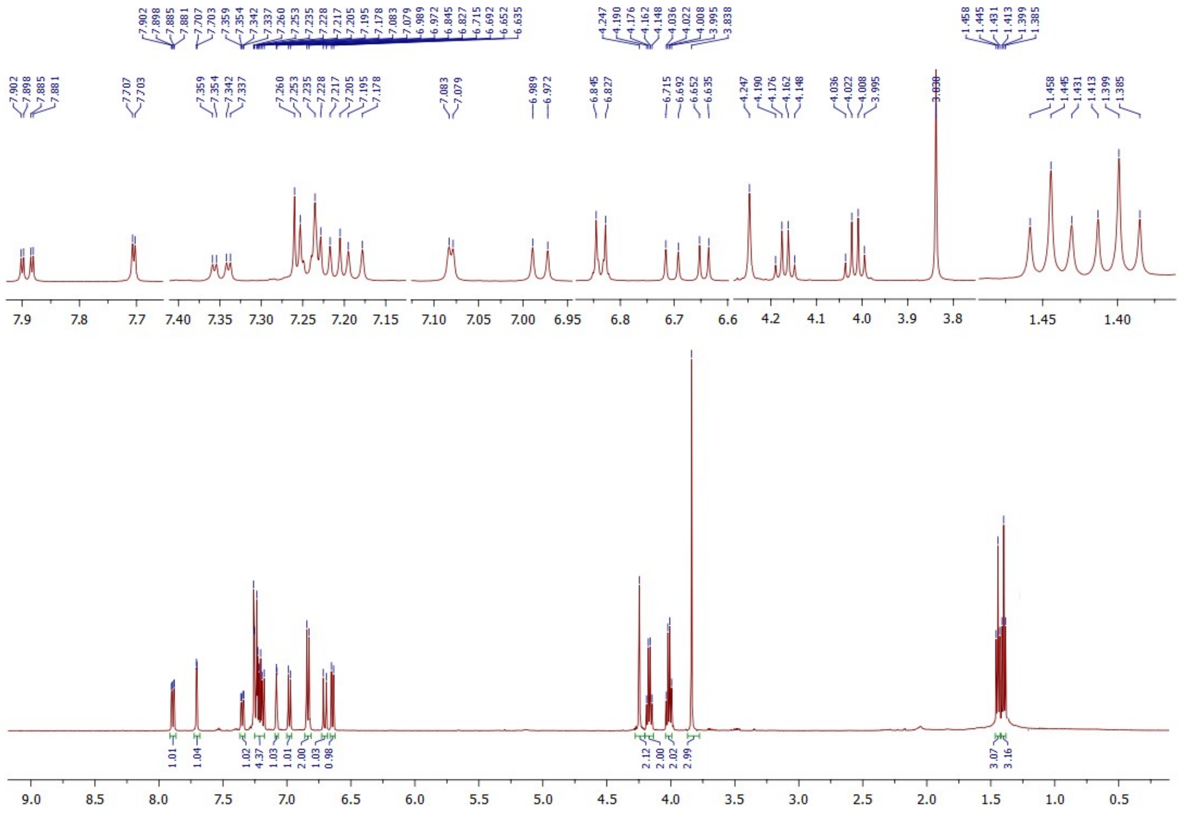

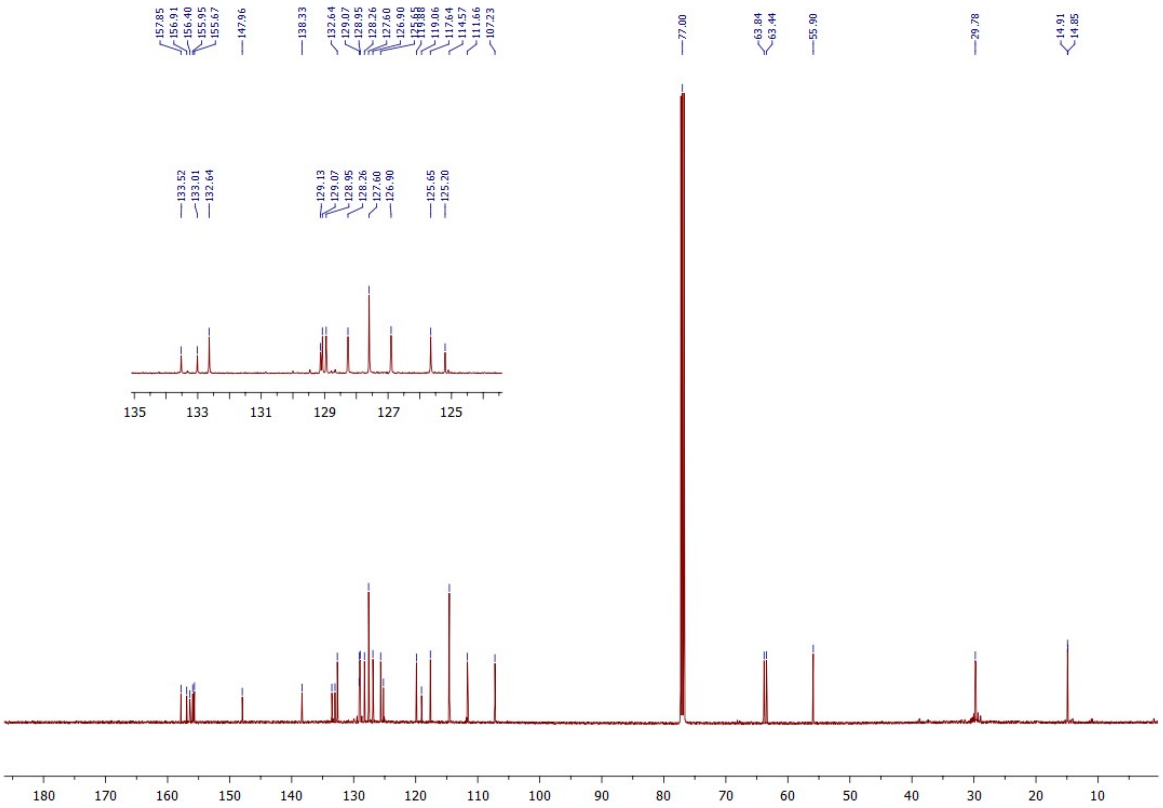

^{1}H NMR (500 MHz, $CDCl_3$, 298 K): δ (ppm): **Biphenyl rings**: 7.35 (1H, dd, $J_{H2,H3}$ = 8.5 Hz, $J_{H2,H6}$ = 2.5 Hz, H_2), 7.24 (2H, d, $J_{H2',H3'}$ = 9 Hz, $H_{2'}$), 7.08 (1H, d, H_6), 6.98 (1H, d, H_3), 6.84 (2H, d, $H_{3'}$), 4.17 (2H, q, $J_{OCH2,CH3}$ = 7 Hz, OCH_2), 4.02 (2H, q, $J_{OCH2,CH3}$ = 7 Hz, OCH_2), 1.45 (3H, t, CH_3), 1.40 (3H, t, CH_3); **Dibenzo[*b,f*]oxepine ring**: 7.89 (1H, dd, $J_{H8,H9}$ = 8.5 Hz, $J_{H6,H8}$ = 2 Hz, H_8), 7.71 (1H, d, H_6), 7.23 (1H, d, H_9), 7.22 (1H, AB spin system, d, $J_{H10,H11}$ = 11.5 Hz, H_{11}), 7.19 (1H, d, $J_{H3,H4}$ = 8.5 Hz, H_3), 6.70 (1H, AB spin system, d, H_{10}), 6.64 (1H, d, H_4), 3.84 (3H, s, OCH_3); **Linker**: 4.25 (2H, s, CH_2).
^{13}C NMR (125 MHz, $CDCl_3$, 298 K): δ (ppm): 157.85, 156.91, 156.40, 155.95, 155.67, 147.96, 138.33, 133.52, 133.01 132.64, 129.13, 129.07, 128.95, 128.26, 127.60, 126.90, 125.65, 125.20, 119.88, 119.06, 117.64, 114.57, 111.66, 107.23, 63.84, 63.44, 55.90, 29.78, 14.91, 14.85.
HRMS (ESI): *m/z* calculated for $C_{32}H_{29}NO_6$ 523.19948, found: 523.19920.

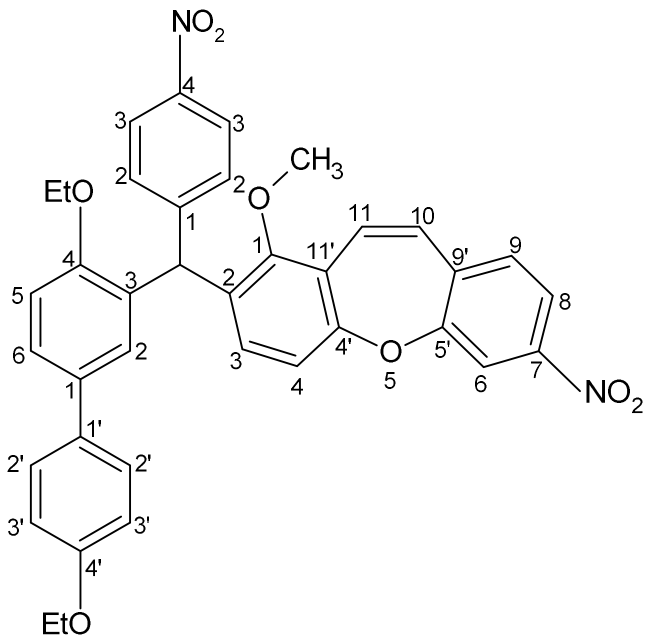

NO2
4
3
3
2
2
1
CH3
O
EtO
11
10
9'
9
4
3
1
11'
2
5
8
6
2
4'
O
5'
3
4
5
6
7
NO2
1
1'
2'
2'
3'
3'
4'
EtO

2g

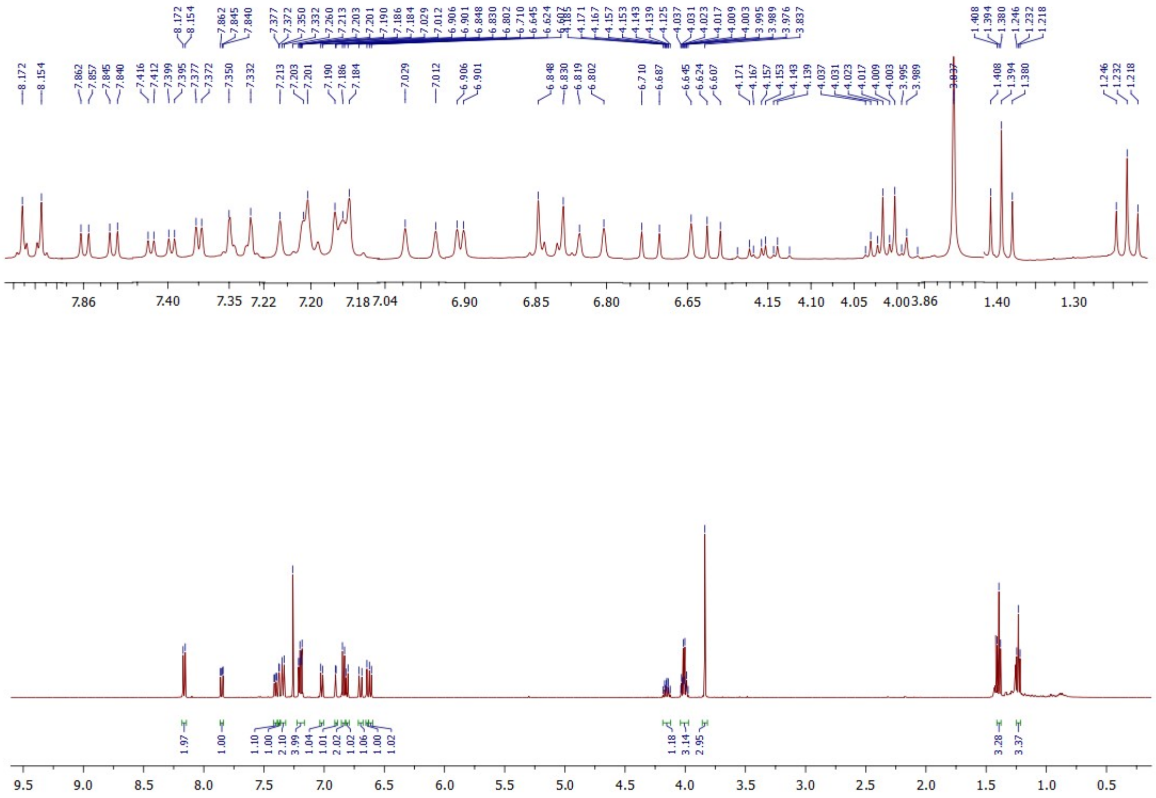

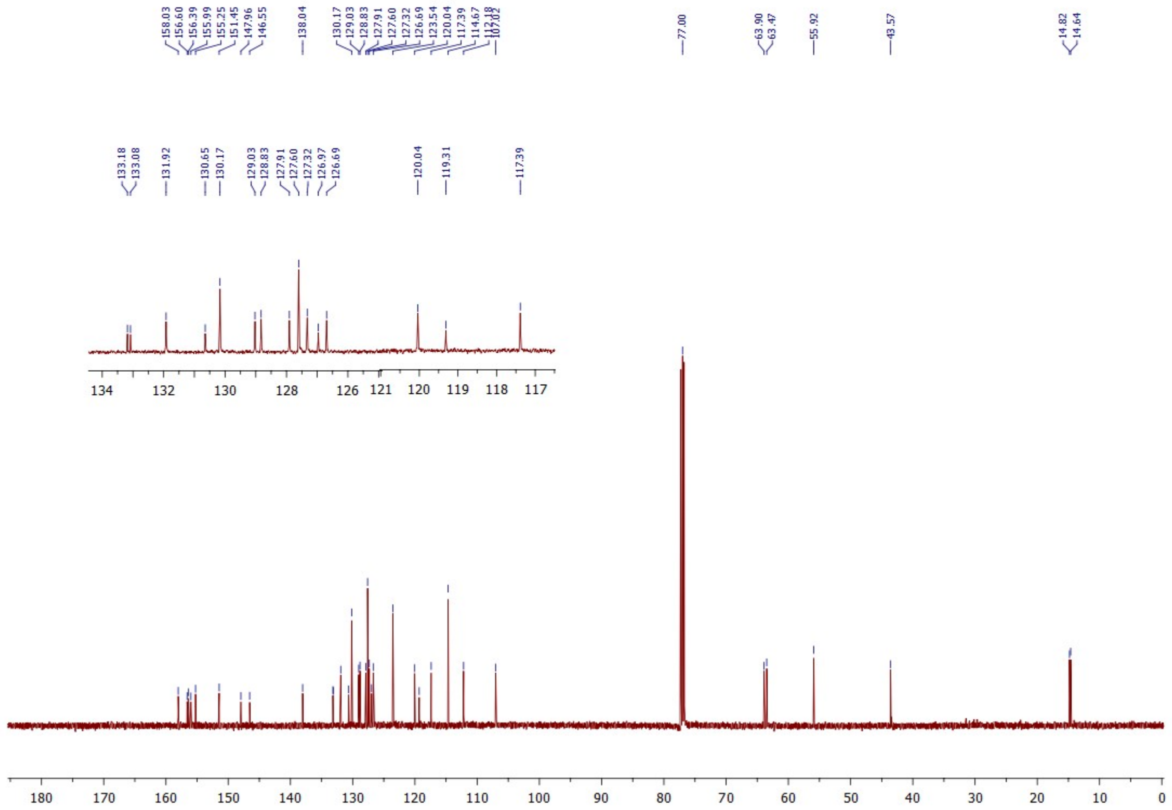

^{1}H NMR (500 MHz, $CDCl_3$, 298 K): δ (ppm): **Biphenyl rings**: 7.41 (1H, dd, $J_{H5,H6}$ = 8.5 Hz, $J_{H2,H6}$ = 2 Hz, H_6), 7.19 (2H, d, $J_{H2',H3'}$ = 9 Hz, $H_{2'}$), 7.02 (1H, d, H_5), 6.90 (1H, d, H_2), 6.84 (2H, d, $H_{3'}$), 4.16 (1H, qd, $J_{OCH2,CH3}$ = 7 Hz, $J_{OCH2,OCH2}$ = 2 Hz, OCH_2), 4.01 (1H, qd, OCH_2), 4.01 (2H, q, $J_{OCH2,OCH3}$ = 7 Hz, OCH_2), 1.39 (3H, t, CH_3), 1.23 (3H, t, CH_3); **Dibenzo[*b,f*]oxepine ring**: 7.85 (1H, dd, $J_{H8,H9}$ = 8.5 Hz, $J_{H6,H8}$ = 2.5 Hz, H_8), 7.37 (1H, d, H_6), 7.20 (1H, AB spin system, d, $J_{H10,H11}$ = 11.5 Hz, H_{11}), 7.19 (1H, d, H_9), 6.81 (1H, d, $J_{H3,H4}$ = 8.5 Hz, H_3), 6.70 (1H, AB spin system, d, H_{10}), 6.62 (1H, d, H_4), 3.84 (3H, s, OCH_3); **Linker**: 8.16 (2H, d, $J_{H2,H3}$ = 9 Hz, H_3), 7.34 (2H, d, H_2), 6.65 (1H, s, CH).

^{13}C NMR (125 MHz, $CDCl_3$, 298 K): δ (ppm): **Biphenyl rings**: 158.03 ($C_{4'}$), 155.25 (C_4), 133.18 ($C_{1'}$), 133.08 (C_1), 130.65 (C_3), 127.91 (C_2), 127.60 ($C_{2'}$), 126.69 (C_6), 114.67 ($C_{3'}$), 112.18 (C_5), 63.90 (OCH_2), 63.47 (OCH_2), 14.82 (CH_3), 14.64 (CH_3); **Dibenzo[*b,f*]oxepine ring**: 156.60 ($C_{5'}$), 156.39 (C_1), 155.99 ($C_{4'}$), 147.96 (C_7), 138.04 ($C_{9'}$), 131.92 (C_3), 129.03 (C_9), 128.83 (C_{11}), 127.32 (C_{10}), 126.97 (C_2), 120.04 (C_8), 119.31 ($C_{11'}$), 117.39 (C_6), 107.02 (C_4), 55.92 (OCH_3); **Linker**: 151.45 (C_1), 146.55 (C_4), 130.17 (C_2), 123.54 (C_3), 43.57 (CH).

HRMS (ESI): *m/z* calculated for $C_{38}H_{32}N_2O_8$+H 645.22314, found: 645.22302.

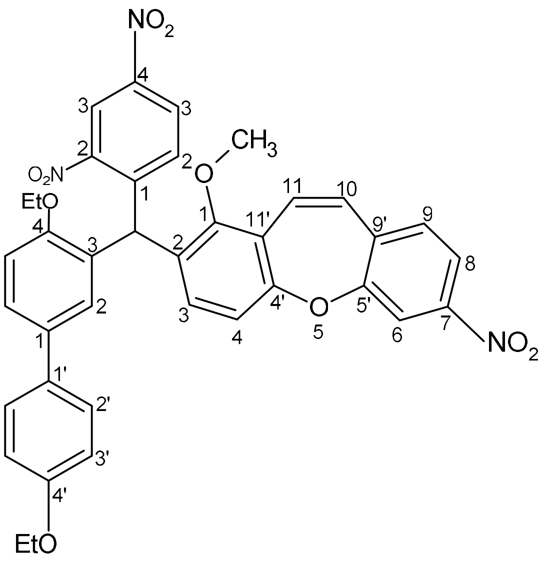

2h

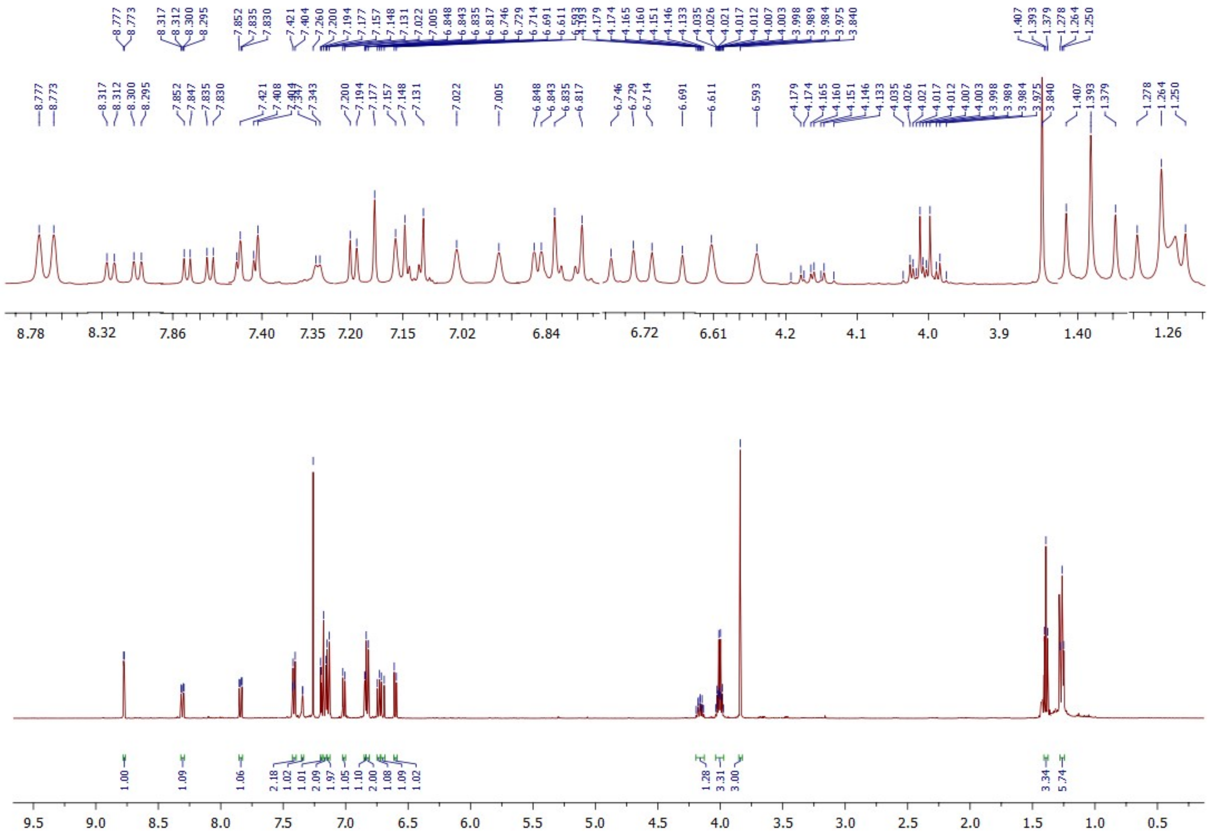

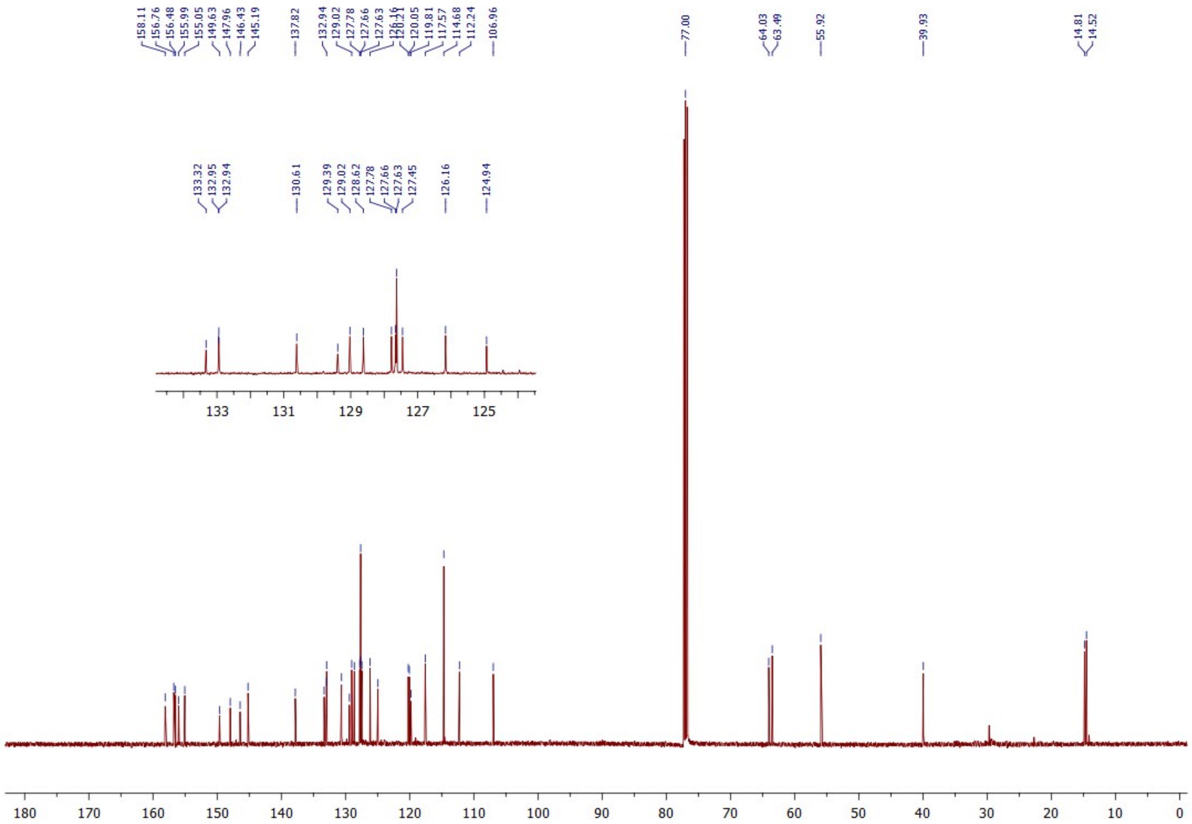

[1]**H NMR** (500 MHz, $CDCl_3$, 298 K): δ (ppm): **Biphenyl rings**: 7.41 (1H, dd, $J_{H5,H6}$ = 8.5 Hz, $J_{H2,H6}$ = 2.5 Hz, H_6), 7.14 (2H, d, $J_{H2',H3'}$ = 8.5 Hz, $H_{2'}$), 7.01 (1H, d, H_5), 6.85 (1H, d, H_2), 6.83 (2H, d, $H_{3'}$), 4.16 (1H, qd, $J_{OCH2,CH3}$ = 7 Hz, $J_{OCH2,OCH2}$ = 2.5 Hz, OCH_2), 4.00 (1H, qd, OCH_2), 4.00 (2H, q, $J_{OCH2,CH3}$ = 7 Hz, OCH_2), 1.39 (3H, t, CH_3), 1.26 (3H, t, CH_3); **Dibenzo[*b,f*]oxepine ring**: 7.84 (1H, dd, $J_{H8.H9}$ = 8.5 Hz, $J_{H6,H8}$ = 2.5 Hz, H_8), 7.35 (1H, d, H_6), 7.19 (1H, AB spin system, d, $J_{H10.H11}$ = 11.5 Hz, H_{11}), 7.19 (1H, d, H_9), 6.74 (1H, d, $J_{H3,H4}$ = 8.5 Hz, H_4), 6.70 (1H, AB spin system, d, H_{10}), 6.60 (1H, d, H_3), 3.84 (3H, s, OCH_3); **Linker**: 8.78 (1H, d, $J_{H3,H5}$ = 2.5 Hz, H_3), 8.31 (1H, dd, $J_{H5,H6}$ = 8.5 Hz, H_5), 7.42 (1H, d, H_6), 7.16 (1H, s, CH).

[13]**C NMR** (125 MHz, $CDCl_3$, 298 K): δ (ppm): 158.11, 156.76, 156.48, 155.99, 155.05, 149.63, 147.96, 146.43, 145.19, 137.82, 133.32, 132.95, 132.94, 130.61, 129.39, 129.02, 128.62, 127.78, 127.66, 127.63, 127.45, 126.16, 124.94, 120.21, 120.05, 119.81, 117.57, 114.68, 112.24, 106.96, 64.03, 63.49, 55.92, 39.93, 14.81, 14.52.

HRMS (ESI): *m/z* calculated for $C_{38}H_{31}N_3O_{10}$ 689.20040, found: 689.19995.

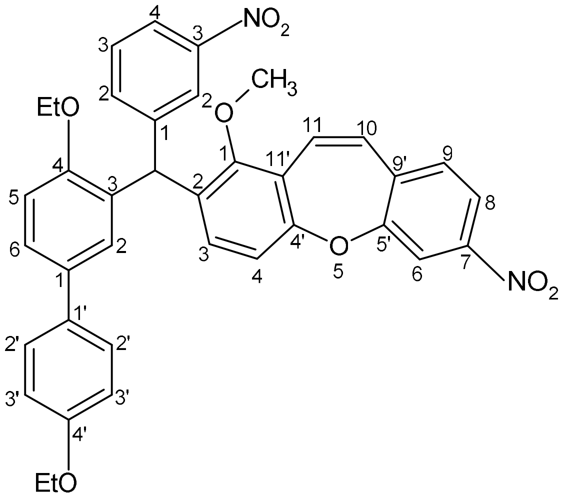

2i

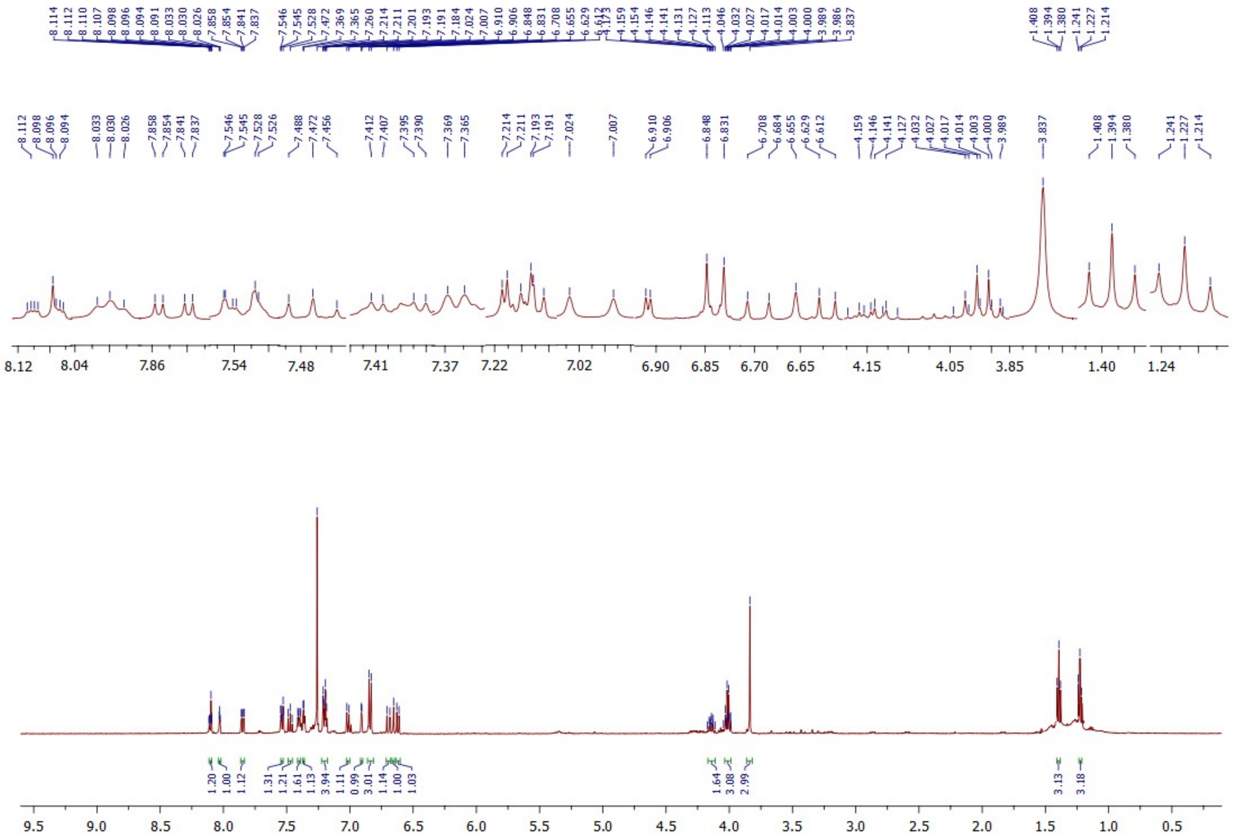

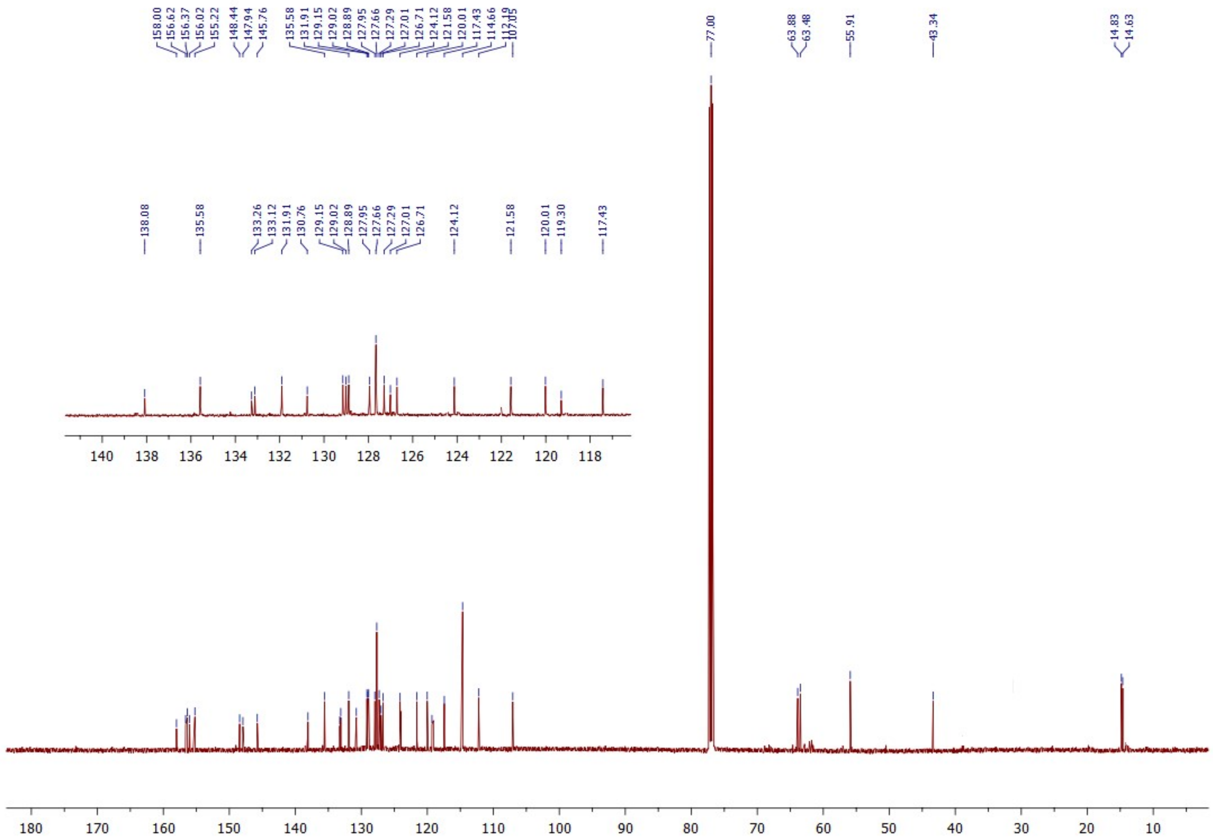

^{1}H NMR (500 MHz, $CDCl_3$, 298 K): δ (ppm): **Biphenyl rings**: 7.40 (1H, dd, $J_{H5,H6}$ = 8.5 Hz, $J_{H2,H6}$ = 2.5 Hz, H_6), 7.20 (2H, d, $J_{H2',H3'}$ = 9 Hz, $H_{2'}$), 7.02 (1H, d, H_5), 6.91 (1H, d, H_2), 6.84 (2H, d, $H_{3'}$), 4.14 (1H, qd, $J_{OCH2,CH3}$ = 7 Hz, $J_{OCH2,OCH2}$ = 2 Hz, OCH_2), 4.02 (1H, qd, OCH_2), 4.01 (2H, q, $J_{OCH2,CH3}$ = 7 Hz, OCH_2), 1.39 (3H, t, CH_3), 1.23 (3H, t, CH_3); **Dibenzo[*b,f*]oxepine ring**: 7.85 (1H, dd, $J_{H8,H9}$ = 8.5 Hz, $J_{H6,H8}$ = 2 Hz, H_8), 7.37 (1H, d, H_6), 7.20 (1H, AB spin system, d, $J_{H10,H11}$ = 11.5 Hz, H_{11}), 7.19 (1H, d, H_9), 6.84 (1H, d, $J_{H3,H4}$ = 8.5 Hz, H_4), 6.70 (1H, AB spin system, d, H_{10}), 6.62 (1H, d, H_3), 3.84 (3H, s, OCH_3); **Linker**: 8.10 (1H, m, H_4), 8.03 (1H, t, $J_{H2,H4,6}$ = 1.5 Hz, H_2), 7.54 (1H, m, H_6), 7.47 (1H, t, $J_{H5,H4,6}$ = 8 Hz, H_5), 6.66 (1H, s, CH).

^{13}C NMR (125 MHz, $CDCl_3$, 298 K): δ (ppm): 158.00, 156.62, 156.37, 156.02, 155.22, 148.44, 147.94, 145.76, 138.08, 135.58, 133.26, 133.12, 131.91, 130.76, 129.15, 129.02, 128.89, 127.95, 127.66, 127.29, 127.01, 126.71, 124.12, 121.58, 120.01, 119.30, 117.43, 114.66, 112.19, 107.05, 63.88, 63.48, 55.91, 43.34, 14.83, 14.63.

HRMS (ESI): *m/z* calculated for $C_{38}H_{32}N_2O_8$ 644.21532, found: 644.21512.

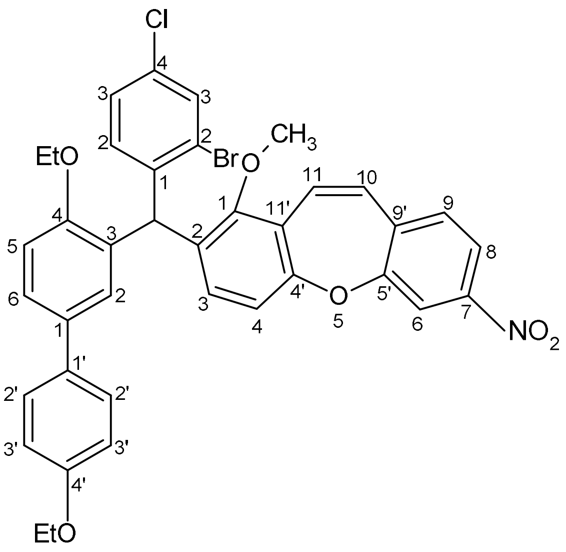

2j

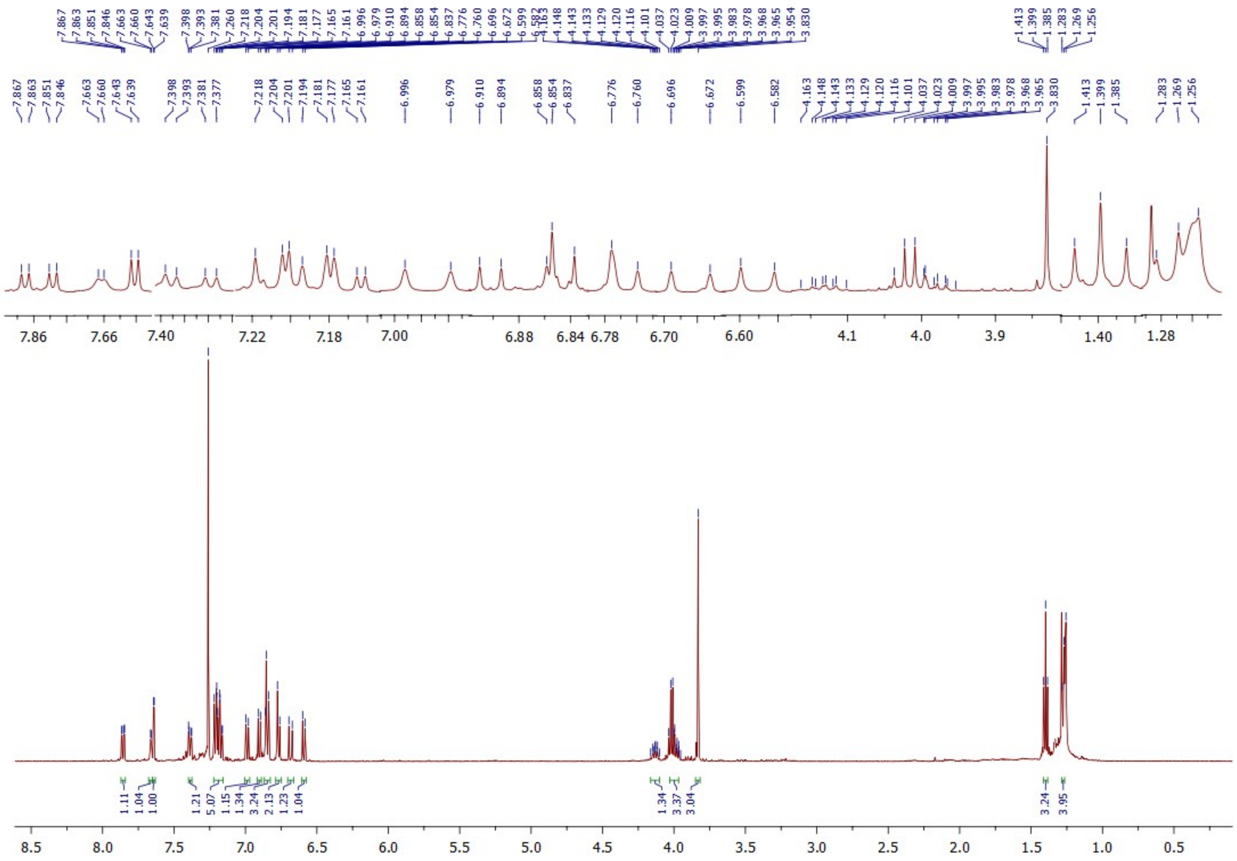

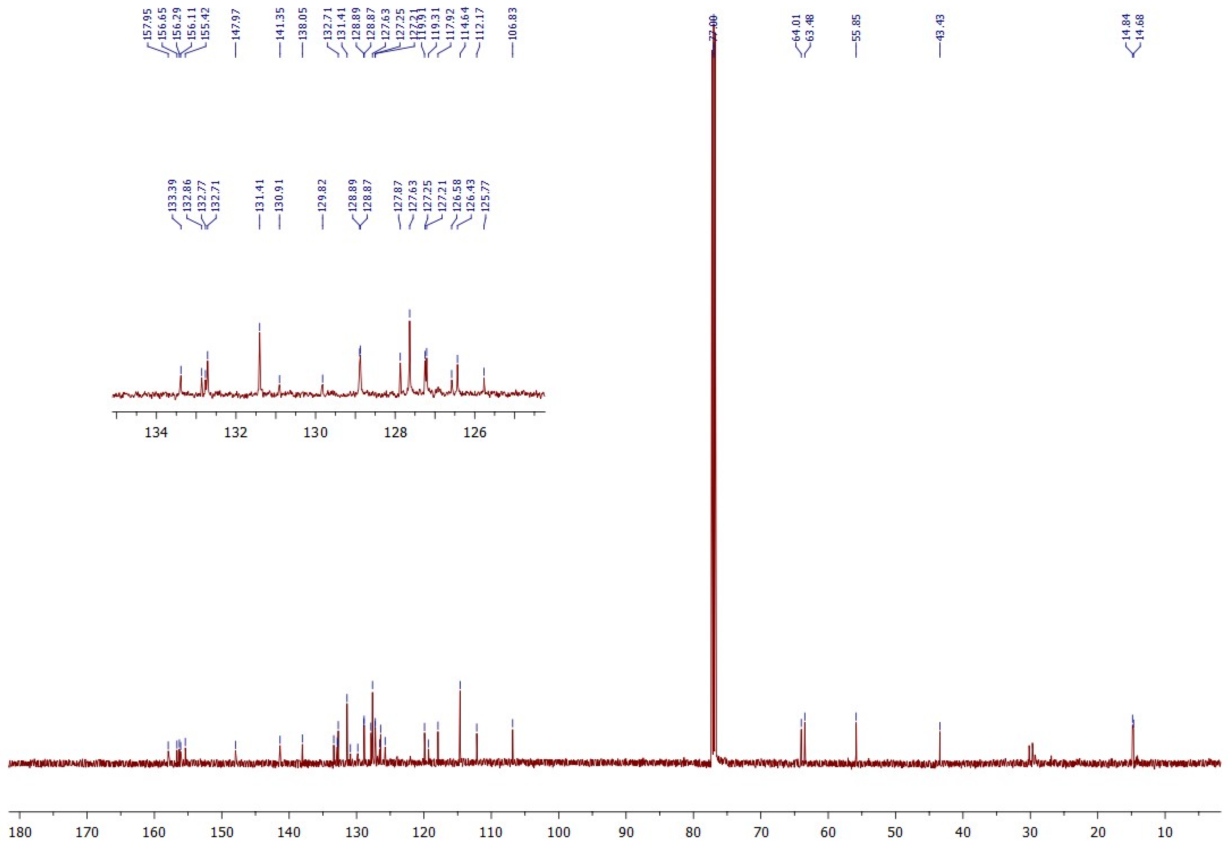

^{1}H NMR (500 MHz, $CDCl_3$, 298 K): δ (ppm): **Biphenyl rings**: 7.39 (1H, dd, $J_{H5,H6}$ = 8.5 Hz, $J_{H2,H6}$ = 2.5 Hz, H_6), 7.21 (2H, d, $J_{H2',H3'}$ = 8.5 Hz, $H_{2'}$), 6.99 (1H, d, H_5), 6.86 (1H, d, H_2), 6.85 (2H, d, $H_{3'}$), 4.13 (1H, qd, $J_{OCH2,CH3}$ = 7 Hz, $J_{OCH2,OCH2}$ = 2 Hz, OCH_2), 4.02 (2H, q, $J_{OCH2,CH3}$ = 7 Hz, OCH_2), 3.98 (1H, qd, OCH_2), 1.40 (3H, t, CH_3), 1.27 (3H, t, CH_3); **Dibenzo[*b,f*]oxepine ring**: 7.86 (1H, dd, $J_{H8,H9}$ = 8.5 Hz, $J_{H6,H8}$ = 2.5 Hz, H_8), 7.66 (1H, d, H_6), 7.19 (1H, AB spin system, d, $J_{H10,H11}$ = 11.5 Hz, H_{11}), 7.19 (1H, d, H_9), 6.77 (1H, d, $J_{H3,H4}$ = 8.5 Hz, H_4), 6.68 (1H, AB spin system, d, H_{10}), 6.59 (1H, d, H_3), 3.83 (3H, s, OCH_3). **Linker**: 7.64 (1H, d, $J_{H3,H5}$ = 2 Hz, H_3), 7.17 (1H, dd, $J_{H5,H6}$ = 8 Hz, H_5), 6.90 (1H, d, H_6), 6.78 (1H, s, CH).

^{13}C NMR (125 MHz, $CDCl_3$, 298 K): δ (ppm): 157.95, 156.65, 156.29, 156.11, 155.42, 147.97, 141.35, 138.05, 133.39, 132.86, 132.77, 132.71, 131.41, 130.91, 129.82, 128.89, 128.87, 127.87, 127.63, 127.25, 127.21, 126.58, 126.43, 125.77, 119.91, 119.31, 117.92, 114.64, 112.17, 106.83, 64.01, 63.48, 55.85, 43.43, 14.84, 14.68.

HRMS (ESI): *m/z* calculated for $C_{38}H_{31}BrClNO_6$ 711.10178, found: 711.10174.

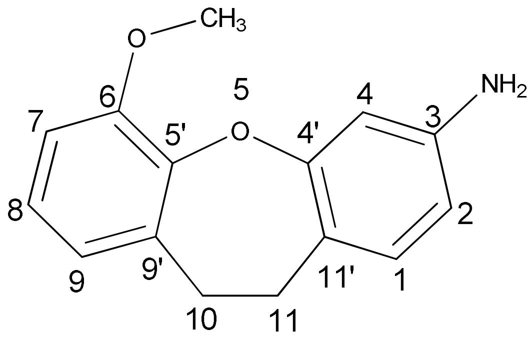

4a

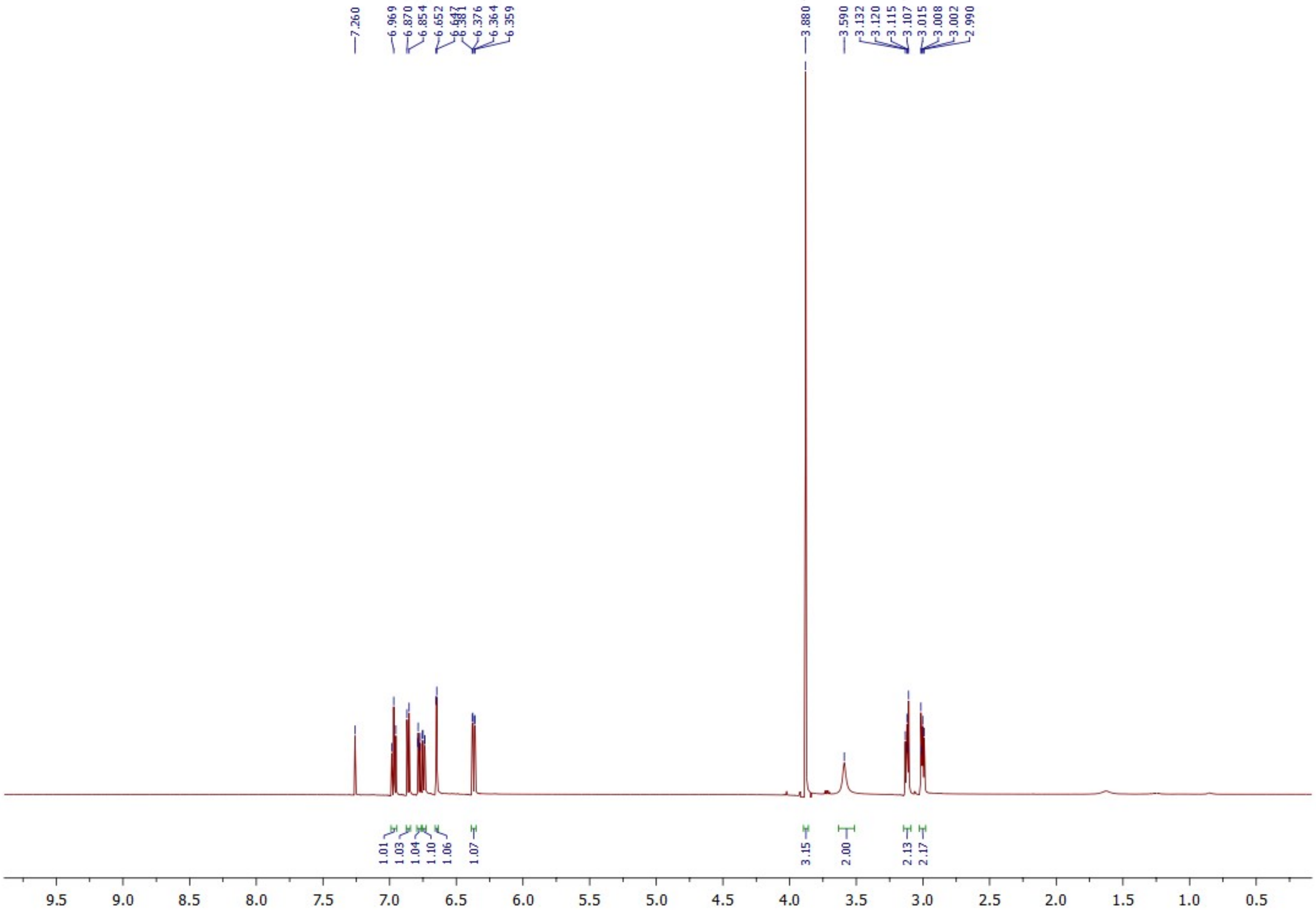
7.260
6.969
6.870
6.854
6.652
6.647
6.381
6.376
6.364
6.359
3.880
3.590
3.132
3.120
3.115
3.107
3.015
3.008
3.002
2.990
1.01
1.03
1.04
1.10
1.06
1.07
3.15
2.00
2.13
2.17
9.5
9.0
8.5
8.0
7.5
7.0
6.5
6.0
5.5
5.0
4.5
4.0
3.5
3.0
2.5
2.0
1.5
1.0
0.5

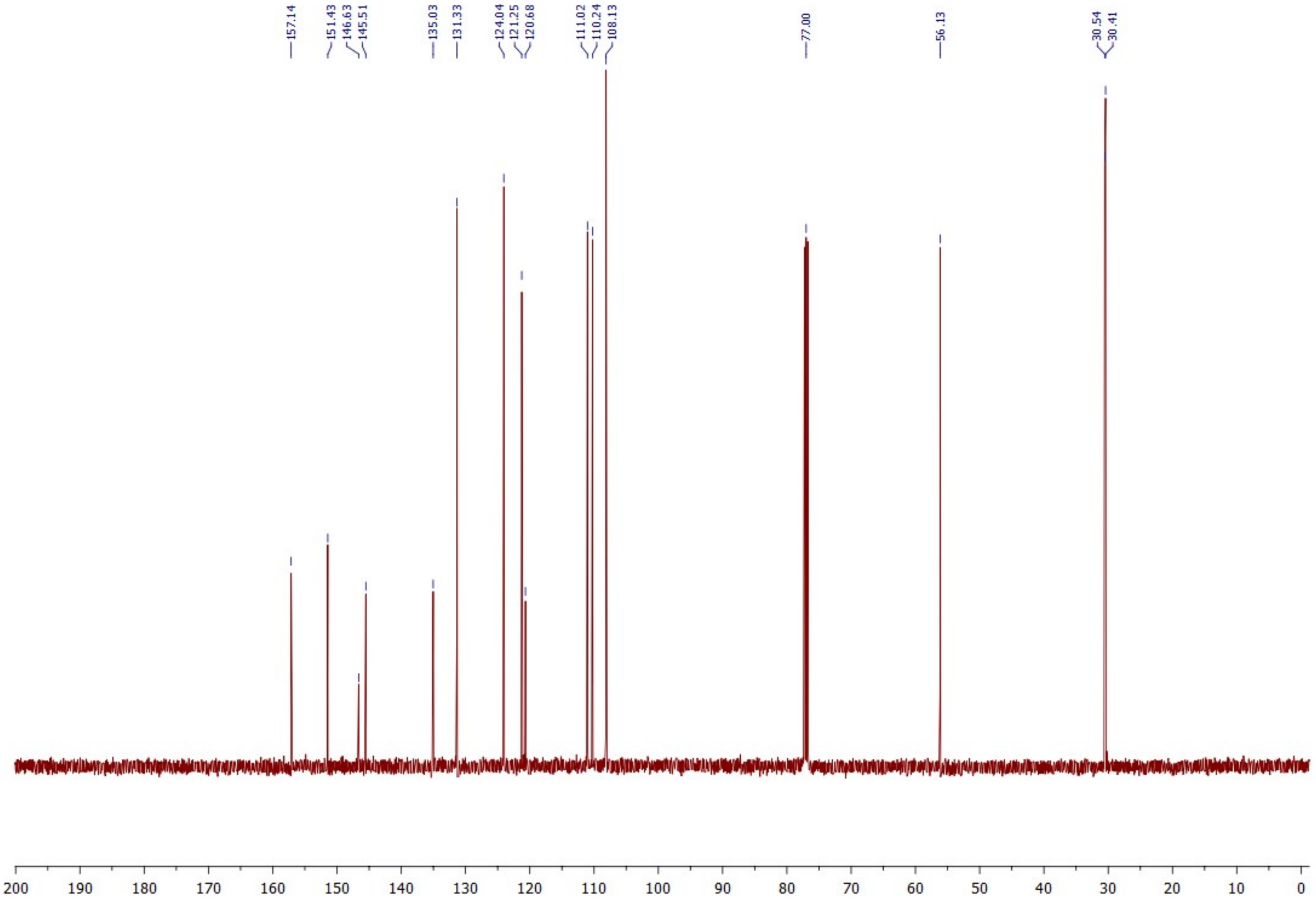

^{1}H NMR (500 MHz, $CDCl_3$, 298K): δ (ppm): 6.97 (1H, t, $J_{H8,H7.9}$ = 8.5 Hz, H_8), 6.86 (1H, d, $J_{H1,H2}$ = 8 Hz, H_1), 6.77 (1H, dd, $J_{H8,H9}$ = 1.5 Hz, H_7), 6.75 (1H, dd, H_9), 6.65 (1H, d, $J_{H2,H4}$ = 2.5 Hz, H_4), 6.37 (1H, dd, $J_{H1,H2}$ = 8 Hz, H_2), 3.88 (3H, s, OCH_3), 3.59 (2H, br. s, NH_2), 3.13-3.11 (2H, m, H_{11}), 3.02-2.99 (2H, m, H_{10}).
^{13}C NMR (125 MHz, $CDCl_3$, 298K): δ (ppm): 157.14, 151.43, 146.63, 145.51, 135.03, 131.33, 124.04, 121.25, 120.68, 111.02, 110.24, 108.13, 56.13, 30.54, 30.41.
HRMS (ESI): *m/z* calculated for $C_{15}H_{16}NO_2$ 242.11756, found: 242.11731.

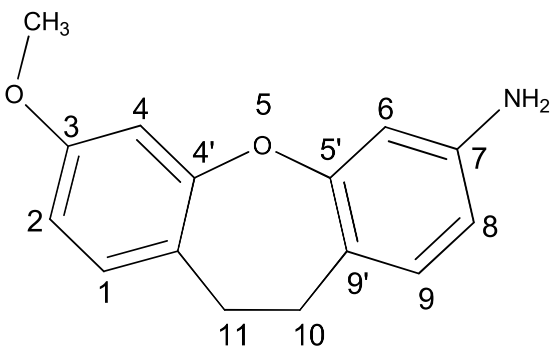

4b

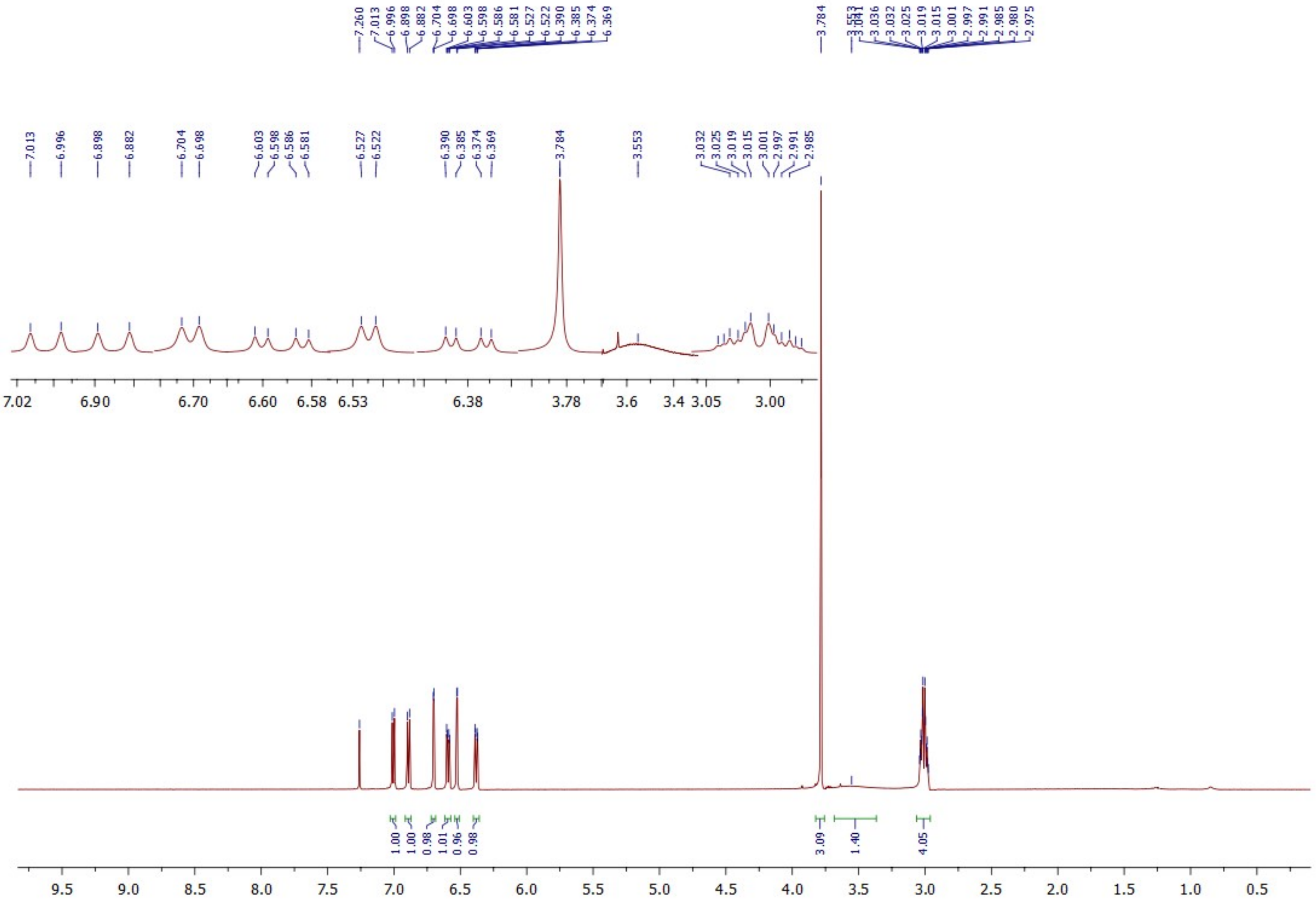
7.260
7.013
6.996
6.898
6.882
6.704
6.698
6.603
6.598
6.586
6.581
6.527
6.522
6.390
6.385
6.374
6.369
3.784
3.553
3.041
3.036
3.032
3.025
3.019
3.015
3.001
2.997
2.991
2.985
2.980
2.975
1.00
1.00
0.98
1.01
0.96
0.98
3.09
1.40
4.05
9.5
9.0
8.5
8.0
7.5
7.0
6.5
6.0
5.5
5.0
4.5
4.0
3.5
3.0
2.5
2.0
1.5
1.0
0.5
7.02
6.90
6.70
6.60
6.58
6.53
6.38
3.78
3.6
3.4
3.05
3.00

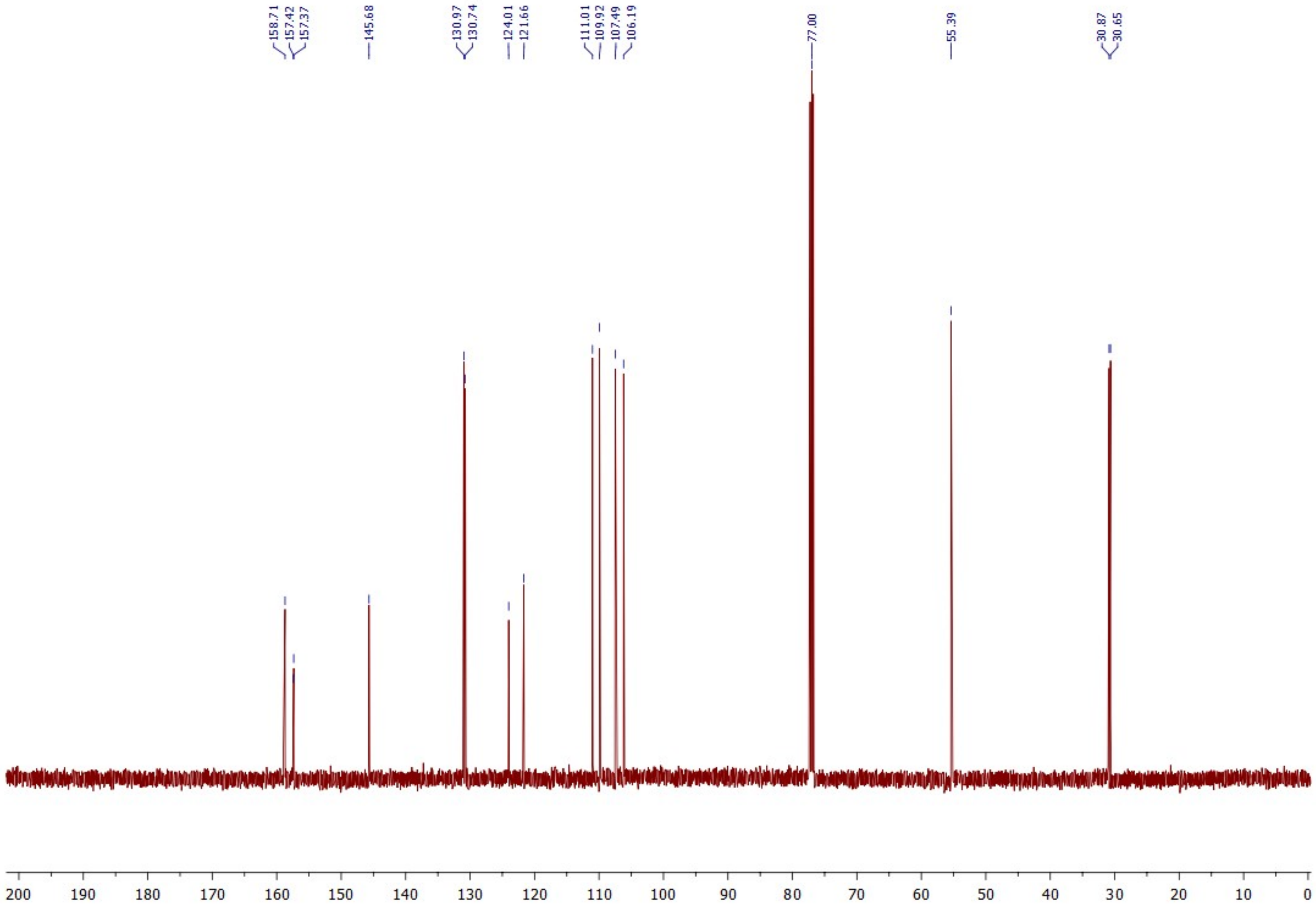

^{1}H NMR (500 MHz, $CDCl_3$, 298K): δ (ppm): 7.00 (1H, d, $J_{H1,H2}$ = 8.5 Hz, H_1), 6.89 (1H, d, $J_{H8.H9}$ = 8 Hz, H_9), 6.70 (1H, d, $J_{H2,H4}$ = 2.5 Hz, H_4), 6.59 (1H, dd, H_2), 6.52 (1H, d, $J_{H6,H8}$ = 2.5 Hz, H_6), 6.38 (1H, dd, H_8), 3.78 (3H, s, OCH_3), 3.55 (2H, br. s, NH_2), 3.04-3.01 (2H, m, H_{10}), 3.00-2.97 (2H, m, H_{11}).
^{13}C NMR (125 MHz, $CDCl_3$, 298K): δ (ppm): 158.71, 157.42, 157.37, 145.68, 130.97, 130.74, 124.01, 121.66, 111.01, 109.92, 107.49, 106.19, 55.39, 30.87, 30.65.
HRMS (ESI): *m/z* calculated for $C_{15}H_{16}NO_2$ 242.11756, found: 242.11722.

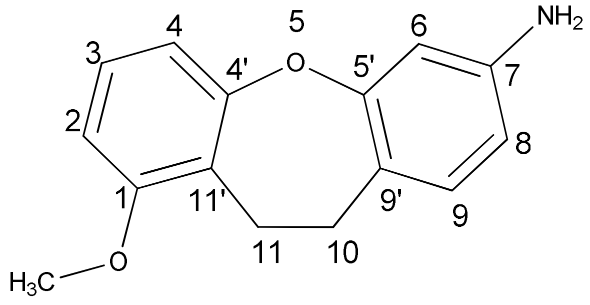

4c

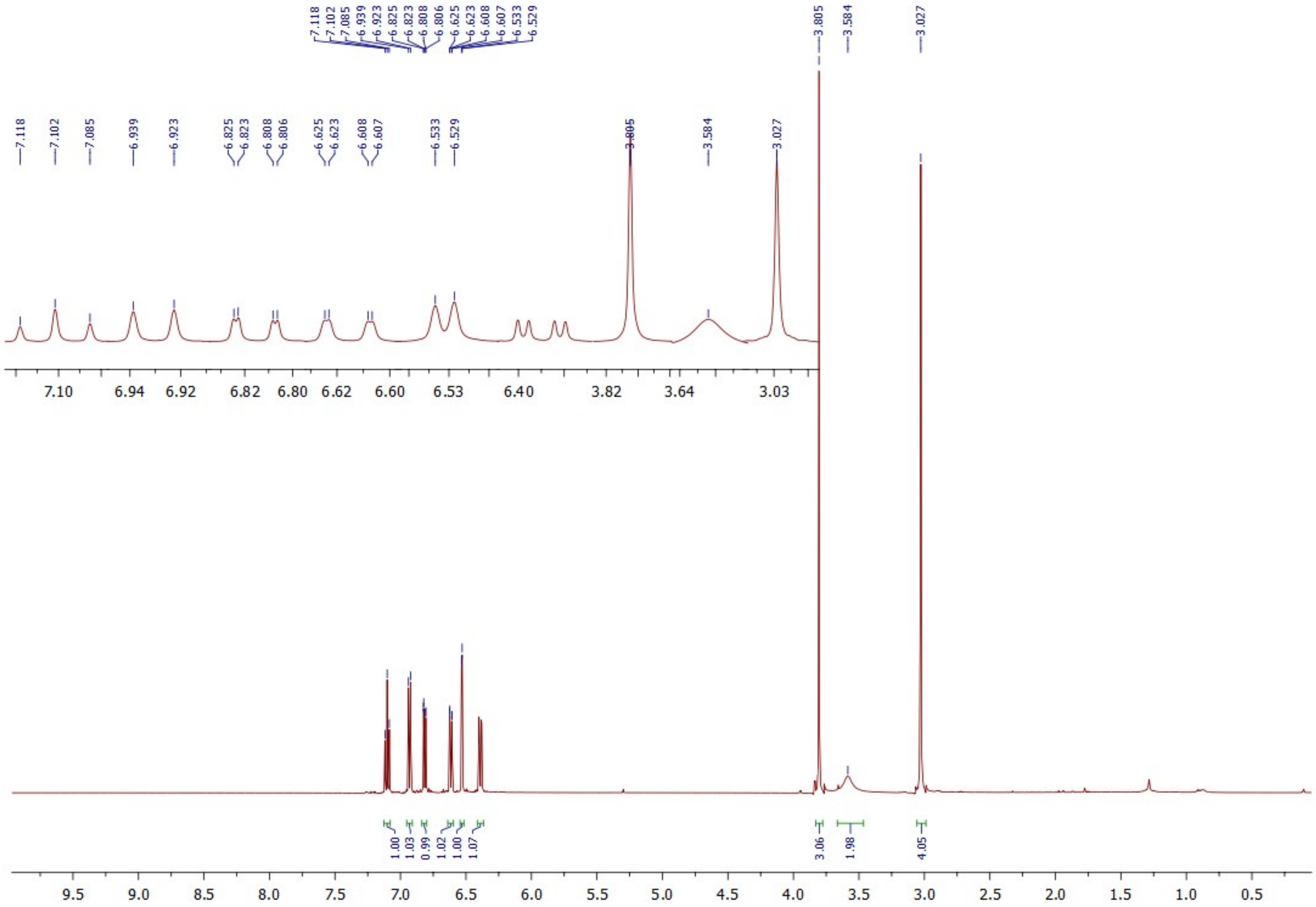

7.118
7.102
7.085
6.939
6.923
6.825
6.823
6.808
6.806
6.625
6.623
6.608
6.607
6.533
6.529
3.805
3.584
3.027
7.10 6.94 6.92 6.82 6.80 6.62 6.60 6.53 6.40 3.82 3.64 3.03
1.00 1.03 0.99 1.02 1.00 1.07 3.06 1.98 4.05
9.5 9.0 8.5 8.0 7.5 7.0 6.5 6.0 5.5 5.0 4.5 4.0 3.5 3.0 2.5 2.0 1.5 1.0 0.5

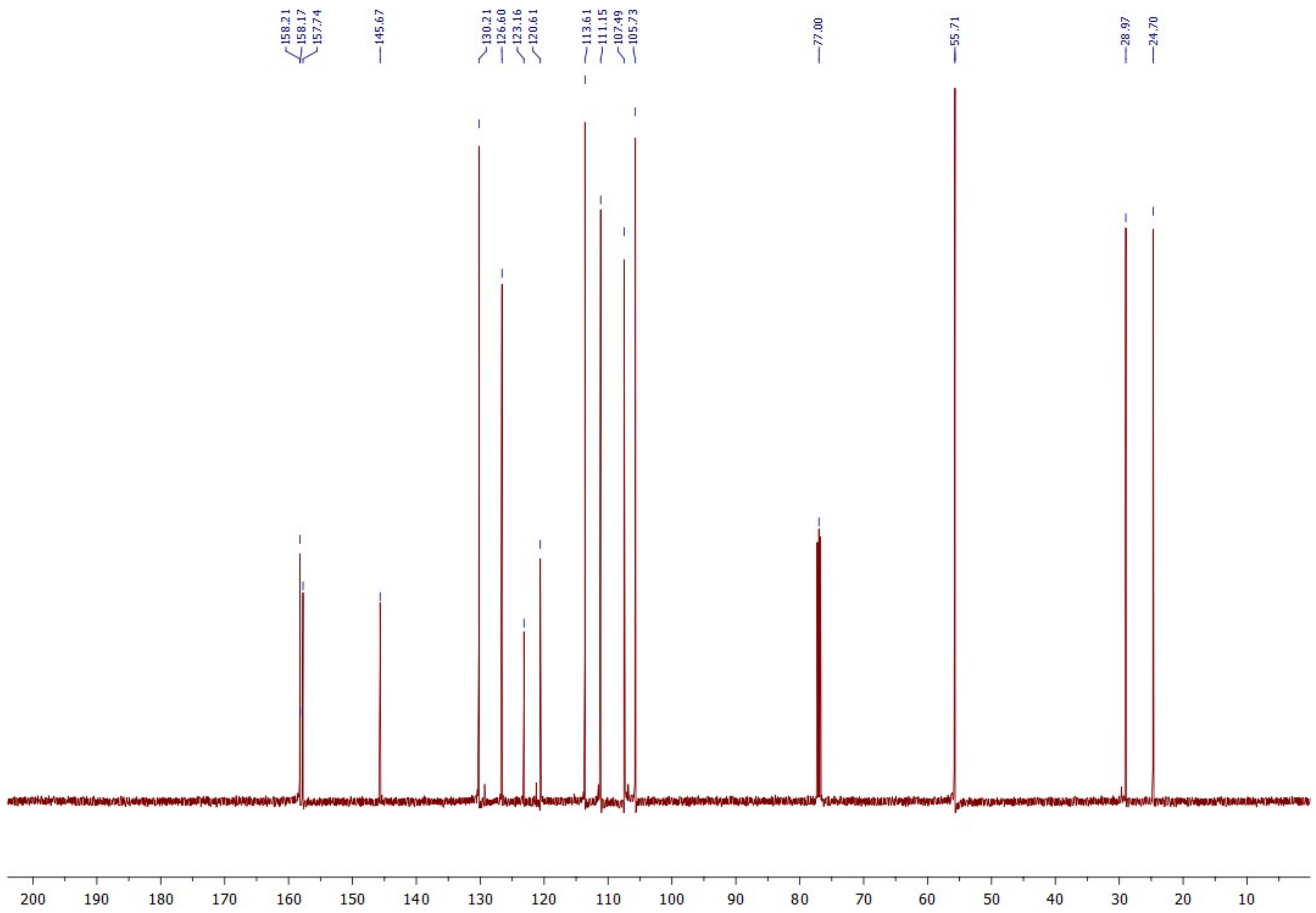

^{1}H NMR (500 MHz, $CDCl_3$, 298K): δ (ppm): 7.10 (1H, t, $J_{H3,H2,4}$ = 8.5 Hz, H_2), 6.93 (1H, d, $J_{H8,H9}$ = 8 Hz, H_9), 6.82 (1H, dd, $J_{H2,H4}$ = 1 Hz, H_2), 6.62 (1H, dd, H_4), 6.53 (1H, d, $J_{H6,H8}$ = 2.5 Hz, H_6), 6.39 (1H, dd, H_8), 3.81 (3H, s, OCH_3), 3.58 (2H, br. s, NH_2), 3.03 (4H, s, H_{10}, H_{11}).
^{13}C NMR (125 MHz, $CDCl_3$, 298K): δ (ppm): 158.21, 158.17, 157.74, 145.67, 130.21, 126.60, 123.16, 120.61, 113.61, 111.15, 107.49, 105.73, 55.71, 28.97, 24.70.
HRMS (ESI): *m/z* calculated for $C_{15}H_{16}NO_2$ 242.11756, found: 242.11734.

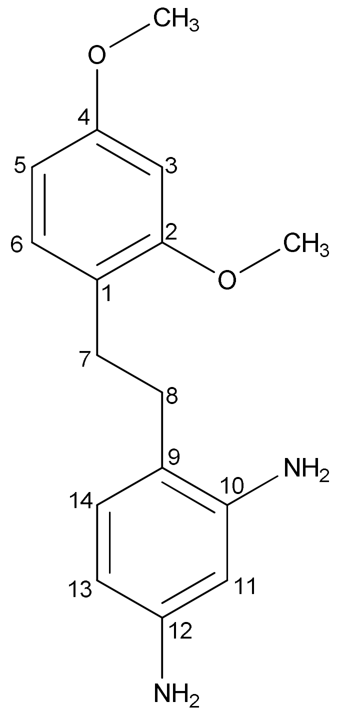

4d

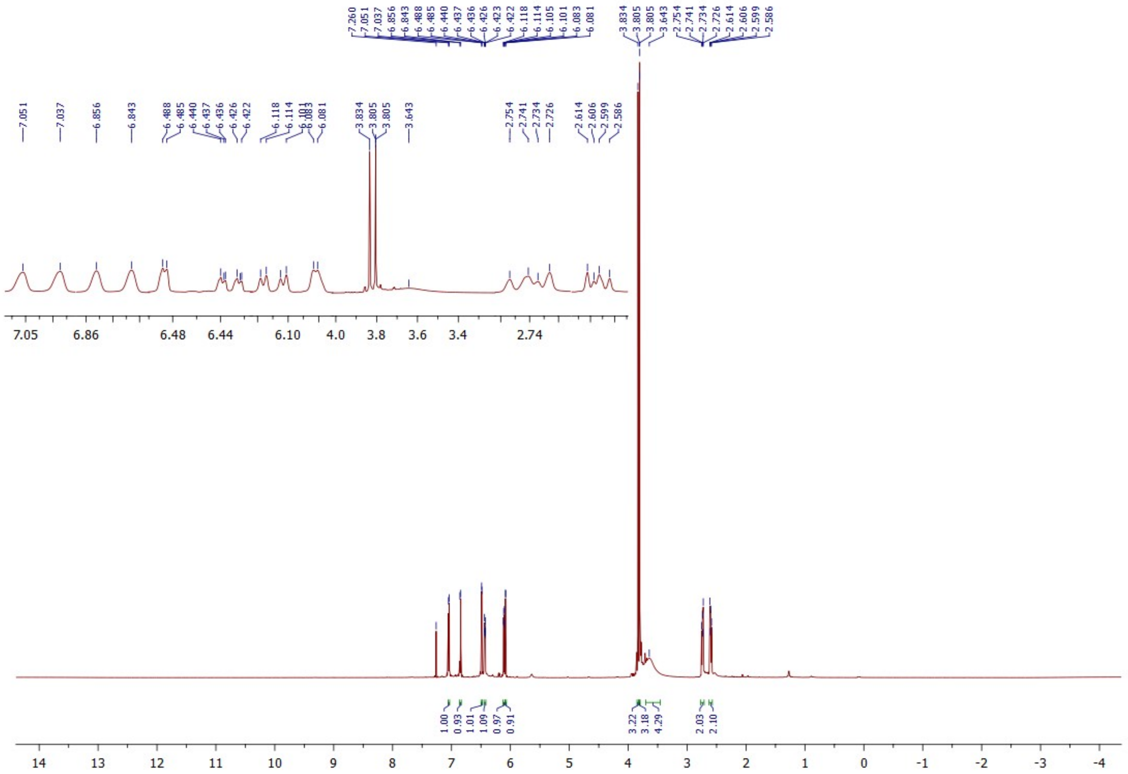
7.05
6.86
6.48
6.44
6.10
4.0
3.8
3.6
3.4
2.74
14
13
12
11
10
9
8
7
6
5
4
3
2
1
0
-1
-2
-3
-4

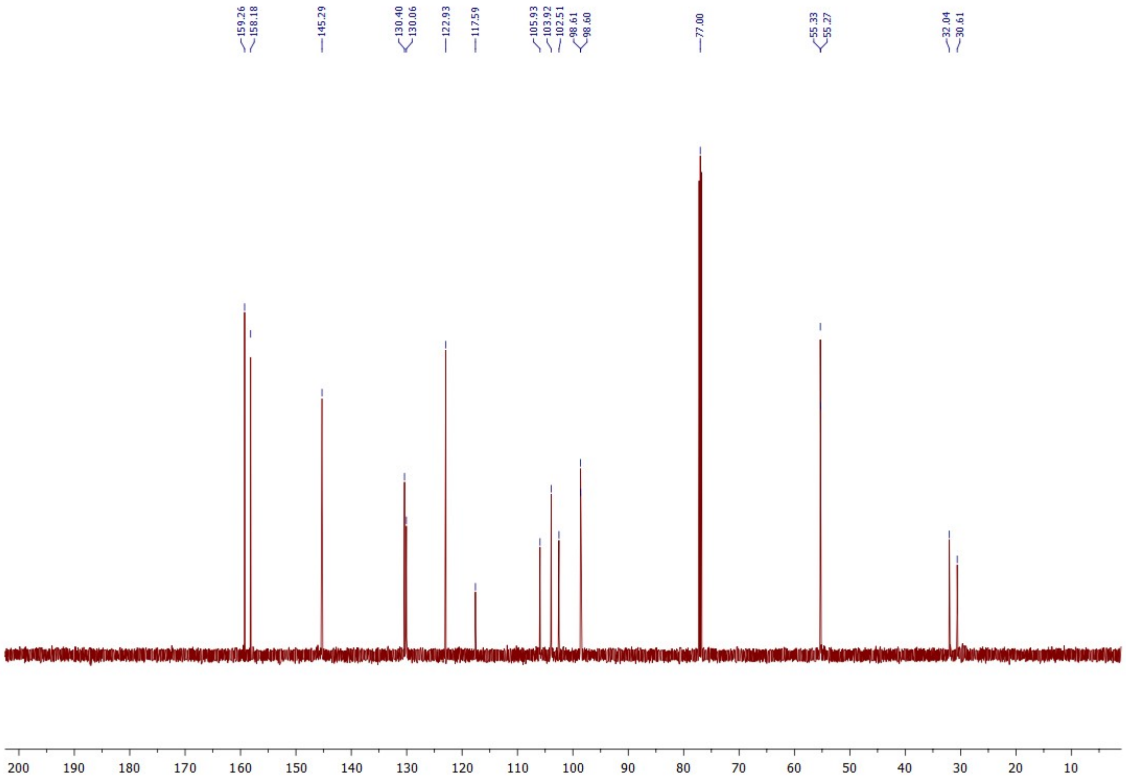

^{1}H NMR (600 MHz, $CDCl_3$, 298K): δ (ppm): 7.04 (1H, d, $J_{H5,H6}$ = 8.4 Hz, H_6), 6.85 (1H, d, $J_{H13,H14}$ = 7.8 Hz, H_{14}), 6.49 (1H, d, $J_{H3,H5}$ = 1.8 Hz, H_3), 6.44-6.42 (1H, m, H_5), 6.11 (1H, dd, $J_{H11,H13}$ = 2.4 Hz, H_{13}), 6.08 (1H, d, H_{11}), 3.83 (3H, s, OCH_3), 3.81 (3H, s, OCH_3), 3.64 (4H, br.s NH_2), 2.75-2.73 (2H, m, CH_2), 2.61-2.59 (2H, m, CH_2).

^{13}C NMR (150 MHz, $CDCl_3$, 298K): δ (ppm): 159.26, 158.18, 145.29, 130.40, 130.06, 122.93, 117.59, 105.93, 103.92, 102.51, 98.61, 98.60, 55.33, 55.27, 32.04, 30.61.

HRMS (ESI): *m/z* calculated for $C_{16}H_{21}N_2O_2$ 273.15975, found 273.15952.

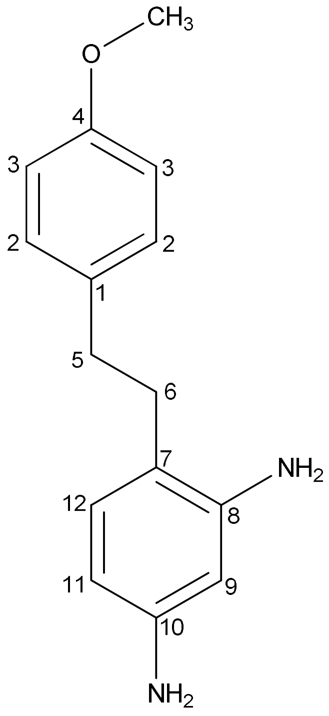

4e

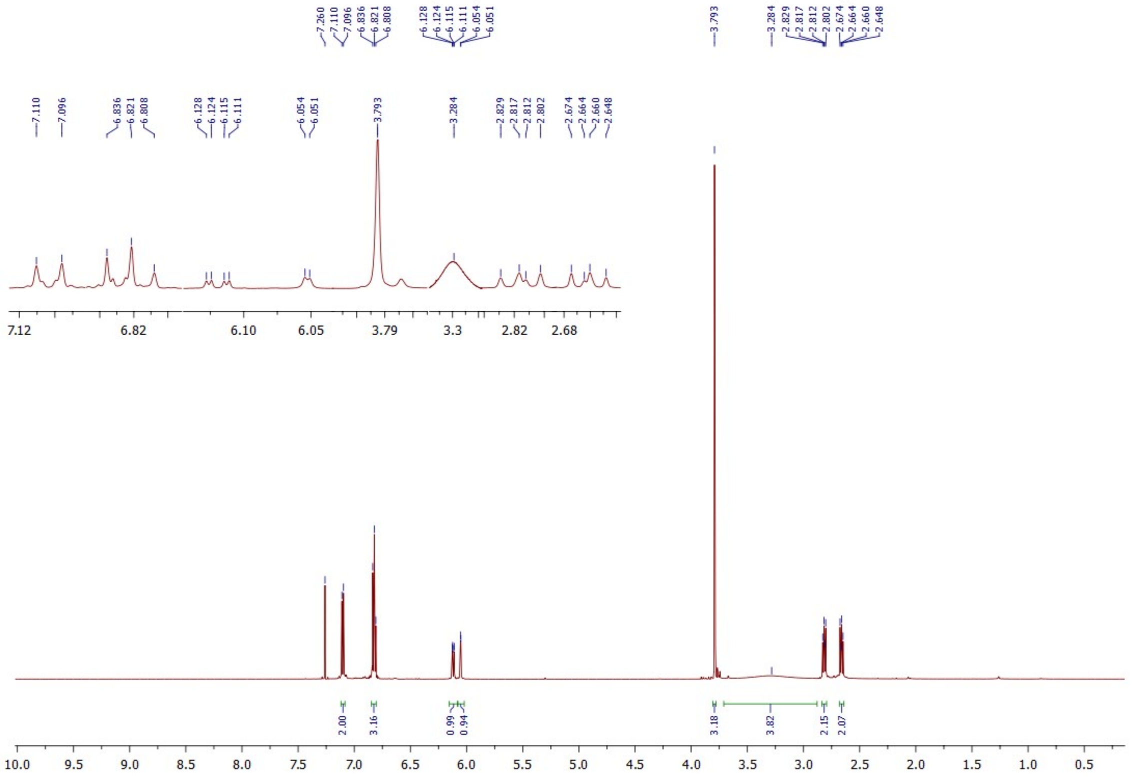
7.260
7.110
7.096
6.836
6.821
6.808
6.128
6.124
6.115
6.111
6.054
6.051
3.793
3.284
2.829
2.817
2.812
2.802
2.674
2.664
2.660
2.648
7.12
6.82
6.10
6.05
3.79
3.3
2.82
2.68
2.00
3.16
0.99
0.94
3.18
3.82
2.15
2.07
10.0
9.5
9.0
8.5
8.0
7.5
7.0
6.5
6.0
5.5
5.0
4.5
4.0
3.5
3.0
2.5
2.0
1.5
1.0
0.5

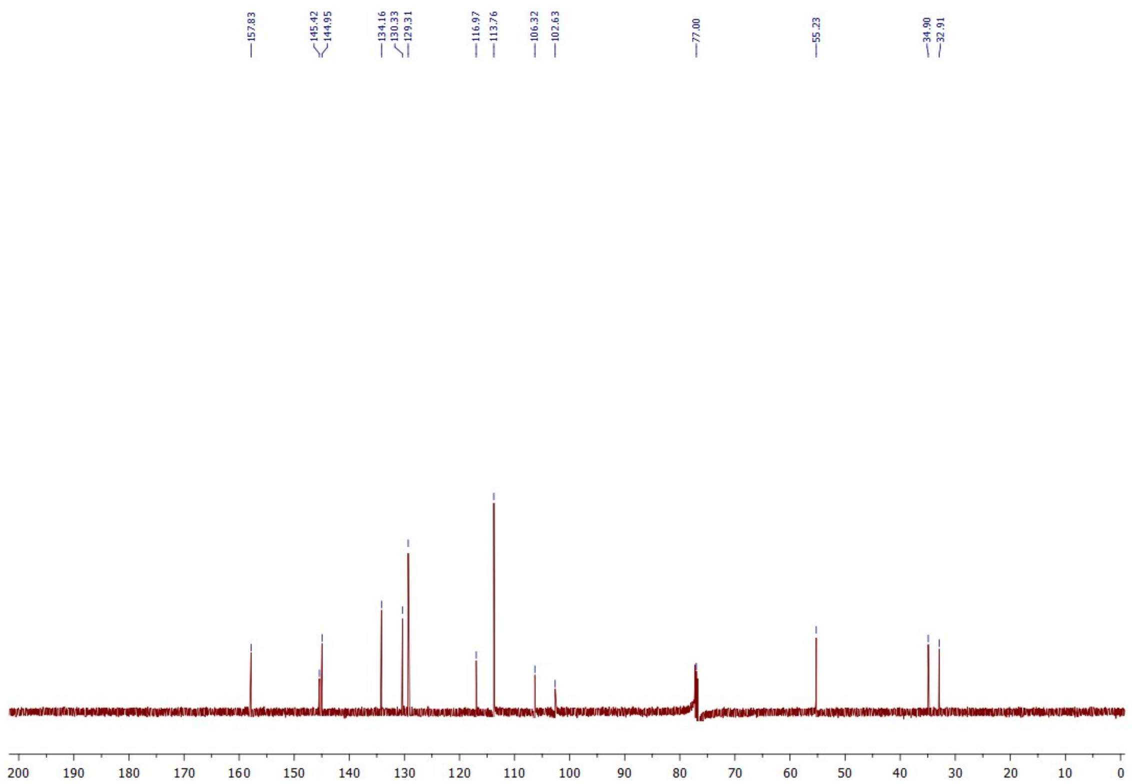

^{1}H NMR (600 MHz, $CDCl_3$, 298K): δ (ppm): 7.10 (2H, d, $J_{H2,H3}$ = 8.4 Hz, H_2), 6.83 (2H, d, H_3), 6.81 (1H, d, $J_{H11,H12}$ = 7.8 Hz, H_{12}), 6.12 (1H, dd, $J_{H9,H11}$ = 2.4 Hz, H_{11}), 6.05 (1H, d, H_9), 3.79 (3H, s, OCH_3), 3.28 (2H, br.s, NH_2), 2.83-2.80 (2H, m, CH_2), 2.67-2.65 (2H, m, CH_2).
^{13}C NMR (150 MHz, $CDCl_3$, 298K): δ (ppm): 157.83, 145.42, 144.95, 134.16, 130.33, 129.31, 116.97, 113.76, 106.32, 102.63, 55.23, 34.90, 32.91.
HRMS (ESI): *m/z* calculated for $C_{15}H_{19}N_2O$ 243.14919, found 243.14920.

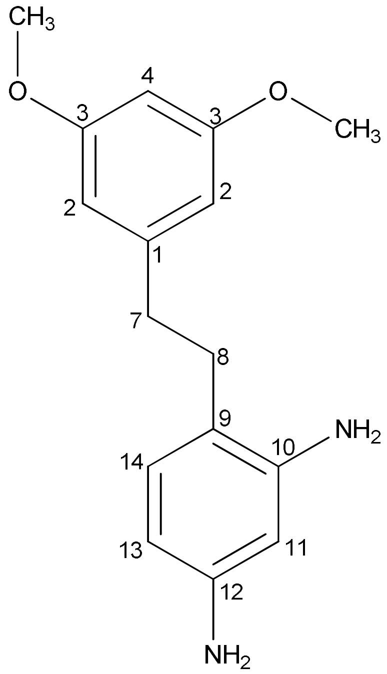

4f

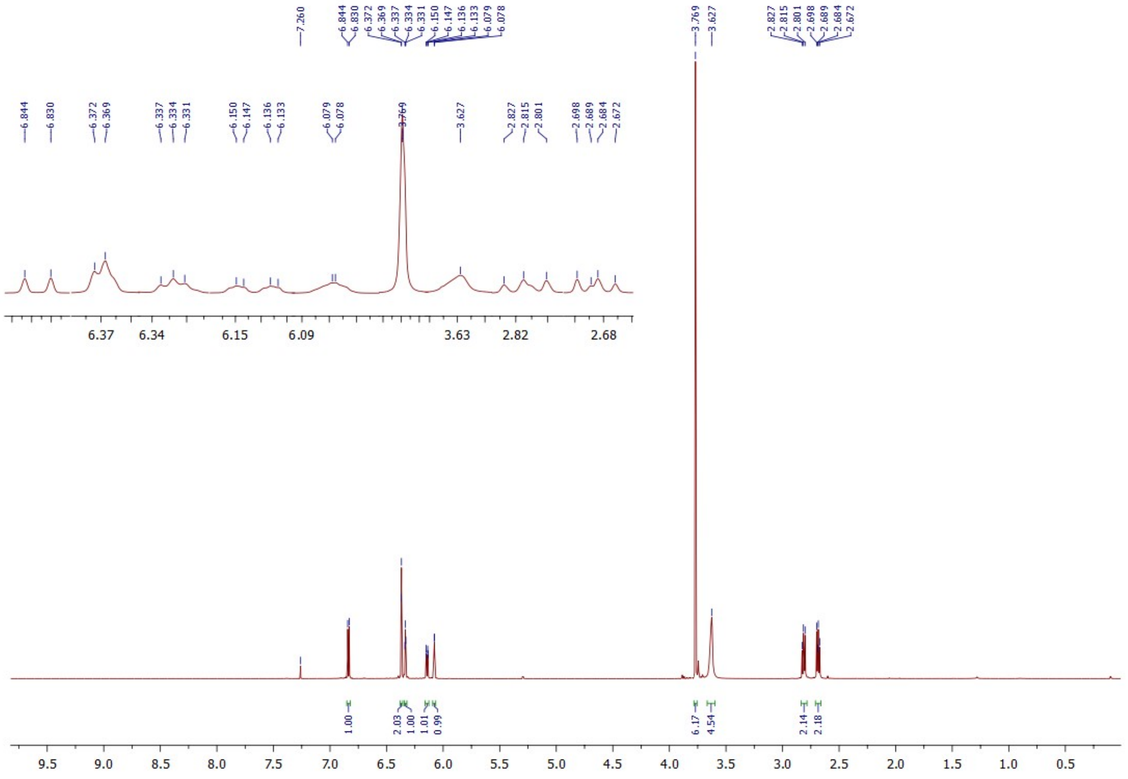
7.260
6.844
6.830
6.372
6.369
6.337
6.334
6.331
6.150
6.147
6.136
6.133
6.079
6.078
3.769
3.627
2.827
2.815
2.801
2.698
2.689
2.684
2.672
6.37
6.34
6.15
6.09
3.63
2.82
2.68
1.00
2.03
1.00
1.01
0.99
6.17
4.54
2.14
2.18
9.5
9.0
8.5
8.0
7.5
7.0
6.5
6.0
5.5
5.0
4.5
4.0
3.5
3.0
2.5
2.0
1.5
1.0
0.5

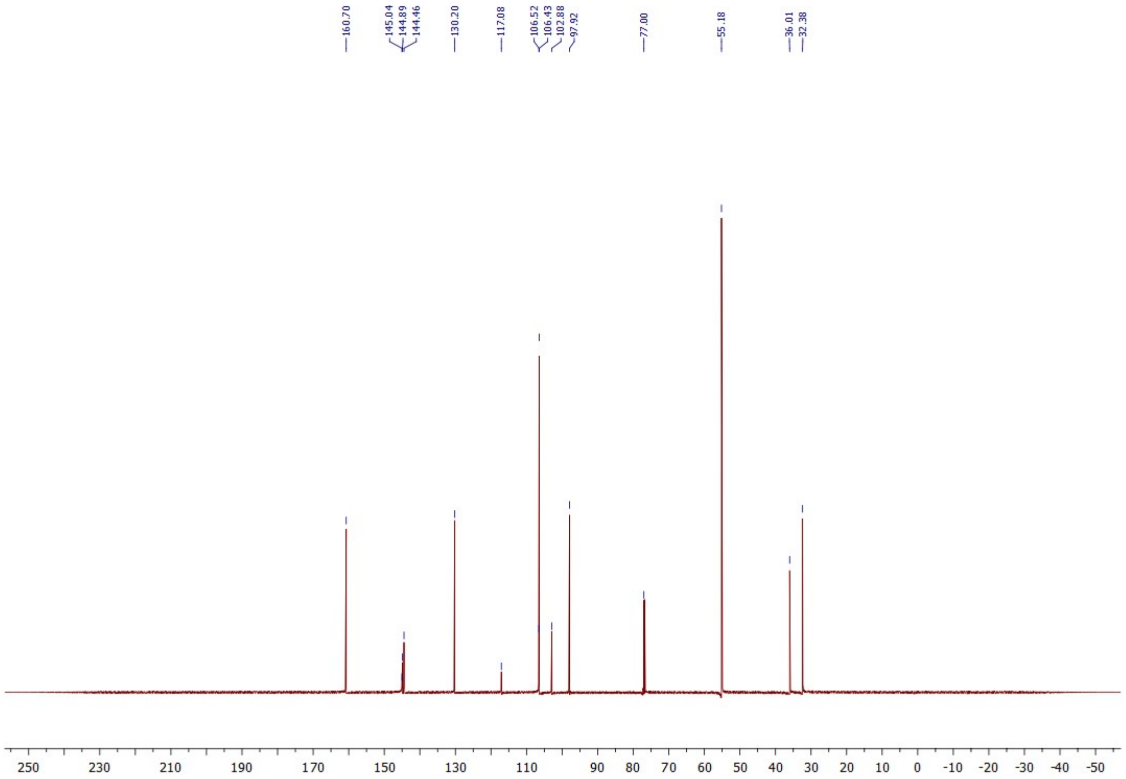

^{1}H NMR (600 MHz, $CDCl_3$, 298K): δ (ppm): 6.84 (1H, d, $J_{H13,H14}$ = 8.4 Hz, H_{14}), 6.37 (2H, d, $J_{H2,H4}$ = 1.8 Hz, H_2), 6.33 (1H, t, H_4), 6.15 (1H, dd, $J_{H11,H13}$ = 1.2 Hz, H_{13}), 6.08 (1H, d, H_{11}), 3.77 (3H, s, OCH_3), 3.63 (4H, br. s, NH_2), 2.83-2.80 (2H, m, CH_2), 2.70-2.67 (2H, m, CH_2).
^{13}C NMR (150 MHz, $CDCl_3$, 298K): δ (ppm): 160.70, 145.04, 144.89, 144.46, 130.20, 117.08, 106.52, 106.43, 102.88, 97.92, 55.18, 36.01, 32.38.
HRMS (ESI): *m/z* calculated for $C_{16}H_{21}N_2O_2$ 273.15975, found 273.15958.

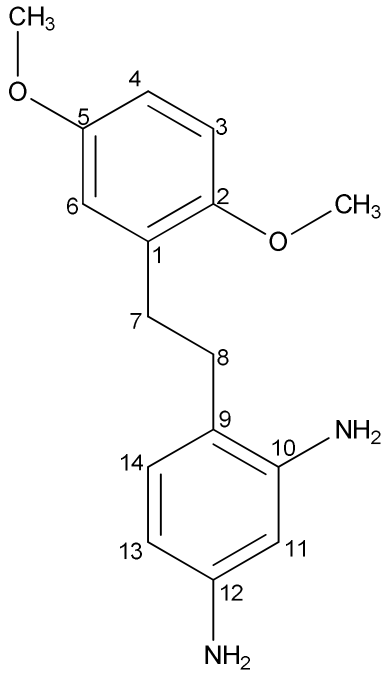

4g

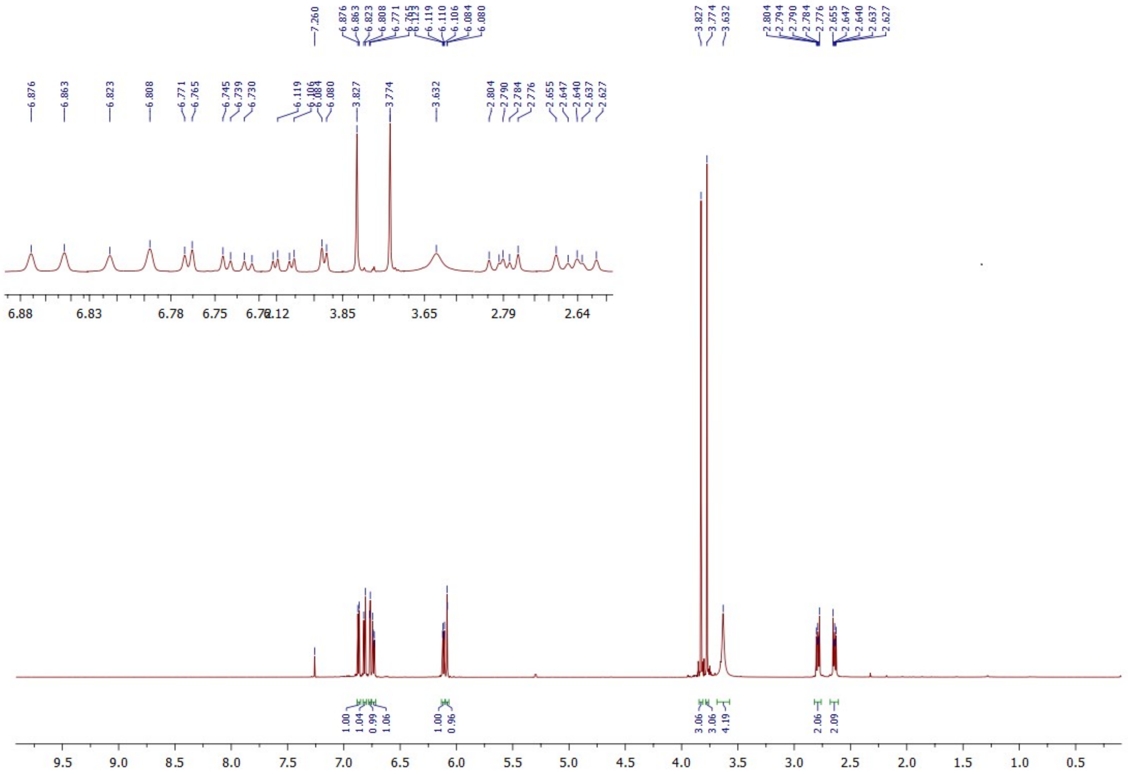
7.260
6.876
6.863
6.823
6.808
6.771
6.765
6.745
6.739
6.730
6.119
6.110
6.106
6.084
6.080
3.827
3.774
3.632
2.804
2.794
2.790
2.784
2.776
2.655
2.647
2.640
2.637
2.627
1.00
1.04
0.99
1.06
1.00
0.96
3.06
3.06
4.19
2.06
2.09
9.5
9.0
8.5
8.0
7.5
7.0
6.5
6.0
5.5
5.0
4.5
4.0
3.5
3.0
2.5
2.0
1.5
1.0
0.5

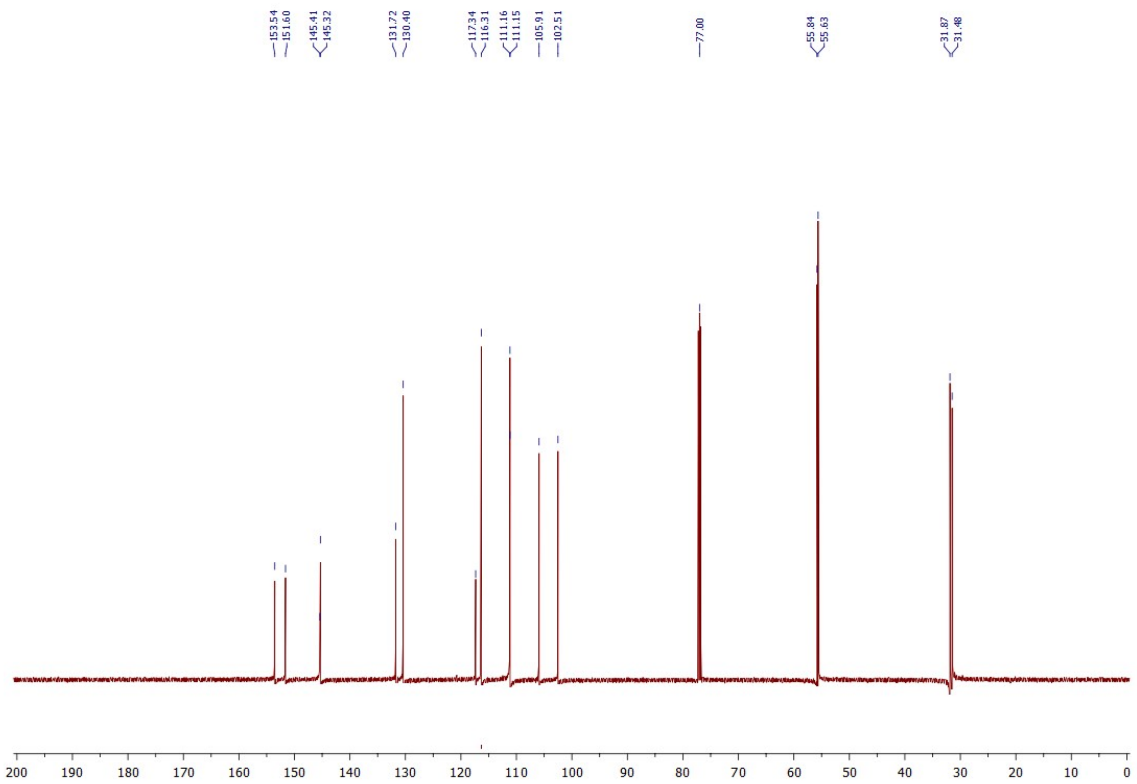

^{1}H NMR (600 MHz, $CDCl_3$, 298K): δ (ppm): 6.87 (1H, d, $J_{H13,H14}$ = 7.8 Hz, H_{14}), 6.82 (1H, d, $J_{H3,H4}$ = 9 Hz, H_3), 6.77 (1H, d, $J_{H4,H6}$ = 3.6 Hz, H_6), 6.74 (1H, dd, H_4), 6.11 (1H, dd, $J_{H11,H13}$ = 2.4 Hz, H_{13}), 6.08 (1H, d, H_{11}), 3.83 (3H, s, OCH_3), 3.77 (3H, s, OCH_3), 3.63 (4H, br.s, NH_2), 2.80-2.78 (2H, m, CH_2), 2.66-2.63 (2H, m, CH_2).
^{13}C NMR (150 MHz, $CDCl_3$, 298K): δ (ppm): 153.54, 151.60, 145.41, 145.32, 131.72, 130.40, 117.34, 116.31, 111.16, 111.15, 105.91, 102.51, 55.84, 55.63, 31.87, 31.48.
HRMS (ESI): *m/z* calculated for $C_{17}H_{23}N_2O_3$ 273.15975, found 273.15948.

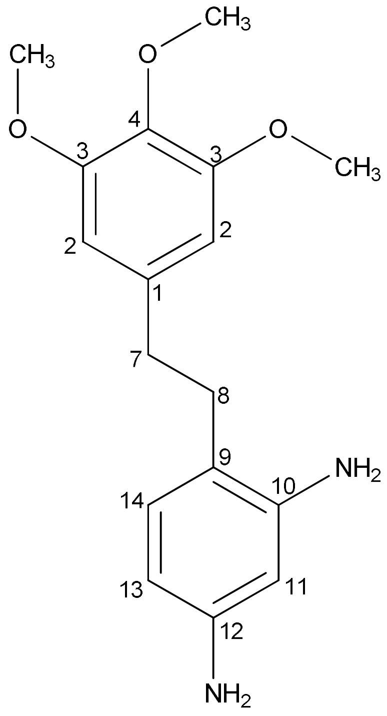

4h

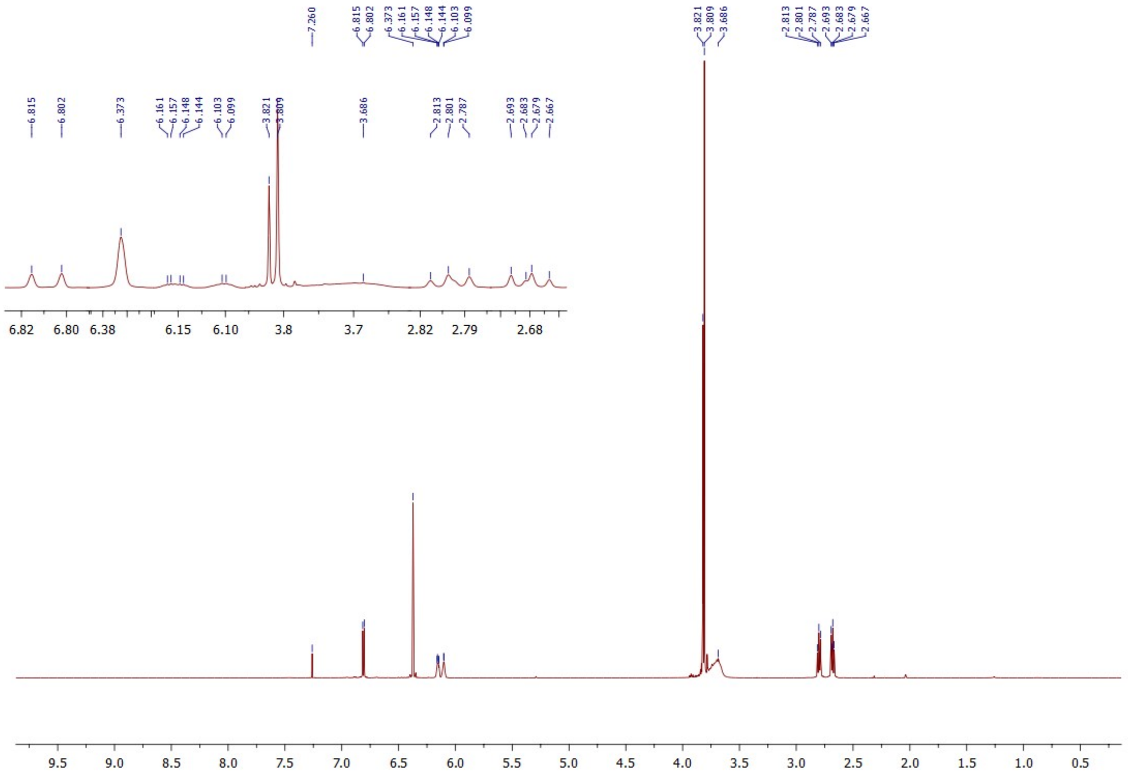
7.260
6.815
6.802
6.373
6.161
6.157
6.148
6.144
6.103
6.099
3.821
3.809
3.686
2.813
2.801
2.787
2.693
2.683
2.679
2.667
6.82 6.80 6.38 6.15 6.10 3.8 3.7 2.82 2.79 2.68
9.5 9.0 8.5 8.0 7.5 7.0 6.5 6.0 5.5 5.0 4.5 4.0 3.5 3.0 2.5 2.0 1.5 1.0 0.5

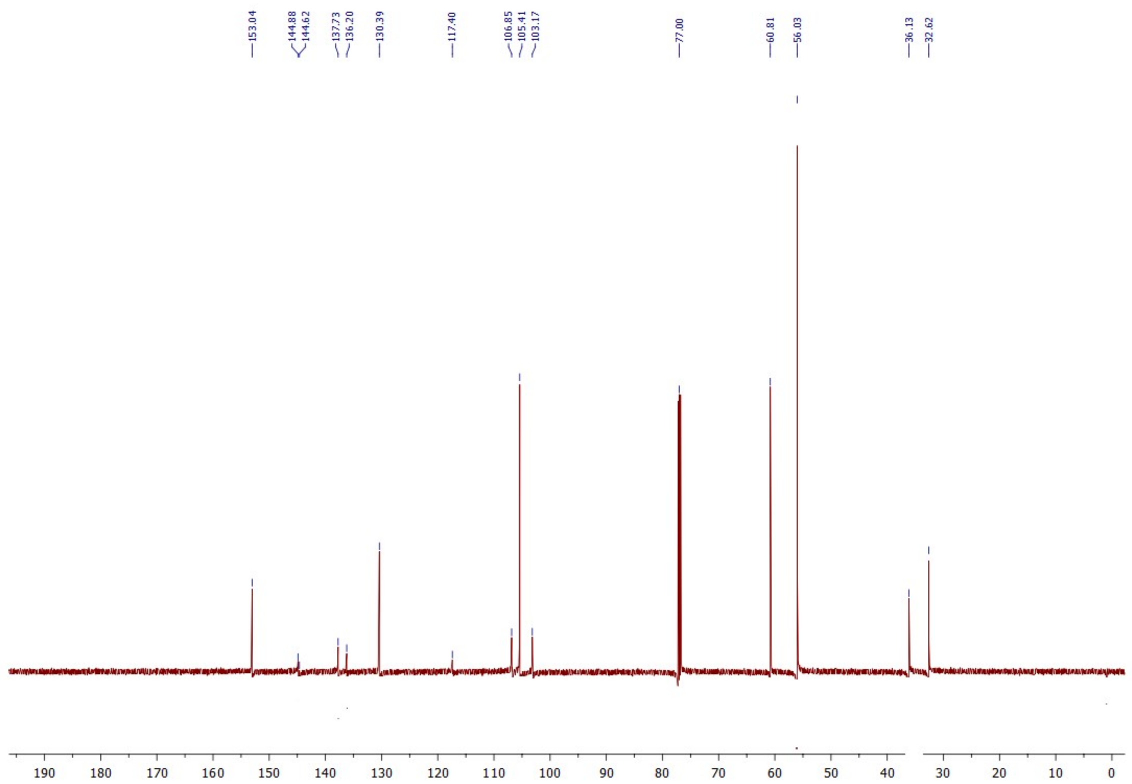

1H NMR (600 MHz, $CDCl_3$, 298K): δ (ppm): 6.81 (1H, d, $J_{H13,H14}$ = 7.8 Hz, H_{14}), 6.37 (2H, s, H_2), 6.15 (1H, dd, $J_{H11,H13}$ = 2.4 Hz, H13), 6.10 (1H, d, H_{11}), 3.82 (3H, s, OCH_3), 3.81 (6H, s, OCH_3), 3.69 (4H, br.s, NH_2), 2.81-2.79 (2H, m, CH_2), 2.69-2.67 (2H, m, CH_2).

13C NMR (150 MHz, $CDCl_3$, 298K): δ (ppm): 153.04, 144.88, 144.62, 137.73, 136.20, 130.39, 117.40, 106.85, 105.41, 103.17, 77.00, 60.81, 56.03, 36.13, 32.62.

HRMS (ESI): *m/z* calculated for $C_{17}H_{23}N_2O_3$ 303.17032, found 303.17020.

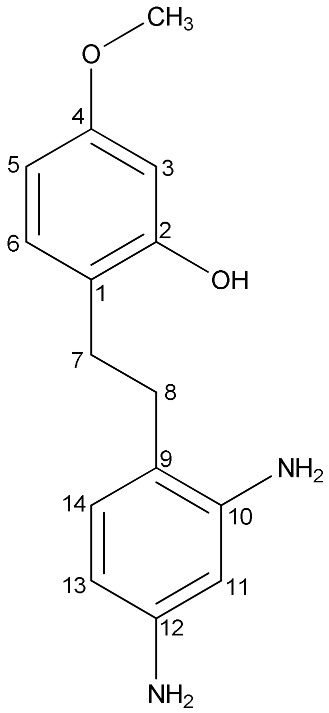

4i

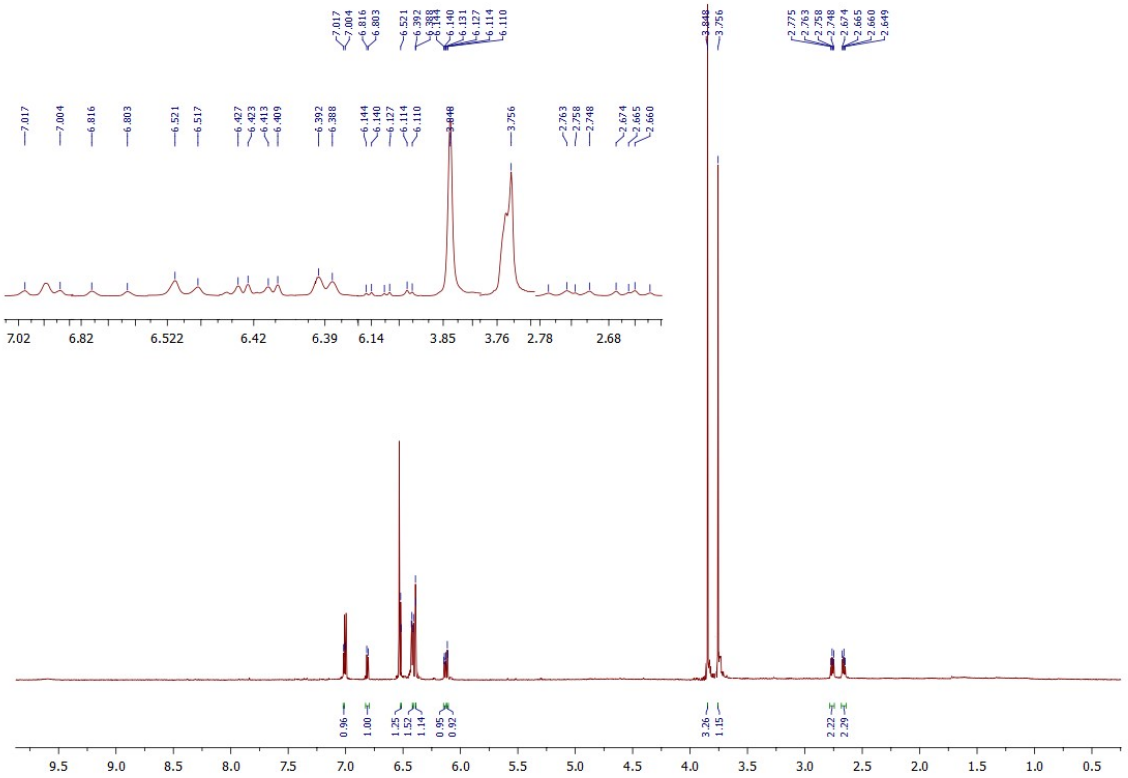

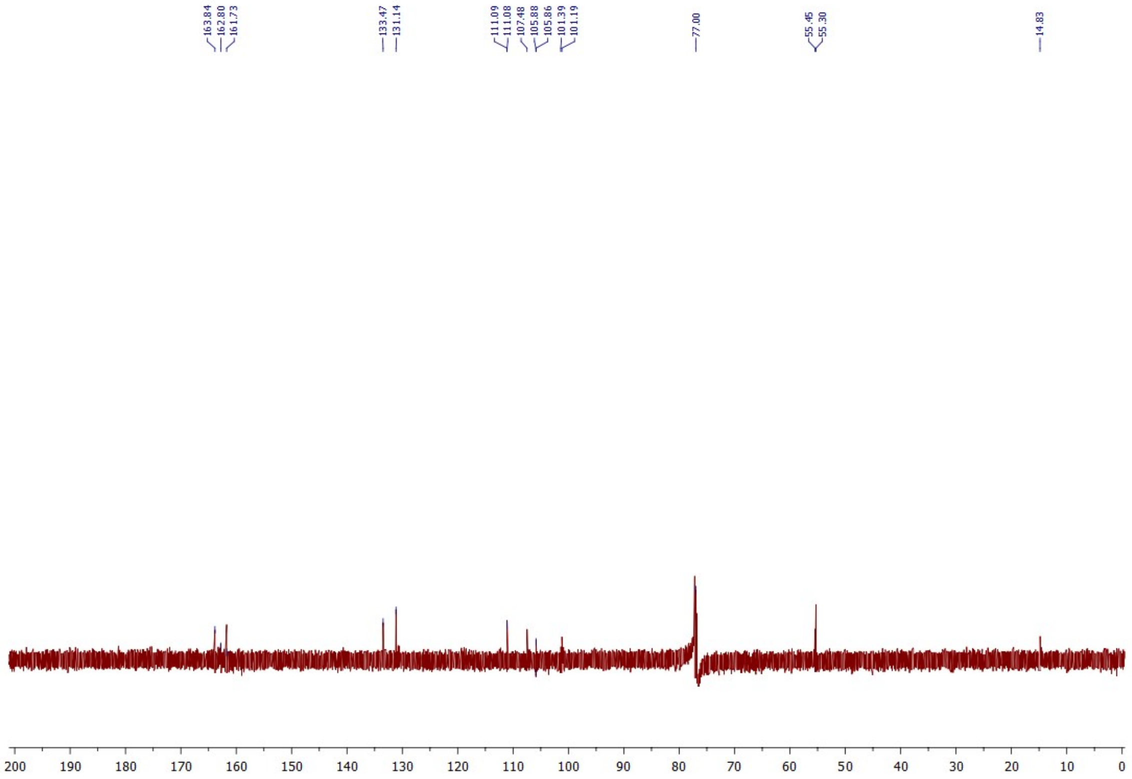

^{1}H NMR (600 MHz, $CDCl_3$, 298K): δ (ppm): 7.01 (1H, d, $J_{H5,H6}$ = 7.8 Hz, H_6), 6.81 (1H, $J_{H13,H14}$ = 7.8 Hz, H_{14}), 6.52 (1H, d, $J_{H3,H5}$ = 2.4 Hz, H_3), 6.42 (1H, dd, H_5), 6.14 (1H, dd, $J_{H11,H13}$ = 2.4 Hz, H_{13}), 6.11 (1H, d, H_{11}), 3.85 (3H, s, OCH_3), 3.76 (3H, s, OCH_3), 2.78-2.75 (2H, m, CH_2), 2.67-2.65 (2H, m, CH_2).
^{13}C NMR (150 MHz, $CDCl_3$, 298K): δ (ppm): 163.84, 162.80, 161.73, 133.47, 131.14, 111.09, 111.08, 107.48, 105.88, 105.86, 101.39, 101.19, 55.45, 55.30, 14.83.
HRMS (ESI): *m/z* calculated for $C_{15}H_{19}N_2O_2$ 259.14410, found 259.14403.

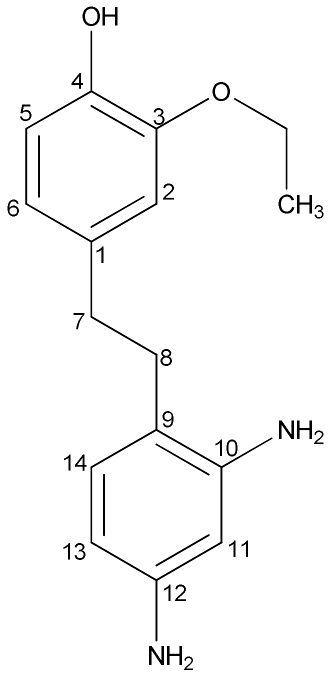

4j

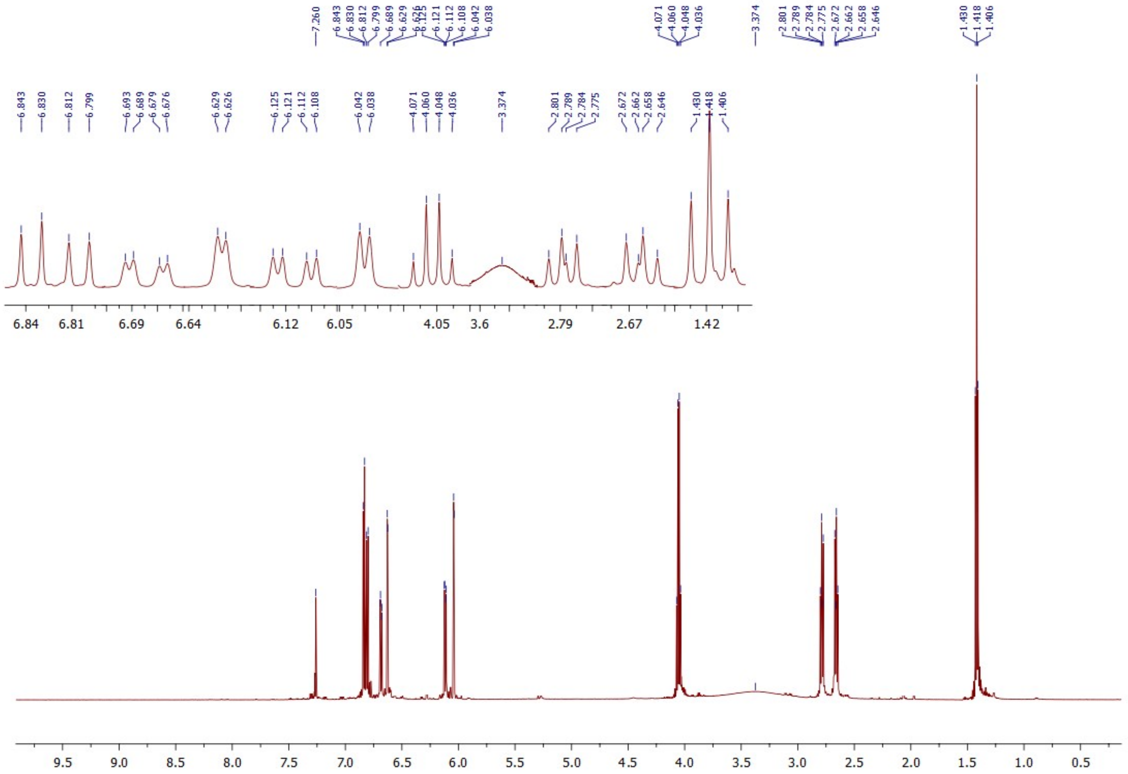
7.260
6.843
6.830
6.812
6.799
6.693
6.689
6.679
6.676
6.629
6.626
6.125
6.121
6.112
6.108
6.042
6.038
4.071
4.060
4.048
4.036
3.374
2.801
2.789
2.784
2.775
2.672
2.662
2.658
2.646
1.430
1.418
1.406
6.84
6.81
6.69
6.64
6.12
6.05
4.05
3.6
2.79
2.67
1.42
9.5
9.0
8.5
8.0
7.5
7.0
6.5
6.0
5.5
5.0
4.5
4.0
3.5
3.0
2.5
2.0
1.5
1.0
0.5

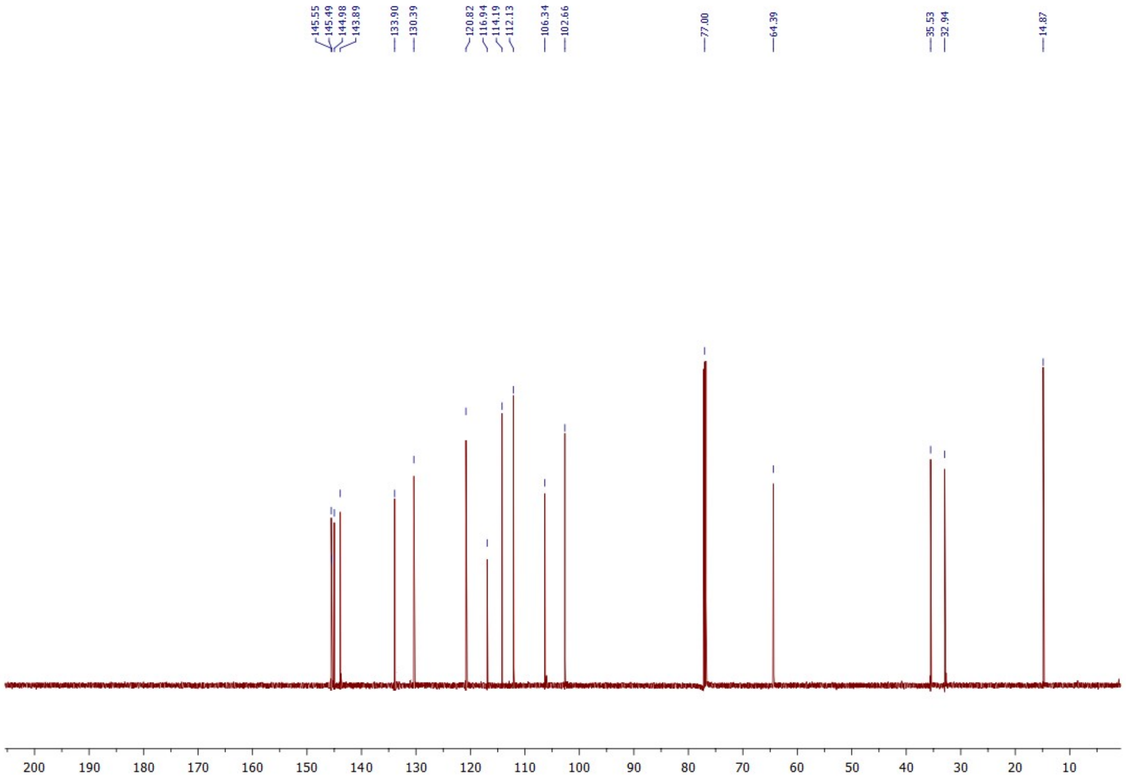

^{1}H NMR (600 MHz, $CDCl_3$, 298K): δ (ppm): 6.84 (1H, d, $J_{H5,H6}$ = 7.8 Hz, H_5), 6.81 (1H, d, $J_{H13,H14}$ = 7.8 Hz, H_{14}), 6.68 (1H, dd, $J_{H2,H6}$ = 1.8 Hz, H_6), 6.63 (1H, d, H_2), 6.12 (1H, dd, $J_{H11,H12}$ = 2.4 Hz, H_{13}), 6.04 (1H, d, H_{11}), 4.05 (2H, q, $J_{CH2,CH3}$ = 7.2 Hz, CH_2), 3.37 (4H, br.s, NH_2), 2.80-2.78 (2H, m, CH_2), 2.67-2.65 (2H, m, CH_2), 1.42 (3H, t, CH_3).
^{13}C NMR (150 MHz, $CDCl_3$, 298K): δ (ppm): 145.55, 145.49, 144.98, 143.89, 133.90, 130.39, 120.82, 116.94, 114.19, 112.13, 106.34, 102.66, 64.39, 35.53, 32.94, 14.87.
HRMS (ESI): *m/z* calculated for $C_{16}H_{21}N_2O_2$ 273.15975, found 273.15933.

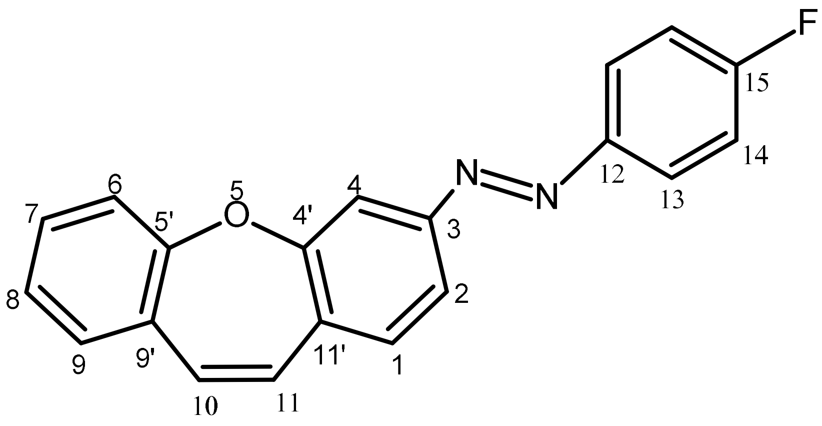

7a

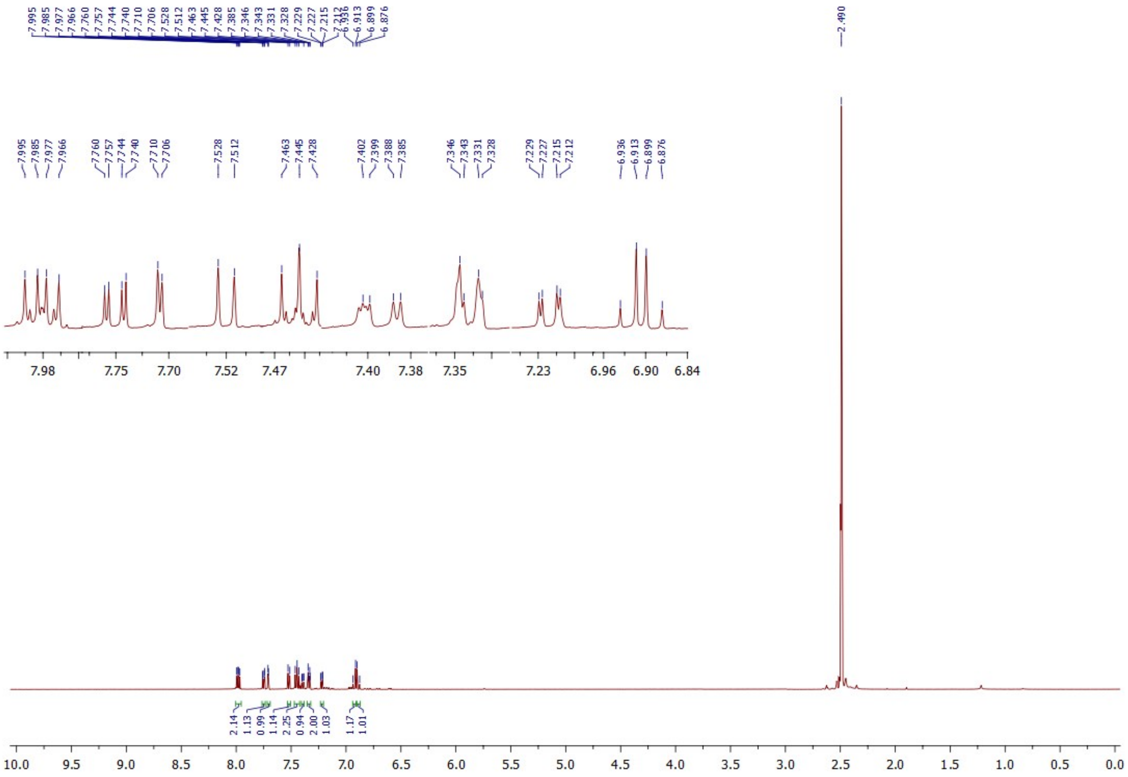

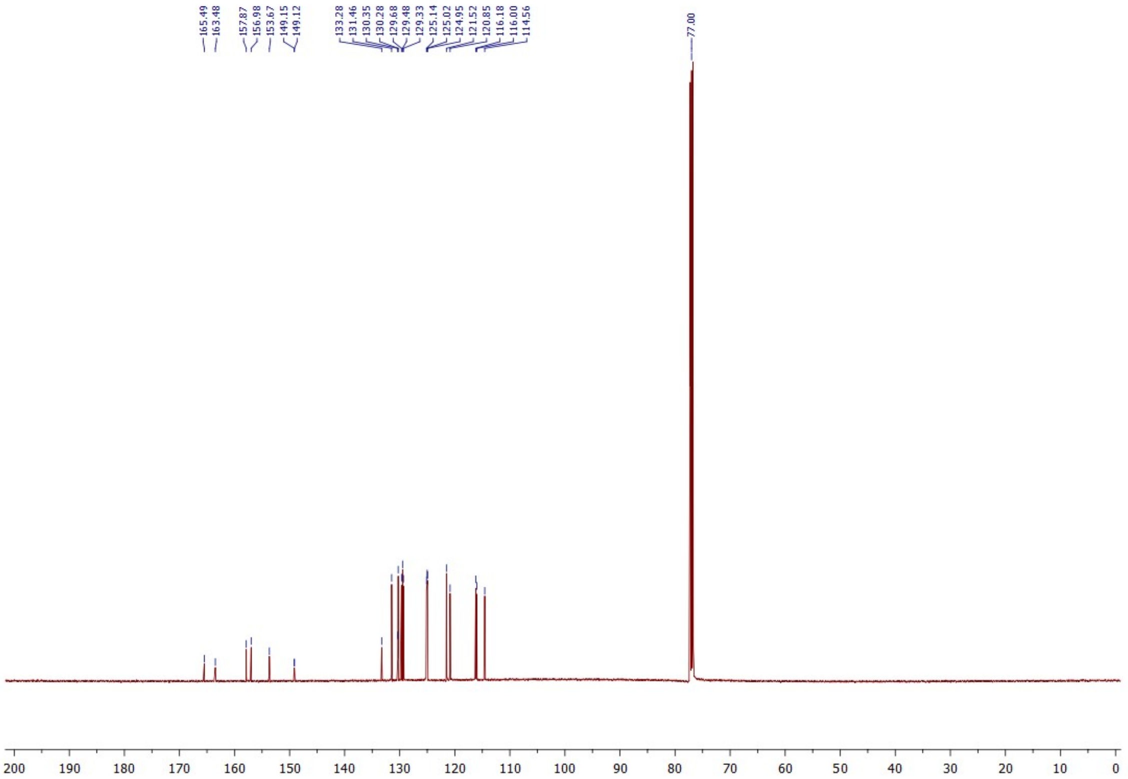

^{1}H NMR (500 MHz, DMSO-d$_6$, 298K): δ (ppm): 7.98 (2H, dd, $J_{H13,H14}$ = 9 Hz, $J_{H13,F}$ = 5.5 Hz, H_{13}), 7.75 (1H, dd, $J_{H1,H2}$ = 8.5 Hz, $J_{H2,H4}$ = 2 Hz, H_2), 7.71 (1H, d, $J_{H2,H4}$ = 2 Hz, H_4), 7.52 (1H, d, H_1), 7.45 (2H, t, $J_{H14,F}$ = 9 Hz, H_{14}), 7.39 (1H, dd, $J_{H6,H7}$ = 7 Hz, $J_{H6,H8}$ = 1.5 Hz, H_6), 7.35-7.33 (2H, m, H_7, H_8), 7.22 (1H, dd, $J_{H8,H9}$ = 7 Hz, $J_{H7,H9}$ = 1 Hz, H_9), 6.92 (1H, AB spin system, d, $J_{H10,H11}$ = 11.5 Hz, H_{10}), 6.89 (1H, AB spin system, d, H_{11}).
^{13}C NMR (125 MHz, CDCl$_3$, 298K): δ (ppm): 164.44 (d, $J_{C15,F}$ = 251.25 Hz, C15), 157.87 (C4'), 156.98 (C5'), 153.67 (C3), 149.13 (d, $J_{C12,F}$ = 3 Hz, C12), 133.28 (C11'), 131.46 (C10), 130.35 (C9'), 130.28 (C7), 129.68 (C1), 129.48 (C9), 129.33 (C11), 125.14 (C8), 124.98 (d, $J_{C13,F}$ = 8.875 Hz, C13), 121.52 (C6), 120.85 (C2), 116.09 (d, $J_{C14,F}$ = 22.75 Hz, C14), 114.56 (C4).
HRMS (ESI): *m/z* calculated for $C_{20}H_{13}FN_2O$+H 317.10847, found: 317.10829.

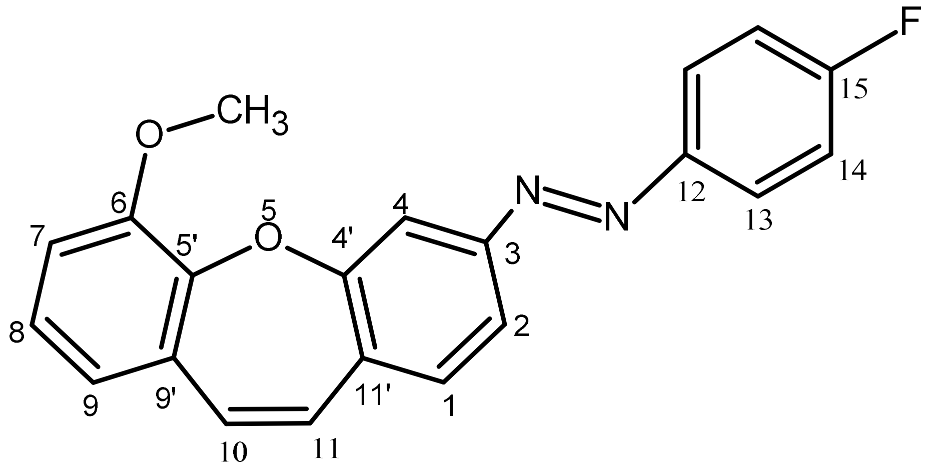

7b

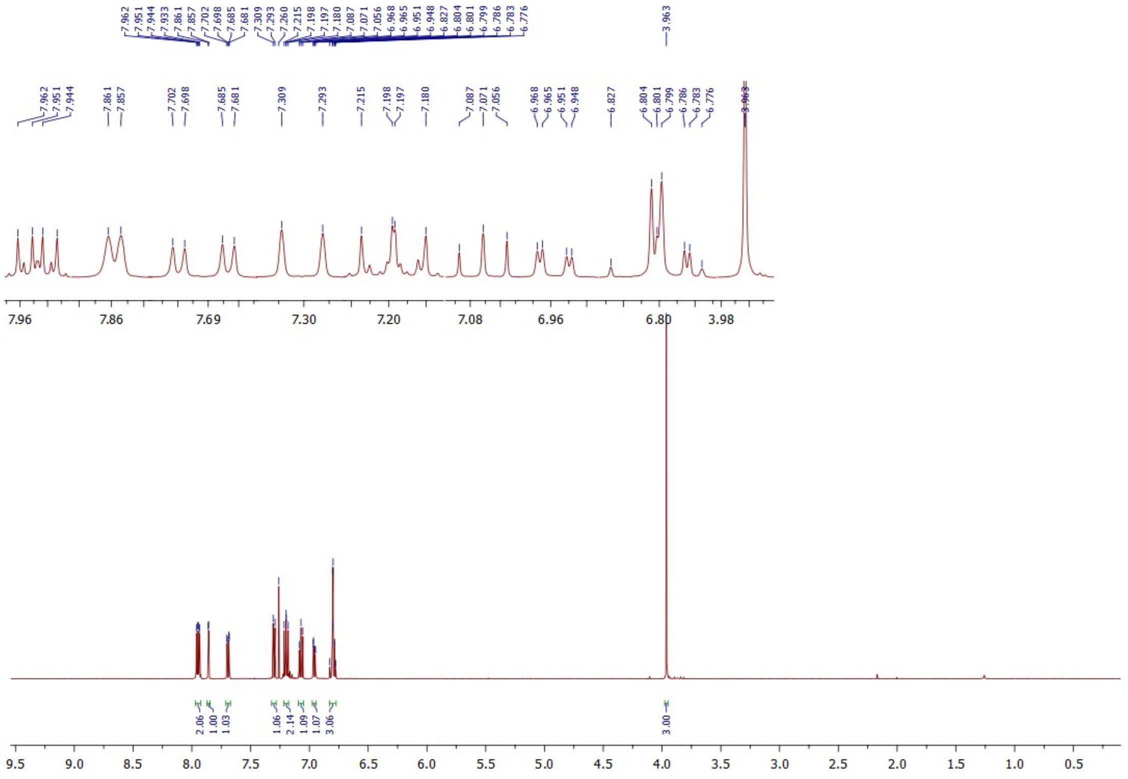

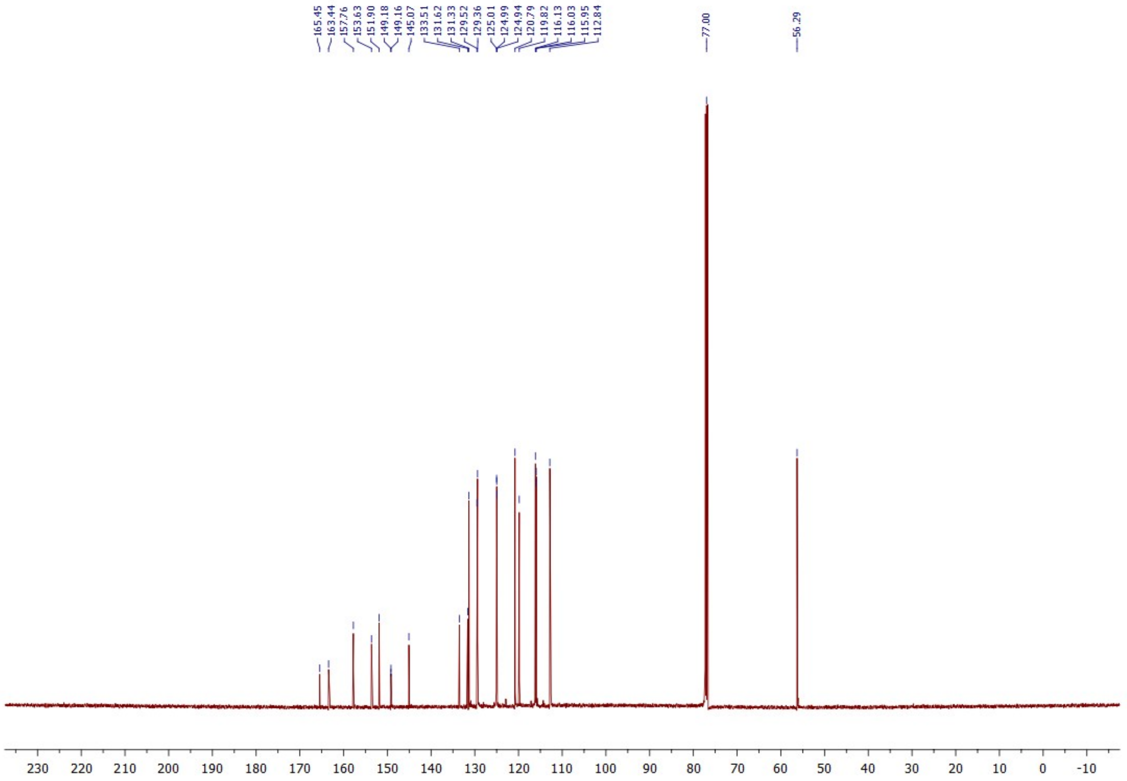

^{1}H NMR (500 MHz, $CDCl_3$, 298K): δ (ppm): 7.95 (2H, dd, $J_{H13,H14}$ = 9 Hz, $J_{H13,F}$ = 5.5 Hz, H_{13}), 7.86 (1H, d, $J_{H2,H4}$ = 2 Hz, H_4), 7.69 (1H, dd, $J_{H1,H2}$ = 8.5 Hz, H_2), 7.30 (1H, d, H_1), 7.20 (2H, dd, $J_{H14,F}$ = 9 Hz, H_{14}), 7.07 (1H, t, $J_{H8,H7,9}$ = 8 Hz, H_8), 6.96 (1H, dd, $J_{H7,H9}$ = 1.5 Hz, H_7), 6.82 (1H, AB spin system, d, $J_{H10,H11}$ = 11.5 Hz, H_{10}), 6.79 (1H, dd, H_9), 6.79 (1H, AB spin system, d, H_{11}), 3.96 (3H, s, OCH_3).
^{13}C NMR (125 MHz, $CDCl_3$, 298K): δ (ppm): 164.44 (d, $J_{C15,F}$ = 251.25 Hz, C15), 157.76 (C4'), 153.63 (C3), 151.90 (C6), 149.17 (d, $J_{C12,F}$ = 2.5 Hz, C12), 145.07 (C5'), 133.51 (C11'), 131.62 (C9'), 131.33 (C10), 129.52 (C1), 129.36 (C11), 124.99 (C9), 124.98 (d, $J_{C13,F}$ = 8.75 Hz, C13), 120.79 (C2), 119.82 (C8), 116.04 (d, $J_{C14,F}$ = 22.5 Hz, C14), 116.03 (C4), 112.84 (C7), 56.29 (OCH_3).
HRMS (ESI): *m/z* calculated for $C_{21}H_{15}FN_2O_2$+H 347.11903, found: 347.11867.

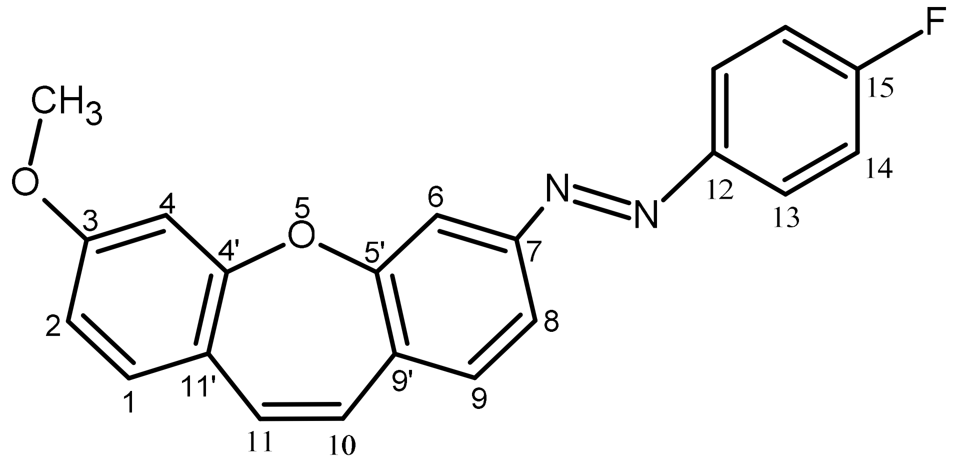

7c

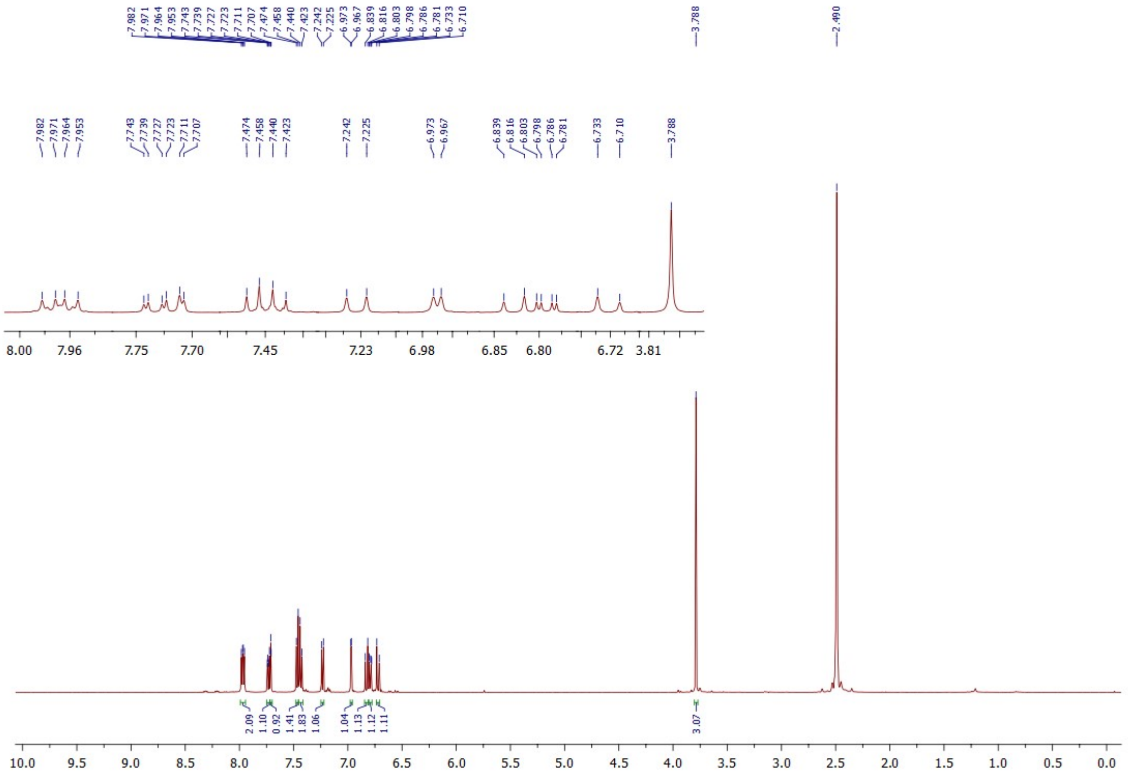

7.982
7.971
7.964
7.953
7.743
7.739
7.727
7.723
7.711
7.707
7.474
7.458
7.440
7.423
7.242
7.225
6.973
6.967
6.839
6.816
6.803
6.798
6.786
6.781
6.733
6.710
3.788
2.490
8.00 7.96 7.75 7.70 7.45 7.23 6.98 6.85 6.80 6.72 3.81
2.09 1.10 0.92 1.41 1.83 1.06 1.04 1.13 1.12 1.11 3.07
10.0 9.5 9.0 8.5 8.0 7.5 7.0 6.5 6.0 5.5 5.0 4.5 4.0 3.5 3.0 2.5 2.0 1.5 1.0 0.5 0.0

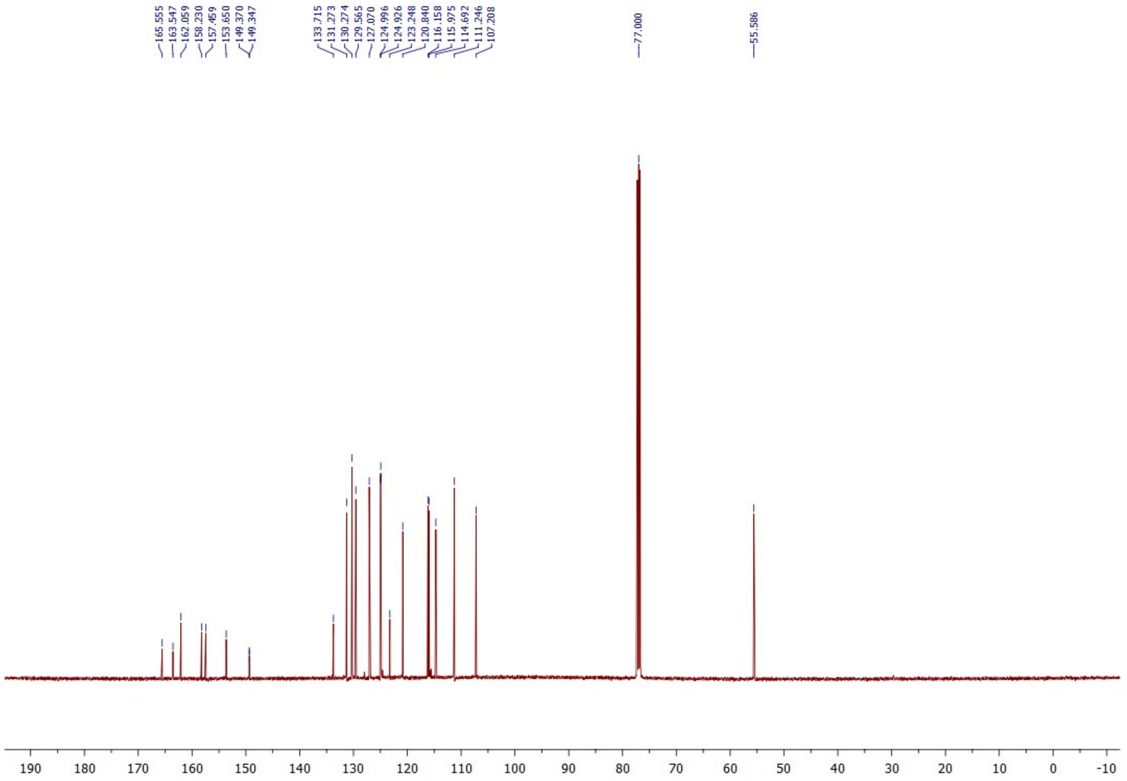

^{1}H NMR (500 MHz, DMSO-d$_6$, 298K): δ (ppm): 7.97 (2H, dd, $J_{H13,H14}$ = 9 Hz, $J_{H13,F}$ = 5.5 Hz, H_{13}), 7.73 (1H, dd, $J_{H8,H9}$ = 8 Hz, $J_{H6,H8}$ = 2 Hz, H_8), 7.71 (1H, d, H_6), 7.47 (1H, d, H_9), 7.44 (2H, t, $J_{H14,F}$ = 9 Hz, H_{14}), 7.23 (1H, d, $J_{H1,H2}$ = 8.5 Hz, H_1), 6.97 (1H, d, $J_{H2,H4}$ = 2.5 Hz, H_4), 6.83 (1H, AB spin system, d, $J_{H10,H11}$ = 11.5 Hz, H_{11}), 6.79 (1H, dd, H_2), 6.72 (1H, AB spin system, d, H_{10}), 3.79 (3H, s, OCH_3).

^{13}C NMR (125 MHz, $CDCl_3$, 298K): δ (ppm): 164.55 (d, $J_{C15,F}$ = 251 Hz, C15), 162.06 (C3), 158.23 (C4'), 157.46 (C5'), 153.65 (C7), 149.36 (d, $J_{C12,F}$ = 2.875 Hz, C12), 133.72 (C9'), 131.27 (C11), 130.27 (C1), 129.57 (C9), 127.07 (C10), 124.98 (d, $J_{C13,F}$ = 8.75 Hz, C13), 123.25 (C11'), 120.84 (C8), 116.09 (d, $J_{C14,F}$ = 22.875 Hz, C14), 114.69 (C6), 111.25 (C2), 107.21 (C4), 55.59 (OCH_3).

HRMS (ESI): *m/z* calculated for $C_{21}H_{15}FN_2O_2$+H 347.11903, found: 347.11871.

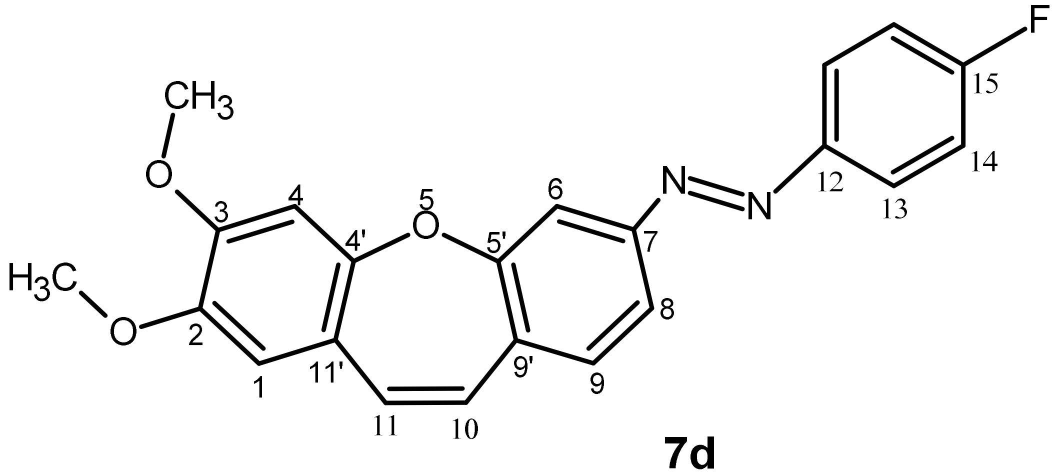

7d

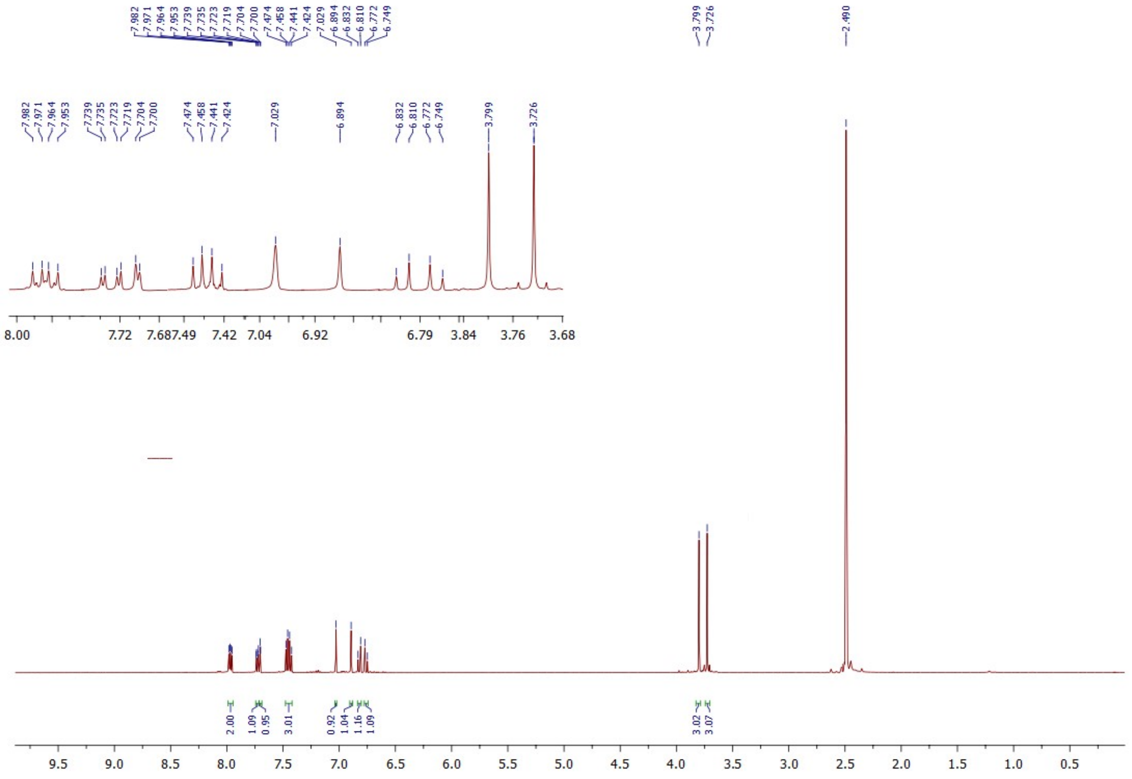

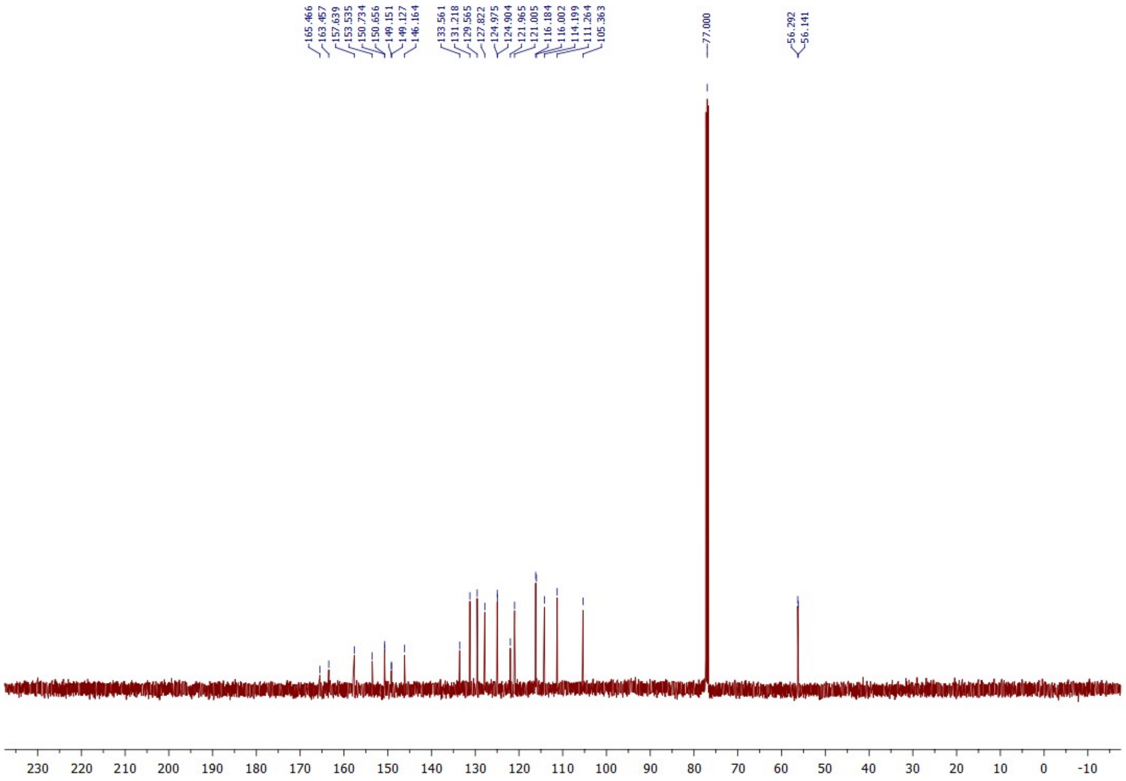

^{1}H NMR (500 MHz, DMSO-d$_6$, 298K): δ (ppm): 7.97 (2H, dd, $J_{H13,H14}$ = 9 Hz, $J_{H13,F}$ = 5.5 Hz, H_{13}), 7.73 (1H, dd, $J_{H8,H9}$ = 8 Hz, $J_{H6,H8}$ = 2 Hz, H_8), 7.70 (1H, d, H_6), 7.47 (1H, d, H_9), 7.44 (2H, t, $J_{H14,F}$ = 9 Hz, H_{14}), 7.03 (1H, s, H_4), 6.89 (1H, s, H_1), 6.82 (1H, AB spin system, d, $J_{H10,H11}$ = 11.5 Hz, H_{11}), 6.76 (1H, AB spin system, d, H_{10}), 3.80 (3H, s, OCH_3), 3.73 (3H, s, OCH_3).

^{13}C NMR (125 MHz, $CDCl_3$, 298K): δ (ppm): 164.46 (d, $J_{C15,F}$ = 251.125 Hz, C15), 157.64 (C5'), 153.54 (C7), 150.73 (C3), 150.66 (C4'), 149.14 (d, $J_{C12,F}$ = 3 Hz, C12), 146.16 (C2), 133.56 (C9'), 131.22 (C11), 129.56 (C9), 127.82 (C10), 124.94 (d, $J_{C13,F}$ = 8.875 Hz, C13), 121.97 (C11'), 121.01 (C8), 116.09 (56.29,d, $J_{C14,F}$ = 22.75 Hz, C14), 114.20 (C6), 111.26 (C1), 105.36 (C4), 56.29 (OCH_3), 56.14 (OCH_3).

HRMS (ESI): *m/z* calculated for $C_{22}H_{17}FN_2O_3$+H 377.12960, found: 377.12948.

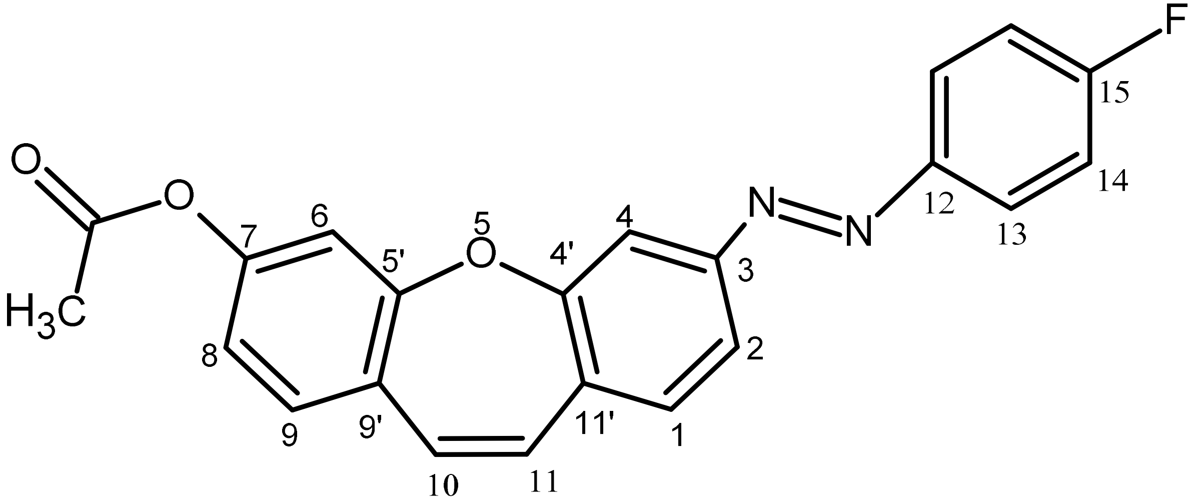

7e

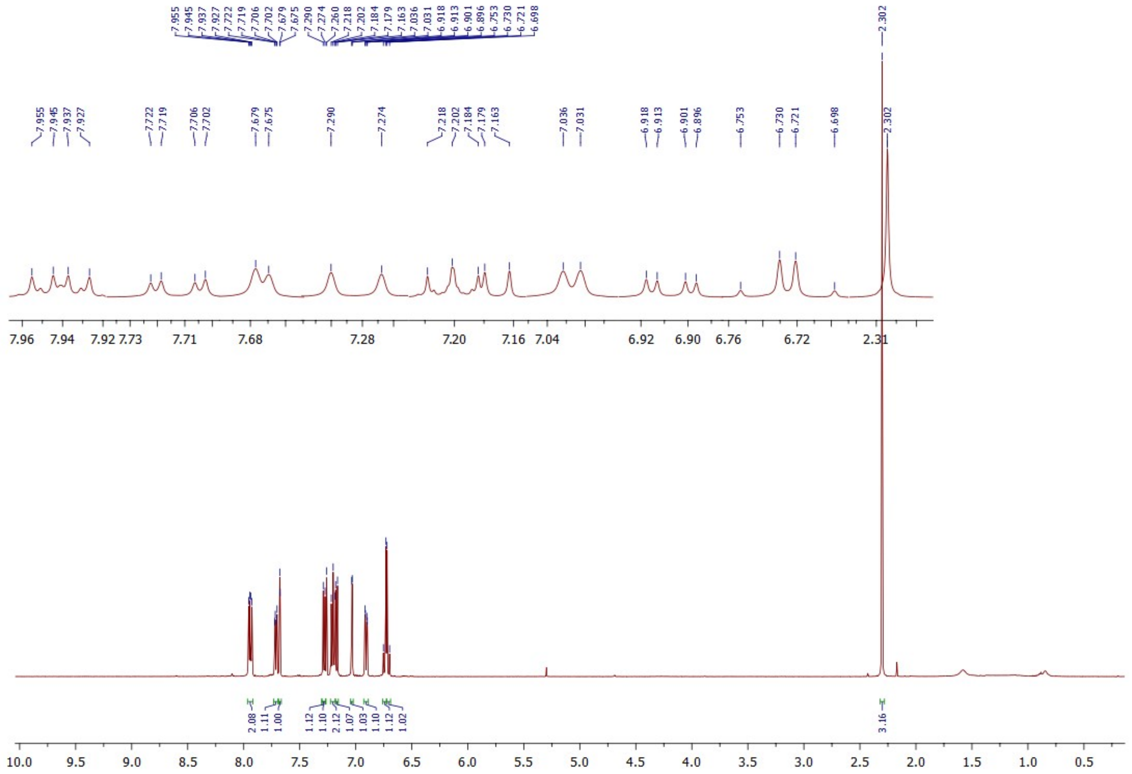

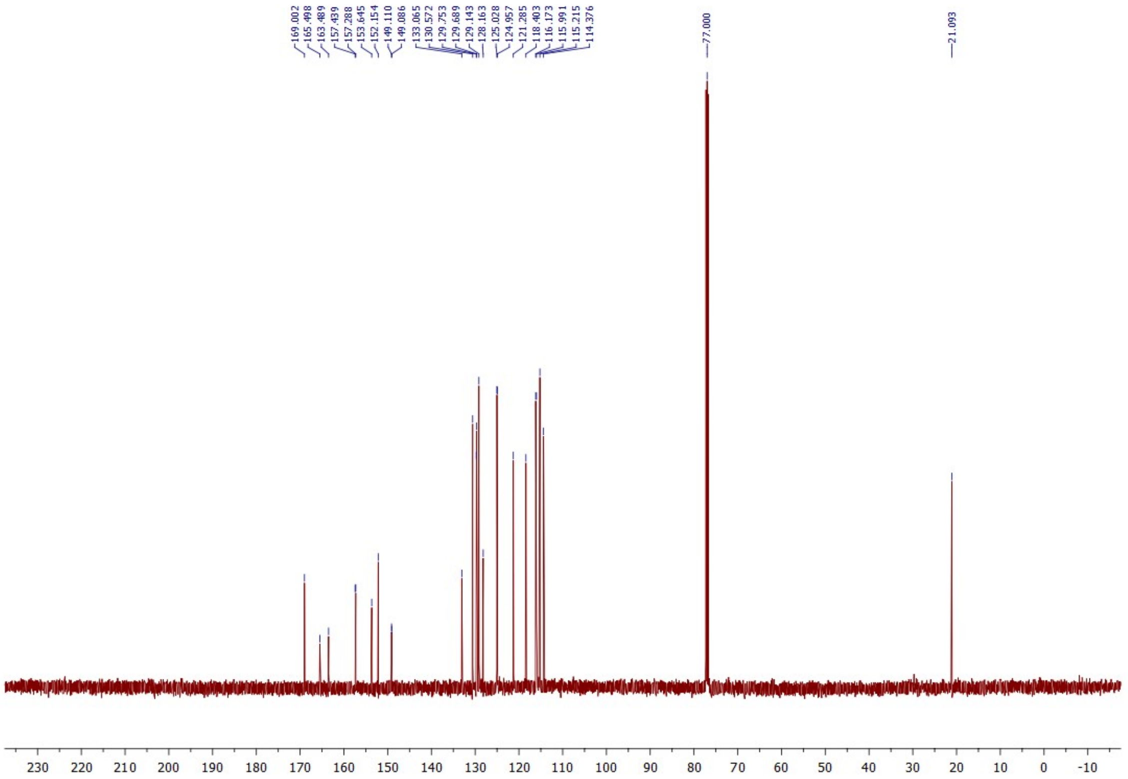

^{1}H NMR (500 MHz, $CDCl_3$, 298K): δ (ppm): 7.95 (2H, dd, $J_{H13,H14}$ = 9 Hz, $J_{H13,F}$ = 5 Hz, H_{13}), 7.71 (1H, dd, $J_{H1,H2}$ = 8.5 Hz, $J_{H2,H4}$ = 2 Hz, H_2), 7.68 (1H, d, H_4), 7.28 (1H, d, H_1), 7.20 (2H, dd, $J_{H14,F}$ = 9 Hz, H_{14}), 7.17 (1H, d, $J_{H8,H9}$ = 8.5 Hz, H_9), 7.03 (1H, d, $J_{H6,H8}$ = 2.5 Hz, H_6), 6.91 (1H, dd, H_8), 6.74 (1H, AB spin system, d, $J_{H10,H11}$ = 11.5 Hz, H_{10}), 6.71 (1H, AB spin system, d, H_{11}), 2.30 (3H, s, OCH_3).
^{13}C NMR (125 MHz, $CDCl_3$, 298K): δ (ppm): 169.00 ($C_{karb.}$), 164.49 (d, $J_{C15,F}$ = 251.13 Hz, C15), 157.44 (C5'), 157.29 (C4'), 153.65 (C3), 152.15 (C7), 149.10 (d, $J_{C12,F}$ = 3 Hz, C12), 133.07 (C11'), 130.57 (C10), 129.75 (C9), 129.69 (C1), 129.14 (C11), 128.16 (C9'), 124.99 (d, $J_{C13,F}$ = 8.75 Hz, C13), 121.29 (C2), 118.40 (C8), 116.08 (d, $J_{C14,F}$ = 22.75 Hz, C14), 115.22, 114.38 (C4), 21.09 (CH_3).
HRMS (ESI): *m/z* calculated for $C_{22}H_{15}FN_2O_3$+H 375.11395, found: 377.11403.

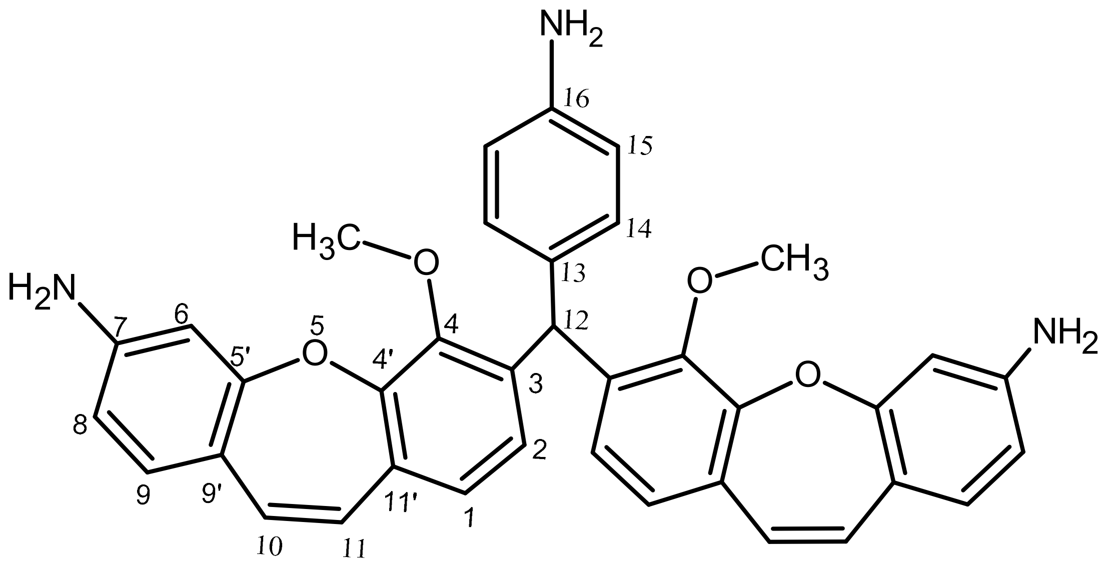

9c

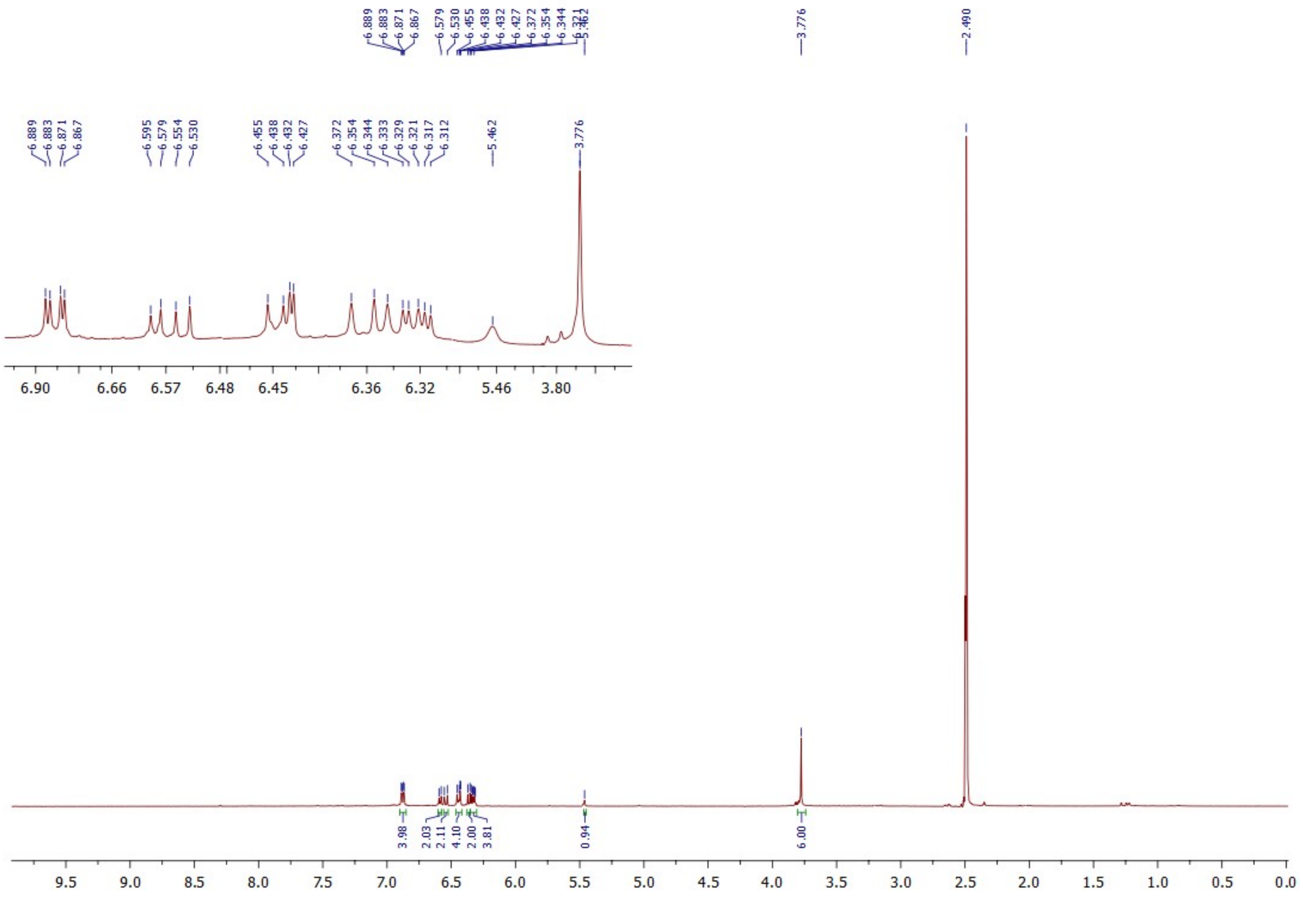

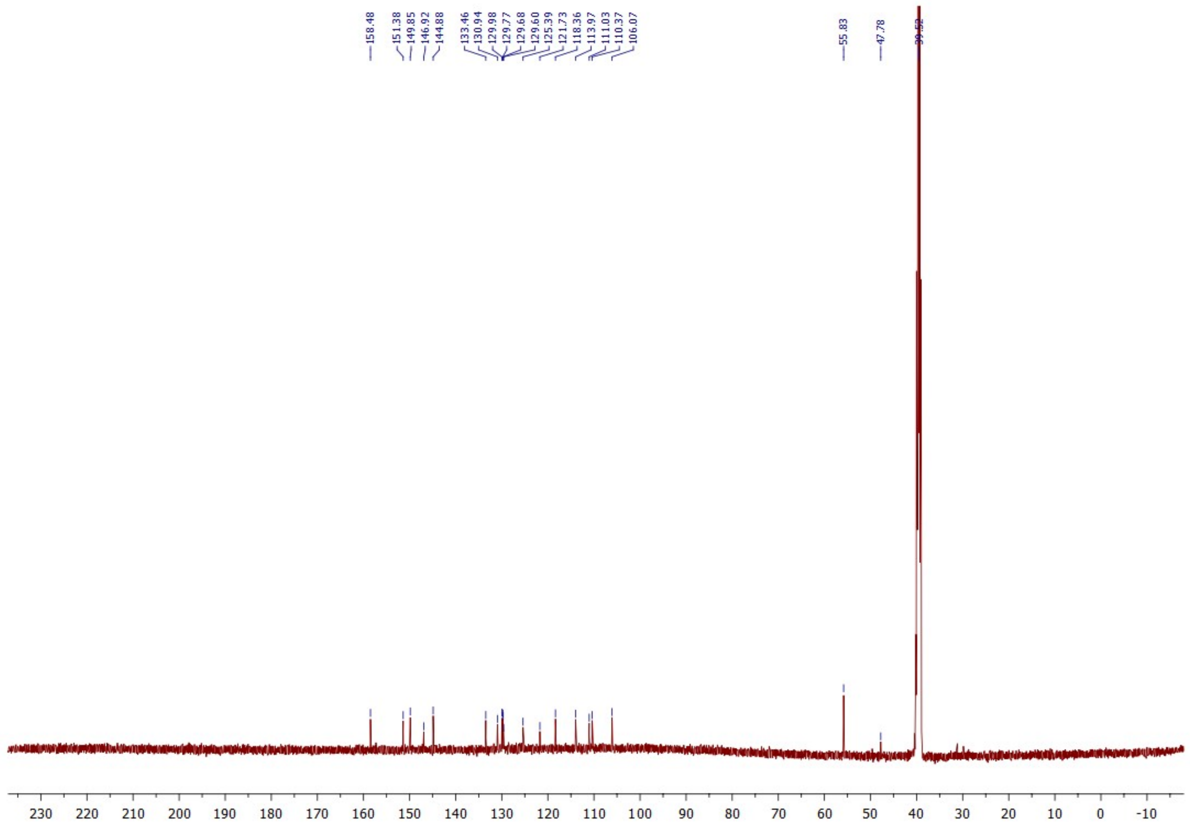

^{1}H NMR (500 MHz, DMSO-d$_6$, 298K): δ (ppm): 6.88 (2H, d, $J_{H8,H9}$ = 9 Hz, H$_9$), 6.88 (2H, d, $J_{H14,H15}$ = 8 Hz, H$_{14}$), 6.59 (2H, d, $J_{H1,H2}$ = 8 Hz, H$_1$), 6.54 (2H, AB spin system, d, $J_{H10,H11}$ = 11.5 Hz, H$_{10}$), 6.45 (2H, d, H$_2$), 6.43 (2H, d, $J_{H6,H8}$ = 2.5 Hz, H$_6$), 6.36 (2H, d, H$_{15}$), 6.33 (2H, AB spin system, d, H$_{11}$), 6.32 (2H, dd, H$_8$), 5.46 (1H, s, CH), 3.78 (6H, s, OCH$_3$).

^{13}C NMR (125 MHz, DMSO-d$_6$, 298K): δ (ppm): 158.48, 151.38, 149.85, 146.92, 144.88, 133.46, 130.94, 129.98, 129.77, 129.68, 129.60, 125.39, 121.73, 118.36, 113.97, 111.03, 110.37, 106.07, 55.83, 47.78.

HRMS (ESI): *m/z* calculated for $C_{37}H_{31}N_3O_4$+H 582.23873, found: 582.23869.

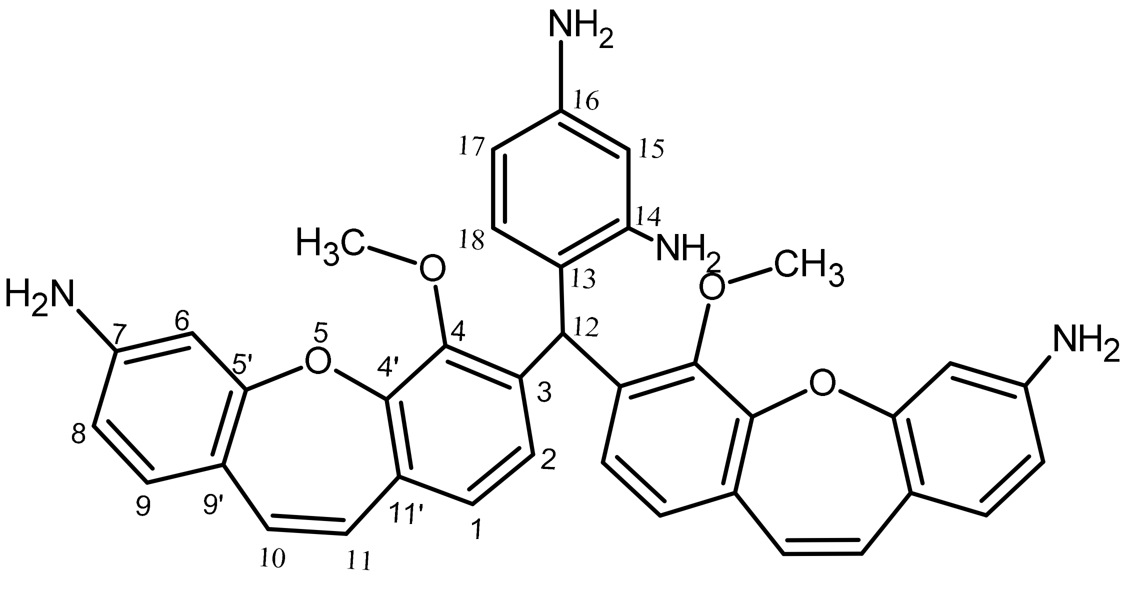
NH2
16
17
15
14
18
NH2
H3C
O
O
CH3
13
H2N
6
12
NH2
7
5
O
4
5'
O
4'
3
8
2
9
9'
11'
1
10
11

9d

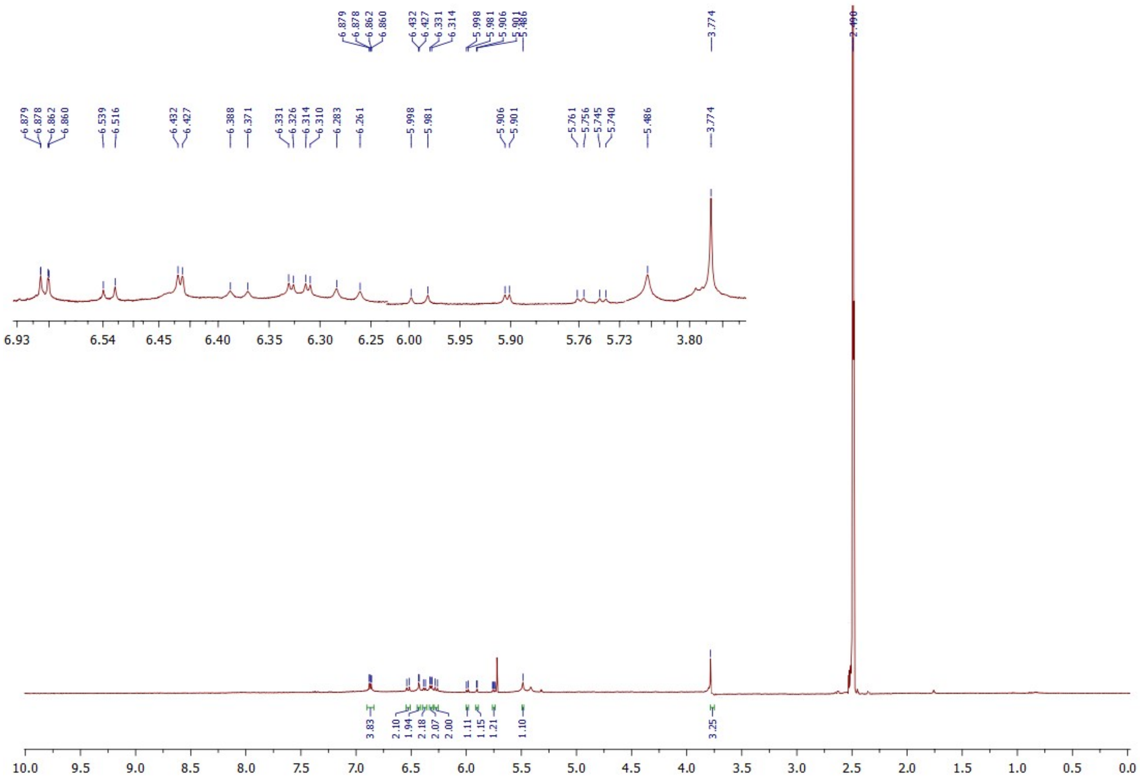

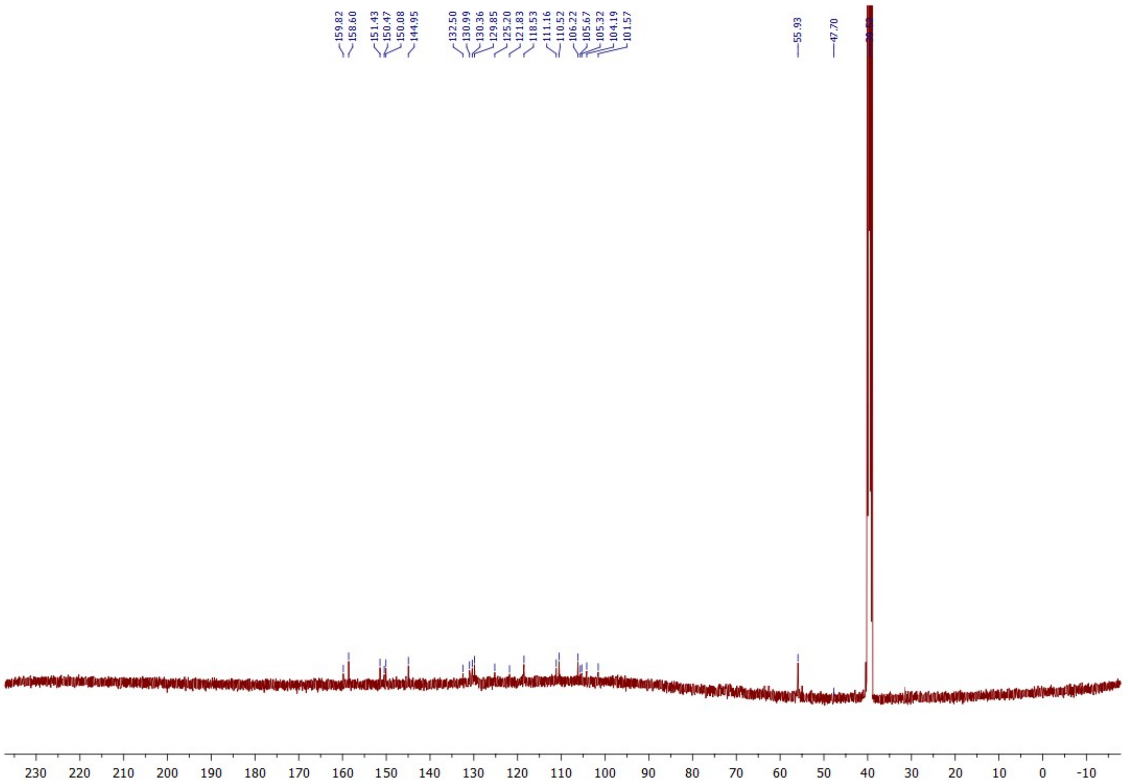

^{1}**H NMR** (500 MHz, DMSO-d$_6$, 298K): δ (ppm): 6.87 (2H, d, $J_{H8,H9}$ = 8.5 Hz, H_9), 6.87 (1H, d, $J_{H17,H18}$ = 9 Hz, H_{18}), 6.53 (2H, AB spin system, d, $J_{H10,H11}$ = 11.5 Hz, H_{10}), 6.43 (2H, d, $J_{H6,H8}$ = 2.5 Hz, H_6), 6.38 (2H, d, $J_{H1,H2}$ = 8.5 Hz, H_1), 6.32 (2H, dd, $J_{H6,H8}$ = 2.5 Hz, H_8), 6.27 (2H, AB spin system, d, H_{11}), 5.99 (2H, d, H_2), 5.90 (1H, d, $J_{H15,H17}$ = 2.5 Hz, H_{15}), 5.75 (1H, dd, H_{17}), 5.49 (1H, s, CH), 3.77 (3H, s, OCH_3).

^{13}**C NMR** (125 MHz, DMSO-d$_6$, 298K): δ (ppm): 159.82, 158.60, 151.43, 150.47, 150.08, 144.95, 132.50, 130.99, 130.36, 129.85, 125.20, 121.83, 118.53, 111.16, 110.52, 106.22, 105.67, 105.32, 104.19, 101.57, 55.93, 47.70.

HRMS (ESI): *m/z* calculated for $C_{37}H_{32}N_4O_4$+H 597.24963, found: 597.24930.

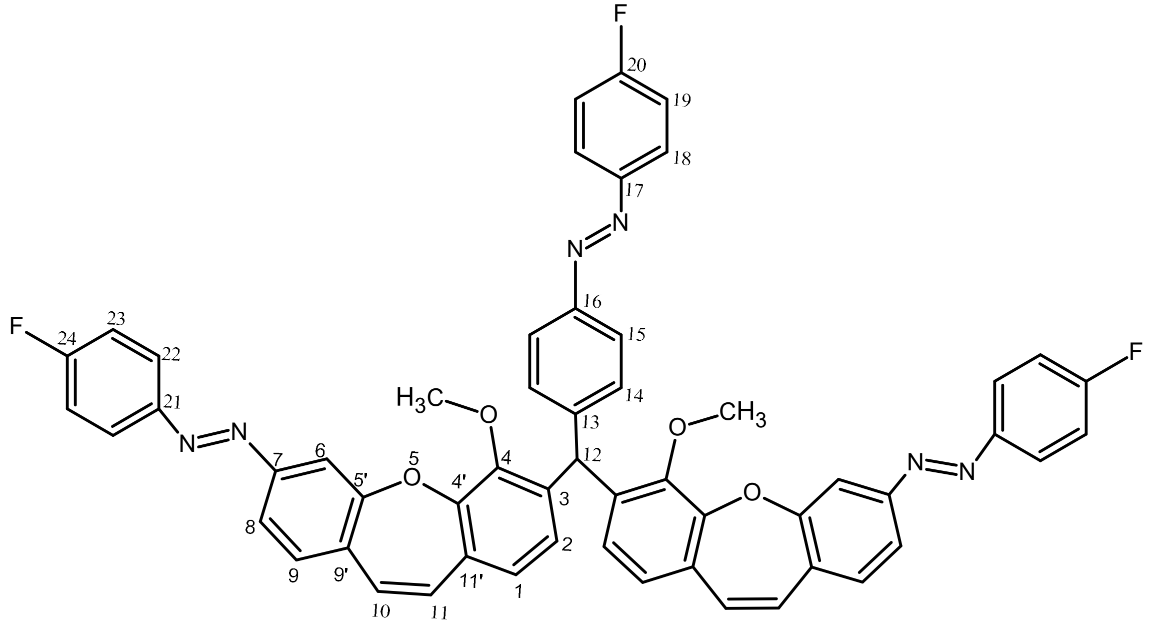

F
20
19
18
17
N
N
16
15
14
13
12
F
24
23
22
21
N
N
7
6
5
5'
O
4'
4
O
H_3C
3
2
1
11'
11
10
9'
9
8
O
CH_3
O
N
N
F

9e

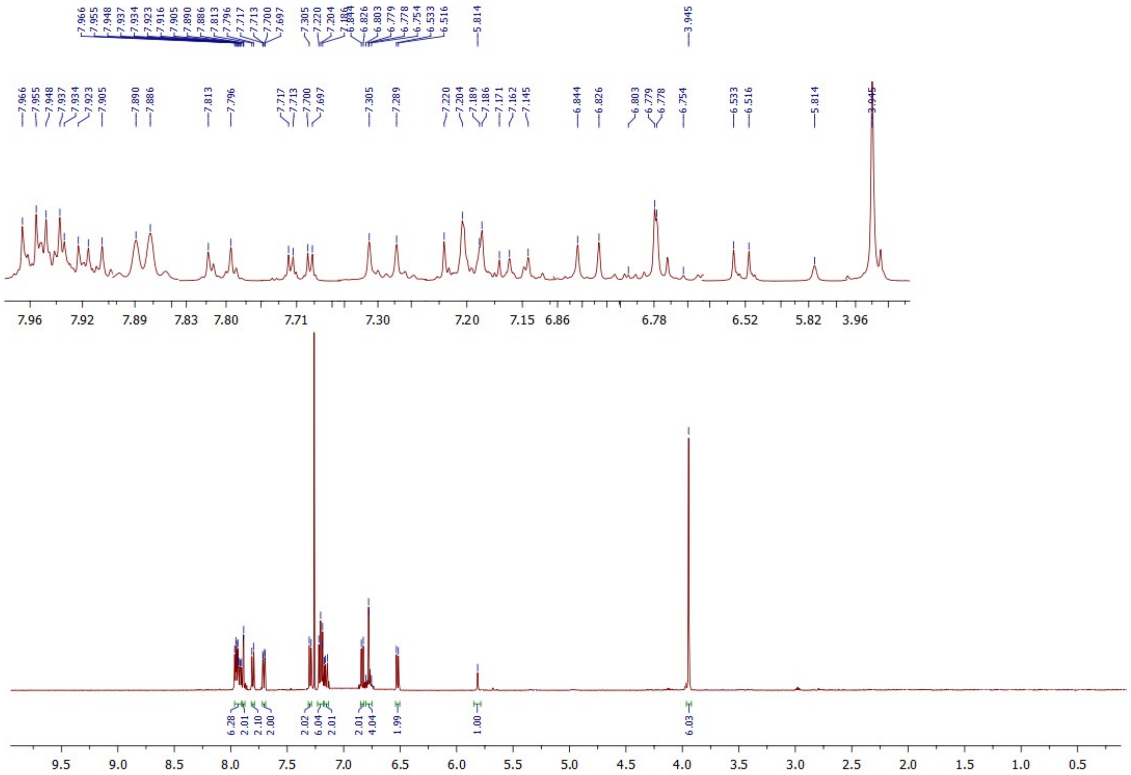

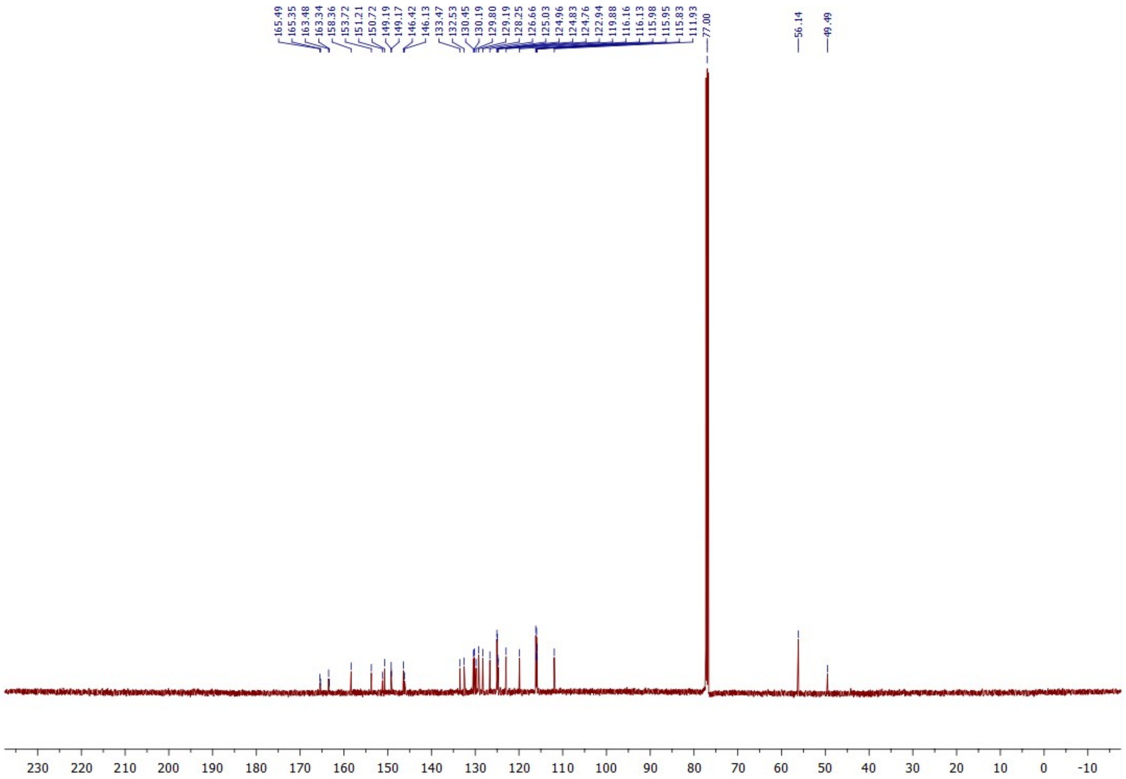

^{1}**H NMR** (500 MHz, $CDCl_3$, 298K): δ (ppm): 7.95 (4H, dd, $J_{H22,H23}$ = 9 Hz, $J_{H22,F}$ = 5.5 Hz, H_{22}), 7.92 (4H, dd, $J_{H18,H19}$ = 9 Hz, $J_{H18,F}$ = 5.5 Hz, H_{18}), 7.89 (2H, d, $J_{H6,H8}$ = 2 Hz, H_6), 7.80 (2H, d, $J_{H14,H15}$ = 8.5 Hz, H_{15}), 7.71 (2H, dd, $J_{H8,H9}$ = 8.5 Hz, H_8), 7.30 (2H, d, H_9), 7.20 (4H, dd, $J_{H23,F}$ = 9 Hz, H_{23}), 7.19 (2H, dd, $J_{H19,F}$ = 9 Hz, H_{19}), 7.15 (2H, d, H_{14}), 6.84 (2H, d, $J_{H1,H2}$ = 8.5 Hz, H_1), 6.79 (2H, AB spin system, d, $J_{H10,H11}$ = 12 Hz, H_{10}), 6.77 (2H, AB spin system, d, H_{11}), 6.52 (2H, d, H_2), 5.81 (1H, s , CH), 3.95 (6H, s, OCH_3).

^{13}**C NMR** (125 MHz, $CDCl_3$, 298K): δ (ppm): 164.42 (d, $J_{C24,F}$ = 250 Hz, C24), 164.35 (d, $J_{C20,F}$ = 250.75 Hz, C20), 158.36, 153.72, 151.21, 150.72, 149.18 (d, $J_{C21,F}$ = 2.625 Hz, C21), 149.18 (d, $J_{C17,F}$ = 2.625 Hz, C17), 146.42, 146.13, 133.47, 132.53, 130.45, 130.19, 129.80, 129.19, 128.25, 126.66, 125.00 (d, $J_{C22,F}$ = 8.875 Hz, C22), 124.79 (d, $J_{C18,F}$ = 8.625 Hz, C18), 122.94, 119.88, 116.07 (d, $J_{C23,F}$ = 22.75 Hz, C23), 116.04 (d, $J_{C19,F}$ = 22.75 Hz, C19), 115.83, 111.93, 56.14, 49.49.

HRMS (ESI): *m/z* calculated for $C_{55}H_{38}F_3N_6O_4$ 903.29011, found: 903.29020.

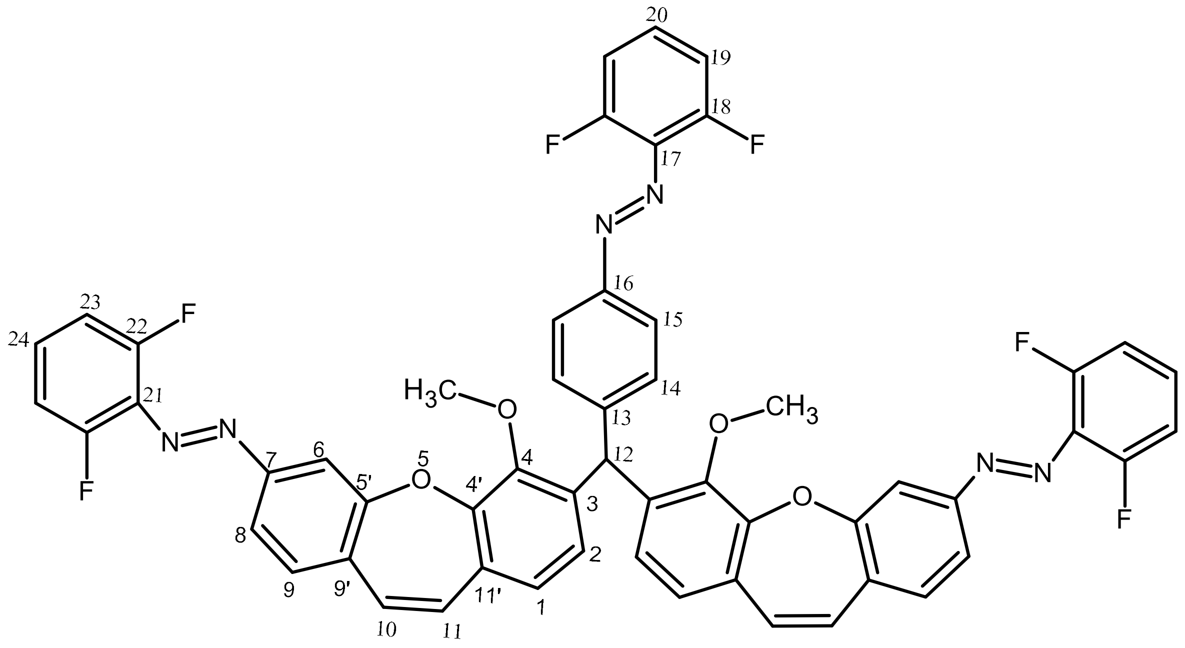
20
19
18
F
F
17
N
N
16
15
23
22
F
24
21
14
H3C
O
13
O
CH3
N
N
6
7
5
4'
4
12
F
O
N
N
5'
3
O
F
8
2
F
9
9'
11'
1
10
11

9f

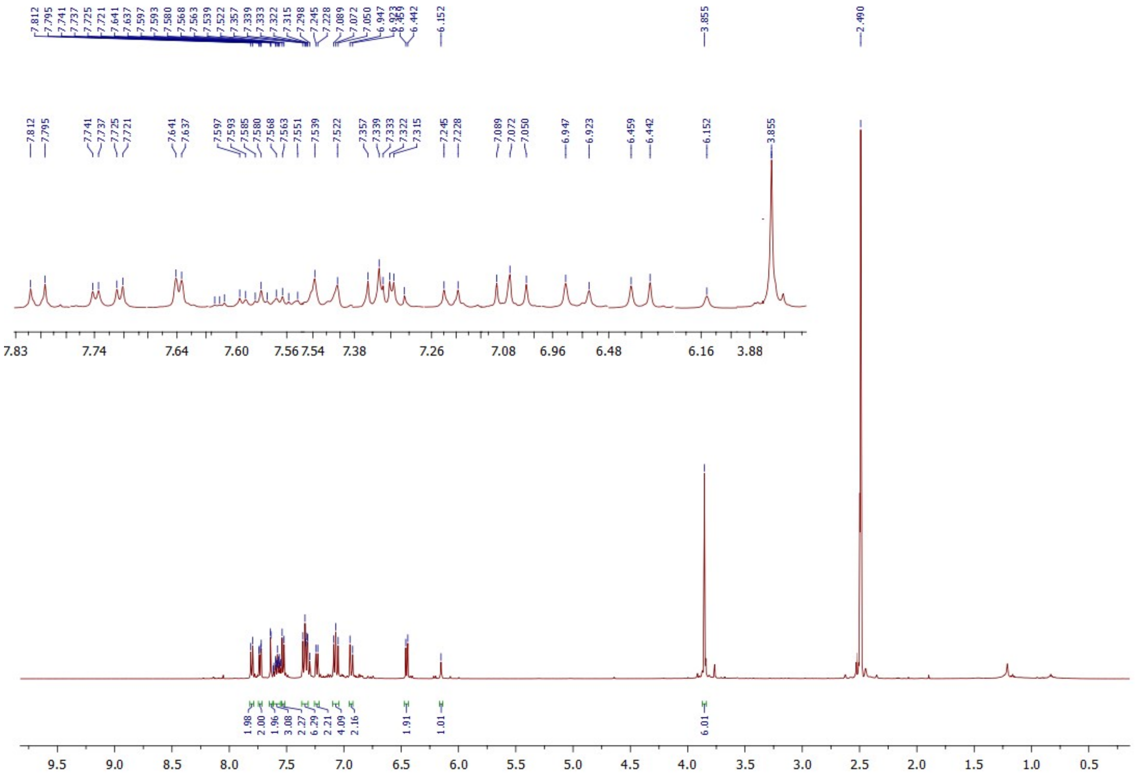

[1]**H NMR** (500 MHz, DMSO-d_6, 298K): δ (ppm): 7.80 (2H, d, $J_{H14,H15}$ = 8.5 Hz, H_{15}), 7.73 (2H, dd, $J_{H8,H9}$ = 8 Hz, $J_{H6,H8}$ = 2 Hz, H_8), 7.64 (2H, d, H_6), 7.58 (2H, tt, $J_{H23,H24}$ = 8.5 Hz, $J_{H24,F}$ = 6.5 Hz, H_{24}), 7.58 (1H, tt, $J_{H19,H20}$ = 8.5 Hz, $J_{H20,F}$ = 6.5 Hz, H_{20}), 7.53 (2H, d, H_9), 7.34 (4H, t, $J_{H23,F}$ = 8.5 Hz, H_{23}), 7.32 (2H, t, $J_{H19,F}$ = 8.5 Hz, H_{19}), 7.24 (2H, d, H_{14}), 7.08 (2H, d, $J_{H1,H2}$ = 8.5 Hz, H_1), 7.06 (2H, AB spin system, d, $J_{H10,H11}$ = 11.5 Hz, H_{11}), 6.94 (2H, AB spin system, d, H_{10}), 6.45 (2H, d, H_2), 6.15 (1H, s, CH), 3.86 (6H, s, OCH_3).

HRMS (ESI): *m/z* calculated for $C_{55}H_{34}F_6N_6O_4$+H 957.26185, found: 957.26190

Photostationary state (PSS) compositions for dibenzo[*b*,*f*]oxepin-diazenes (**7a-7e**) and (**9e**) and (**9f**) determined by ^{19}F NMR analysis (c ≈ 100 mM in DMSO-*d*6). The spectrum marked "dark" shows fluorine signals for the *E* stereoisomer.

7a:

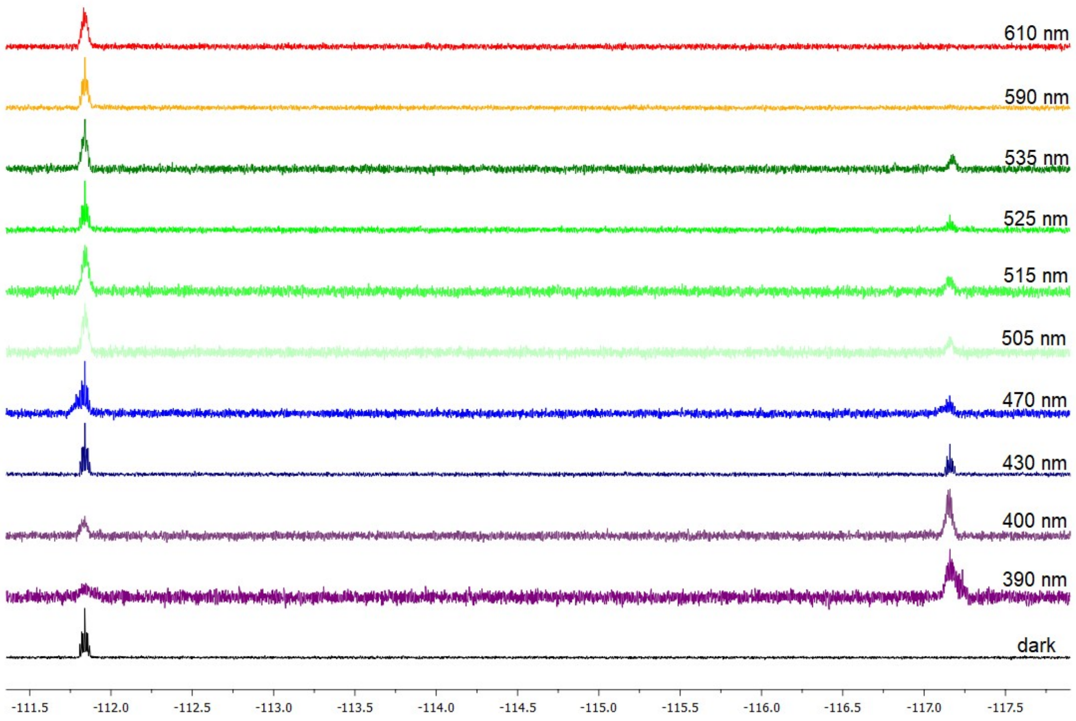

7b:

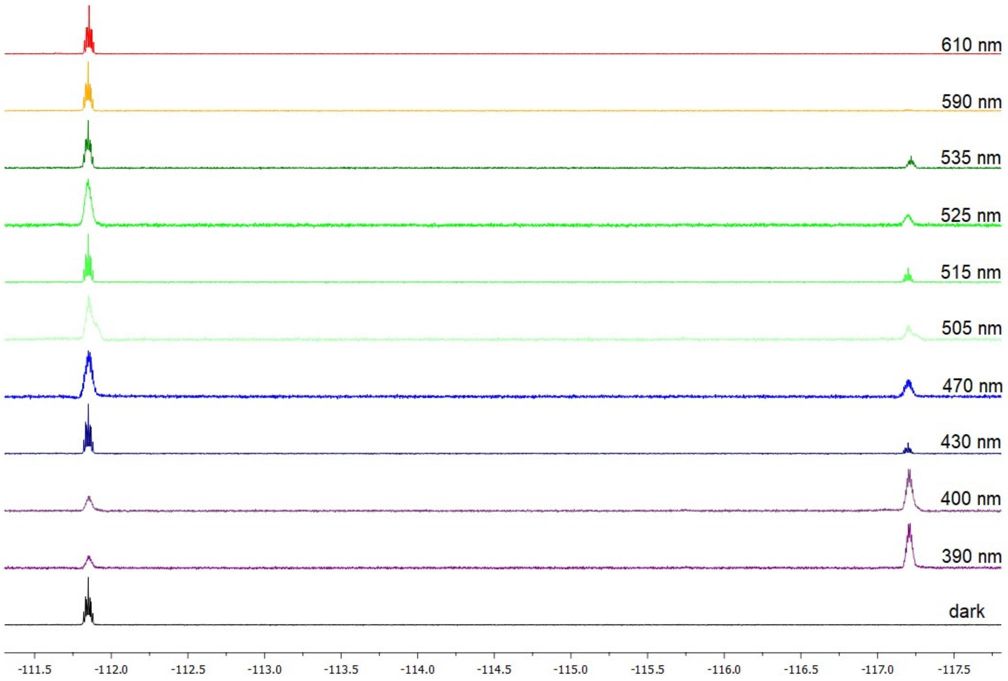

610 nm
590 nm
535 nm
525 nm
515 nm
505 nm
470 nm
430 nm
400 nm
390 nm
dark
-111.5
-112.0
-112.5
-113.0
-113.5
-114.0
-114.5
-115.0
-115.5
-116.0
-116.5
-117.0
-117.5

7c:

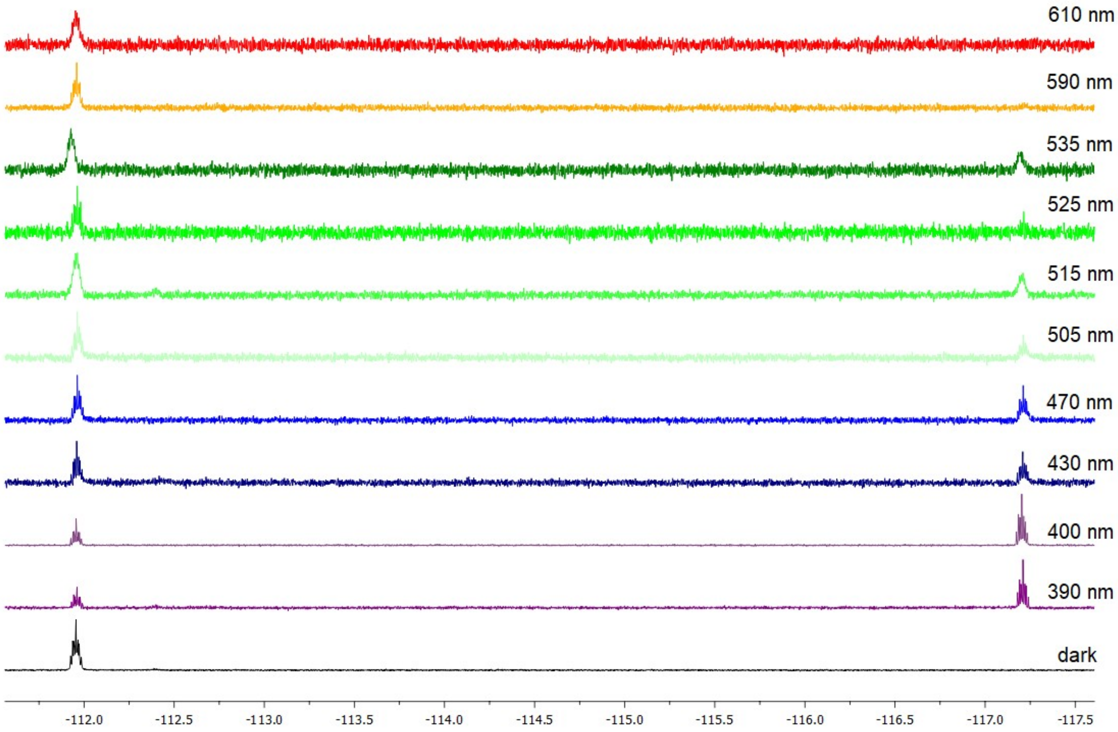

610 nm
590 nm
535 nm
525 nm
515 nm
505 nm
470 nm
430 nm
400 nm
390 nm
dark
-112.0
-112.5
-113.0
-113.5
-114.0
-114.5
-115.0
-115.5
-116.0
-116.5
-117.0
-117.5

7d:

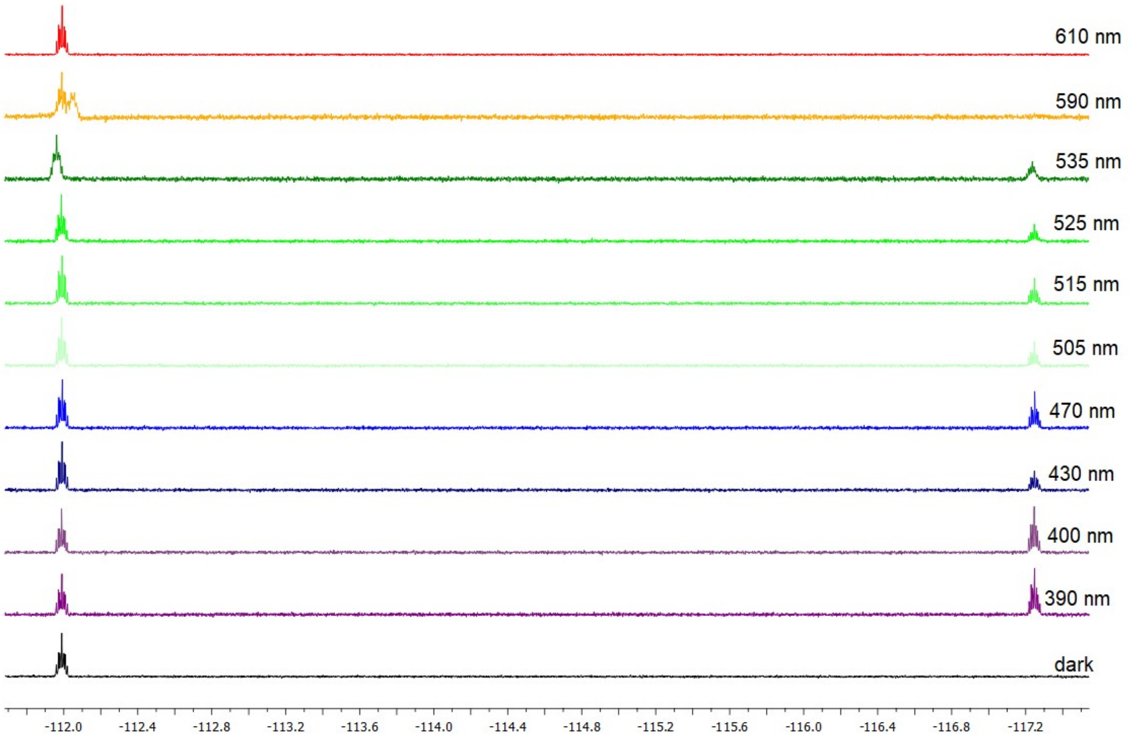
610 nm
590 nm
535 nm
525 nm
515 nm
505 nm
470 nm
430 nm
400 nm
390 nm
dark
-112.0
-112.4
-112.8
-113.2
-113.6
-114.0
-114.4
-114.8
-115.2
-115.6
-116.0
-116.4
-116.8
-117.2

7e:

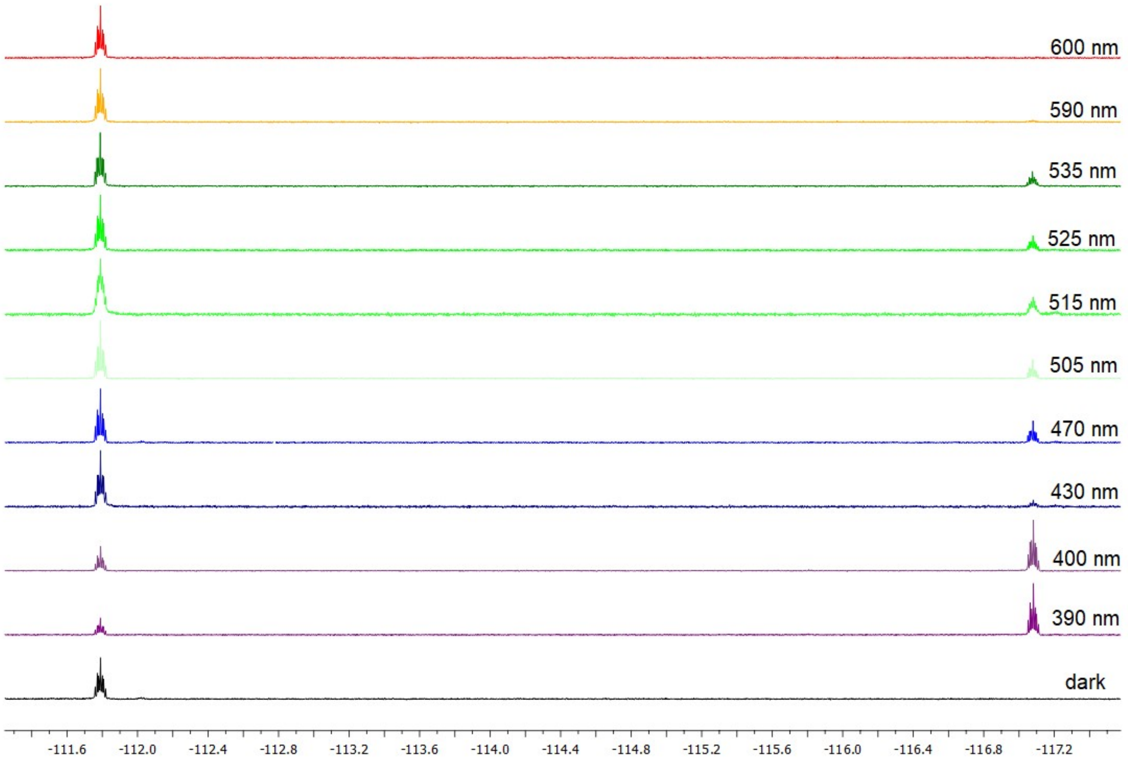
600 nm
590 nm
535 nm
525 nm
515 nm
505 nm
470 nm
430 nm
400 nm
390 nm
dark
-111.6
-112.0
-112.4
-112.8
-113.2
-113.6
-114.0
-114.4
-114.8
-115.2
-115.6
-116.0
-116.4
-116.8
-117.2

9e:

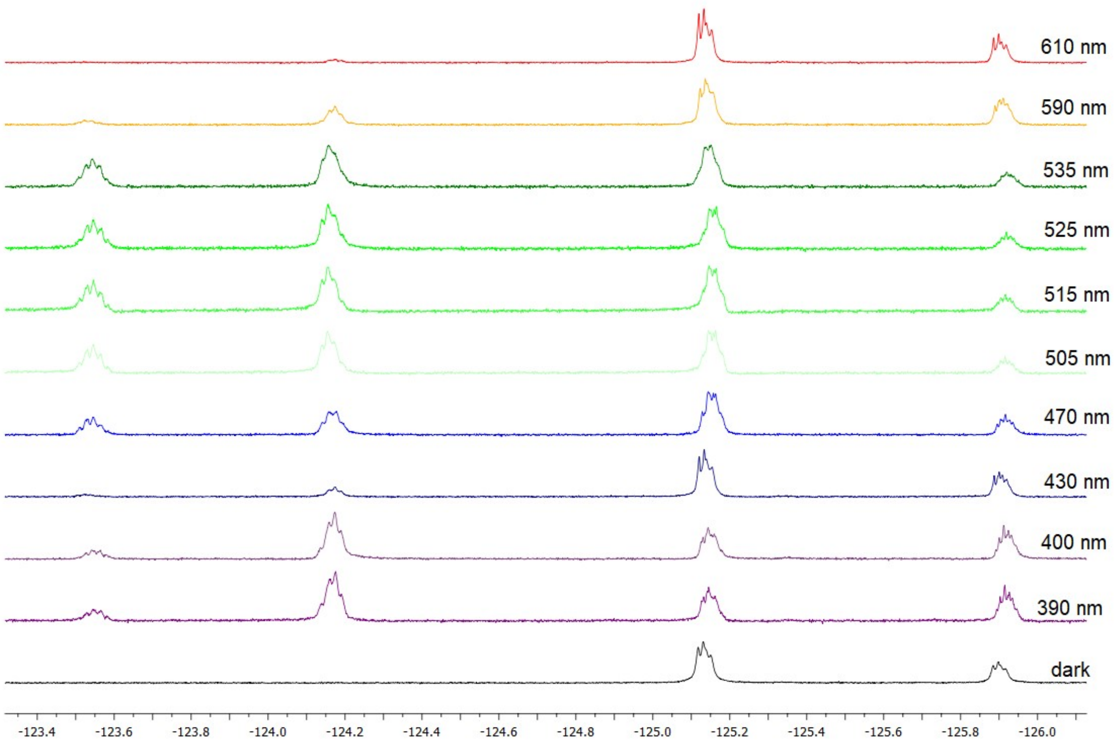
610 nm
590 nm
535 nm
525 nm
515 nm
505 nm
470 nm
430 nm
400 nm
390 nm
dark
-123.4
-123.6
-123.8
-124.0
-124.2
-124.4
-124.6
-124.8
-125.0
-125.2
-125.4
-125.6
-125.8
-126.0

9f:

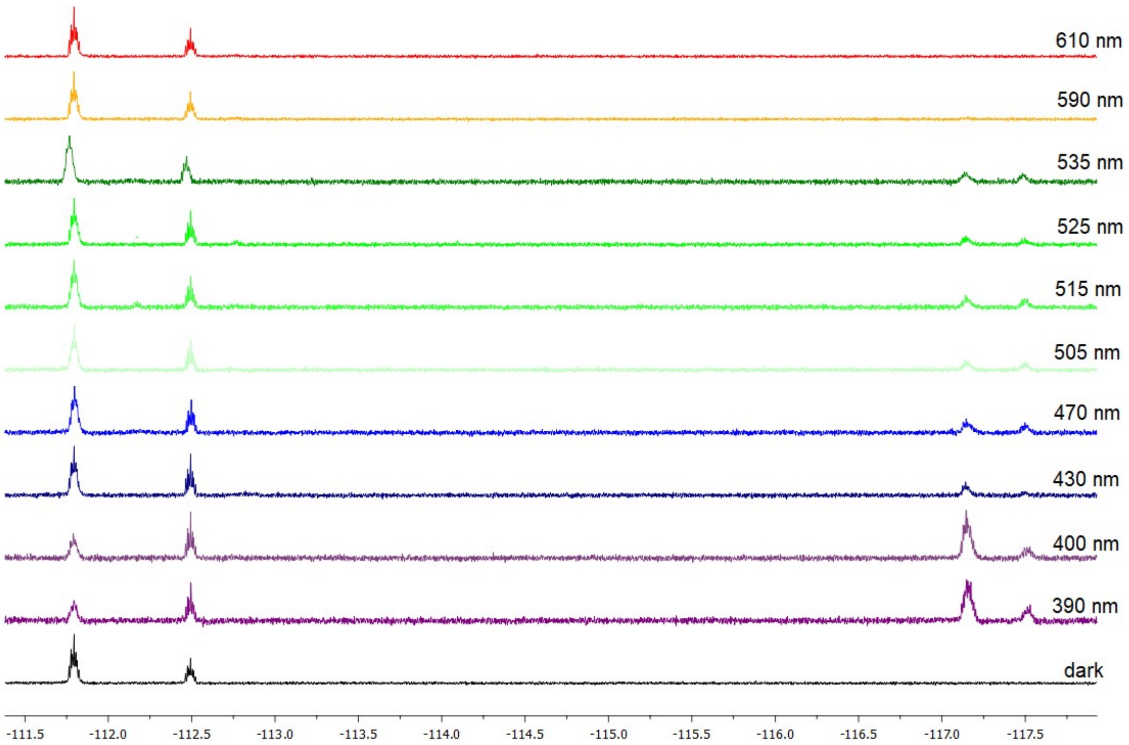

610 nm
590 nm
535 nm
525 nm
515 nm
505 nm
470 nm
430 nm
400 nm
390 nm
dark
-111.5
-112.0
-112.5
-113.0
-113.5
-114.0
-114.5
-115.0
-115.5
-116.0
-116.5
-117.0
-117.5

Changes over time (photoisomerization kinetics) transitions from *Z* to *E* upon irradiation for (**7a-7e**) irradiated 390 nm and for (**9e**) irradiated 535 nm (for sample concentration 100 mM in DMSO) determined by ^{19}F NMR analysis. The spectrum marked “dark” shows fluorine signals for the predominant amount of *Z* stereoisomer.

7a:

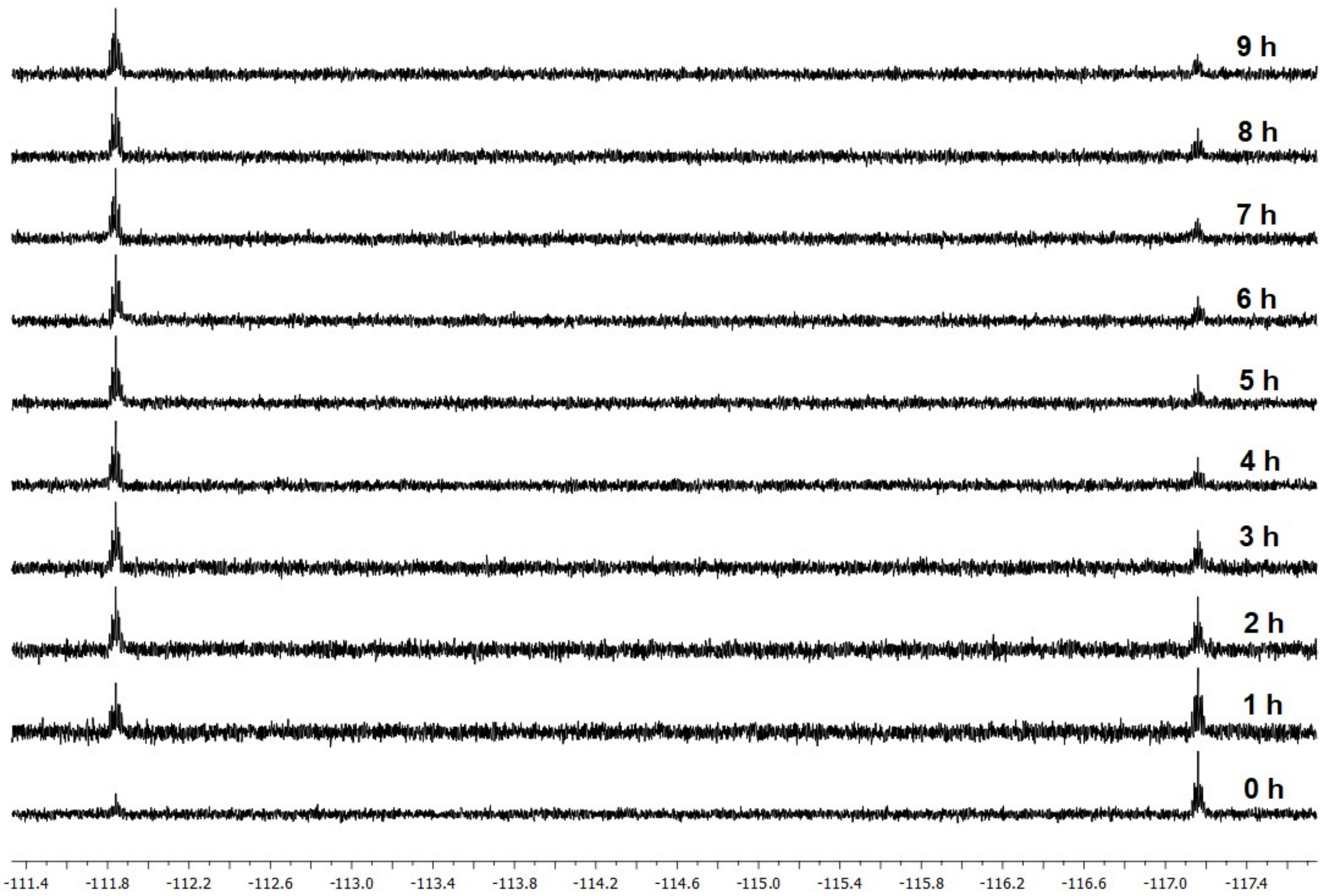

7b:

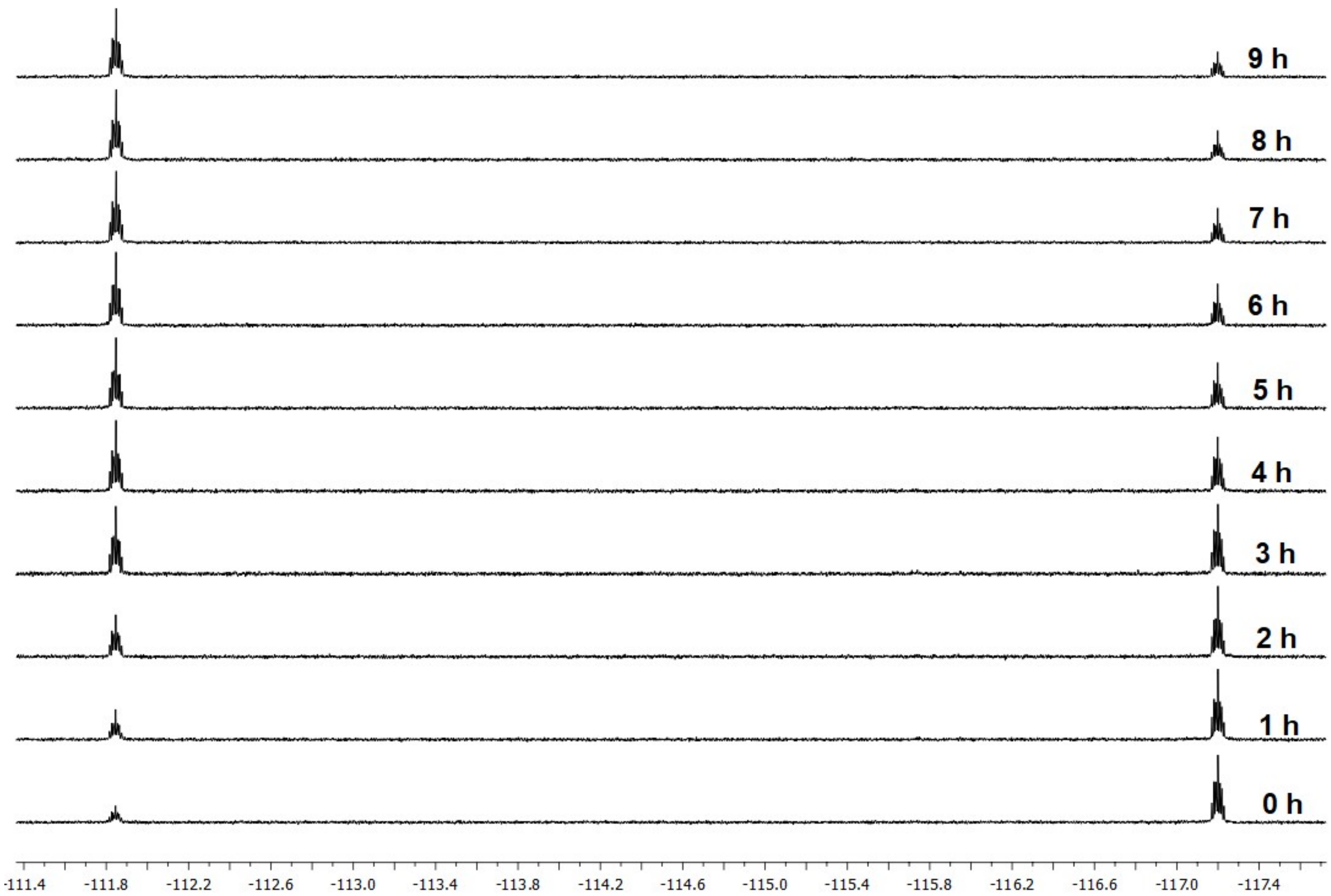
9 h
8 h
7 h
6 h
5 h
4 h
3 h
2 h
1 h
0 h
-111.4
-111.8
-112.2
-112.6
-113.0
-113.4
-113.8
-114.2
-114.6
-115.0
-115.4
-115.8
-116.2
-116.6
-117.0
-117.4

7c:

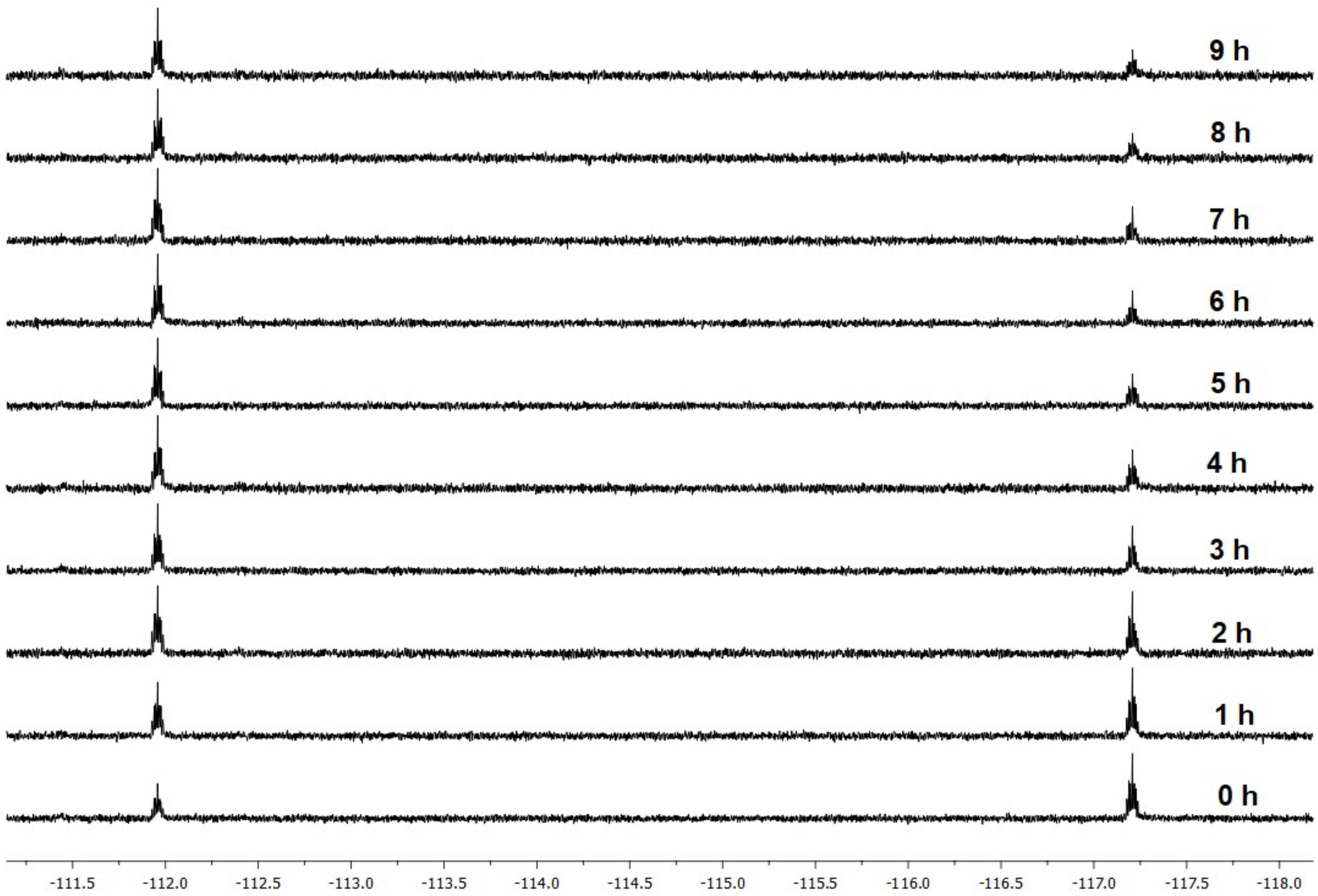
9 h
8 h
7 h
6 h
5 h
4 h
3 h
2 h
1 h
0 h
-111.5
-112.0
-112.5
-113.0
-113.5
-114.0
-114.5
-115.0
-115.5
-116.0
-116.5
-117.0
-117.5
-118.0

7d:

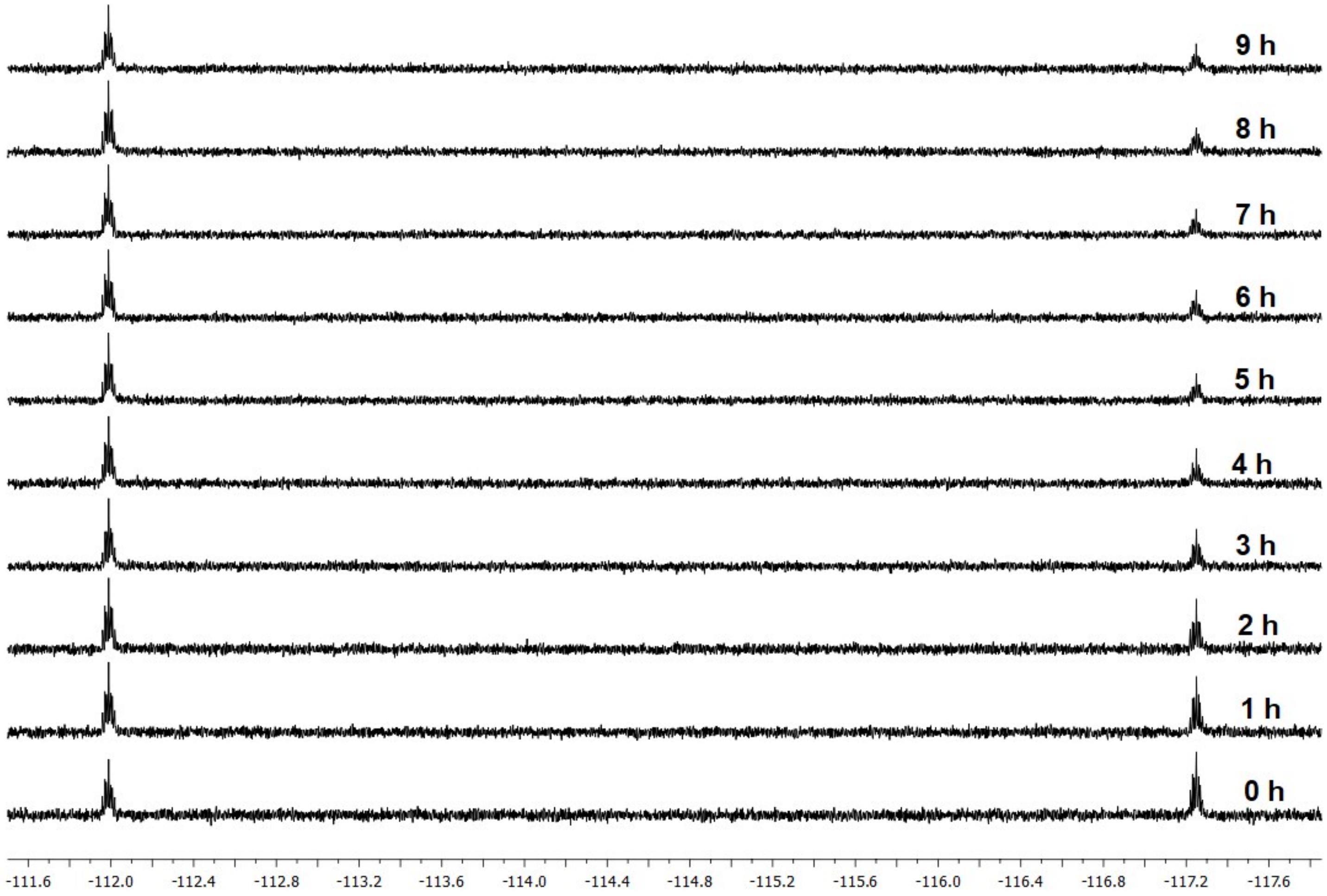
9 h
8 h
7 h
6 h
5 h
4 h
3 h
2 h
1 h
0 h
-111.6 -112.0 -112.4 -112.8 -113.2 -113.6 -114.0 -114.4 -114.8 -115.2 -115.6 -116.0 -116.4 -116.8 -117.2 -117.6

7e:

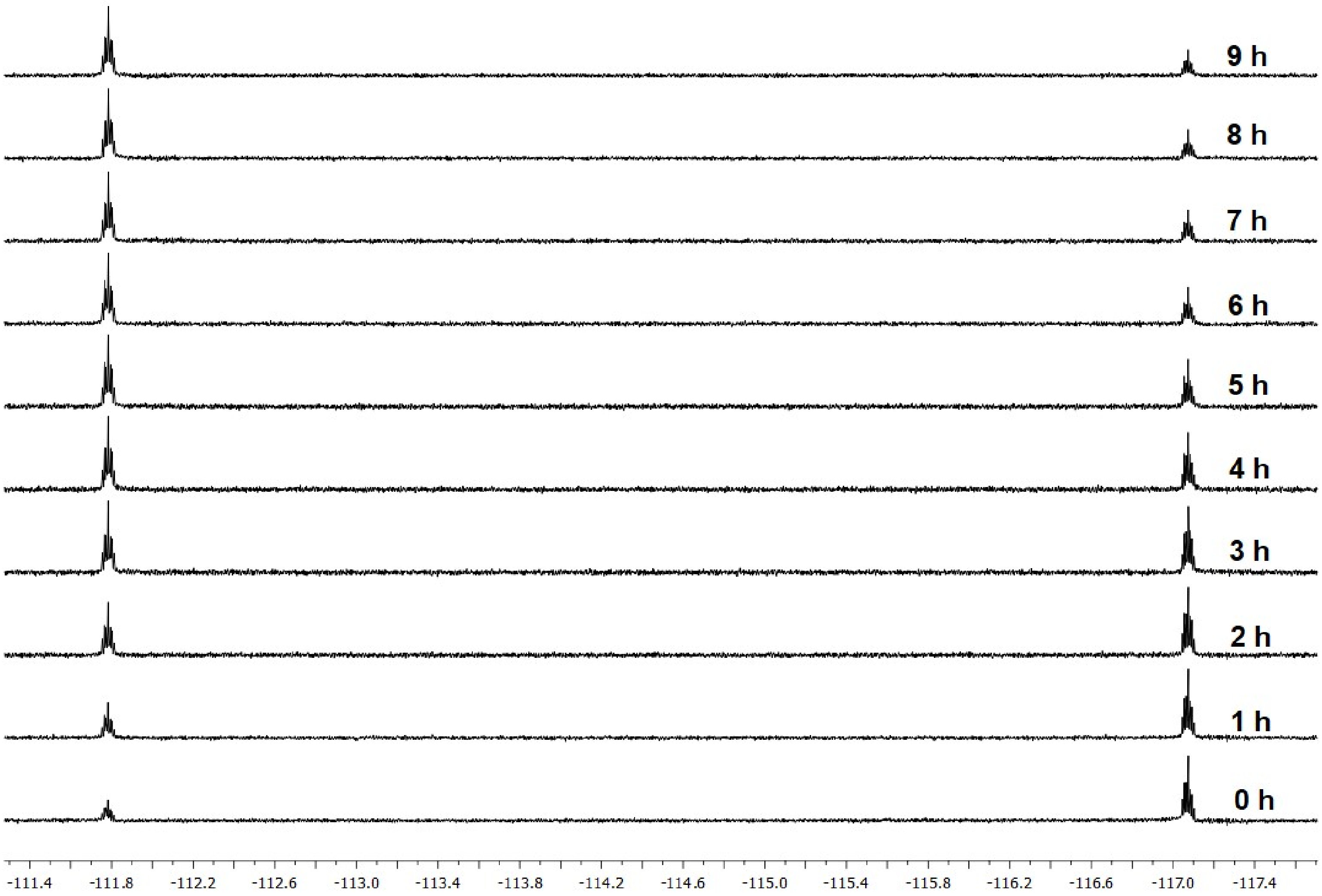
9 h
8 h
7 h
6 h
5 h
4 h
3 h
2 h
1 h
0 h
-111.4
-111.8
-112.2
-112.6
-113.0
-113.4
-113.8
-114.2
-114.6
-115.0
-115.4
-115.8
-116.2
-116.6
-117.0
-117.4

9e:

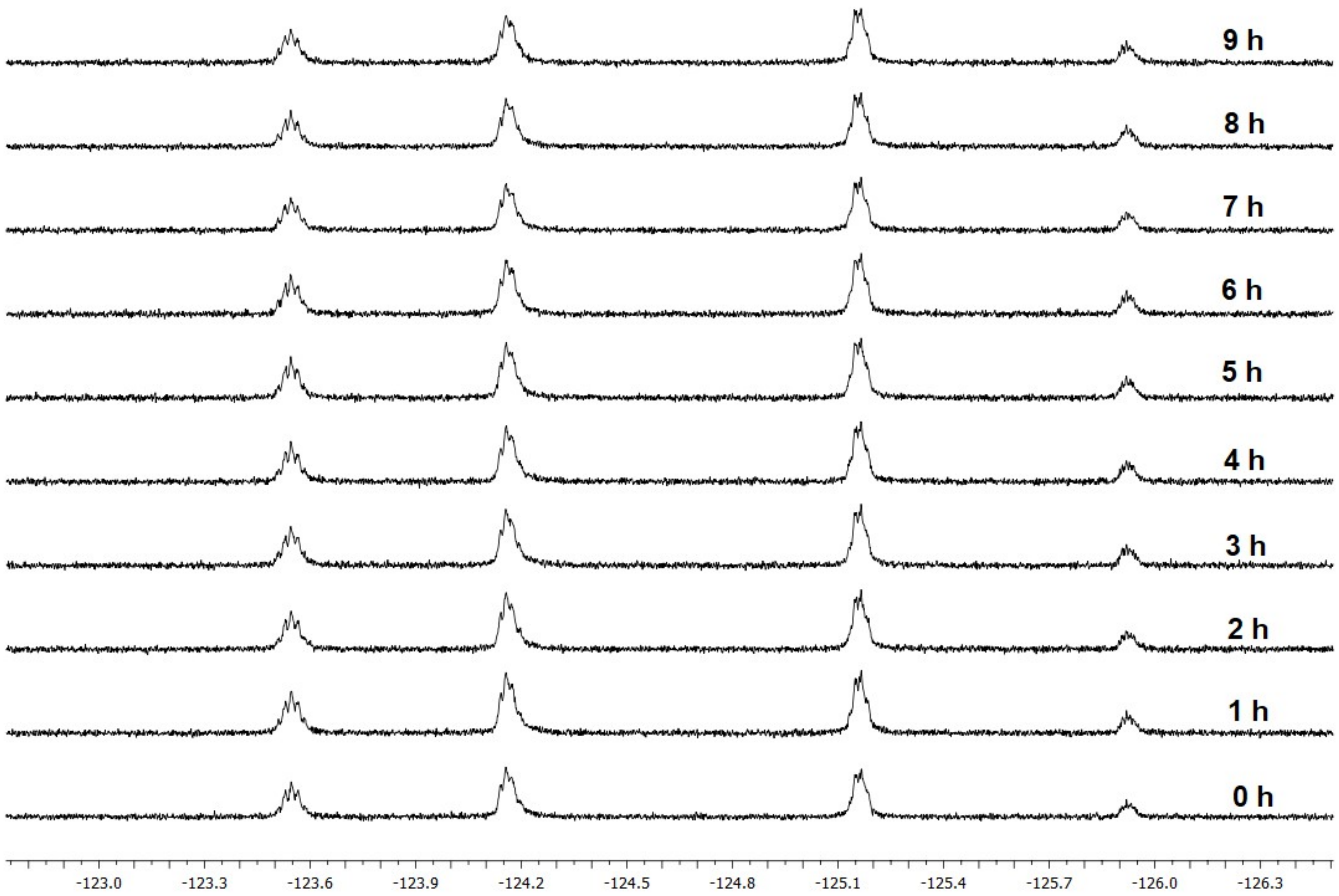
9 h
8 h
7 h
6 h
5 h
4 h
3 h
2 h
1 h
0 h
-123.0
-123.3
-123.6
-123.9
-124.2
-124.5
-124.8
-125.1
-125.4
-125.7
-126.0
-126.3

Mas spectra of product(s) 6a:

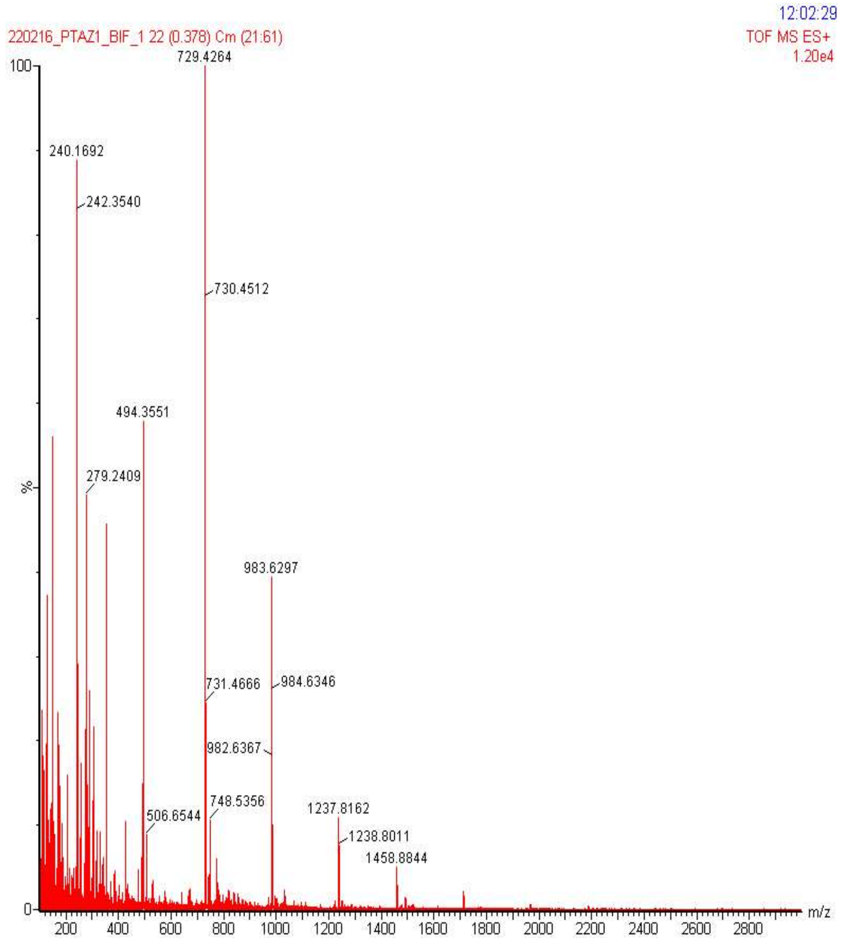

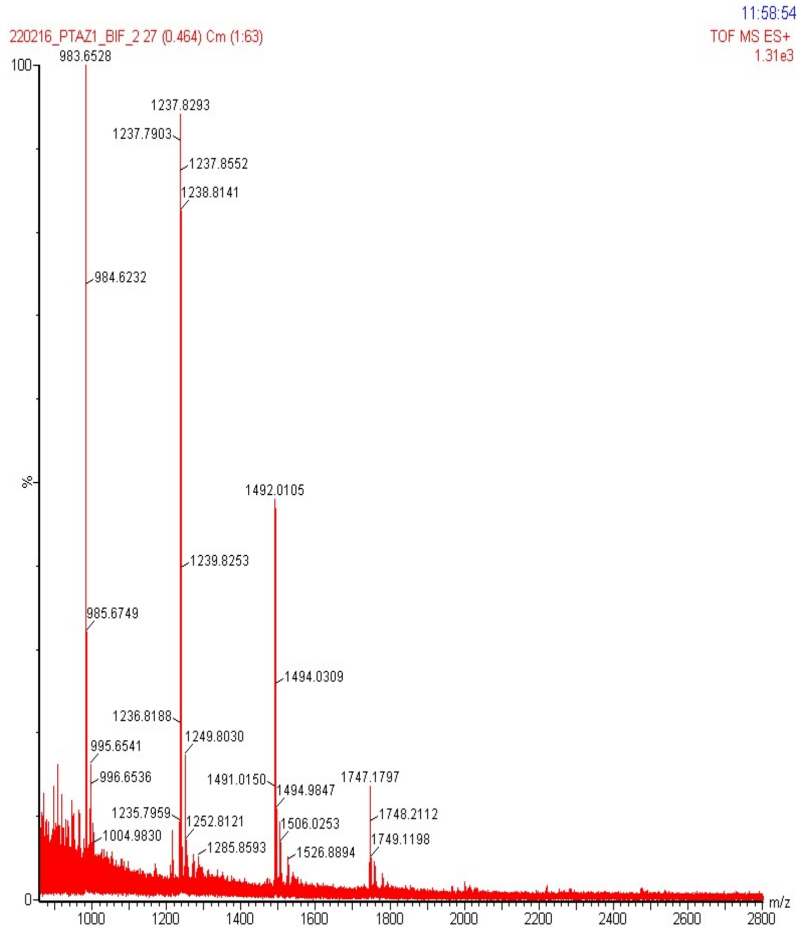

Proposed structures:

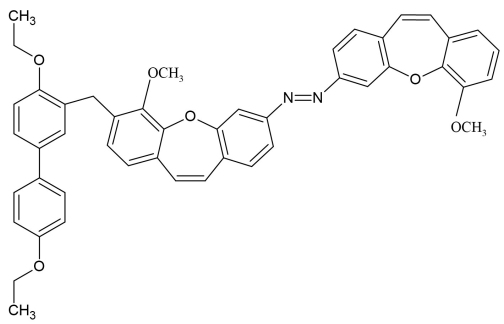

729.4264

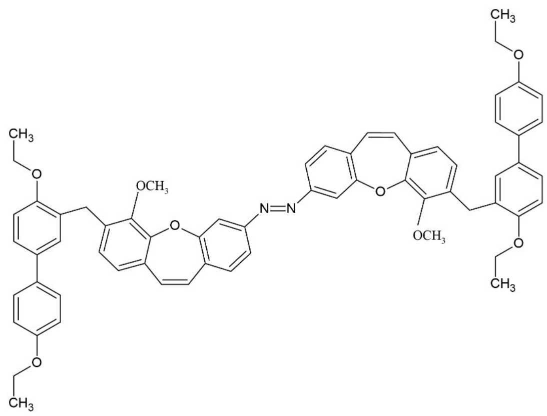

983.6297

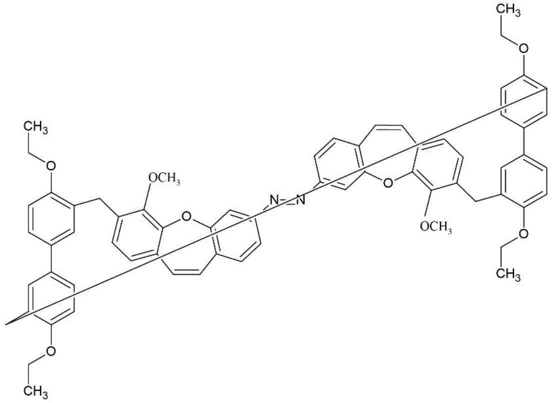

995.6541

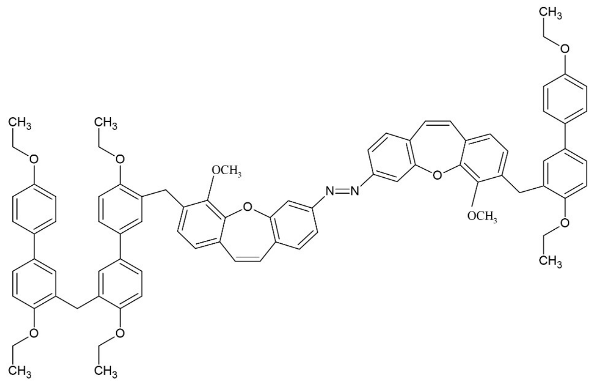

1237.8293

UV-VIS spectra of 7b and 9f *E/Z* isomers

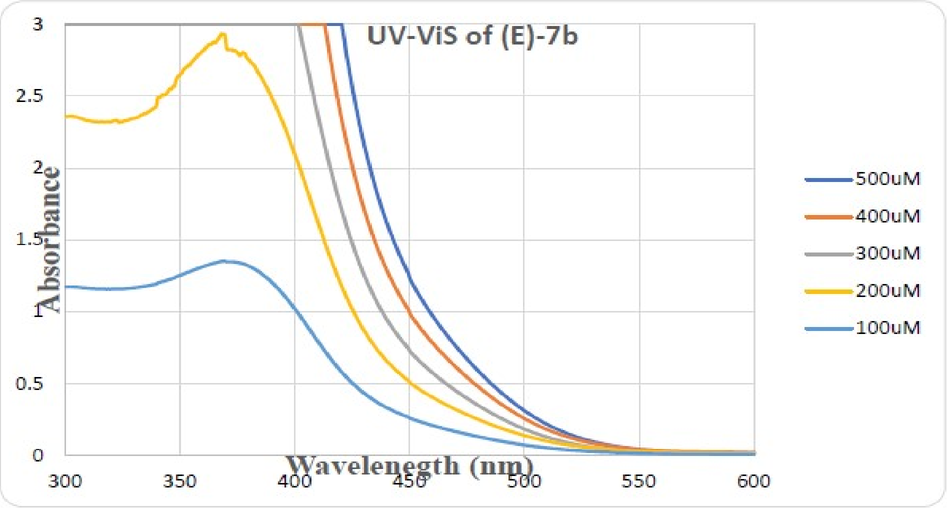

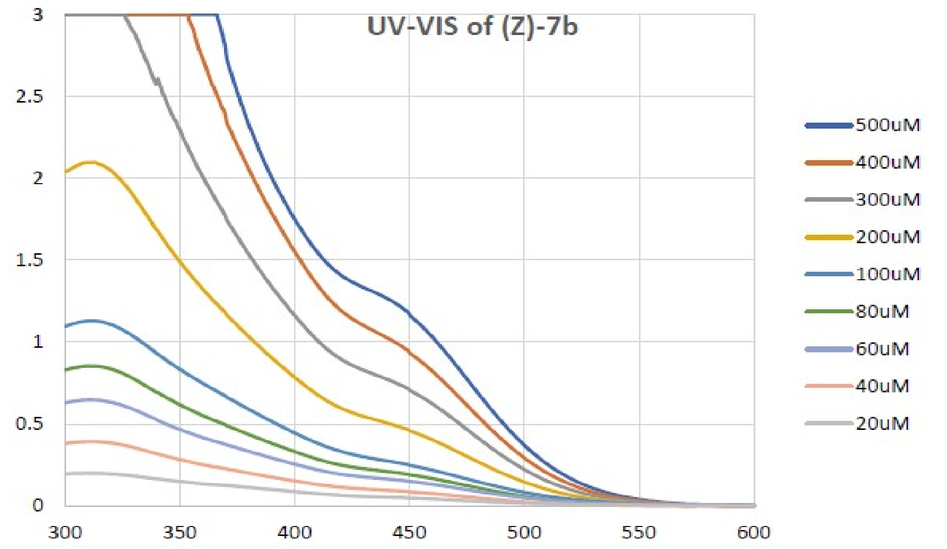

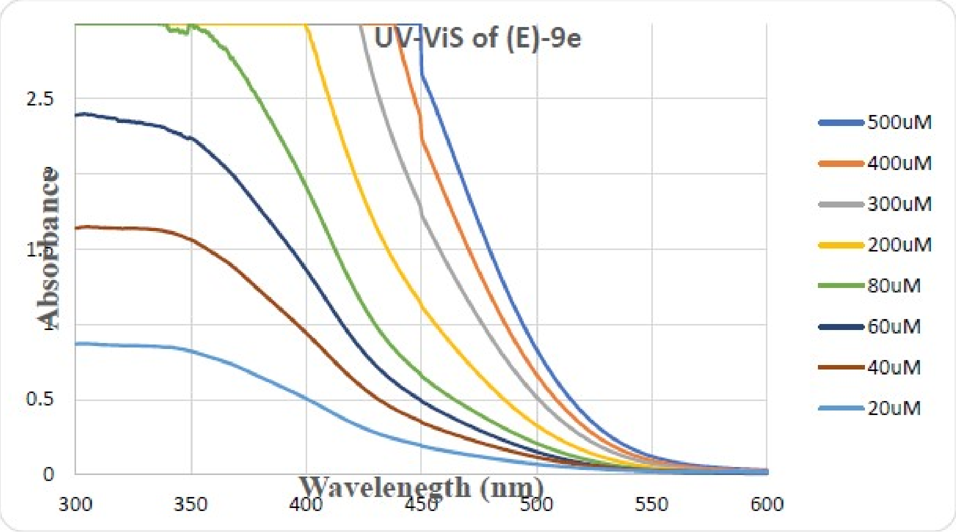
UV-ViS of (E)-9e
Absorbance
Wavelenegth (nm)
0
0.5
1
1.5
2
2.5
300
350
400
450
500
550
600
500uM
400uM
300uM
200uM
80uM
60uM
40uM
20uM

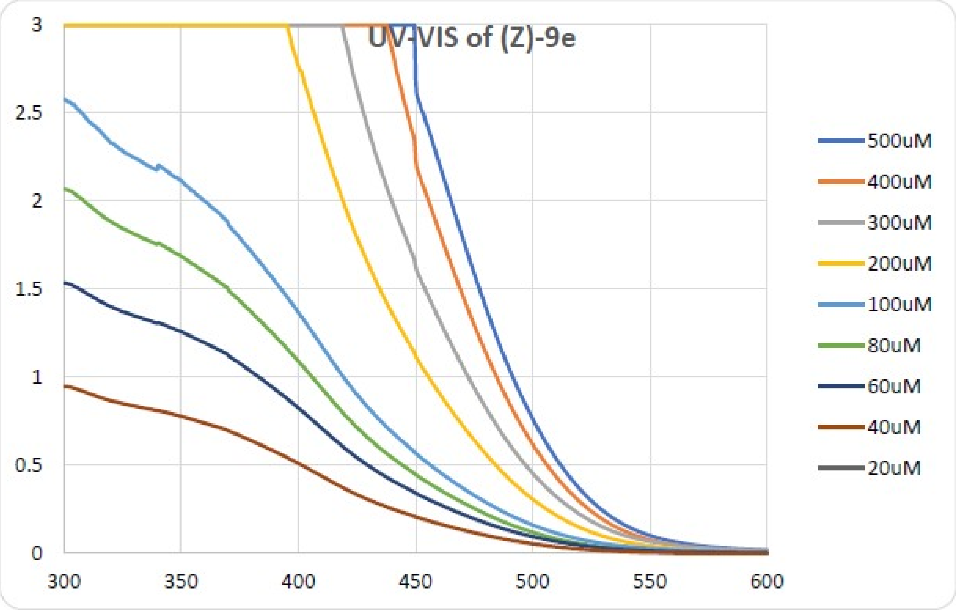
UV-VIS of (Z)-9e
0
0.5
1
1.5
2
2.5
3
300
350
400
450
500
550
600
500uM
400uM
300uM
200uM
100uM
80uM
60uM
40uM
20uM

References

----

[1] M.J. Frisch, G.W. Trucks, H.B. Schlegel, G.E. Scuseria, M.A. Robb, J.R. Cheeseman, J.A. Montgomery, T. Vreven, K.N. Kudin, J.C. Burant, J.M. Millam, S.S. Iyengar, J.Tomasi, V. Barone, B. Mennucci, M. Cossi, G. Scalmani, N. Rega, G.A. Petersson, H. Nakatsuji, M. Hada, M. Ehara, K. Toyota, R. Fukuda, J. Hasegawa, M. Ishida, T.Nakajima, Y. Honda, O. Kitao, H. Nakai, M. Klene, X. Li, J.E. Knox, H.P. Hratchian, J.B. Cross, V. Bakken, C. Adamo, J. Jaramillo, R. Gomperts, R.E. Stratmann, O. Yazyev, A.J. Austin, R. Cammi, C. Pomelli, J.W. Ochterski, P.Y. Ayala, K. Morokuma, G.A. Voth, P. Salvador, J.J. Dannenberg, V.G. Zakrzewski, S. Dapprich, A.D. Daniels, M.C. Strain, O. Farkas, D.K. Malick, A.D. Rabuck, K.Raghavachari, J.B. Foresman, J.V. Ortiz, Q. Cui, A.G. Baboul, S. Clifford, J. Cioslowski, B.B. Stefanov, G. Liu, A. Liashenko, P. Piskorz, I. Komaromi, R.L. Martin, D.J. Fox, T. Keith, M.A. Al-Laham, C.Y. Peng, A. Nanayakkara, M. Challacombe, P.M.W. Gill, B. Johnson, W. Chen, M.W. Wong, C. Gonzalez, J.A. Pople, Gaussian Inc., Wallingford, CT, **2004** (Gaussian 03, Revision E.01).

[2] J. Tomasi, B. Mennucci, R. Cammi, Quantum mechanical continuum solvation models, *Chem. Rev*. 2005, *105*, 2999.

[3] RCSB Protein Data Bank—RCSB PDB. Available online: http://www.rcsb.org/pdb/home/home.do (accessed on 23 February **2004**).

[4] Trott, O.; Olson, A.J. AutoDock Vina: Improving the speed and accuracy of docking with a new scoring function, efficient optimization and multithreading. *J. Comput. Chem.* **2010**, *31*, 455–461.

[5] Pettersen, E.F.; Goddard, T.D.; Huang, C.C.; Couch, G.S.; Greenblatt, D.M.; Meng, E.C.; Ferrin, T.E. UCSF Chimera-a visualization system for exploratory research and analysis. *J. Comput. Chem*. **2004**, *25*, 1605–1612.

[6] F. Borys, P. Tobiasz, E. Joachimiak, H. Fabczak, H. Krawczyk, Systematic studies on anti-cancer evaluation of stilbene and dibenzo[*b,f*]oxepine derivatives. *Molecules* 2023, *2023*, 28, 3558, https://doi.org/10.3390/ molecules28083558.
